# Access to spirocyclic vinyl sulfones *via* radical cyclization and functional group migration†

**DOI:** 10.1039/d5sc02555a

**Published:** 2025-04-29

**Authors:** Shan Yang, Yasu Chen, Chen Zhu

**Affiliations:** a Frontiers Science Center for Transformative Molecules, School of Chemistry and Chemical Engineering, State Key Laboratory of Synergistic Chem-Bio Synthesis, Shanghai Key Laboratory for Molecular Engineering of Chiral Drugs, Shanghai Jiao Tong University 800 Dongchuan Road Shanghai 200240 China chzhu@sjtu.edu.cn

## Abstract

Spirocyclic vinyl sulfones, which incorporate the three-dimensional structure inherent to spiro compounds and the Michael acceptor reactivity associated with vinyl sulfones, hold promise for novel biological activities. The lack of efficient synthetic methods, however, hinders their extensive investigations in drug discovery and development. In this work, we describe a practical and versatile approach for the synthesis of multi-functionalized spirocyclic vinyl sulfones from easily available materials. The reaction proceeds efficiently through a cascade of radical cyclization followed by (hetero)aryl migration. The protocol features mild photocatalytic conditions and provides access to a diverse range of products, enabling the construction of complex scaffolds, including medium-sized ring-fused spirocyclic vinyl sulfones.

## Abstract 2

A practical approach for the synthesis of multi-functionalized spirocyclic vinyl sulfones is disclosed, which proceeds *via* consecutive radical cyclization and (hetero)aryl migration.

## Graphical abstract

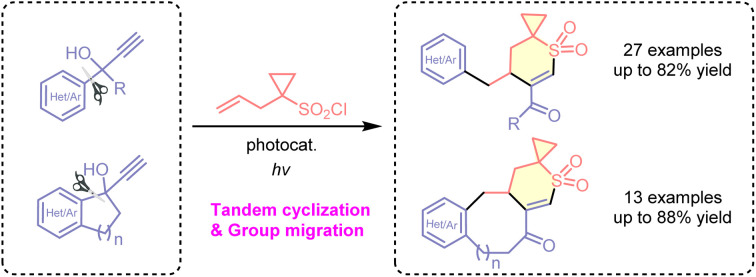

## Introduction

**Scheme 1:**
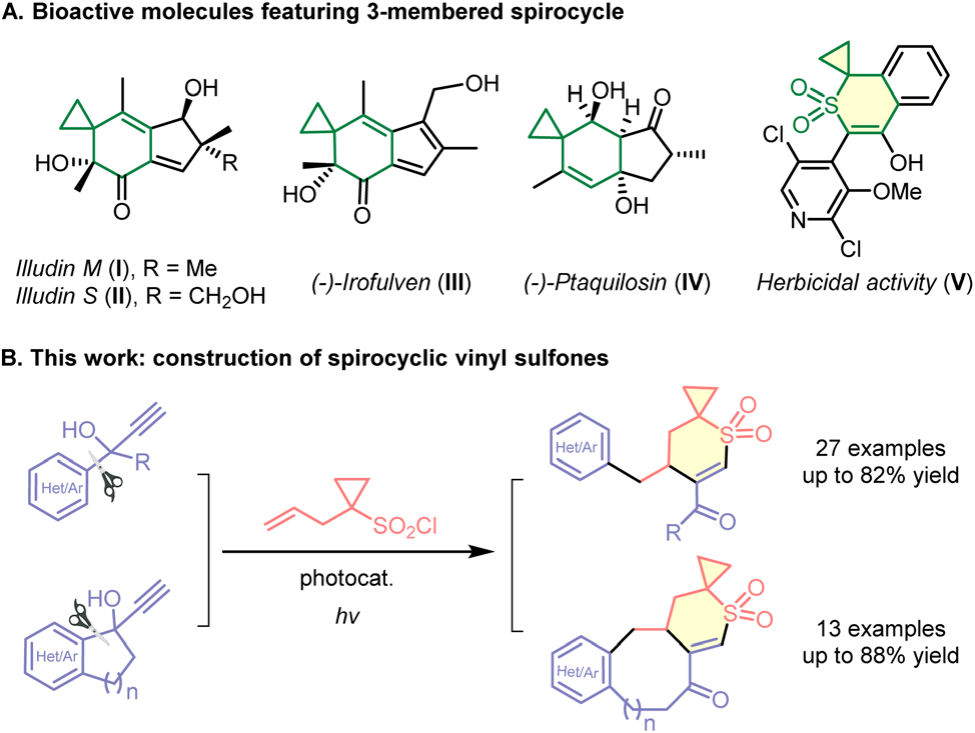
Importance and practical synthesis of spirocyclic vinyl sulfones.

One widely employed strategy in drug design is to increase the complexity of molecular architectures by introducing a greater number of sp^3^-hybridized carbons and rings, which serves to rigidify the ligand's conformation.^1^ Spirocyclic compounds, distinguished by their unique and rigid three-dimensional geometry and composed of two rings connected through a single spiroatom, have been receiving increasing interest from both synthetic and medicinal chemists.^2^ The non-planar conformations that these compounds often adopt can lead to improved binding affinity and enhanced selectivity for biological targets, such as proteins. Spirocyclic systems incorporating a three-membered ring are notable for their capacity to improve pharmacokinetic and pharmacodynamic profiles, rendering them versatile building blocks in medicinal chemistry.^3^ For instance (Scheme 1A), illudin M (I)^4^ and illudin S(ii),^5^ natural sesquiterpenoids with antitumor, antiviral, and genotoxic activities, are key precursors in the development of anticancer drugs; irofulven (III),^6^ a semi-synthetic analog of the illudin class, has advanced to clinical trials for the treatment of solid tumors such as prostate cancer, pancreatic cancer, and ovarian cancer; ptaquilosin (IV) demonstrates selective toxicity across species in NCI antitumor screening assays,^7^ exhibiting notable activity against human myeloid leukemia and a range of carcinoma cell lines.

Vinyl sulfones represent valuable Michael acceptors in drug design, owing to their reactivity towards nucleophilic species.^8^ The incorporation of both a vinyl sulfone moiety and a three-membered spirocyclic subunit within a single molecular framework has the potential to unlock unique biological activities. For example (Scheme 1A), compound V demonstrates notable herbicidal efficacy at lower dosages while exhibiting favorable compatibility with crops, positioning it as a promising candidate for commercial agrochemical development.^9^ Nevertheless, the absence of efficient synthetic routes to access spirocyclic vinyl sulfones limits the extensive exploration of their physicochemical and physiological attributes.^10^

In this report, we describe a versatile and operationally simple method for the synthesis of multi-functionalized spirocyclic vinyl sulfones, employing inexpensive allylcyclopropane sulfonyl chloride and easily accessible tertiary propargyl alcohols as starting materials (Scheme 1B). The photocatalyzed reaction proceeds *via* a cascade process involving radical cyclization followed by (hetero)aryl migration,^11,12^ driven by visible-light irradiation. Notably, the use of cyclic tertiary propargyl alcohols facilitates the efficient construction of complex medium-sized ring-fused spirocyclic frameworks through ring expansion, structures that are otherwise difficult to synthesize.

## Results and discussion

The investigations were performed under blue LED light irradiation at room temperature, using benzothiazole-substituted propargyl alcohol 1a and commercially available allylcyclopropane sulfonyl chloride 2 as substrates (Table 1). A range of photocatalysts were screened, with *fac*-Ir(ppy)_3_ demonstrating the best catalytic activity (Entries 1–5). Considering that HCl is the only byproduct generated during the reaction, the addition of one equivalent of base to neutralize the strong acid is essential for successful transformation. Among several organic and inorganic bases tested (Entries 5–8), Na_2_HPO_4_ was identified as the optimal one. The inclusion of a small quantity of H_2_O as a co-solvent enhanced the solubility of Na_2_HPO_4_ within the reaction system, thereby promoting the conversion. Subsequent evaluation of various organic solvents revealed that the reaction carried out in DCM/H_2_O provided the desired product 3a with a favorable yield (Entries 9–13). Furthermore, control experiments demonstrated that the reaction was completely inhibited in the absence of a photocatalyst or upon exclusion of light (Entries 14 and 15), and the yield was compromised when the reaction was performed in air or without the base (Entries 16 and 17).

**Table 1:** Reaction conditions survey

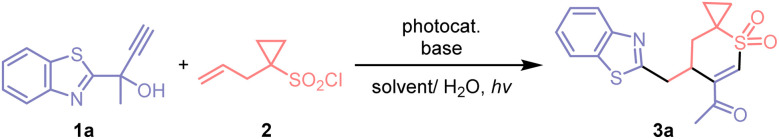				
Entry^a^	Photocatalyst	Base	Solvent	Yield b(%)
1	Eosin Y	Na_2_HPO_4_	CH_3_CN	7
2	Ru(bpy)_3_Cl_2_·6H_2_O	Na_2_HPO_4_	CH_3_CN	13
3	Mes-Acr^+^ClO_4_^−^	Na_2_HPO_4_	CH_3_CN	16
4	4CzIPN	Na_2_HPO_4_	CH_3_CN	30
5	*fac*-Ir(ppy)_3_	Na_2_HPO_4_	CH_3_CN	41
6	*fac*-Ir(ppy)_3_	NaH_2_PO_4_	CH_3_CN	36
7	*fac*-Ir(ppy)_3_	Na_3_PO_4_	CH_3_CN	39
8	*fac*-Ir(ppy)_3_	DIPEA	CH_3_CN	29
9	*fac*-Ir(ppy)_3_	Na_2_HPO_4_	DCM	74
10	*fac*-Ir(ppy)_3_	Na_2_HPO_4_	MeOH	23
11	*fac*-Ir(ppy)_3_	Na_2_HPO_4_	DMSO	Trace
12	*fac*-Ir(ppy)_3_	Na_2_HPO_4_	PhCF_3_	38
13	*fac*-Ir(ppy)_3_	Na_2_HPO_4_	EtOAc	43
14	—	Na_2_HPO_4_	DCM	0
15^c^	*fac*-Ir(ppy)_3_	Na_2_HPO_4_	DCM	0
16^d^	*fac*-Ir(ppy)_3_	Na_2_HPO_4_	DCM	39
17	*fac*-Ir(ppy)_3_	—	DCM	58

a Standard conditions: 1a (0.2 mmol), 2a (0.3 mmol), base (0.2 mmol), and photocatalyst (3 mol%) in solvent/H_2_O (*v*/*v* 2 mL/0.2 mL), irradiated with 12 W blue LEDs at room temperature under N_2_.

b Yields of isolated products are given.

c In dark.

d Under air.

**Scheme 2:**
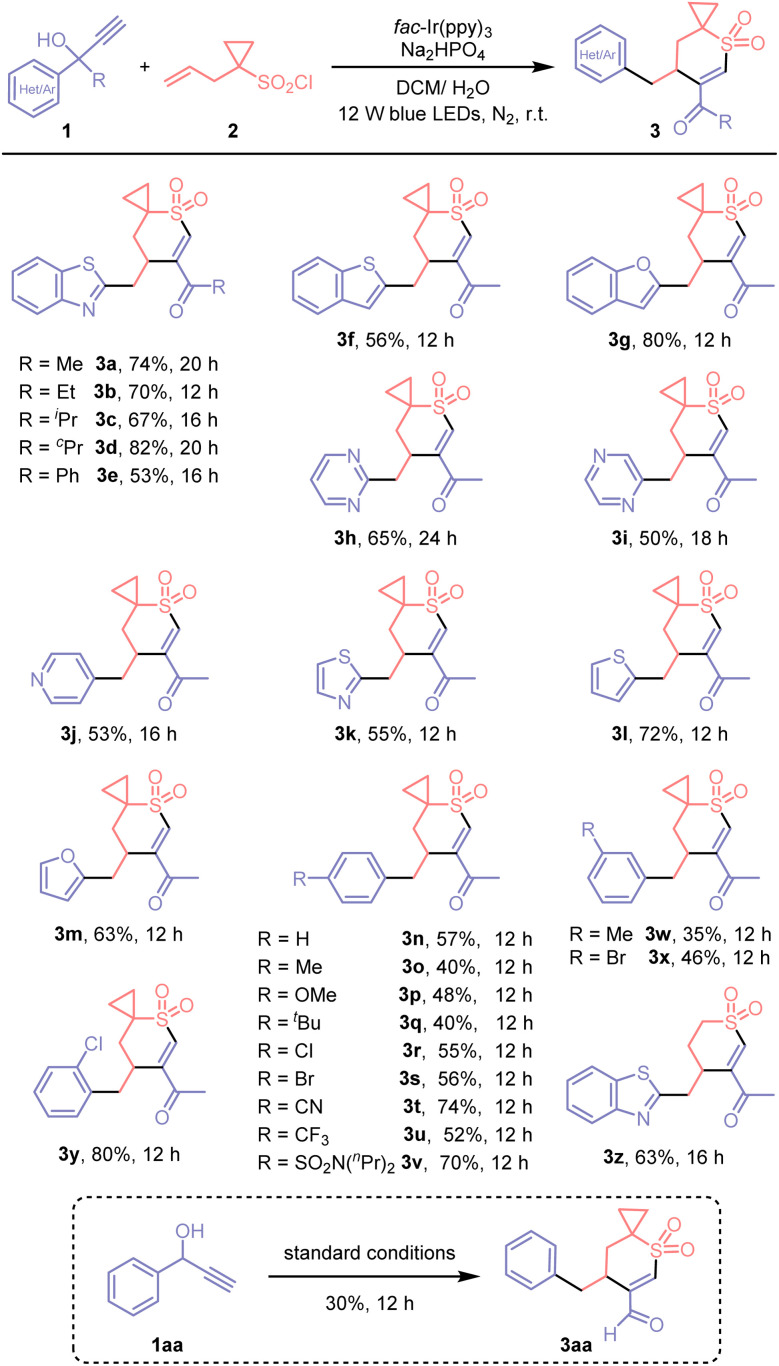
Demonstration of broad functional group compatibility. Reaction conditions: 1 (0.2 mmol), 2 (0.3 mmol), Na_2_HPO_4_ (0.2 mmol), and *fac*-Ir(ppy)_3_ (3 mol%) in DCM/H_2_O (*v*/*v* 2 mL/0.2 mL), irradiated with 12 W blue LEDs at room temperature under N_2_. Yields of isolated products are given.

With the optimized reaction conditions in hand, we investigated the compatibility of various functional groups by testing a broad range of tertiary propargyl alcohols (Scheme 2). First, we examined the impact of aliphatic substituents of different sizes surrounding the migrating group, showing that steric hindrance did not significantly impede reaction efficiency (3a–3d). A slight decrease in yield was observed with the phenyl substituent (3e). Beyond benzothiazolyl, a range of five-membered heteroaryl groups, including benzothienyl, benzofuryl, thiazolyl, thienyl, and furyl, as well as six-membered heteroaryls such as pyrimidyl, pyrazinyl, and pyridyl, demonstrated sufficient migratory ability, resulting in the formation of the corresponding products (3f–3m) in synthetically useful yields. Moreover, a diversity of functionalized phenyl groups readily participated in the migration process (3n–3y). Substrates featuring substitution on the benzene ring at the *para*, *meta*, or *ortho* positions were all competent to generate the desired products. Both electron-donating groups (*e.g.*, Me, OMe, ^*t*^Bu) and electron-withdrawing groups (*e.g.*, halo, CN, CF_3_, SO_2_R) proved compatible with the reaction conditions; the latter typically afforded enhanced yields, which can be attributed to the propensity of electron-deficient arenes to undergo migration promoted by nucleophilic alkyl radicals. While *m*-substituted substrates provided comparatively lower yields (3w and 3x), the *o*-chloro substrate afforded the product in high yield (3y). Furthermore, allylcyclopropane sulfonyl chloride 2 could be successfully substituted with homoallylic sulfonyl chloride, providing the desired product in a useful yield (3z). Besides tertiary propargyl alcohols, secondary propargyl alcohol (1aa) was also amenable to the formation of spirocyclic vinyl sulfone (3aa) bearing a formyl group, albeit with a low yield.

**Scheme 3:**
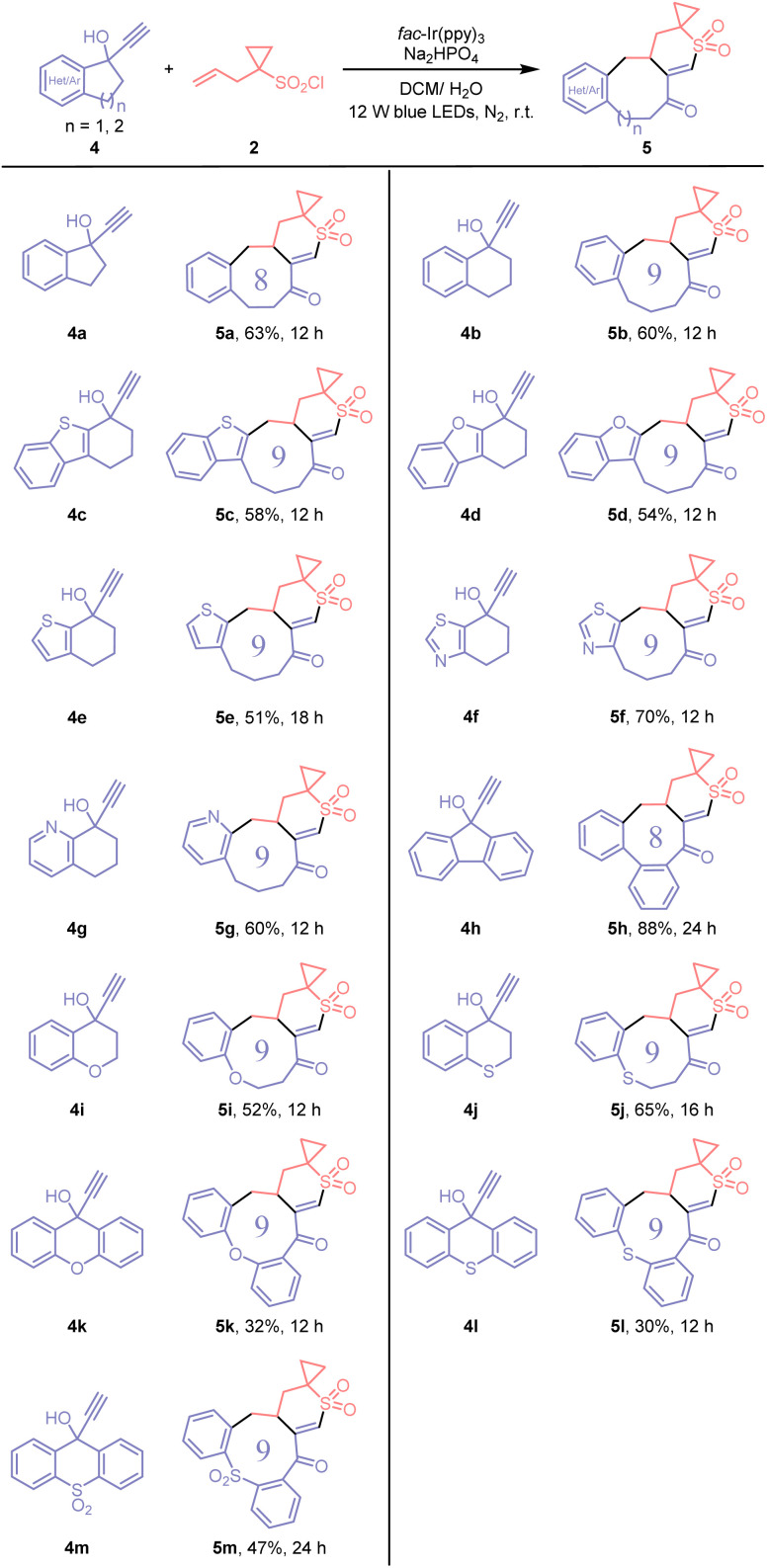
Construction of complex ring-fused spirocyclic vinyl sulfones. Reaction conditions: 4 (0.2 mmol), 2 (0.3 mmol), Na_2_HPO_4_ (0.2 mmol), and *fac*-Ir(ppy)_3_ (3 mol%) in DCM/H_2_O (*v*/*v* 2 mL/0.2 mL), irradiated with 12 W blue LEDs at room temperature under N_2_. Yields of isolated products are given.

This protocol is applicable to the construction of structurally complex ring-fused spirocyclic vinyl sulfones *via* ring expansion (Scheme 3). When cyclic propargyl alcohols are employed, aryl or heteroaryl group migration proceeds concomitantly with ring expansion. This enables facile editing of cyclic skeletons through the addition of three carbons, transforming five- and six-membered rings into eight- and nine-membered rings.^13^ The reaction with the substrates (4a and 4b) derived from indanone and tetralone gave rise to the corresponding benzo-octanone and nonanone-fused products (5a and 5b). Likewise, this approach proved applicable to heteroaryl-fused analogues (4c–4g), facilitating the efficient migration of benzothienyl, benzofuryl, thienyl, thiazolyl and pyridyl moieties to afford the corresponding products (5c–5g). The substrate 4h, obtained from fluorenone, was also suitable, affording a more structurally elaborate product containing five cyclic units in a notably high yield (5h). The presence of heteroatoms, such as O or S atoms, within the substrates did not hinder the reaction outcomes. For example, substrates 4i–4m, synthesized from chromanone, thiochromanone, xanthenone, and thioxanthenone precursors, were all amenable to furnishing the corresponding products in synthetically useful yields (5i–5m).

**Scheme 4:**
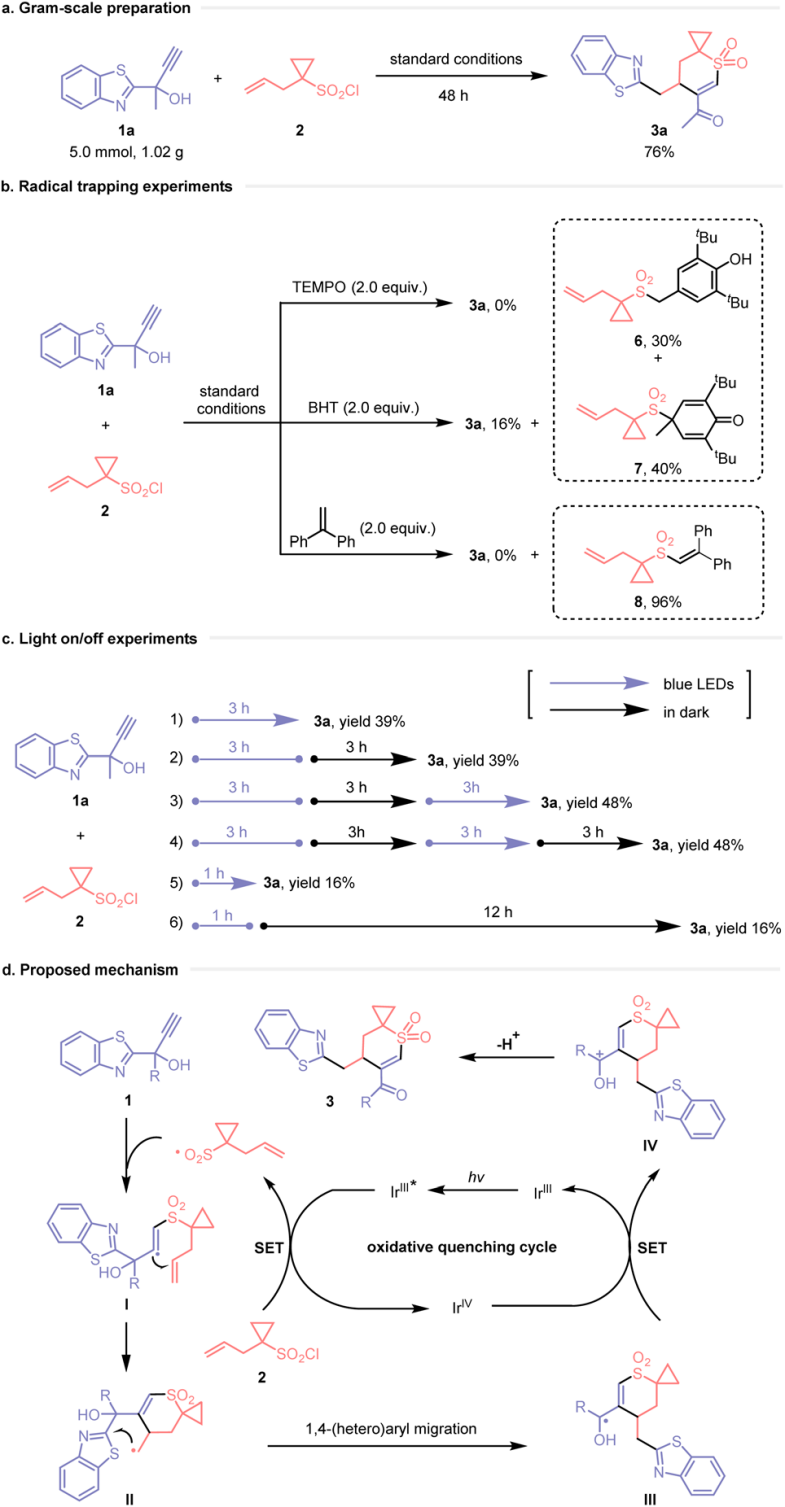
Gram-scale preparation, mechanistic studies and proposed mechanism.

Gram-scale preparation of 3a was successfully performed without compromising the yield, demonstrating the practicality of the method (Scheme 4a). To gain deeper insight into the reaction mechanism, a series of experiments was conducted. The addition of stoichiometric amounts of the radical scavenger 2,2,6,6-tetramethyl-1-piperidinyloxy (TEMPO) completely inhibited the formation of the target product 3a. When 2,6-di-*tert*-butyl-4-methylphenol (BHT) and 1,1-diphenylethylene were used as trapping reagents, the corresponding adducts (6, 7, and 8) resulting from the interception of sulfonyl radicals were isolated and characterized. This result confirmed the involvement of free-radical species in the transformation, which was initiated by a sulfonyl radical (Scheme 4b). Furthermore, light on/off experiments demonstrated that product formation occurred only during periods of continuous light irradiation, suggesting the predominance of a photocatalytic pathway (Scheme 4c). The quantum yield measurement (*Φ* = 0.81) indicated that the reaction proceeded primarily *via* a photocatalytic pathway, although the contribution of a radical chain process could not be ruled out (for details, see the ESI†).

A plausible reaction mechanism is depicted in Scheme 4d. Initially, upon irradiation with blue LED light, the excited photocatalyst Ir^III^* reduces sulfonyl chloride 2*via* single-electron transfer (SET), generating a sulfonyl radical and an Ir^IV^ species. This sulfonyl radical then adds to the alkynyl group of propargyl alcohol 1, resulting in vinyl radical intermediate I. Subsequently, addition of the vinyl radical to the distal alkene through a six-membered cyclic transition state forms the highly reactive primary alkyl radical II. This intermediate undergoes intramolecular 1,4-(hetero)aryl migration *via* a five-membered cyclic transition state, leading to the formation of ketyl radical intermediate III. Oxidation of III to cation IV by the Ir^IV^ species simultaneously regenerates the Ir^III^ species, perpetuating the photocatalytic cycle. Finally, deprotonation of cation IV affords the desired product 3.

## Conclusions

In summary, we have disclosed a novel photocatalytic approach to access spirocyclic vinyl sulfones *via* tandem radical cyclization and functional group migration processes. The reaction features mild conditions and a broad functional group compatibility. A diverse array of complex scaffolds, including medium-sized ring-fused spirocyclic vinyl sulfones, can be readily generated in synthetically useful yields. Evaluation of the biological activities of these compounds is ongoing in our laboratories. This protocol not only facilitates the synthesis of spirocyclic vinyl sulfones that are otherwise difficult to access but also expands their potential applications in medicinal chemistry.

## Data availability

Data supporting the manuscript are provided in the ESI,† including the experimental methods, compound characterization, and NMR spectra for this study.

## Author contributions

C. Z. conceived of and directed the project, S. Y. and Y. C. conducted the experiments and analyzed the data, S. Y. and C. Z. wrote the manuscript.

## Conflicts of interest

There are no conflicts to declare.

## Supplementary Material

SC-OLF-D5SC02555A-s001
