# Supplementary material for: Access to spirocyclic vinyl sulfones via radical cyclization and functional group migration
Source: Chem Sci. 2025 Apr 29;16(22):9715–9. doi: 10.1039/d5sc02555a (PMC12044546; doi:10.1039/d5sc02555a)
Supplement: SC-016-D5SC02555A-s001 [file SC-016-D5SC02555A-s001.pdf]

Supplementary Information (SI) for Chemical Science.
This journal is © The Royal Society of Chemistry 2025

Supporting Information

Access to Spirocyclic Vinyl Sulfones via Radical Cyclization and Functional Group Migration

Shan Yang, Yasu Chen, and Chen Zhu*

[*] Frontiers Science Center for Transformative Molecules, School of Chemistry and Chemical Engineering, State Key Laboratory of Synergistic Chem-Bio Synthesis, and Shanghai Key Laboratory for Molecular Engineering of Chiral Drugs, Shanghai Jiao Tong University, 800 Dongchuan Road, Shanghai 200240, China

Email: chzhu@sjtu.edu.cn

Table of Contents

1. General experimental details S2
2. General procedure for the synthesis of spirocyclic vinyl sulfones S2
3. Synthesis of starting materials S3
4. Characterization of new starting materials and products S3
5. Mechanistic studies S19
6. ^{1}H, ^{13}C, and ^{19}F NMR spectra S23

1. General experimental details

All reactions were maintained under nitrogen unless otherwise stated. Commercially available reagents were used without further purification. Infrared (FT-IR) spectra were recorded on a BRUKER VERTEX 70, ν_{max} in cm^{-1}. ^{1}H-NMR spectra were recorded on a BRUKER AVANCE III HD (400 MHz) spectrometer and a BRUKER AVANCE III HD (500 MHz) spectrometer. Chemical shifts are reported in ppm from tetramethylsilane with the solvent resonance as internal standard ($CDCl_3$: δ 7.26). Data are reported as follows: chemical shift, multiplicity (s = singlet, d = doublet, t = triplet, q = quadruplet, br = broad, m = multiplet), coupling constants (Hz) and integration. ^{13}C-NMR spectra were recorded on a Bruker AVANCE III HD (100 or 125 MHz) spectrometer with complete proton decoupling. Chemical shifts are reported in ppm from tetramethylsilane with the solvent resonance as the internal standard ($CDCl_3$: δ 77.16). ^{19}F-NMR spectra were recorded on a BRUKER AVANCE III HD (376 MHz) spectrometer. High resolution mass spectrometry (HRMS) was measured with a GCT Premier^{TM} and BRUKER micrOTF-Q III. Melting points were measured using INESA WRR and values are uncorrected.

Propargyl alcohol **1aa** and allylcyclopropane sulfonyl chloride **2** are commercially available reagents.

2. General procedure for the synthesis of spirocyclic vinyl sulfones

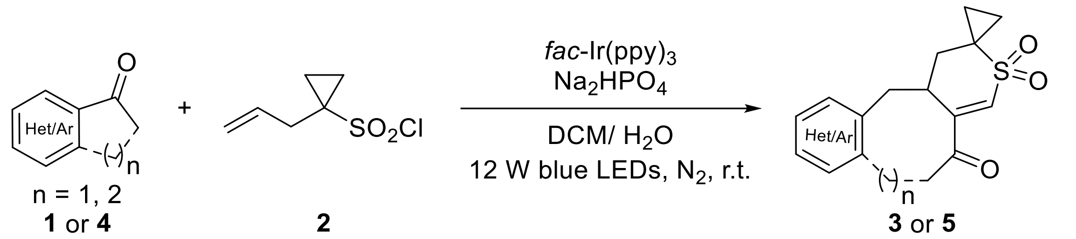

Propargyl alcohol **1** or **4** (0.2 mmol), Na_2HPO_4 (0.2 mmol), and *fac*-Ir(ppy)$_3$ (0.006 mmol, 3 mol %) were loaded in a reaction vial which was subjected to evacuation/ flushing with N_2 three times. Then allylcyclopropane sulfonyl chloride **2** (0.3 mmol) in CH_3CN (2 mL) and H_2O (0.2 mL) were added to the mixture via syringe. The reaction was irradiated with 12 W blue LEDs for about 12 hours. After reaction completion, the reaction mixture was directly concentrated in vacuo. Purification the residue by flash column chromatography on silica gel afforded the desired product **3** or **5**.

Gram-scale preparation:
Propargyl alcohol **1a** (5.0 mmol, 1.016 g), Na_2HPO_4 (5.0 mmol, 0.710 g), and *fac*-Ir(ppy)$_3$ (3 mol %, 100 mg) were loaded in a 100 mL round-bottom flask which was subjected to evacuation/ flushing with N_2 three times. Then allylcyclopropane sulfonyl chloride (7.5 mmol, 1.355 g) in CH_3CN (50 mL) and H_2O (5 mL) were added to the mixture. The reaction was irradiated with 12 W blue LEDs for 48 hours. After reaction completion, the reaction mixture was extracted with EtOAc, and the organic layers were

dried over anhydrous Na_2SO_4, and concentrated in vacuo. Purification the residue by flash column chromatography on silica gel afforded the desired product **3a** (76 % yield, 1.3203 g).

3. Synthesis of starting materials

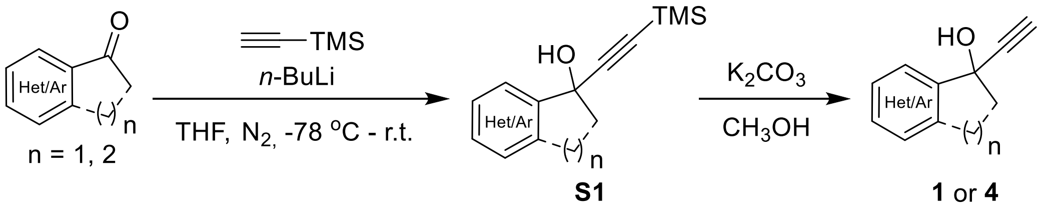

First step: To a solution of trimethylsilylacetylene (5.5 mmol, 1.1 equiv.) in anhydrous THF (11 mL, 0.5 M) was slowly added *n*-butyllithium (5.5 mmol, 1.1 equiv.) at -78 °C, then the mixture was stirred for 30 min at room temperature. Then ketone (5.0 mmol, 1.0 equiv.) in anhydrous THF (5 mL) was added dropwise via syringe and the mixture was stirred at room temperature for another 1 h. After reaction completion, the mixture was quenched by H_2O and extracted with EtOAc for three times. The combined organic extracts were washed by brine, dried over $MgSO_4$, filtered, concentrated, and purified by flash column chromatography on silica gel to give **S1**.

Second Step: A solution of **S1** (4.0 mmol, 1.0 equiv.) in CH_3OH (8 mL,0.5 M) was added K_2CO_3 (4.8 mmol, 1.2 equiv.) and stirred for 1 h at room temperature. After reaction completion, the crude reaction mixture was extracted with EtOAc, and the organic layers were dried over anhydrous Na_2SO_4, and concentrated in vacuo. Purification the residue by flash column chromatography on silica gel afforded the corresponding propargyl alcohol **1** or **4**.

4. Characterization of new starting materials and products

a. Starting materials

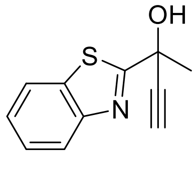

1a: yellow solid, m.p. 162-163 °C. ^1H NMR (400 MHz, $CDCl_3$) δ 8.07-8.03 (m, 1H), 7.91-7.87 (m, 1H), 7.52-7.46 (m, 1H), 7.43-7.37 (m, 1H), 3.94 (br, 1H), 2.73 (s, 1H), 2.02 (s, 3H); ^{13}C NMR (100 MHz, $CDCl_3$) δ 174.9, 152.7, 135.5, 126.3, 125.5, 123.5, 121.8, 84.9, 74.0, 68.9, 31.5. FT-IR: ν (cm^{-1}) 3232, 2212, 1651, 1633, 1364, 1155, 1036, 760. HRMS [ESI] calcd for $C_{11}H_{10}NOS$ $[M+H]^+$ 204.0478, found 204.0477.

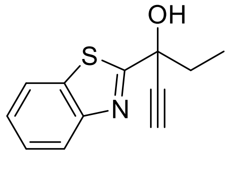

1b: yellow solid, m.p. 154-155 °C. ^1H NMR (500 MHz, $CDCl_3$) δ 8.05 (d, *J* = 8.0 Hz, 1H), 7.89 (d, *J* = 8.0 Hz, 1H), 7.52-7.47 (m, 1H), 7.43-7.38 (m, 1H), 3.80 (br, 1H), 2.74 (s, 1H), 2.31-2.16 (m, 2H), 1.11 (t, *J* = 7.0 Hz, 3H); ^{13}C NMR (100 MHz, $CDCl_3$) δ 174.4,

152.6, 135.5, 126.3, 125.4, 123.4, 121.8, 83.9, 74.8, 72.5, 37.2, 8.3. FT-IR: ν (cm^{-1}) 3242, 2102, 1651, 1633, 1315, 1180, 1057, 758. HRMS [ESI] calcd for $C_{12}H_{12}NOS$ $[M+H]^+$ 218.0643, found 218.0638.

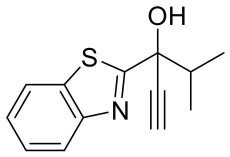

1c: yellow solid, m.p. 122-123 °C. ^1H NMR (400 MHz, $CDCl_3$) δ 8.05 (d, J = 8.0 Hz, 1H), 7.88 (d, J = 8.0 Hz, 1H), 7.52-7.46 (m, 1H), 7.43-7.37 (m, 1H), 3.79 (s, 1H), 2.74 (s, 1H), 2.50-2.39 (m, 1H), 1.14 (d, J = 6.8 Hz, 3H), 1.04 (d, J = 6.4 Hz, 3H); ^{13}C NMR (125 MHz, $CDCl_3$) δ 174.5, 152.4, 135.5, 126.2, 125.4, 123.4, 121.8, 83.4, 75.6, 75.4, 40.2, 17.6, 16.5. FT-IR: ν (cm^{-1}) 3271, 2972, 2116, 1633, 1506, 1381, 1139, 1005. HRMS [ESI] calcd for $C_{13}H_{14}NOS$ $[M+H]^+$ 232.0791, found 232.0792.

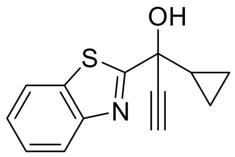

1d: yellow solid, m.p. 157-158 °C. ^1H NMR (400 MHz, d_6-DMSO) δ 8.08 (d, J = 7.6 Hz, 1H), 8.00 (d, J = 8.0 Hz, 1H), 7.54-7.48 (m, 1H), 7.46-7.40 (m, 1H), 7.07 (s, 1H), 3.64 (s, 1H), 1.60-1.52 (m, 1H), 0.71-0.63 (m, 2H), 0.59-0.52 (m, 1H), 0.49-0.42 (m, 1H); ^{13}C NMR (100 MHz, d_6-DMSO) δ 177.4, 153.4, 135.0, 126.6, 125.6, 123.3, 122.7, 83.9, 76.6, 71.4, 22.7, 2.8, 2.2. FT-IR: ν (cm^{-1}) 3289, 3065, 2116, 1636, 1450, 1386, 1155, 1051. HRMS [ESI] calcd for $C_{13}H_{12}NOS$ $[M+H]^+$ 230.0634, found 230.0643.

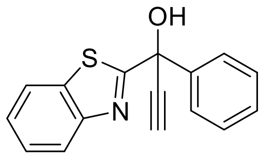

1e: yellow solid, m.p. 172-173 °C. ^1H NMR (500 MHz, d_6-DMSO) δ 8.08-8.05 (m, 1H), 7.98 (br, 1H), 7.96-7.93 (m, 1H), 7.75-7.71 (m, 2H), 7.49-7.45 (m, 1H), 7.43-7.36 (m, 3H), 7.33-7.28 (m, 1H), 3.98 (s, 1H); ^{13}C NMR (125 MHz, d_6-DMSO) δ 177.4, 153.4, 143.4, 135.1, 128.7, 128.6, 126.7, 126.4, 125.7, 123.3, 122.8, 85.2, 78.2, 72.7. FT-IR: ν (cm^{-1}) 3564, 3417, 2120, 1633, 1489, 1157, 1067, 984. HRMS [ESI] calcd for $C_{16}H_{11}NNaOS$ $[M+Na]^+$ 288.0454, found 288.0453.

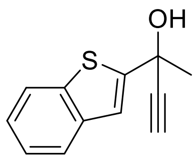

1f: yellow solid, m.p. 50-51 °C. ^1H NMR (500 MHz, $CDCl_3$) δ 7.83-7.79 (m, 1H), 7.75-7.72 (m, 1H), 7.44 (s, 1H), 7.38-7.30 (m, 2H), 2.82 (br, 1H), 2.75 (s, 1H), 1.96 (s, 3H); ^{13}C NMR (125 MHz, $CDCl_3$) δ 150.1, 139.7, 139.2, 124.6, 124.5, 123.9, 122.5, 120.7, 86.0, 73.2, 67.6, 32.8. FT-IR: ν (cm^{-1}) 3291, 2985, 2116, 1653, 1369, 1155, 1069, 748. HRMS [ESI] calcd for $C_{12}H_{11}OS$ $[M+H]^+$ 203.0525, found 203.0519.

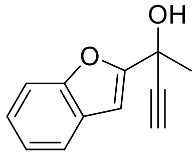

1g: yellow solid, m.p. 53-54 °C. ^1H NMR (500 MHz, $CDCl_3$) δ 7.58-7.55 (m, 1H), 7.52-7.48 (m, 1H), 7.33-7.28 (m, 1H), 7.27-7.22 (m, 1H), 6.81 (s, 1H), 2.79 (br, 1H), 2.68 (s, 1H), 1.95 (s, 3H); ^{13}C NMR (125 MHz, $CDCl_3$) δ 158.1, 155.1, 127.8, 124.7, 123.0, 121.4, 111.5, 102.7, 84.7, 72.9, 65.2, 28.6. FT-IR: ν (cm^{-1}) 3292, 2993, 2120, 1651, 1454, 1373, 1141, 752. HRMS [ESI] calcd for $C_{12}H_{10}KO_2$ $[M+K]^+$ 225.0312, found 225.0314.

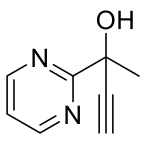

1h: yellow solid, m.p. 65-66 °C. ^{1}H NMR (500 MHz, $CDCl_3$) δ 8.80 (d, *J* = 5.0 Hz, 2H), 7.30 (dd, *J* = 5.0 Hz, 5.0 Hz, 1H), 5.13 (br, 1H), 2.54 (s, 1H), 1.90 (s, 3H); ^{13}C NMR (125 MHz, $CDCl_3$) δ 169.8, 157.4, 120.1, 86.4, 71.8, 69.6, 30.0. FT-IR: ν (cm^{-1}) 3291, 2986, 2112, 1634, 1508, 1404, 1153, 1086. HRMS [ESI] calcd for $C_8H_9N_2O$ $[M+H]^+$ 149.0709, found 149.0709.

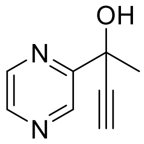

1i: yellow oil. ^{1}H NMR (400 MHz, $CDCl_3$) δ 8.80 (s, 1H), 8.57-8.50 (m, 2H), 4.68 (br, 1H), 2.64 (s, 1H), 1.85 (s, 3H); ^{13}C NMR (100 MHz, $CDCl_3$) δ 157.3, 143.9, 142.5, 142.3, 85.7, 73.3, 68.0, 31.5. FT-IR: ν (cm^{-1}) 3562, 3332, 2114, 1634, 1506, 1339, 1147, 1107. HRMS [ESI] calcd for $C_8H_9N_2O$ $[M+H]^+$ 149.0709, found 149.0705.

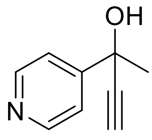

1j: yellow solid, m.p. 149-150 °C. ^{1}H NMR (400 MHz, $CDCl_3$) δ 8.57-8.53 (m, 2H), 7.57-7.54 (m, 2H), 3.70 (br, 1H), 2.68 (s, 1H), 1.76 (s, 3H); ^{13}C NMR (100 MHz, d_6-DMSO) δ 155.3, 150.0, 120.4, 87.9, 75.3, 67.6, 33.3. FT-IR: ν (cm^{-1}) 3235, 1867, 1634, 1506, 1456, 1361, 1161, 1007. HRMS [ESI] calcd for $C_9H_{10}NO$ $[M+H]^+$ 148.0757, found 148.0752.

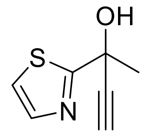

1k: yellow solid, m.p. 92-93 °C. ^{1}H NMR (500 MHz, $CDCl_3$) δ 7.73 (s, 1H), 7.30 (s, 1H), 4.47 (br, 1H), 2.67 (s, 1H), 1.95 (s, 3H); ^{13}C NMR (125 MHz, $CDCl_3$) δ 174.6, 142.5, 119.9, 85.3, 73.4, 68.4, 31.7. FT-IR: ν (cm^{-1}) 3219, 2797, 2112, 1634, 1506, 1368, 1144, 1061. HRMS [ESI] calcd for C_7H_8NOS $[M+H]^+$ 154.0321, found 154.0320.

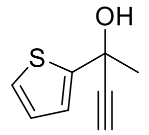

1l: yellow oil. ^{1}H NMR (500 MHz, $CDCl_3$) δ 7.27-7.24 (m, 1H), 7.21-7.19 (m, 1H), 6.95 (dd, *J* = 5.0 Hz, 4.0 Hz, 1H), 2.71 (br, 1H), 2.68 (s, 1H), 1.90 (s, 3H); ^{13}C NMR (125 MHz, $CDCl_3$) δ 149.7, 126.7, 125.3, 124.2, 86.4, 72.6, 67.2, 33.0. FT-IR: ν (cm^{-1}) 3291, 2988, 2114, 1637, 1369, 1236, 1136, 926. HRMS [ESI] calcd for C_8H_7S $[M+H-H_2O]^+$ 135.0263, found 135.0260.

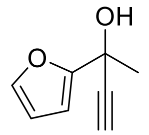

1m: yellow oil. ^{1}H NMR (500 MHz, $CDCl_3$) δ 7.41-7.39 (m, 1H), 6.42-6.39 (m, 1H), 6.35-6.32 (m, 1H), 2.65 (br, 1H), 2.61 (s, 1H), 1.85 (s, 3H); ^{13}C NMR (125 MHz, $CDCl_3$) δ 155.7, 142.6, 110.3, 106.0, 85.1, 72.2, 64.7, 28.5. FT-IR: ν (cm^{-1}) 3294, 2993, 2118, 1633, 1373, 1339, 1159, 1010. HRMS [ESI] calcd for C_8H_7O $[M+H-H_2O]^+$ 119.0491, found 119.0486.

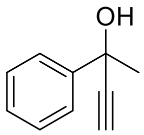

1n white solid, m.p. 47-48 °C. ^{1}H NMR (500 MHz, $CDCl_3$) δ 7.69-7.65 (m, 2H), 7.41-7.35 (m, 2H), 7.34-7.29 (m, 1H), 2.68 (s, 1H), 2.44 (br, 1H), 1.80 (s, 3H); ^{13}C NMR (125 MHz, $CDCl_3$) δ 145.0, 128.4, 127.9, 124.9, 87.2, 73.1, 69.9, 33.1. FT-IR: ν (cm^{-1}) 3294, 2987, 2114, 1635, 1447, 1225, 1091, 764. HRMS [ESI] calcd for $C_{10}H_9$ $[M+H-H_2O]^+$ 129.0699, found 129.0695.

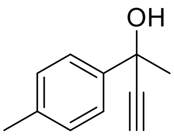

1o: white solid, m.p. 30-31 °C. ^{1}H NMR (500 MHz, $CDCl_3$) δ 7.58-7.53 (m, 2H), 7.20-7.17 (m, 2H), 2.67 (s, 1H), 2.43 (br, 1H), 2.36 (s, 3H), 1.79 (s, 3H); ^{13}C NMR (125 MHz, $CDCl_3$) δ 142.2, 137.6, 129.0, 124.8, 87.4, 72.9, 69.7, 33.0, 21.1. FT-IR: ν (cm^{-1}) 3291, 2986, 2012, 1634, 1508, 1339, 1153, 1086. HRMS [ESI] calcd for $C_{11}H_{12}KO$ $[M+K]^+$ 199.0520, found 199.0520.

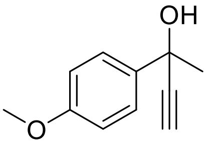

1p: white solid, m.p. 39-40 °C. ^{1}H NMR (500 MHz, $CDCl_3$) δ 7.60-7.56 (m, 2H), 6.90-6.87 (m, 2H), 3.81 (s, 3H), 2.67 (s, 1H), 2.53-2.50 (m, 1H), 1.77 (s, 3H); ^{13}C NMR (125 MHz, $CDCl_3$) δ 159.2, 137.2, 126.2, 113.6, 87.4, 73.0, 69.5, 55.4, 33.0. FT-IR: ν (cm^{-1}) 3294, 2986, 2112, 1611, 1508, 1252, 1179, 1030. HRMS [ESI] calcd for $C_{11}H_{12}NaO$ $[M+Na]^+$ 199.0730, found 199.0730.

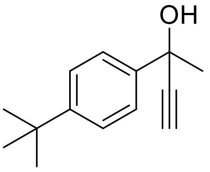

1q: white solid, m.p. 73-74 °C. ^{1}H NMR (500 MHz, $CDCl_3$) δ 7.52-7.49 (m, 2H), 7.33-7.29 (m, 2H), 2.58 (s, 1H), 2.37 (br, 1H), 1.70 (s, 3H), 1.24 (s, 9H); ^{13}C NMR (125 MHz, $CDCl_3$) δ 150.9, 142.0, 125.3, 124.6, 87.4, 72.9, 69.7, 34.5, 32.9, 31.4. FT-IR: ν (cm^{-1}) 3305, 2964, 2114, 1634, 1506, 1361, 1157, 1086. HRMS [ESI] calcd for $C_{14}H_{18}NaO$ $[M+Na]^+$ 225.1250, found 225.1241.

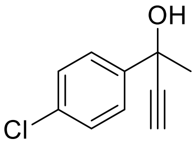

1r: yellow solid, m.p. 39-40 °C. ^{1}H NMR (500 MHz, $CDCl_3$) δ 7.61-7.56 (m, 2H), 7.35-7.31 (m, 2H), 2.69 (s, 1H), 2.56 (br, 1H), 1.76 (s, 3H); ^{13}C NMR (125 MHz, $CDCl_3$) δ 143.6, 133.7, 128.5, 126.5, 86.8, 73.5, 69.4, 33.2. FT-IR: ν (cm^{-1}) 3298, 2988, 2114, 1636, 1489, 1396, 1153, 1094. HRMS [EI] calcd for $C_{10}H_9ClO$ $[M]^+$ 180.0337, found 180.0339.

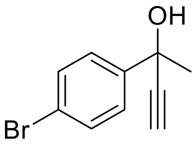

1s: white solid, m.p. 42-43 °C. ^{1}H NMR (500 MHz, $CDCl_3$) δ 7.54-7.50 (m, 2H), 7.50-7.46 (m, 2H), 2.68 (s, 1H), 2.59 (br, 1H), 1.75 (s, 3H); ^{13}C NMR (125 MHz, $CDCl_3$) δ 144.1, 131.4, 126.8, 121.9, 86.7, 73.5, 69.5, 33.2. FT-IR: ν (cm^{-1}) 3296, 2986, 2114, 1636, 1487, 1394, 1152, 1086. HRMS [ESI] calcd for $C_{10}H_{10}BrO$ $[M+H]^+$ 224.9910, found 224.9902.

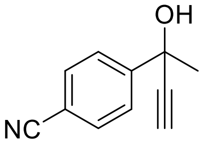

1t: yellow oil. ^{1}H NMR (500 MHz, $CDCl_3$) δ 7.78-7.74 (m, 2H), 7.66-7.63 (m, 2H), 2.72 (s, 1H), 2.71 (br, 1H), 1.76 (s, 3H); ^{13}C NMR (125 MHz, $CDCl_3$) δ 150.2, 132.3, 125.8, 118.7, 111.6, 86.1, 74.0, 69.4, 33.3. FT-IR: ν (cm^{-1}) 3294, 2988, 2232, 2114, 1609, 1404, 1368, 1159. HRMS [ESI] calcd for $C_{11}H_9NNaO$ $[M+Na]^+$ 194.0576, found 194.0570.

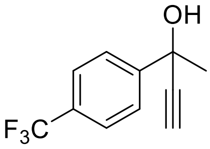

1u: yellow solid, m.p. 48-49 °C. ^{1}H NMR (400 MHz, $CDCl_3$) δ 7.80-7.75 (m, 2H), 7.65-7.60 (m, 2H), 2.71 (s, 1H), 2.64 (s, 1H), 1.78 (s, 3H); ^{13}C NMR (125 MHz, $CDCl_3$) δ 148.9, 130.1 (q, J = 32.1 Hz), 125.4, 125.4 (q, J = 3.8 Hz), 124.1 (q, J = 270.4 Hz), 86.5, 73.7, 69.6, 33.3; ^{19}F NMR (376 MHz, $CDCl_3$) δ -62.5. FT-IR: ν (cm^{-1}) 3306,

2989, 2118, 1620, 1414, 1371, 1165, 1070. HRMS [ESI] calcd for $C_{11}H_{10}F_3O$ $[M+H]^+$ 215.0678, found 215.0683.

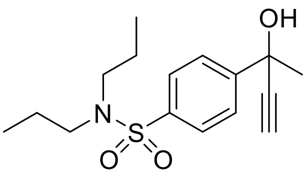

1v: yellow solid, m.p. 84-85 °C. ^{1}H NMR (400 MHz, $CDCl_3$) δ 7.78-7.75 (m, 4H), 3.05 (t, *J* = 7.6 Hz, 4H), 2.76 (s, 1H), 2.71 (s, 1H), 1.77 (s, 3H), 1.60-1.50 (m, 4H), 0.86 (t, *J* = 7.6 Hz, 6H); ^{13}C NMR (100 MHz, $CDCl_3$) δ 149.4, 139.4, 127.2, 125.6, 86.4, 73.8, 69.5, 50.2, 33.4, 22.1, 11.2. FT-IR: ν (cm^{-1}) 3308, 2967, 2345, 1636, 1489, 1361, 1155, 1094. HRMS [ESI] calcd for $C_{16}H_{24}NO_3S$ $[M+H]^+$ 310.1471, found 310.1466.

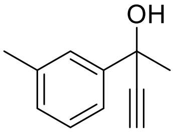

1w: colorless oil. ^{1}H NMR (400 MHz, $CDCl_3$) δ 7.50-7.44 (m, 2H), 7.29-7.24 (m, 1H), 7.15-7.10 (m, 1H), 2.67 (s, 1H), 2.42 (br, 1H), 2.39 (s, 3H), 1,79 (s, 3H); ^{13}C NMR (100 MHz, $CDCl_3$) δ 145.0, 138.1, 128.6, 128.3, 125.5, 121.9, 87.4, 73.0, 69.8, 33.1, 21.6. FT-IR: ν (cm^{-1}) 3294, 2986, 2113, 1607, 1487, 1368, 1153, 1086. HRMS [ESI] calcd for $C_{11}H_{12}KO$ $[M+K]^+$ 199.0520, found 199.0515.

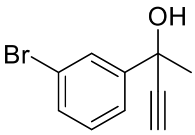

1x: colorless oil. ^{1}H NMR (400 MHz, $CDCl_3$) δ 7.83-7.80 (m, 1H), 7.60-7.56 (m, 1H), 7.46-7.41 (m, 1H), 7.27-7.21 (m, 1H), 2.70 (s, 1H), 2.54 (s, 1H), 1.77 (s, 3H); ^{13}C NMR (100 MHz, $CDCl_3$) δ 147.3, 130.9, 130.0, 128.2, 123.6, 122.5, 86.6, 73.6, 69.4, 33.2. FT-IR: ν (cm^{-1}) 3296, 2986, 2115, 1636, 1472, 1338, 1155, 1074. HRMS [ESI] calcd for $C_{10}H_{10}BrO$ $[M+H]^+$ 224.9910, found 224.9907.

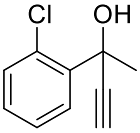

1y: yellow oil. ^{1}H NMR (400 MHz, $CDCl_3$) δ 7.80-7.76 (m, 1H), 7.42-7.37 (m, 1H), 7.31-7.21 (m, 2H), 3.31 (br, 1H), 2.64 (s, 1H), 1.95 (s, 3H); ^{13}C NMR (100 MHz, $CDCl_3$) δ 140.7, 131.8, 131.4, 129.2, 127.0, 126.8, 86.2, 72.9, 68.9, 29.6. FT-IR: ν (cm^{-1}) 3296, 2986, 2114, 1636, 1431, 1371, 1155, 1097. HRMS [EI] calcd for $C_{10}H_9ClO$ $[M]^+$ 180.0337, found 180.0338.

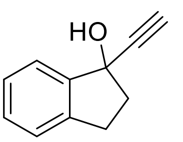

4a: white solid, m.p. 63-64 °C. ^{1}H NMR (400 MHz, $CDCl_3$) δ 7.54-7.49 (m, 1H), 7.31-7.23 (m, 3H), 3.16-3.06 (m, 1H), 2.96-2.86 (m, 1H), 2.63 (s, 1H), 2.61-2.51 (m, 1H), 2.47-2.39 (m, 1H), 2.37 (s, 1H); ^{13}C NMR (100 MHz, $CDCl_3$) δ 145.3, 143.1, 129.2, 127.2, 125.1, 123.2, 85.9, 76.2, 73.0, 43.1, 29.6. FT-IR: ν (cm^{-1}) 3291, 3022, 2988, 1634, 1474, 1338, 1157, 1042. HRMS [ESI] calcd for $C_{11}H_{10}KO$ $[M+K]^+$ 197.0363, found 197.0370.

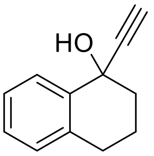

4b: yellow solid, m.p. 46-47 °C. ^{1}H NMR (400 MHz, $CDCl_3$) δ 7.80-7.75 (m, 1H), 7.28-7.20 (m, 2H), 7.13-7.08 (m, 1H), 2.89-2.75 (m, 2H), 2.60 (s, 1H), 2.36 (s, 1H), 2.25-2.20 (m, 2H), 2.09-1.99 (m, 1H), 1.99-1.88 (m, 1H); ^{13}C NMR (100 MHz, $CDCl_3$) δ 138.6, 136.1, 129.2, 128.3, 127.7, 126.6, 88.1, 72.4, 67.7, 38.9, 29.2, 19.1. FT-IR: ν (cm^{-1}) 3289, 2940, 2106,

1636, 1489, 1362, 1161, 1086. HRMS [ESI] calcd for $C_{12}H_{12}NaO$ $[M+Na]^+$ 195.0780, found 195.0783.

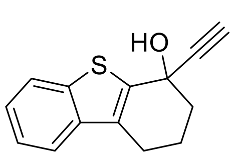

4c: white solid, m.p. 77-78 °C. ^1H NMR (400 MHz, $CDCl_3$) δ 7.85-7.79 (m, 1H), 7.65-7.59 (m, 1H), 7.40-7.31 (m, 2H), 2.86-2.73 (m, 2H), 2.68 (s, 1H), 2.63 (s, 1H), 2.40-2.26 (m, 2H), 2.20-2.06 (m, 2H); ^{13}C NMR (125 MHz, $CDCl_3$) δ 139.3, 139.3, 138.7, 132.1, 125.1, 124.2, 122.8, 121.9, 86.4, 73.2, 66.2, 39.7, 23.5, 19.3. FT-IR: ν (cm^{-1}) 3291, 2932, 2114, 1634, 1471, 1373, 1153, 1047. HRMS [ESI] calcd for $C_{14}H_{11}S$ $[M+H-H_2O]^+$ 211.0576, found 211.0571.

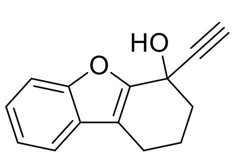

4d: yellow oil. ^1H NMR (500 MHz, $CDCl_3$) δ 7.52-7.46 (m, 2H), 7.33-7.28 (m, 1H), 7.26-7.21 (m, 1H), 2.77 (s, 1H), 2.76-2.69 (m, 1H), 2.65 (s, 1H), 2.69-2.62 (m, 1H), 2.37-2.26 (m, 2H), 2.10-1.97 (m, 2H); ^{13}C NMR (125 MHz, $CDCl_3$) δ 154.7, 151.2, 127.7, 124.9, 122.7, 119.8, 115.8, 111.7, 84.4, 73.2, 64.0, 39.5, 20.6, 19.9. FT-IR: ν (cm^{-1}) 3291, 2947, 2114, 1634, 1452, 1250, 1134, 970. HRMS [ESI] calcd for $C_{14}H_{12}NaO_2$ $[M+Na]^+$ 235.0730, found 235.0734.

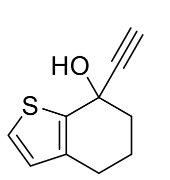

4e: yellow oil. ^1H NMR (500 MHz, $CDCl_3$) δ 7.24 (d, J = 4.0 Hz, 1H), 6.74 (d, J = 4.0 Hz, 1H), 2.74-2.67 (m, 1H), 2.63 (s, 1H), 2.65-2.58 (m, 1H), 2.47 (s, 1H), 2.24-2.20 (m, 2H), 2.08-1.99 (m, 1H), 1.99-1.91 (m, 1H); ^{13}C NMR (125 MHz, $CDCl_3$) δ 138.6, 137.7, 127.2, 125.2, 87.1, 72.4, 65.5, 39.6, 25.4, 19.6. FT-IR: ν (cm^{-1}) 3289, 2936, 2112, 1651, 1454, 1319, 1159, 1089. HRMS [ESI] calcd for $C_{10}H_9S$ $[M+H-H_2O]^+$ 161.0419, found 161.0415.

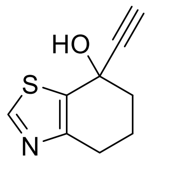

4f: yellow solid, m.p. 101-102 °C. ^1H NMR (400 MHz, $CDCl_3$) δ 8.67 (s, 1H), 4.06 (s, 1H), 2.91-2.75 (m, 2H), 2.63 (s, 1H), 2.28-2.15 (m, 2H), 2.12-1.96 (m, 2H); ^{13}C NMR (100 MHz, $CDCl_3$) δ 152.7, 152.6, 133.6, 86.7, 72.9, 65.1, 39.6, 26.4, 19.5. FT-IR: ν (cm^{-1}) 3287, 2949, 2110, 1651, 1454, 1359, 1265, 1085. HRMS [ESI] calcd for $C_9H_{10}NOS$ $[M+H]^+$ 180.0478, found 180.0473.

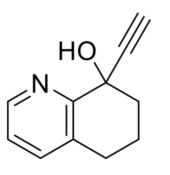

4g: yellow solid, m.p. 91-92 °C. ^1H NMR (500 MHz, $CDCl_3$) δ 8.45 (d, J = 4.5 Hz, 1H), 7.44 (d, J = 7.5 Hz, 1H), 7.16 (dd, J = 7.5 Hz, 4.5 Hz, 1H), 4.71 (s, 1H), 2.86-2.81 (m, 2H), 2.55 (s, 1H), 2.47-2.42 (m, 1H), 2.15-2.07 (m, 1H), 2.06-1.97 (m, 2H); ^{13}C NMR (125 MHz, $CDCl_3$) δ 156.8, 147.3, 137.6, 130.6, 123.2, 87. 6, 73.0, 68.0, 36.5, 27.9, 19.2. FT-IR: ν (cm^{-1}) 3286, 2949, 2104, 1573, 1445, 1329, 1169, 1086. HRMS [ESI] calcd for $C_{11}H_{12}NO$ $[M+H]^+$ 174.0914, found 174.0913.

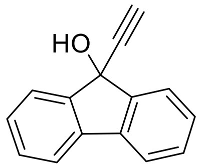

4h: white solid, m.p. 105-106 °C. ^{1}H NMR (400 MHz, $CDCl_3$) δ 7.73-7.68 (m, 2H), 7.64-7.59 (m, 2H), 7.44-7.32 (m, 4H), 2.70 (s, 1H), 2.47 (s, 1H); ^{13}C NMR (100 MHz, $CDCl_3$) δ 146.6, 139.1, 129.9, 128.7, 124.3, 120.3, 83.9, 74.6, 71.5. FT-IR: ν (cm^{-1}) 3290, 2989, 2112, 1633, 1506, 1365, 1158, 1059. HRMS [ESI] calcd for $C_{15}H_{11}O$ $[M+H]^+$ 207.0804, found 207.0810.

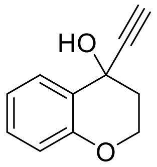

4i: white solid, m.p. 40-41 °C. ^{1}H NMR (400 MHz, $CDCl_3$) δ 7.68-7.64 (m, 1H), 7.25-7.20 (m, 1H), 6.98-6.93 (m, 1H), 6.86-6.82 (m, 1H), 4.34-4.29 (m, 2H), 2.71 (s, 1H), 2.64 (s, 1H), 2.41-2.33 (m, 1H), 2.32-2.26 (m, 1H); ^{13}C NMR (100 MHz, $CDCl_3$) δ 153.5, 130.3, 128.2, 124.6, 120.9, 117.3, 86.3, 73.1, 63.6, 62.2, 37.0. FT-IR: ν (cm^{-1}) 3289, 2926, 2117, 1633, 1456, 1373, 1159, 758. HRMS [ESI] calcd for $C_{11}H_{10}NaO_2$ $[M+Na]^+$ 197.0573, found 197.0582.

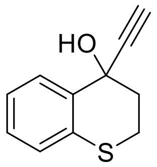

4j: yellow solid, m.p. 54-55 °C. ^{1}H NMR (500 MHz, $CDCl_3$) δ 7.86-7.82 (m, 1H), 7.19-7.15 (m, 1H), 7.13-7.08 (m, 2H), 3.33-3.26 (m, 1H), 3.05-2.99 (m, 1H), 2.68 (s, 1H), 2.59-2.53 (m, 1H), 2.46 (s, 1H), 2.41-1.34 (m, 1H); ^{13}C NMR (125 MHz, $CDCl_3$) δ 134.7, 132.5, 128.8, 128.6, 126.8, 124.6, 86.5, 73.9, 66.4, 37.0, 22.2. FT-IR: ν (cm^{-1}) 3287, 2924, 2112, 1636, 1471, 1173, 1080, 1036. HRMS [ESI] calcd for $C_{11}H_{10}KOS$ $[M+K]^+$ 229.0084, found 229.0086.

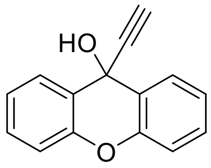

4k: yellow solid, m.p. 63-64 °C. ^{1}H NMR (400 MHz, $CDCl_3$) δ 7.99-7.95 (m, 2H), 7.44-7.38 (m, 2H), 7.27-7.18 (m, 4H), 2.92 (s, 1H), 2.75 (s, 1H); ^{13}C NMR (100 MHz, $CDCl_3$) δ 149.6, 130.2, 128.6, 123.7, 123.3, 116.9, 85.1, 75.8, 64.0. FT-IR: ν (cm^{-1}) 3291, 2118, 1603, 1450, 1317, 1242, 1153, 756. HRMS [ESI] calcd for $C_{15}H_{10}NaO_2$ $[M+Na]^+$ 245.0573, found 245.0579.

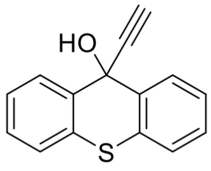

4l: yellow solid, m.p. 96-97 °C. ^{1}H NMR (400 MHz, $CDCl_3$) δ 8.17-8.11 (m, 2H), 7.54-7.48 (m, 2H), 7.40-7.29 (m, 4H), 2.96 (s, 1H), 2.93 (s, 1H); ^{13}C NMR (100 MHz, $CDCl_3$) δ 136.0, 131.5, 128.2, 127.0, 126.7, 126.6, 82.7, 76.7, 70.5. FT-IR: ν (cm^{-1}) 3289, 2926, 2114, 1634, 1456, 1267, 1159, 1063. HRMS [ESI] calcd for $C_{15}H_{10}NaOS$ $[M+Na]^+$ 261.0345, found 261.0346.

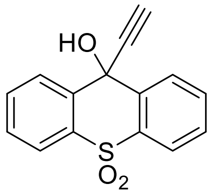

4m: yellow solid, m.p. 211-212 °C. ^{1}H NMR (400 MHz, d_6-DMSO) δ 8.17-8.13 (m, 2H), 8.10-8.06 (m, 2H), 7.86-7.80 (m, 2H), 7.75-7.68 (m, 2H), 3.76 (s, 1H), 3.37 (s, 1H); ^{13}C NMR (100 MHz, d_6-DMSO) δ 142.3, 135.4, 134.0, 130.0, 127.4, 123.7, 85.1, 77.6, 64.2. FT-IR: ν (cm^{-1}) 3258, 2920, 2114, 1445, 1292, 1163, 1136, 1063. HRMS [ESI] calcd for $C_{15}H_{14}NO_3S$ $[M+NH_4]^+$ 288.0689, found 288.0687.

b. Products

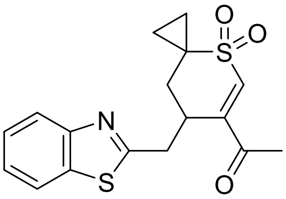

3a: yellow solid, m.p. 144-145 °C. ^{1}H NMR (500 MHz, $CDCl_3$) δ 7.95 (d, *J* = 8.5 Hz, 1H), 7.84 (d, *J* = 8.0 Hz, 1H), 7.48-7.43 (m, 1H), 7.39-7.34 (m, 1H), 7.15 (s, 1H), 3.59-3.53 (m, 1H), 3.37-3.31 (m, 1H), 3.29-3.24 (m, 1H), 2.52 (dd, *J* = 15.5 Hz, 5.0 Hz, 1H), 2.46 (s, 3H), 2.08 (dd, *J* = 15.5 Hz, 5.0 Hz, 1H), 1.72-1.65 (m, 1H), 1.48-1.40 (m, 2H), 0.86-0.79 (m, 1H); ^{13}C NMR (125 MHz, $CDCl_3$) δ 197.3, 167.5, 153.0, 147.6, 135.4, 135.3, 126.2, 125.3, 122. 9, 121.7, 35.9, 35.3, 34.2, 31.0, 26.5, 13.3, 8.4. FT-IR: ν (cm^{-1}) 2924, 1688, 1435, 1362, 1296, 1211, 1125, 763. HRMS [ESI] calcd for $C_{17}H_{18}NO_3S_2$ $[M+H]^+$ 348.0723, found 348.0732.

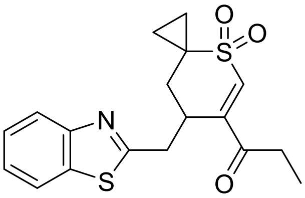

3b: yellow solid, m.p. 106-107 °C. ^{1}H NMR (500 MHz, $CDCl_3$) δ 7.95 (d, *J* = 8.0 Hz, 1H), 7.85 (d, *J* = 8.0 Hz, 1H), 7.48-7.44 (m, 1H), 7.40-7.35 (m, 1H), 7.11 (s, 1H), 3.61-3.55 (m, 1H), 3.37-3.30 (m, 1H), 3.28-3.23 (m, 1H), 2.90-2.81 (m, 1H), 2.78-2.69 (m, 1H), 2.52 (dd, *J* = 15.5 Hz, 5.0 Hz, 1H), 2.09 (dd, *J* = 15.5 Hz, 5.0 Hz, 1H), 1.72-1.65 (m, 1H), 1.48-1.41 (m, 2H), 1.14 (t, *J* = 7.5 Hz, 3H), 0.84-0.80 (m, 1H); ^{13}C NMR (125 MHz, $CDCl_3$) δ 200.1, 167. 6, 153.0, 147.5, 135.3, 134.2, 126.2, 125.3, 122.9, 121.7, 36.0, 35.3, 34.5, 31.8, 31.1, 13.2, 8.4, 7.9. FT-IR: ν (cm^{-1}) 2922, 1668, 1514, 1435, 1298, 1169, 1125, 762. HRMS [ESI] calcd for $C_{18}H_{20}NO_3S_2$ $[M+H]^+$ 362.0879, found 362.0877.

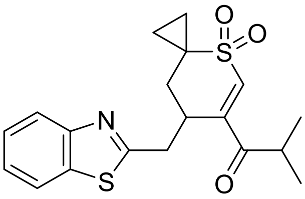

3c: yellow solid, m.p. 125-126 °C. ^{1}H NMR (500 MHz, $CDCl_3$) δ 7.96 (d, *J* = 8.0 Hz, 1H), 7.85 (d, *J* = 8.0 Hz, 1H), 7.49-7.44 (m, 1H), 7.40-7.35 (m, 1H), 7.11 (s, 1H), 3.64-3.57 (m, 1H), 3.31-3.18 (m, 3H), 2.50 (dd, *J* = 15.5 Hz, 5.0 Hz, 1H), 2.10 (dd, *J* = 15.5 Hz, 5.0 Hz, 1H), 1.73-1.65 (m, 1H), 1.47-1.40 (m, 2H), 1.19 (d, *J* = 6.0 Hz, 3H), 1.18 (d, *J* = 6.5 Hz, 3H), 0.85-0.80 (m, 1H); ^{13}C NMR (125 MHz, $CDCl_3$) δ 203.9, 167.5, 153.0, 147.1, 135.3, 133.8, 126.2, 125.3, 122.9, 121.7, 36.0, 35.7, 35.3, 34.6, 30.9, 19.4, 18.5, 13.0, 8.5. FT-IR: ν (cm^{-1}) 2928, 2359, 1688, 1514, 1435, 1298, 1196, 1123. HRMS [ESI] calcd for $C_{19}H_{22}NO_3S_2$ $[M+H]^+$ 376.1036, found 376.1044.

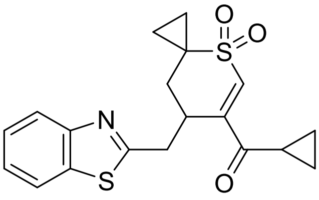

3d: yellow solid, m.p. 153-154 °C. ^{1}H NMR (500 MHz, $CDCl_3$) δ 7.95 (d, *J* = 8.5 Hz, 1H), 7.84 (d, *J* = 8.0 Hz, 1H), 7.48-7.43 (m, 1H), 7.39-7.34 (m, 1H), 7.24 (s, 1H), 3.63-3.57 (m, 1H), 3.34-3.25 (m, 2H), 2.44 (dd, *J* = 15.5 Hz, 5.0 Hz, 1H), 2.37-2.31 (m, 1H), 2.19 (dd, *J* = 15.5 Hz, 5.5 Hz, 1H), 1.70-1.64 (m, 1H), 1.47-1.41 (m, 1H), 1.38-1.32 (m, 1H), 1.20-1.13 (m, 2H), 1.12-1.08 (m, 1H), 1.07-1.00 (m, 1H), 0.87-0.81 (m, 1H); ^{13}C NMR (125 MHz, $CDCl_3$) δ 200.0, 167.5, 153.0, 149.0, 135.3, 133.8, 126.2, 125.3, 122.9, 121.7, 36.1, 35.5, 34.8, 31.4, 18.2, 13.6, 12.7, 12.4, 8.9. FT-IR: ν (cm^{-1}) 2922, 2359,

1688, 1506, 1456, 1298, 1163, 1125. HRMS [ESI] calcd for $C_{19}H_{20}NO_3S_2$ $[M+H]^+$ 374.0879, found 374.0877.

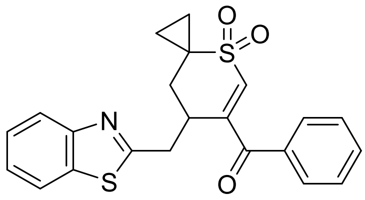

3e: white solid, m.p. 173-174 °C. ^{1}H NMR (500 MHz, $CDCl_3$) δ 7.91-7.86 (m, 3H), 7.81 (d, *J* = 8.0 Hz, 1H), 7.66-7.61 (m, 1H), 7.52-7.47 (m, 2H), 7.46-7.41 (m, 1H), 7.38-7.33 (m, 1H), 6.76 (s, 1H), 3.83-3.76 (m, 1H), 3.44 (dd, *J* = 15.0 Hz, 9.0 Hz, 1H), 3.33 (dd, *J* = 15.0 Hz, 4.0 Hz, 1H), 2.65 (dd, *J* = 15.0 Hz, 8.0 Hz, 1H), 2.25 (dd, *J* = 15.0 Hz, 5.0 Hz, 1H), 1.70-1.64 (m, 1H), 1.50-1.43 (m, 1H), 1.23-1.17 (m, 1H), 0.97-0.91 (m, 1H); ^{13}C NMR (100 MHz, $CDCl_3$) δ 194.2, 166.7, 153.1, 148.3, 135.2, 135.0, 134.4, 134.2, 130.2, 128.9, 126.2, 125.3, 122.9, 121.6, 36.3, 36.0, 35.9, 32.5, 11.1, 10.2. FT-IR: ν (cm^{-1}) 2922, 1668, 1446, 1300, 1273, 1236, 1128, 762. HRMS [ESI] calcd for $C_{22}H_{20}NO_3S_2$ $[M+H]^+$ 410.0879, found 410.0878.

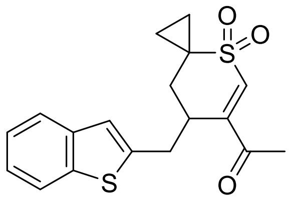

3f: yellow solid, m.p. 151-152 °C. ^{1}H NMR (500 MHz, $CDCl_3$) δ 7.77 (d, *J* = 8.0 Hz, 1H), 7.69 (d, *J* = 7.5 Hz, 1H), 7.36-7.27 (m, 2H), 7.12 (s, 1H), 7.05 (s, 1H), 3.42-3.36 (m, 1H), 3.17 (dd, *J* = 15.0 Hz, 3.0 Hz, 1H), 3.00 (dd, *J* = 15.0 Hz, 11.0 Hz, 1H), 2.43 (s, 3H), 2.39 (dd, *J* = 15.5 Hz, 5.0 Hz, 1H), 2.12 (dd, *J* = 15.5 Hz, 5.5 Hz, 1H), 1.73-1.66 (m, 1H), 1.45-1.39 (m, 1H), 1.17-1.11 (m, 1H), 0.81-0.76 (m, 1H); ^{13}C NMR (125 MHz, $CDCl_3$) δ 197.4, 148.4, 141.6, 139.8, 139.5, 134.8, 124.5, 124.2, 123.1, 122.8, 122.3, 35.5, 35.4, 33.3, 31.1, 26.6, 12.9, 8.7. FT-IR: ν (cm^{-1}) 2920, 2359, 1688, 1435, 1296, 1221, 1125, 750. HRMS [ESI] calcd for $C_{18}H_{19}O_3S_2$ $[M+H]^+$ 347.0770, found 347.0761.

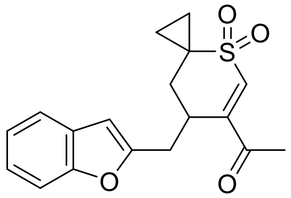

3g: white solid, m.p. 109-110 °C. ^{1}H NMR (500 MHz, $CDCl_3$) δ 7.49 (d, *J* = 7.5 Hz, 1H), 7.41 (d, *J* = 8.0 Hz, 1H), 7.25-7.18 (m, 2H), 7.08 (s, 1H), 6.47 (s, 1H), 3.52-3.46 (m, 1H), 3.06-2.95 (m, 2H), 2.42 (s, 3H), 2.37 (dd, *J* = 15.5 Hz, 5.0 Hz, 1H), 2.16 (dd, *J* = 15.5 Hz, 5.5 Hz, 1H), 1.71-1.65 (m, 1H), 1.46-1.40 (m, 1H), 1.22-1.16 (m, 1H), 0.86-0.80 (m, 1H); ^{13}C NMR (125 MHz, $CDCl_3$) δ 197.4, 154.9, 154.8, 148.5, 134.7, 128.3, 124.0, 122.9, 120.6, 111.0, 104.7, 35.5, 33.4, 31.7, 31.1, 26.5, 12.6, 8.9. FT-IR: ν (cm^{-1}) 2924, 2357, 1694, 1454, 1362, 1298, 1211, 1125. HRMS [ESI] calcd for $C_{18}H_{19}O_4S$ $[M+H]^+$ 331.0999, found 331.0992.

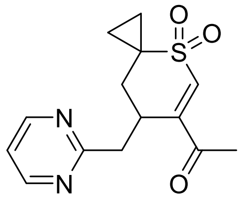

3h: yellow solid, m.p. 142-143 °C. ^{1}H NMR (500 MHz, $CDCl_3$) δ 8.64 (d, *J* = 4.0 Hz, 2H), 7.17-7.13 (m, 1H), 7.02 (s, 1H), 3.68-3.61 (m, 1H), 3.22 (dd, *J* = 14.0 Hz, 10.0 Hz, 1H), 3.11-3.05 (m, 1H), 2.43 (s, 3H), 2.38 (dd, *J* = 15.0 Hz, 4.5 Hz, 1H), 1.95 (dd, *J* = 15.0 Hz, 5.0 Hz, 1H), 1.64-1.57 (m, 1H), 1.42-1.35 (m, 1H), 1.30-1.23 (m, 1H), 0.82-0.75 (m, 1H); ^{13}C NMR (125 MHz, $CDCl_3$) δ 197.6, 168.0, 157.1, 149.4, 133.9, 119.2, 41.1, 35.5, 33.4, 31.6, 26.6, 12.5, 8.8. FT-

IR: ν (cm^{-1}) 2922, 2359, 1694, 1564, 1423, 1294, 1213, 1125. HRMS [ESI] calcd for $C_{14}H_{17}N_2O_3S$ $[M+H]^+$ 293.0954, found 293.0944.

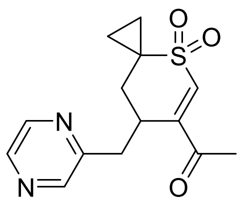

3i: yellow solid, m.p. 70-71 °C. ^{1}H NMR (500 MHz, $CDCl_3$) δ 8.49 (s, 1H), 8.48-8.46 (m, 1H), 8.45-8.43 (m, 1H), 7.09 (s, 1H), 3.50-3.44 (m, 1H), 3.07-2.96 (m, 2H), 2.45 (s, 3H), 2.34 (dd, *J* = 15.5 Hz, 5.0 Hz, 1H), 2.00 (dd, *J* = 15.5 Hz, 5.5 Hz, 1H), 1.69-1.63 (m, 1H), 1.45-1.39 (m, 1H), 1.37-1.31 (m, 1H), 0.85-0.79 (m, 1H); ^{13}C NMR (125 MHz, $CDCl_3$) δ 197.5, 154.3, 148.6, 145.1, 143.9, 143.1, 134.7, 36.8, 35.4, 33.9, 31.1, 26.5, 12.8, 8.7. FT-IR: ν (cm^{-1}) 2920, 1688, 1506, 1456, 1294, 1215, 1125, 1018. HRMS [ESI] calcd for $C_{14}H_{17}N_2O_3S$ $[M+H]^+$ 293.0954, found 293.0960.

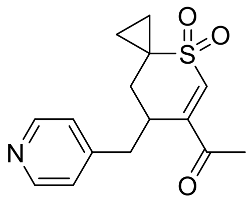

3j: yellow solid, m.p. 67-68 °C. ^{1}H NMR (500 MHz, $CDCl_3$) δ 8.54 (d, *J* = 4.5 Hz, 2H), 7.15 (d, *J* = 5.0 Hz, 2H), 7.13 (s, 1H), 3.37-3.30 (m, 1H), 2.99 (dd, *J* = 13.5 Hz, 3.0 Hz, 1H), 2.55 (dd, *J* = 13.5 Hz, 11.0 Hz, 1H), 2.44 (s, 3H), 2.24 (dd, *J* = 15.5 Hz, 5.0 Hz, 1H), 1.90 (dd, *J* = 15.5 Hz, 5.5 Hz, 1H), 1.72-1.66 (m, 1H), 1.44-1.38 (m, 1H), 1.04-0.98 (m, 1H), 0.79-0.72 (m, 1H); ^{13}C NMR (125 MHz, $CDCl_3$) δ 197.4, 150.2, 148.5, 147.5, 135.0, 124.1, 37.6, 35.3, 34.4, 31.2, 26.6, 12.7, 8.9. FT-IR: ν (cm^{-1}) 2922, 1688, 1603, 1506, 1456, 1294, 1213, 1125. HRMS [ESI] calcd for $C_{15}H_{18}NO_3S$ $[M+H]^+$ 292.1002, found 292.1010.

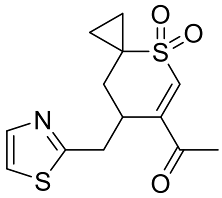

3k: red solid, m.p. 125-126 °C. ^{1}H NMR (500 MHz, $CDCl_3$) δ 7.65 (d, *J* = 3.0 Hz, 1H), 7.24 (d, *J* = 3.0 Hz, 1H), 7.11 (s, 1H), 3.45-3.39 (m, 1H), 3.27 (dd, *J* = 14.5 Hz, 10.0 Hz, 1H), 3.15 (dd, *J* = 15.0 Hz, 5.0 Hz, 1H), 2.45 (dd, *J* = 15.0 Hz, 4.5 Hz, 1H), 2.44 (s, 3H), 2.05 (dd, *J* = 15.0 Hz, 5.0 Hz, 1H), 1.68-1.62 (m, 1H), 1.46-1.38 (m, 2H), 0.85-0.80 (m, 1H); ^{13}C NMR (125 MHz, $CDCl_3$) δ 197.3, 166.6, 147.8, 142.6, 135.1, 119.3, 35.2, 34.8, 34.7, 30.8, 26.5, 13.2, 8.4. FT-IR: ν (cm^{-1}) 2922, 2357, 1682, 1504, 1294, 1213, 1125, 1041. HRMS [ESI] calcd for $C_{13}H_{16}NO_3S_2$ $[M+H]^+$ 298.0566, found 298.0558.

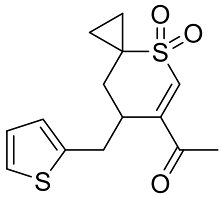

3l: white solid, m.p. 116-117 °C. ^{1}H NMR (500 MHz, $CDCl_3$) δ 7.17 (d, *J* = 5.0 Hz, 1H), 7.07 (s, 1H), 6.93 (dd, *J* = 5.0 Hz, 3.5 Hz, 1H), 6.81 (d, *J* = 3.5 Hz, 1H), 3.32-3.27 (m, 1H), 3.06 (dd, *J* = 14.5 Hz, 3.5 Hz, 1H), 2.96 (dd, *J* = 14.5 Hz, 11.0 Hz, 1H), 2.40 (s, 3H), 2.33 (dd, *J* = 15.5 Hz, 5.0 Hz, 1H), 2.10 (dd, *J* = 15.5 Hz, 5.5 Hz, 1H), 1.70-1.64 (m, 1H), 1.44-1.38 (m, 1H), 1.14-1.09 (m, 1H), 0.82-0.77 (m, 1H); ^{13}C NMR (125 MHz, $CDCl_3$) δ 197.4, 148.7, 140.4, 134.5, 127.1, 126.2, 124.4, 36.0, 35.4, 32.4, 31.1, 26.6, 12.8, 8.7. FT-IR: ν (cm^{-1}) 2922, 2359, 1694, 1435, 1360, 1296, 1211, 1125. HRMS [ESI] calcd for $C_{14}H_{17}O_3S_2$ $[M+H]^+$ 297.0614, found 297.0622.

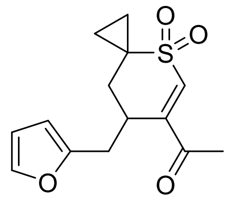

3m: brown oil. ^{1}H NMR (500 MHz, $CDCl_3$) δ 7.32-7.30 (m, 1H), 7.03 (s, 1H), 6.29-6.26 (m, 1H), 6.06-6.04 (m, 1H), 3.37-3.30 (m, 1H), 2.90-2.79 (m, 2H), 2.38 (s, 3H), 2.33 (dd, *J* = 15.0 Hz, 5.0 Hz, 1H), 2.09 (dd, *J* = 15.5 Hz, 5.5 Hz, 1H), 1.68-1.62 (m, 1H), 1.44-1.38 (m, 1H), 1.20-1.14 (m, 1H), 0.86-0.80 (m, 1H); ^{13}C NMR (125 MHz, $CDCl_3$) δ 197.4, 151.9, 148.9, 141.9, 134.3, 110.4, 107.6, 35.5, 33.8, 31.7, 30.6, 26.6, 12.6, 8.8. FT-IR: ν (cm^{-1}) 2920, 2359, 1688, 1506, 1361, 1296, 1213, 1125. HRMS [ESI] calcd for $C_{14}H_{17}O_4S$ $[M+H]^+$ 281.0842, found 218.0844.

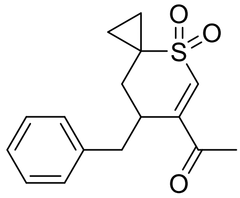

3n: yellow solid, m.p. 62-63 °C. ^{1}H NMR (500 MHz, $CDCl_3$) δ 7.33-7.28 (m, 2H), 7.25-7.21 (m, 1H), 7.21-7.18 (m, 2H), 7.06 (s, 1H), 3.37-3.30 (m, 1H), 2.98 (dd, *J* = 14.0 Hz, 4.0 Hz, 1H), 2.55 (dd, *J* = 14.0 Hz, 11.0 Hz, 1H), 2.40 (s, 3H), 2.25 (dd, *J* = 15.5 Hz, 5.0 Hz, 1H), 1.94 (dd, *J* = 15.5 Hz, 5.0 Hz, 1H), 1.72-1.65 (m, 1H), 1.43-1.36 (m, 1H), 1.10-1.04 (m, 1H), 0.77-0.72 (m, 1H); ^{13}C NMR (125 MHz, $CDCl_3$) δ 197.7, 149.7, 138.3, 133.9, 128.9, 128.8, 126.9, 38.3, 35.4, 35.4, 31.2, 26.7, 12.8, 8.7. FT-IR: ν (cm^{-1}) 2920, 1690, 1454, 1296, 1267, 1211, 1126, 743. HRMS [ESI] calcd for $C_{16}H_{19}O_3S$ $[M+H]^+$ 291.1049, found 291.1052.

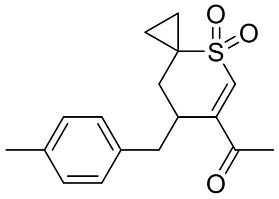

3o: yellow solid, m.p. 141-142 °C. ^{1}H NMR (500 MHz, $CDCl_3$) δ 7.13-7.06 (m, 4H), 7.05 (s, 1H), 3.34-3.27 (m, 1H), 2.97-2.90 (m, 1H), 2.51 (dd, *J* = 12.5 Hz, 12.5 Hz, 1H), 2.40 (s, 3H), 2.32 (s, 3H), 2.25 (dd, *J* = 15.5 Hz, 5.0 Hz, 1H), 1.94 (dd, *J* = 15.5 Hz, 5.0 Hz, 1H), 1.70-1.64 (m, 1H), 1.43-1.36 (m, 1H), 1.10-1.04 (m, 1H), 0.77-0.71 (m, 1H); ^{13}C NMR (125 MHz, $CDCl_3$) δ 197.7, 149.8, 136.5, 135.1, 133.7, 129.5, 128.7, 37.8, 35.5, 35.4, 31.1, 26.7, 21.1, 12.8, 8.7. FT-IR: ν (cm^{-1}) 2922, 1684, 1506, 1456, 1296, 1211, 1126, 922. HRMS [ESI] calcd for $C_{17}H_{20}NaO_3S$ $[M+Na]^+$ 327.1025, found 327.1030.

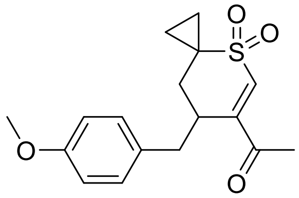

3p: white solid, m.p. 92-93 °C. ^{1}H NMR (400 MHz, $CDCl_3$) δ 7.12-7.07 (m, 2H), 7.03 (s, 1H), 6.86-6.81 (m, 2H), 3.78 (s, 3H), 3.31-3.23 (m, 1H), 2.91 (dd, *J* = 13.6 Hz, 4.0 Hz, 1H), 2.49 (dd, *J* = 13.6 Hz, 10.8 Hz, 1H), 2.39 (s, 3H), 2.23 (dd, *J* = 15.2 Hz, 5.2 Hz, 1H), 1.95 (dd, *J* = 15.2 Hz, 5.2 Hz, 1H), 1.71-1.63 (m, 1H), 1.42-1.35 (m, 1H), 1.09-1.02 (m, 1H), 0.78-0.70 (m, 1H); ^{13}C NMR (100 MHz, $CDCl_3$) δ 197.7, 158.5, 149.9, 133.7, 130.1, 129.8, 114.2, 55.3, 37.5, 35.7, 35.5, 31.2, 26.7, 12.8, 8.8. FT-IR: ν (cm^{-1}) 2922, 1695, 1508, 1456, 1296, 1211, 1125, 1034. HRMS [ESI] calcd for $C_{17}H_{20}NaO_4S$ $[M+Na]^+$ 343.0975, found 343.0984.

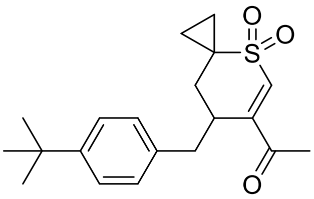

3q: white solid, m.p. 135-136 °C. ^{1}H NMR (500 MHz, $CDCl_3$) δ 7.33-7.30 (m, 2H), 7.14-7.10 (m, 2H), 7.05 (s, 1H), 3.36-3.29 (m, 1H), 2.93 (dd, *J* = 13.5 Hz, 3.5 Hz, 1H), 2.54 (dd, *J* = 13.5 Hz, 11.0 Hz, 1H), 2.39 (s, 3H), 2.29 (dd, *J* = 15.5 Hz, 5.5 Hz, 1H), 1.95 (dd, *J* = 15.5 Hz, 5.5 Hz, 1H), 1.72-1.66 (m, 1H), 1.43-1.37 (m, 1H), 1.30 (s, 9H), 1.13-1.07 (m, 1H), 0.79-0.73 (m, 1H); ^{13}C NMR (125 MHz, $CDCl_3$) δ 197.6, 149.9, 149.8, 135.1, 133.7, 128.5, 125.7, 37.7, 35.4, 35.4, 34.5, 31.4, 31.1, 26.7, 13.0, 8.6. FT-IR: ν (cm^{-1}) 2961, 1694, 1558, 1456, 1362, 1298, 1209, 1126. HRMS [ESI] calcd for $C_{20}H_{26}NaO_3S$ [M+Na]$^+$ 369.1495, found 369.1485.

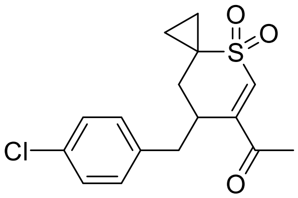

3r: white solid, m.p. 163-164 °C. ^{1}H NMR (500 MHz, $CDCl_3$) δ 7.27 (d, *J* = 8.0 Hz, 2H), 7.13 (d, *J* = 8.0 Hz, 2H), 7.07 (s, 1H), 3.30-3.23 (m, 1H), 2.98-2.91 (m, 1H), 2.51 (dd, *J* = 12.0 Hz, 12.0 Hz, 1H), 2.40 (s, 3H), 2.11 (dd, *J* = 15.5 Hz, 5.5 Hz, 1H), 1.93 (dd, *J* = 15.5 Hz, 5.5 Hz, 1H), 1.70-1.63 (m, 1H), 1.42-1.35 (m, 1H), 1.05-0.99 (m, 1H), 0.77-0.71 (m, 1H); ^{13}C NMR (100 MHz, $CDCl_3$) δ 197.6, 149.2, 136.8, 134.3, 132.7, 130.2, 129.0, 37.7, 35.4, 35.3, 31.2, 26.7, 12.7, 8.9. FT-IR: ν (cm^{-1}) 2922, 1694, 1506, 1491, 1456, 1296, 1211, 1126. HRMS [ESI] calcd for $C_{16}H_{18}ClO_3S$ [M+H]$^+$ 325.0660, found 325.0666.

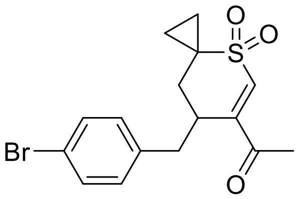

3s: white solid, m.p. 158-159 °C. ^{1}H NMR (400 MHz, $CDCl_3$) δ 7.44-7.40 (m, 2H), 7.08 (s, 1H), 7.09-7.05 (m, 2H), 3.31-3.22 (m, 1H), 2.93 (dd, *J* = 13.6 Hz, 3.6 Hz, 1H), 2.49 (dd, *J* = 13.6 Hz, 11.2 Hz, 1H), 2.41 (s, 3H), 2.20 (dd, *J* = 15.2 Hz, 5.2 Hz, 1H), 1.93 (dd, *J* = 15.2 Hz, 5.6 Hz, 1H), 1.70-1.63 (m, 1H), 1.42-1.35 (m, 1H), 1.05-0.98 (m, 1H), 0.78-0.70 (m, 1H); ^{13}C NMR (100 MHz, $CDCl_3$) δ 197.6, 149.2, 137.3, 134.3, 131.9, 130.5, 120.8, 37.7, 35.4, 35.2, 31.2, 26.7, 12.7, 8.9. FT-IR: ν (cm^{-1}) 2922, 1688, 1634, 1506, 1456, 1296, 1211, 1126. HRMS [ESI] calcd for $C_{17}H_{20}NaO_4S$ [M+Na]$^+$ 343.0975, found 343.0984.

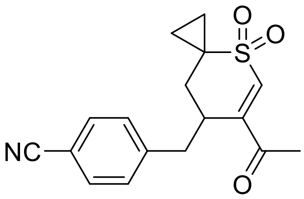

3t: white solid, m.p. 162-163 °C. ^{1}H NMR (400 MHz, $CDCl_3$) δ 7.59 (d, *J* = 8.0 Hz, 2H), 7.32 (d, *J* = 8.0 Hz, 2H), 7.13 (s, 1H), 3.34-3.26 (m, 1H), 3.03 (dd, *J* = 13.6 Hz, 3.6 Hz, 1H), 2.60 (dd, *J* = 13.6 Hz, 11.2 Hz, 1H), 2.41 (s, 3H), 2.20 (dd, *J* = 15.2 Hz, 5.2 Hz, 1H), 1.91 (dd, *J* = 15.2 Hz, 5.6 Hz, 1H), 1.71-1.63 (m, 1H), 1.42-1.34 (m, 1H), 1.04-0.96 (m, 1H), 0.79-0.71 (m, 1H); ^{13}C NMR (100 MHz, $CDCl_3$) δ 197.6, 148.6, 144.0, 134.9, 132.6, 129.7, 118.6, 110.9, 38.5, 35.4, 35.0, 31.3, 26.6, 12.7, 8.9. FT-IR: ν (cm^{-1}) 2922, 2228, 1684, 1506, 1456, 1296, 1211, 1125. HRMS [ESI] calcd for $C_{17}H_{18}NO_3S$ [M+H]$^+$ 316.1002, found 316.0993.

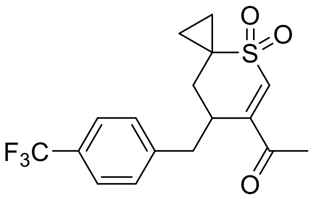

3u: white solid, m.p. 150-151 °C. ^{1}H NMR (400 MHz, $CDCl_3$) δ 7.56 (d, *J* = 8.0 Hz, 2H), 7.33 (d, *J* = 8.0 Hz, 2H), 7.11 (s, 1H), 3.37-3.28 (m, 1H), 3.05 (dd, *J* = 13.6 Hz, 2.4 Hz, 1H), 2.60 (dd, *J* = 13.6 Hz, 11.2 Hz, 1H), 2.42 (s, 3H), 2.21 (dd, *J* = 15.2 Hz, 5.6 Hz, 1H), 1.94 (dd, *J* = 15.2 Hz, 5.6 Hz, 1H), 1.72-1.65 (m, 1H), 1.44-1.36 (m, 1H), 1.07-0.99 (m, 1H), 0.79-0.71 (m, 1H); ^{13}C NMR (125 MHz, $CDCl_3$) δ 197.6, 148.9, 142.5 (q, $J_{C\text{-}F}$ = 0.9 Hz), 134.6, 129.3 (q, $J_{C\text{-}F}$ = 32.5 Hz), 129.2, 125.8 (q, $J_{C\text{-}F}$ = 3.8 Hz), 124.1 (q, $J_{C\text{-}F}$ = 270.3 Hz), 38.1, 35.4, 35.1, 31.2, 26.6, 12.7, 8.9; ^{19}F NMR (376 MHz, $CDCl_3$) δ -62.5 (s). FT-IR: ν (cm^{-1}) 2922, 2359, 1688, 1506, 1456, 1327, 1298, 1126. HRMS [ESI] calcd for $C_{17}H_{17}F_3NaO_3S$ [M+Na]$^+$ 381.0743, found 381.0737.

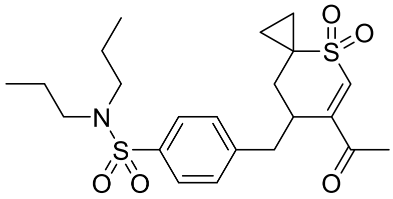

3v: yellow oil. ^{1}H NMR (400 MHz, $CDCl_3$) δ 7.72 (d, *J* = 8.0 Hz, 2H), 7.32 (d, *J* = 8.0 Hz, 2H), 7.11 (s, 1H), 3.35-3.26 (m, 1H), 3.09-3.00 (m, 5H), 2.60 (dd, *J* = 13.6 Hz, 11.2 Hz, 1H), 2.41 (s, 3H), 2.18 (dd, *J* = 15.2 Hz, 5.6 Hz, 1H), 1.91 (dd, *J* = 15.2 Hz, 5.6 Hz, 1H), 1.71-1.62 (m, 1H), 1.58-1.46 (m, 4H), 1.42-1.34 (m, 1H), 1.04-0.97 (m, 1H), 0.84 (t, *J* = 7.2 Hz, 6H), 0.77-0.70 (m, 1H); ^{13}C NMR (100 MHz, $CDCl_3$) δ 197.6, 148.8, 143.1, 139.0, 134.7, 129.4, 127.6, 49.9, 38.2, 35.4, 35.1, 31.3, 26.6, 22.0, 12.6, 11.2, 8.9. FT-IR: ν (cm^{-1}) 2967, 2876, 1686, 1506, 1373, 1298, 1155, 1126. HRMS [ESI] calcd for $C_{22}H_{32}NO_5S_2$ [M+H]$^+$ 454.1716, found 454.1723.

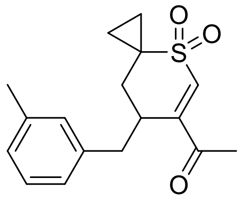

3w: white solid, m.p. 76-77 °C. ^{1}H NMR (400 MHz, $CDCl_3$) δ 7.22-7.16 (m, 1H), 7.07-6.96 (m, 4H), 3.37-3.28 (m, 1H), 2.95 (dd, *J* = 13.6 Hz, 3.2 Hz, 1H), 2.50 (dd, *J* = 12.8 Hz, 11.6 Hz, 1H), 2.40 (s, 3H), 2.33 (s, 3H), 2.25 (dd, *J* = 15.2 Hz, 5.2 Hz, 1H), 1.96 (dd, *J* = 15.2 Hz, 5.2 Hz, 1H), 1.72-1.64 (m, 1H), 1.44-1.36 (m, 1H), 1.12-1.04 (m, 1H), 0.79-0.71 (m, 1H); ^{13}C NMR (100 MHz, $CDCl_3$) δ 197.7, 149.8, 138.5, 138.1, 133.8, 129.5, 128.7, 127.6, 125.9, 38.3, 35.5, 35.4, 31.2, 26.7, 21.4, 12.8, 8.8. FT-IR: ν (cm^{-1}) 2922, 1694, 1609, 1506, 1456, 1296, 1211, 1126. HRMS [ESI] calcd for $C_{17}H_{20}NaO_3S$ [M+Na]$^+$ 327.1025, found 327.1030.

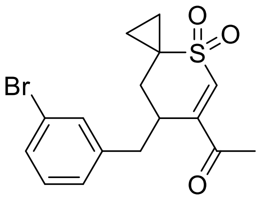

3x: white solid, m.p. 61-62 °C. ^{1}H NMR (400 MHz, $CDCl_3$) δ 7.39-7.33 (m, 2H), 7.21-7.11 (m, 2H), 7.08 (s, 1H), 3.33-3.25 (m, 1H), 2.97 (dd, *J* = 13.6 Hz, 3.6 Hz, 1H), 2.51 (dd, *J* = 13.6 Hz, 10.8 Hz, 1H), 2.41 (s, 3H), 2.24 (dd, *J* = 15.2 Hz, 5.2 Hz, 1H), 1.95 (dd, *J* = 15.2 Hz, 5.6 Hz, 1H), 1.72-1.65 (m, 1H), 1.44-1.36 (m, 1H), 1.07-1.00 (m, 1H), 0.80-0.73 (m, 1H); ^{13}C NMR (100 MHz, $CDCl_3$) δ 192.8, 144.3, 135.9, 129.7, 127.0, 125.6, 125.4, 122.8, 118.1, 33.3, 30.7, 30.5, 26.5, 21.9, 8.0, 4.1. FT-IR: ν (cm^{-1}) 2920, 1684, 1506, 1473, 1361, 1296, 1211, 1125. HRMS [ESI] calcd for $C_{16}H_{18}BrO_3S$ [M+H]$^+$ 369.0155, found 369.0163.

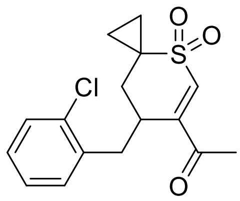

3y: white solid, m.p. 69-70 °C. ^{1}H NMR (400 MHz, $CDCl_3$) δ 7.36-7.32 (m, 1H), 7.25-7.14 (m, 3H), 7.01 (s, 1H), 3.54-3.46 (m, 1H), 3.03 (dd, *J* = 13.6 Hz, 5.6 Hz, 1H), 2.87 (dd, *J* = 13.6 Hz, 5.6 Hz, 1H), 2.32 (s, 3H), 2.21 (dd, *J* = 15.2 Hz, 5.6 Hz, 1H), 2.09 (dd, *J* = 15.2 Hz, 6.0 Hz, 1H), 1.70-1.62 (m, 1H), 1.43-1.36 (m, 1H), 1.12-1.05 (m, 1H), 0.81-0.74 (m, 1H); ^{13}C NMR (100 MHz, $CDCl_3$) δ 197.6, 150.1, 135.7, 134.3, 133.5, 130.8, 130.0, 128.4, 127.1, 36.3, 35.6, 34.0, 32.4, 26.7, 12.4, 9.1. FT-IR: ν (cm^{-1}) 2924, 1694, 1633, 1506, 1473, 1296, 1211, 1125. HRMS [ESI] calcd for $C_{16}H_{18}ClO_3S$ [M+H]$^+$ 325.0660, found 325.0652.

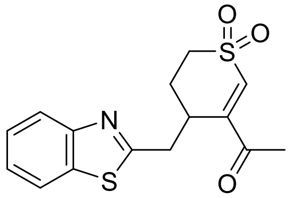

3z: white solid, m.p. 133-134 °C. ^{1}H NMR (400 MHz, $CDCl_3$) δ 7.99-7.95 (m, 1H), 7.88-7.84 (m, 1H), 7.51-7.45 (m, 1H), 7.42-7.36 (m, 1H), 7.04 (s, 1H), 3.54-3.43 (m, 2H), 3.31 (dd, *J* = 14.8 Hz, 4.0 Hz, 1H), 3.23-3.16 (m, 2H), 2.50-2.42 (m, 1H), 2.45 (s, 3H), 2.42-2.36 (m, 1H); ^{13}C NMR (100 MHz, $CDCl_3$) δ 196.8, 167.0, 153.1, 147.2, 135.4, 135.3, 126.3, 125.4, 122.9, 121.7, 46.7, 35.2, 32.5, 26.2, 23.9. FT-IR: ν (cm^{-1}) 2922, 1688, 1514, 1435, 1358, 1296, 1215, 1126. HRMS [ESI] calcd for $C_{15}H_{16}NO_3S_2$ [M+H]$^+$ 322.0566, found 322.0572.

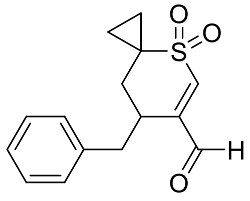

3aa: yellow solid, m.p. 75-76 °C. ^{1}H NMR (400 MHz, $CDCl_3$) δ 9.57 (s, 1H), 7.35-7.30 (m, 2H), 7.25-7.19 (m, 3H), 7.13 (s, 1H), 3.23-3.15 (m, 2H), 2.55 (dd, *J* = 14.0 Hz, 12.0 Hz, 1H), 2.29 (dd, *J* = 15.6 Hz, 5.2 Hz, 1H), 1.95 (dd, *J* = 15.6 Hz, 4.8 Hz, 1H), 1.75-1.67 (m, 1H), 1.46-1.38 (m, 1H), 1.13-1.07 (m, 1H), 0.79-0.72 (m, 1H); ^{13}C NMR (125 MHz, $CDCl_3$) δ 191.2, 148.9, 142.0, 138.1, 128.9, 128.8, 127.0, 37.7, 36.1, 34.3, 31.0, 12.8, 8.7. FT-IR: ν (cm^{-1}) 2922, 1697, 1558, 1456, 1417, 1361, 1298, 1138. HRMS [ESI] calcd for $C_{15}H_{17}O_3S$ [M+H]$^+$ 277.0893, found 277.0895.

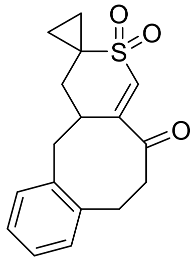

5a: yellow oil. ^{1}H NMR (400 MHz, $CDCl_3$) δ 7.25-7.15 (m, 3H), 7.10-7.06 (m, 1H), 6.29 (s, 1H), 3.21-3.13 (m, 1H), 3.08-3.01 (m, 1H), 3.00-2.86 (m, 4H), 2.67-2.54 (m, 2H), 2.26-2.18 (m, 1H), 1.65-1.58 (m, 1H), 1.47-1.40 (m, 1H), 1.05-0.98 (m, 1H), 0.97-0.90 (m, 1H); ^{13}C NMR (100 MHz, $CDCl_3$) δ 204.6, 154.2, 138.3, 135.4, 131.4, 130.1, 128.3, 127.6, 125.1, 46.0, 38.4, 37.8, 36.4, 34.4, 29.8, 10.6, 10.4. FT-IR: ν (cm^{-1}) 2920, 2849, 1697, 1558, 1456, 1294, 1230, 1120. HRMS [ESI] calcd for $C_{17}H_{19}O_3S$ [M+H]$^+$ 303.1049, found 303.1042.

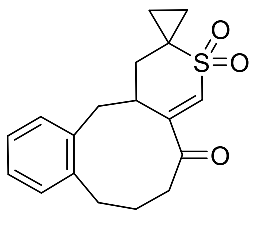

5b: white solid, m.p. 172-173 °C. ^{1}H NMR (500 MHz, $CDCl_3$) δ 7.21-7.17 (m, 2H), 7.13-7.07 (m, 2H), 6.42 (s, 1H), 3.28-3.19 (m, 1H), 2.90-2.76 (m, 4H), 2.75-2.68 (m, 1H), 2.51-2.39 (m, 2H), 2.33-2.24 (m, 1H), 2.16-2.04 (m, 2H), 1.66 (dd, *J* = 10.5 Hz, 5.0 Hz, 1H), 1.45-1.39 (m, 1H), 1.07-0.96 (m, 2H); ^{13}C NMR (125 MHz, $CDCl_3$) δ 204.3, 153.0, 131.7, 130.7, 129.6, 128.5, 127.8,

127.3, 125.2, 38.2, 37.1, 36.9, 30.0, 18.9, 12.8, 12.6, 11.0, 9.9. FT-IR: ν (cm^{-1}) 2920, 1694, 1633, 1506, 1418, 1294, 1142, 1121. HRMS [ESI] calcd for $C_{18}H_{21}O_3S$ $[M+H]^+$ 317.1206, found 317.1196.

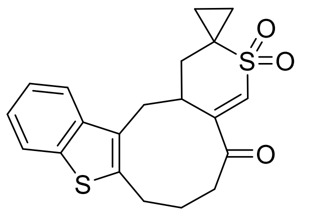

5c: yellow solid, m.p. 203-204 °C. ^1H NMR (400 MHz, $CDCl_3$) δ 7.79-7.75 (m, 1H), 7.64-7.60 (m, 1H), 7.38-7.30 (m, 2H), 6.39 (s, 1H), 3.50-3.42 (m, 1H), 3.40-3.30 (m, 1H), 3.29-3.11 (m, 1H), 2.92-2.84 (m, 2H), 2.83-2.73 (m, 1H), 2.45-2.34 (m, 2H), 2.32-2.18 (m, 2H), 1.99-1.85 (m, 1H), 1.67-1.59 (m, 1H), 1.45-1.38 (m, 1H), 1.05-0.95 (m, 2H); ^{13}C NMR (125 MHz, $CDCl_3$) δ 204.6, 152.7, 139.1, 135.7, 131.9, 128.4, 124.8, 124.4, 122.6, 121.7, 38.2, 37.7, 37.2, 34.9, 30.7, 24.5, 22.9, 11.4, 9.6. FT-IR: ν (cm^{-1}) 2924, 2359, 1695, 1506, 1456, 1296, 1202, 1121. HRMS [ESI] calcd for $C_{20}H_{21}O_3S_2$ $[M+H]^+$ 373.0927, found 373.0922.

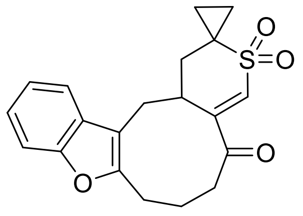

5d: white solid, m.p. 176-177 °C. ^1H NMR (400 MHz, $CDCl_3$) δ 7.45-7.40 (m, 2H), 7.31-7.26 (m, 1H), 7.24-7.29 (m, 1H), 6.37 (d, J = 1.6 Hz, 1H), 3.49-3.41 (m, 1H), 3.25-3.12 (m, 2H), 2.78-2.65 (m, 3H), 2.58-2.49 (m, 1H), 2.42-2.26 (m, 2H), 2.23-2.13 (m, 1H), 1.95 (dd, J = 15.2 Hz, 5.2 Hz, 1H), 1.65-1.58 (m, 1H), 1.45-1.39 (m, 1H), 1.04-0.97 (m, 2H); ^{13}C NMR (100 MHz, $CDCl_3$) δ 204.8, 154.6, 153.0, 150.8, 128.3, 128.2, 124.7, 122.6, 119.1, 115.6, 111.3, 38.3, 37.4, 37.2, 34.9, 29.0, 24.6, 20.1, 11.4, 9.6. FT-IR: ν (cm^{-1}) 2926, 1695, 1616, 1506, 1456, 1296, 1123, 1032. HRMS [ESI] calcd for $C_{20}H_{21}O_4S$ $[M+H]^+$ 357.1155, found 357.1153.

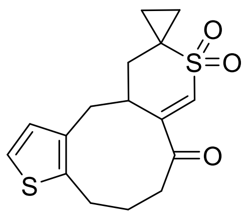

5e: yellow solid, m.p. 157-158 °C. ^1H NMR (500 MHz, $CDCl_3$) δ 7.18 (d, J = 5.5 Hz, 1H), 6.73 (d, J = 5.5 Hz, 1H), 6.40 (d, J = 1.5 Hz, 1H), 3.39-3.32 (m, 1H), 3.30-3.22 (m, 1H), 3.16-3.02 (m, 1H), 2.73-2.57 (m, 3H), 2.50-2.37 (m, 2H), 2.23-2.15 (m, 1H), 2.12-2.02 (m, 1H), 1.94-1.86 (m, 1H), 1.64-1.58 (m, 1H), 1.43-1.38 (m, 1H), 1.03-0.94 (m, 2H); ^{13}C NMR (125 MHz, $CDCl_3$) δ 204.3, 152.9, 138.0, 134.4, 128.9, 128.3, 125.1, 39.0, 37.9, 37.0, 34.9, 30.1, 26.0, 24.9, 11.3, 9.6. FT-IR: ν (cm^{-1}) 2920, 2849, 1696, 1506, 1456, 1296, 1152, 1123. HRMS [ESI] calcd for $C_{16}H_{19}O_3S_2$ $[M+H]^+$ 323.0770, found 323.0768.

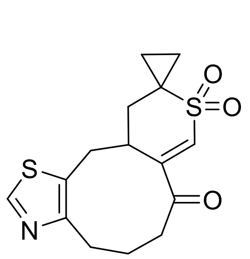

5f: yellow solid, m.p. 145-146 °C. ^1H NMR (400 MHz, $CDCl_3$) δ 8.66 (s, 1H), 6.43 (d, J = 1.6 Hz, 1H), 3.42-3.34 (m, 1H), 3.31-3.24 (m, 1H), 3.02-2.92 (m, 1H), 2.86-2.76 (m, 2H), 2.75-2.68 (m, 1H), 2.47-2.40 (m, 2H), 2.31-2.23 (m, 1H), 2.22-2.14 (m, 1H), 1.93-1.86 (m, 1H), 1.63-1.57 (m, 1H), 1.41-1.36 (m, 1H), 1.01-0.93 (m, 2H); ^{13}C NMR (100 MHz, $CDCl_3$) δ 204.6, 152.9, 152.4, 152.2, 129.0, 128.4, 38.2, 37.8, 36.8, 34.4, 28.6, 25.5, 25.0, 11.3, 9.6. FT-IR: ν (cm^{-1})

2926, 2855, 1689, 1603, 1454, 1346, 1152, 1072. HRMS [ESI] calcd for $C_{15}H_{18}NO_3S_2$ $[M+H]^+$ 324.0723, found 324.0724.

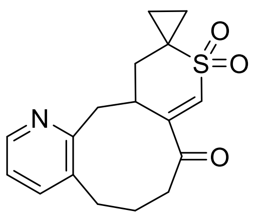

5g: yellow oil. ^{1}H NMR (400 MHz, $CDCl_3$) δ 8.44 (dd, *J* = 4.8 Hz, 1.6 Hz, 1H), 7.40 (dd, *J* = 7.6 Hz, 1.2 Hz, 1H), 7.12 (dd, *J* = 7.6 Hz, 4.8 Hz, 1H), 6.38 (d, *J* = 1.6 Hz, 1H), 3.41-3.32 (m, 1H), 3.15-2.65 (m, 5H), 2.57-2.48 (m, 1H), 2.41-2.22 (m, 2H), 2.14-1.98 (m, 2H), 1.60-1.54 (m, 1H), 1.42-1.36 (m, 1H), 1.05-0.95 (m, 2H); ^{13}C NMR (100 MHz, $CDCl_3$) δ 204.6, 156.8, 153.3, 148.3, 138.5, 134.1, 127.4, 122.7, 38.7, 38.6, 37.3, 35.9, 30.2, 25.7, 22.7, 11.4, 9.4. FT-IR: ν (cm^{-1}) 2924, 2865, 1701, 1574, 1454, 1296, 1197, 1123. HRMS [ESI] calcd for $C_{17}H_{20}NO_3S$ $[M+H]^+$ 318.1158, found 318.1164.

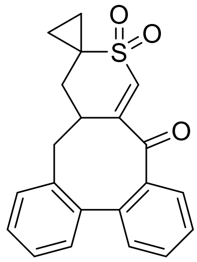

5h: yellow solid, m.p. 164-165 °C. ^{1}H NMR (400 MHz, $CDCl_3$) δ 8.08-8.04 (m, 1H), 7.67-7.62 (m, 1H), 7.53-7.48 (m, 1H), 7.44-7.41 (m, 2H), 7.39-7.35 (m, 1H), 7.27-7.22 (m, 2H), 6.37 (s, 1H), 3.27-3.15 (m, 2H), 3.07 (dd, *J* = 14.8 Hz, 9.6 Hz, 1H), 2.58-2.52 (m, 1H), 1.78 (dd, *J* = 14.8 Hz, 5.6 Hz, 1H), 1.59-1.54 (m, 1H), 1.42-1.36 (m, 1H), 0.99-0.89 (m, 2H); ^{13}C NMR (100 MHz, $CDCl_3$) δ 194.7, 152.4, 141.6, 139.6, 135.7, 133.9, 133.0, 130.8, 130.1, 130.1, 129.5, 128.5, 128.2, 127.9, 38.1, 36.6, 35.7, 33.0, 11.2, 9.3. FT-IR: ν (cm^{-1}) 2924, 2357, 1694, 1591, 1477, 1296, 1231, 1123. HRMS [ESI] calcd for $C_{21}H_{19}O_3S$ $[M+H]^+$ 351.1049, found 351.1052.

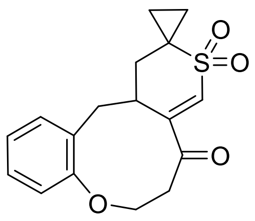

5i: yellow solid, m.p. 164-165 °C. ^{1}H NMR (500 MHz, $CDCl_3$) δ 7.27-7.22 (m, 1H), 7.04-7.01 (m, 2H), 6.95-6.92 (m, 1H), 6.58 (s, 1H), 4.74-4.69 (m, 1H), 4.22-4.15 (m, 1H), 3.46 (dd, *J* = 14.0 Hz, 4.0 Hz, 1H), 3.43-3.36 (m, 1H), 3.10-3.03 (m, 2H), 2.81 (dd, *J* = 15.0 Hz, 11.0 Hz, 1H), 2.31 (dd, *J* = 14.0 Hz, 4.0 Hz, 1H), 1.63 (dd, *J* = 15.0 Hz, 5.0 Hz, 1H), 1.54-1.49 (m, 1H), 1.38-1.33 (m, 1H), 0.98-0.89 (m, 2H); ^{13}C NMR (125 MHz, $CDCl_3$) δ 200.5, 158.3, 150.8, 132.3, 129.9, 129.1, 127.6, 123.5, 118.9, 69.7, 40.8, 36.9, 35.9, 32.4, 32.1, 11.6, 8.9. FT-IR: ν (cm^{-1}) 2924, 1699, 1506, 1489, 1456, 1296, 1248, 1124. HRMS [ESI] calcd for $C_{17}H_{19}O_4S$ $[M+H]^+$ 319.0999, found 319.1007.

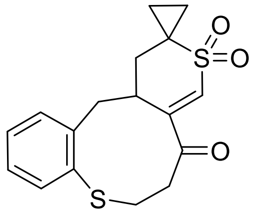

5j: yellow solid, m.p. 174-175 °C. ^{1}H NMR (400 MHz, $CDCl_3$) δ 7.59-7.54 (m, 1H), 7.34-7.28 (m, 1H), 7.25-7.16 (m, 2H), 6.59 (s, 1H), 3.39-3.31 (m, 1H), 3.30-3.05 (m, 5H), 2.69-2.58 (m, 2H), 2.34-2.22 (m, 1H), 1.68-1.60 (m, 1H), 1.45-1.37 (m, 1H), 1.10-1,03 (m, 1H), 0.98-0.92 (m, 1H); ^{13}C NMR (125 MHz, $CDCl_3$) δ 201.6, 152.2, 143.1, 137.6, 134.4, 130.7, 129.8, 128.5, 55.2, 39.9, 38.2, 37.7, 36.4, 34.4, 10.7, 10.3. FT-IR: ν (cm^{-1}) 2920, 2849, 1697, 1506, 1456, 1296, 1219, 1121. HRMS [ESI] calcd for $C_{17}H_{19}O_3S_2$ $[M+H]^+$ 335.0770, found 335.0764.

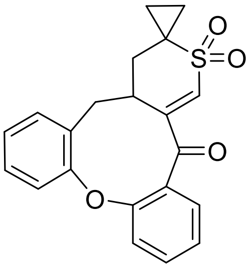

5k: yellow solid, m.p. 164-165 °C. ^{1}H NMR (400 MHz, $CDCl_3$) δ 7.62-7.55 (m, 1H), 7.47-7.42 (m, 1H), 7.40-7.35 (m, 1H), 7.29-7.23 (m, 1H), 7.15-7.09 (m, 2H), 7.06-7.00 (m, 1H), 6.71-6.64 (m, 1H), 6.23 (s, 1H), 3.68-3.55 (m, 1H), 3.27-3.19 (m, 1H), 3.00-2.87 (m, 1H), 2.76-2.68 (m, 1H), 2.01-1.92 (m, 1H), 1.82-1.74 (m, 1H), 1.55-1.47 (m, 1H), 1.20-1.12 (m, 1H), 0.93-0.85 (m, 1H); ^{13}C NMR (125 MHz, $CDCl_3$) δ 195.2, 158.7, 158.6, 158.3, 155.2, 133.7, 131.3, 129.7, 129.3, 129.2, 126.8, 125.2, 124.9, 123.0, 121.0, 38.6, 37.9, 36.0, 35.8, 14.1, 8.4. FT-IR: ν (cm^{-1}) 2922, 2850, 1695, 1636, 1456, 1296, 1227, 1126. HRMS [ESI] calcd for $C_{21}H_{19}O_4S$ $[M+H]^+$ 367.0999, found 367.0992.

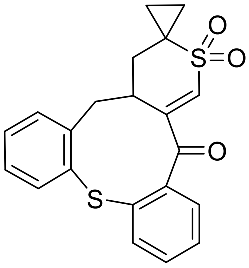

5l: yellow solid, m.p. 156-157 °C. ^{1}H NMR (400 MHz, $CDCl_3$) δ 7.69-7.65 (m, 1H), 7.49-7.43 (m, 1H), 7.35-7.27 (m, 2H), 7.27-7.17 (m, 3H), 7.11-7.05 (m, 1H), 6.31 (s, 1H), 4.15-4.06 (m, 1H), 3.45-3.36 (m, 1H), 3.04 (dd, *J* = 15.2 Hz, 4.4 Hz, 1H), 2.74-2.68 (m, 1H), 2.06-1.99 (m, 1H), 1.86-1.78 (m, 1H), 1.54-1.46 (m, 1H), 1.24-1.18 (m, 1H), 0.95-0.87 (m, 1H); ^{13}C NMR (125 MHz, $CDCl_3$) δ 196.1, 152.6, 142.3, 139.5, 139.4, 136.7, 135.4, 133.0, 131.8, 130.3, 130.3, 130.2, 128.9, 127.8, 127.7, 40.4, 38.0, 35.7, 35.4, 13.8, 8.2. FT-IR: ν (cm^{-1}) 2924, 2850, 1695, 1506, 1456, 1296, 1224, 1126. HRMS [ESI] calcd for $C_{21}H_{19}O_3S_2$ $[M+H]^+$ 383.0770, found 383.0763.

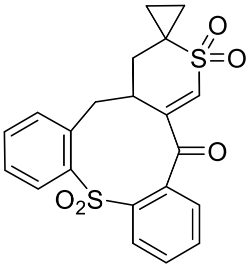

5m: white solid, m.p. 157-158 °C. ^{1}H NMR (400 MHz, $CDCl_3$) δ 8.31 (d, *J* = 8.0 Hz, 1H), 7.77-7.70 (m, 1H), 7.66-7.60 (m, 1H), 7.58-7.48 (m, 2H), 7.35-7.28 (m, 3H), 6.40 (s, 1H), 4.93-4.84 (m, 1H), 3.52-3.42 (m, 1H), 3.16 (dd, *J* = 15.2 Hz, 5.2 Hz, 1H), 2.73 (dd, *J* = 12.4 Hz, 3.6 Hz, 1H), 1.94 (dd, *J* = 15.2 Hz, 2.4 Hz, 1H), 1.89-1.82 (m, 1H), 1.55-1.48 (m, 1H), 1.28-1.21 (m, 1H), 0.96-0.89 (m, 1H); ^{13}C NMR (100 MHz, $CDCl_3$) δ 195.2, 150.5, 141.6, 140.0, 138.4, 136.8, 135.2, 133.8, 133.1, 133.0, 131.9, 131.5, 129.2, 129.1, 126.3, 38.0, 37.5, 35.2, 34.9, 14.4, 7.8. FT-IR: ν (cm^{-1}) 2920, 2851, 1682, 1516, 1436, 1298, 1264, 1126. HRMS [ESI] calcd for $C_{21}H_{19}O_5S_2$ $[M+H]^+$ 415.0668 found 415.0666.

5. Mechanistic studies

a) Radical trapping experiments

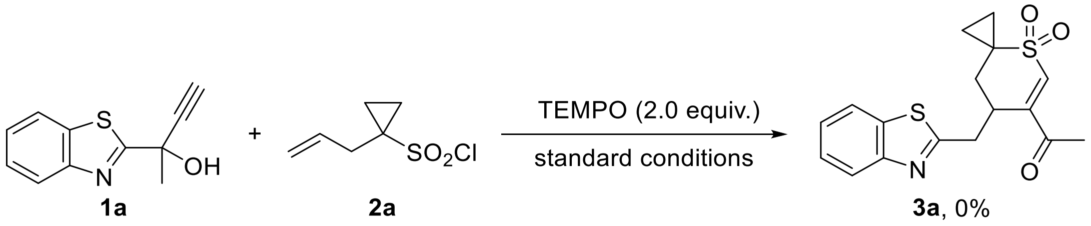

Propargyl alcohol **1a** (0.2 mmol, 40.7 mg), Na_2HPO_4 (0.2 mmol, 28.4 mg), TEMPO

(0.6 mmol, 93.8 mg), and *fac*-Ir(ppy)$_3$ (3 mol %, 4.0 mg) were loaded in a reaction vial which was subjected to evacuation/ flushing with N_2 three times. Then allylcyclopropane sulfonyl chloride **2a** (0.3 mmol, 55.9 mg) in CH_3CN (2 mL) and H_2O (0.2 mL) were added to the mixture via syringe. The reaction was irradiated with 12 W blue LEDs. No desired product **3a** was detected by TLC.

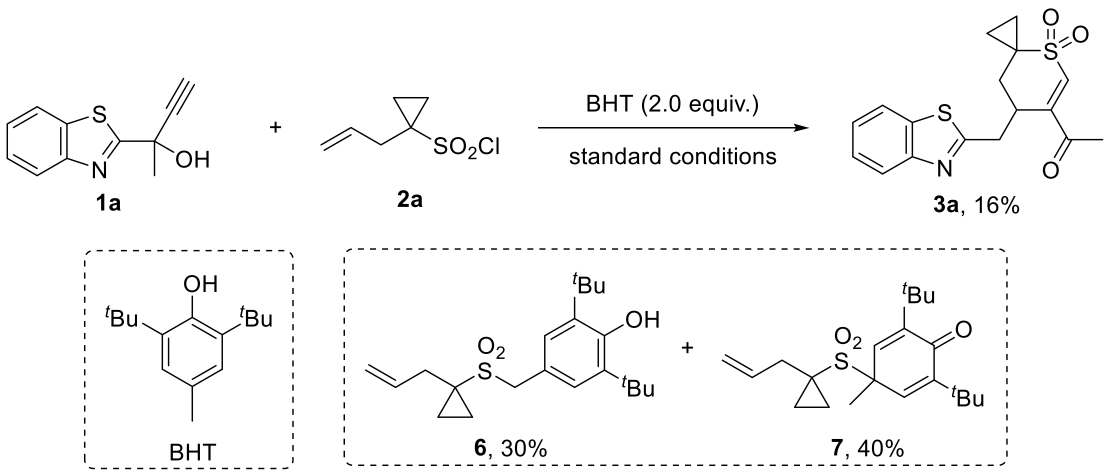

Propargyl alcohol **1a** (0.2 mmol, 40.7 mg), Na_2HPO_4 (0.2 mmol, 28.4 mg), BHT (0.4 mmol, 88.1 mg), and *fac*-Ir(ppy)$_3$ (3 mol %, 4 mg) were loaded in a reaction vial which was subjected to evacuation/ flushing with N_2 three times. Then allylcyclopropane sulfonyl chloride **2a** (0.3 mmol, 55.9 mg) in CH_3CN (2 mL) and H_2O (0.2 mL) were added to the mixture via syringe. The reaction was irradiated with 12 W blue LEDs. After reaction completion, the reaction mixture was concentrated in vacuo. Purification the residue by flash column chromatography on silica gel afforded the desired product **3a** in a 16% yield, radical trapping adducts **6** in a 30% yield and **7** in a 40% yield.

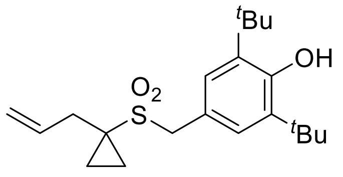

6: yellow solid, m.p. 64-65 °C. ^1H NMR (500 MHz, $CDCl_3$) δ 7.17 (s, 2H), 5.75-5.65 (m, 1H), 5.31 (s, 1H), 5.17-5.09 (m, 2H), 4.21 (s, 2H), 2.61 (d, J = 7.5 Hz, 2H), 1.44 (s, 18H), 1.18-1.14 (m, 2H), 0.75-0.71 (m, 2H); ^{13}C NMR (125 MHz, $CDCl_3$) δ 154.4, 136.3, 132.6, 127.7, 119.4, 118.7, 58.1, 37.8, 34.9, 34.3, 30.3, 9.2. FT-IR: ν (cm^{-1}) 3320, 2967, 2868, 1634, 1471, 1417, 1236, 1109. HRMS [ESI] calcd for $C_{21}H_{32}NaO_3S$ $[M+Na]^+$ 387.1964, found 387.1968.

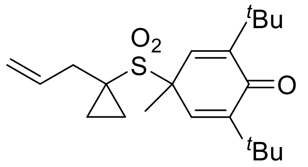

7: white solid, m.p. 115-116 °C. ^1H NMR (500 MHz, $CDCl_3$) δ 6.74 (s, 2H), 5.53-5.42 (m, 1H), 5.02 (d, J = 10.0 Hz, 1H), 4.90 (d, J = 17.0 Hz, 1H), 2.48 (d, J = 7.0 Hz, 2H), 1.65 (s, 3H), 1.34-1.31 (m, 2H), 1.25 (s, 18H), 0.77-0.73 (m, 2H); ^{13}C NMR (125 MHz, $CDCl_3$) δ 184.6, 149.5, 136.9, 131.4, 119.7, 66.8, 39.5, 35.4, 34.7, 29.1, 21.0, 10.0. FT-IR: ν (cm^{-1}) 2961, 2869, 1634, 1417, 1305, 1294, 1246, 1132. HRMS [ESI] calcd for $C_{21}H_{33}O_3S$ $[M+H]^+$ 365.2145, found 365.2147.

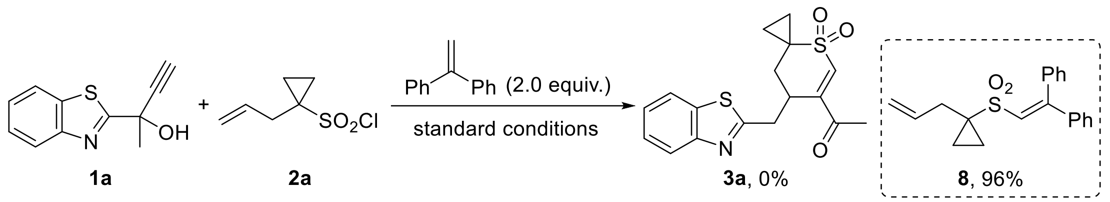

Propargyl alcohol **1a** (0.2 mmol, 40.7 mg), Na_2HPO_4 (0.2 mmol, 28.4 mg), and *fac*-Ir(ppy)$_3$ (3 mol %, 4 mg) were loaded in a reaction vial which was subjected to evacuation/ flushing with N_2 three times. Then allylcyclopropane sulfonyl chloride **2a** (0.3 mmol, 55.9 mg) in CH_3CN (2 mL), 1,1-diphenylethylene (0.4 mmol, 72.1 mg), and H_2O (0.2 mL) were added to the mixture via syringe. The reaction was irradiated with 12 W blue LEDs. No desired product **3a** was detected by TLC. The reaction mixture was concentrated in vacuo. Purification the residue by flash column chromatography on silica gel afforded the radical trapping adduct **8** in a 96% yield.

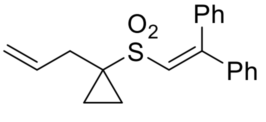

8: yellow oil. ^{1}H NMR (400 MHz, $CDCl_3$) δ 7.43-7.31 (m, 8H), 7.29-7.24 (m, 2H), 6.77 (s, 1H), 5.81-5.69 (m, 1H), 5.17-5.10 (m, 2H), 2.68 (d, *J* = 7.2 Hz, 2H), 1.23-1.19 (m, 2H), 0.78-0.74 (m, 2H); ^{13}C NMR (100 MHz, $CDCl_3$) δ 155.5, 139.8, 136.1, 132.9, 130.2, 129.9, 129.2, 128.7, 128.3, 127.8, 124.9, 119.2, 40.9, 34.9, 9.9. FT-IR: ν (cm^{-1}) 3059, 2920, 1607, 1489, 1445, 1301, 1123, 923. HRMS [ESI] calcd for $C_{20}H_{21}O_3S$ $[M+H]^+$ 325.1257, found 325.1252.

b) Quantum yield measurements

Determination of the light intensity at 456 nm

The photon flux of the kessil light (40 W, λ_{max} = 456 nm) was determined by standard ferrioxalate actinometry following a modified literature procedure of Yoon[1] and Glorius[2]. A 0.15 M solution of ferrioxalate was prepared by dissolving potassium ferrioxalate hydrate (0.737 g) in H_2SO_4 (10 mL of a 0.05 M solution). A buffered solution of 1,10-phenanthroline was prepared by dissolving 1,10-phenanthroline (5.0 mg) and sodium acetate (1.13 g) in H_2SO_4 (5.0 mL of a 0.5 M solution). Both solutions were stored in the dark. To determine the photon flux of the LED, the ferrioxalate solution (3.0 mL) was placed in a cuvette and irradiated for 60 seconds at λ_{max} = 456 nm. After irradiation, the phenanthroline solution (0.525 mL) was added to the cuvette and the mixture was allowed to stir in the dark for 1 h to allow the ferrous ions to completely coordinate to the phenanthroline. The absorbance of the solution was measured at 510 nm. The same procedure was repeated two more times. A nonirradiated sample was also prepared and the absorbance at 510 nm was measured. The average of the absorption of the irradiated and non-irradiated samples was determined and used to calculate the generated amount of Fe(II) according to the Lambert-Beer law (equation 1),

$$\textbf{mol Fe}^{2+} = (V \times \Delta A510\text{nm})/ (l \times \varepsilon) \qquad (1)$$

where V is the total volume ($3.525*10^{-3}$ L), $\Delta A510$nm the difference between absorbance of irradiated samples and the non-irradiated (control) ones (at λ = 510 nm), l is the path length of the cuvette (1.0 cm), and ε is the molar attenuation coefficient of the ferrioxalate actinometer λ = 510 nm (11100 $L\cdot mol^{-1}\cdot cm^{-1}$)[3]. The photonflux can be calculated using equation 2,

$$\textbf{photo flux} = \text{mol } Fe^{2+} / (\Phi_F \times t \times f) \qquad (2)$$

where Φ is the quantum yield of the ferrioxalate actinometer (1.11 at λ = 436 nm)[4] and *t* is the irradiation time (60 s). The fraction of light absorbed at λ = 456 nm by the actinometer (*f*) is calculated by using equation 3. *A*456nm is the absorbance of the ferrioxalate solution at λ = 456 nm.

$$f = 1 - 10^{-A_{456\,nm}} \qquad (3)$$

The absorbance (A_{456nm}) of the ferrioxalate solution was measured to be > 3 indicating that > 99.9% of the photons are absorbed (f > 0.999). The photon flux was therefore calculated to be $5.3*10^{-9}$ einstein s^{-1} as an average of three experiments.

Determination of the quantum yield

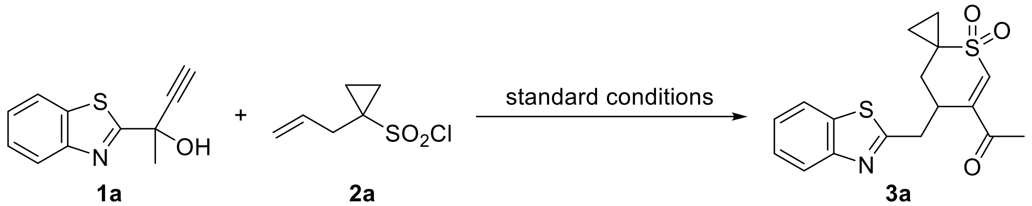

The reaction mixture was stirred and irradiated by blue LED (λ_{max} = 456 nm) for 3600 s. The yield of product was determined by ^1H NMR analysis using toluene as an internal standard. The yield of 3a was determined to be 15.5% (0.0155×10^{-3} mol of **3a**). The reaction quantum yield (Φ) was determined using equation 4 where the photon flux is 5.3×10^{-9} einsteins s^{-1} (determined by actinometry as described above), t is the reaction time (3600 s) and f is the fraction of incident light absorbed by the catalyst, determined using (equation 3).

$$\text{Quantum Yield} = \text{moles of product formed} / (\text{flux} \times f \times t) \qquad (4)$$
$$= 0.0155 \times 10^{-3} / (5.3 \times 10^{-9} \times 1 \times 3600) = 0.81$$

Conclusion: Although the light on/off experiments showed that the product formation occurred only during the periods of constant light irradiation. Typical lifetime of radical chain process can be on the second or sub-second timescale, which means chain processes can terminate faster than the timescale of the analytical measurement used. The quantum yield measurement (Φ < 1) indicated that the reaction mainly proceeded via a photocatalytic pathway, but the contribution of radical chain process could not be ruled out.

1. M. A. Cismesia, T. P. Yoon, *Chem. Sci.* **2015**, *6*, 5426-5434.
2. L. Quach, S. Dutta, P. M. Pflüger, F. Sandfort, P. Belotti, F. Glorius, *ACS Catal.*

2022, *12*, 2499-2504.

3. I. P. Pozdnyakov, O. V. Kel, V. F. Plyusnin, V. P. Grivin, N. M. Bazhin, *J. Phys. Chem. A,* **2008**, *112*, 8316-8322.
4. A. Dewanji, L. Dalsen, J. A. Rossi-Ashton, E. Gasson, G. E. M. Crisenza, D. J. Procter, *Nat. Chem.* **2023**, *15*, 43-52.

6. ^{1}H, ^{13}C, and ^{19}F NMR spectra

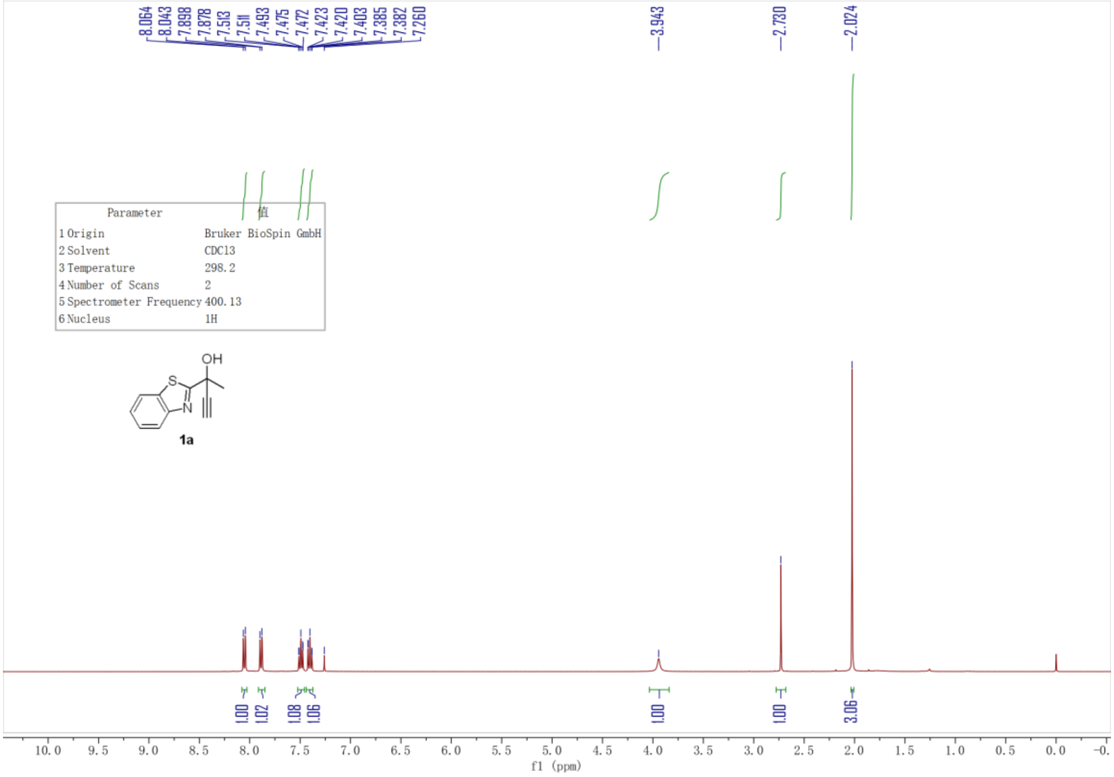

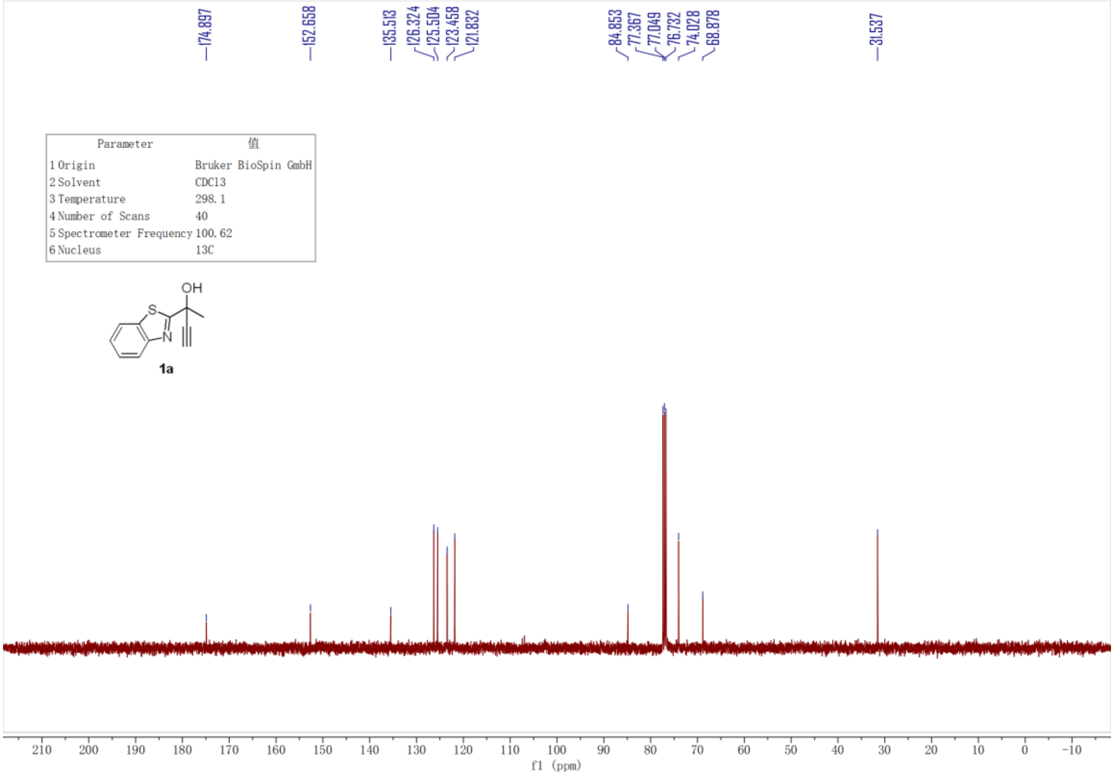

Parameter 值
1 Origin Bruker BioSpin GmbH
2 Solvent CDCl3
3 Temperature 298.1
4 Number of Scans 40
5 Spectrometer Frequency 100.62
6 Nucleus 13C
1a
f1 (ppm)

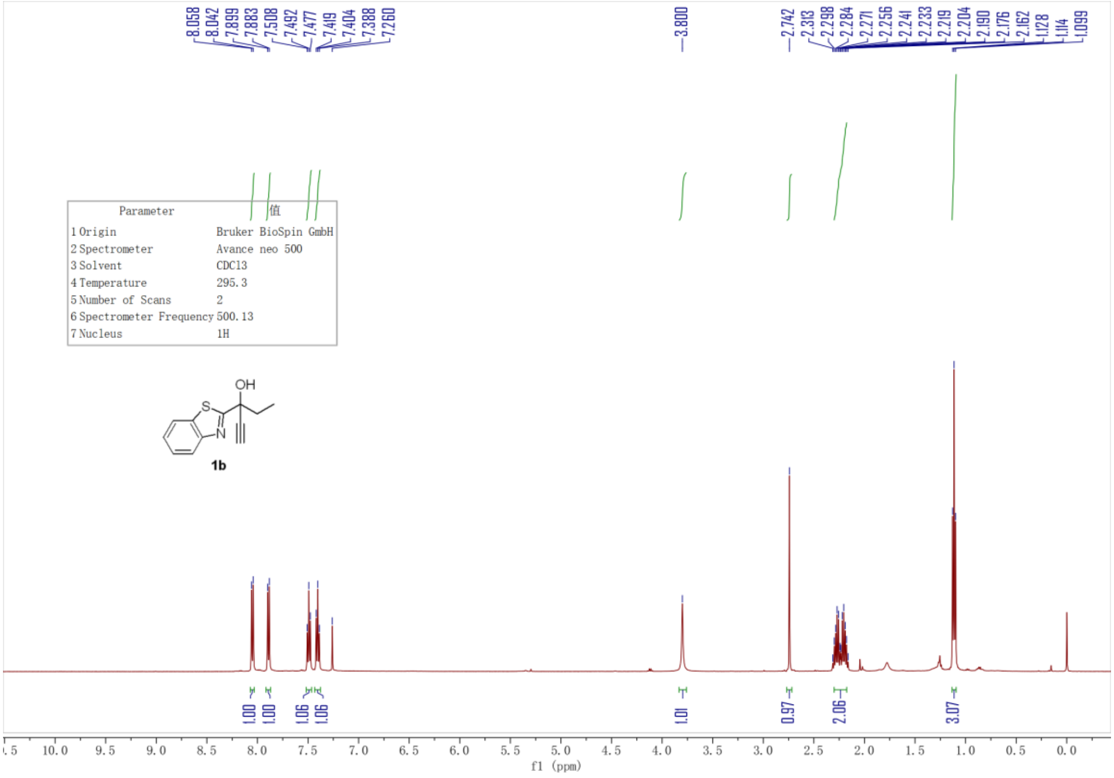

Parameter 值
1 Origin Bruker BioSpin GmbH
2 Spectrometer Avance neo 500
3 Solvent CDCl3
4 Temperature 295.3
5 Number of Scans 2
6 Spectrometer Frequency 500.13
7 Nucleus 1H
1b
f1 (ppm)

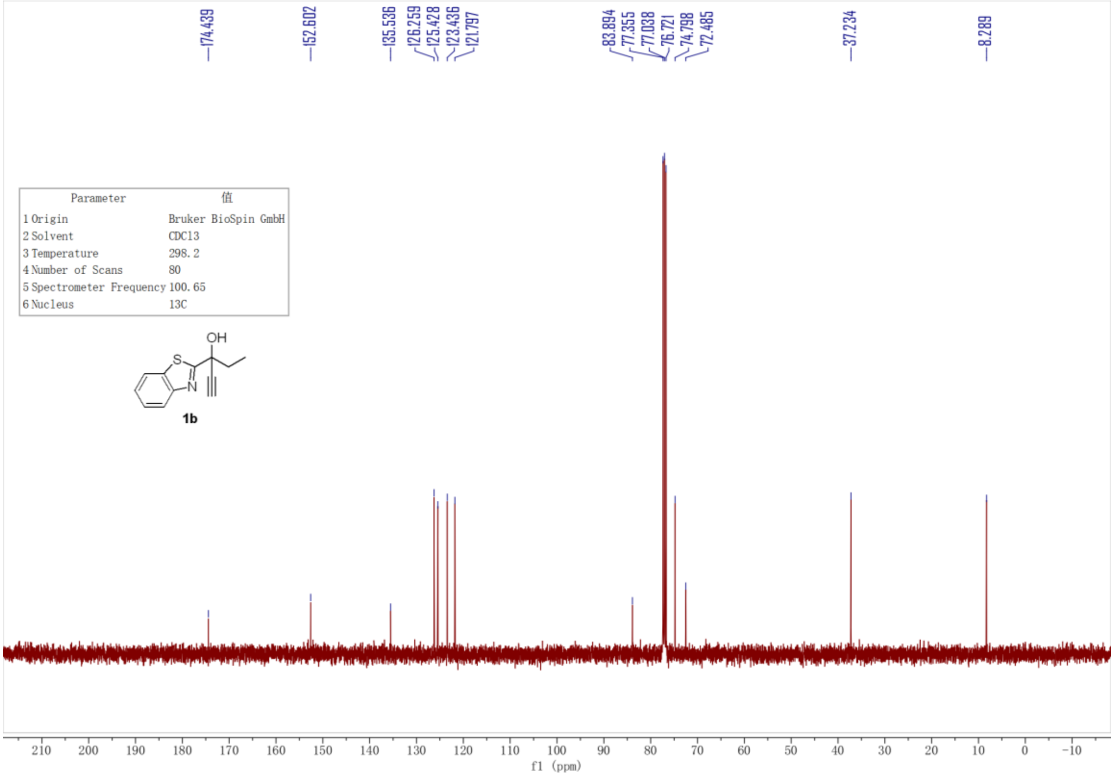
174.439
152.602
135.536
126.259
125.428
123.436
121.797
83.894
77.355
77.038
76.721
74.798
72.485
37.234
8.289
Parameter 值
1 Origin Bruker BioSpin GmbH
2 Solvent CDCl3
3 Temperature 298.2
4 Number of Scans 80
5 Spectrometer Frequency 100.65
6 Nucleus 13C
OH
S
N
1b
f1 (ppm)

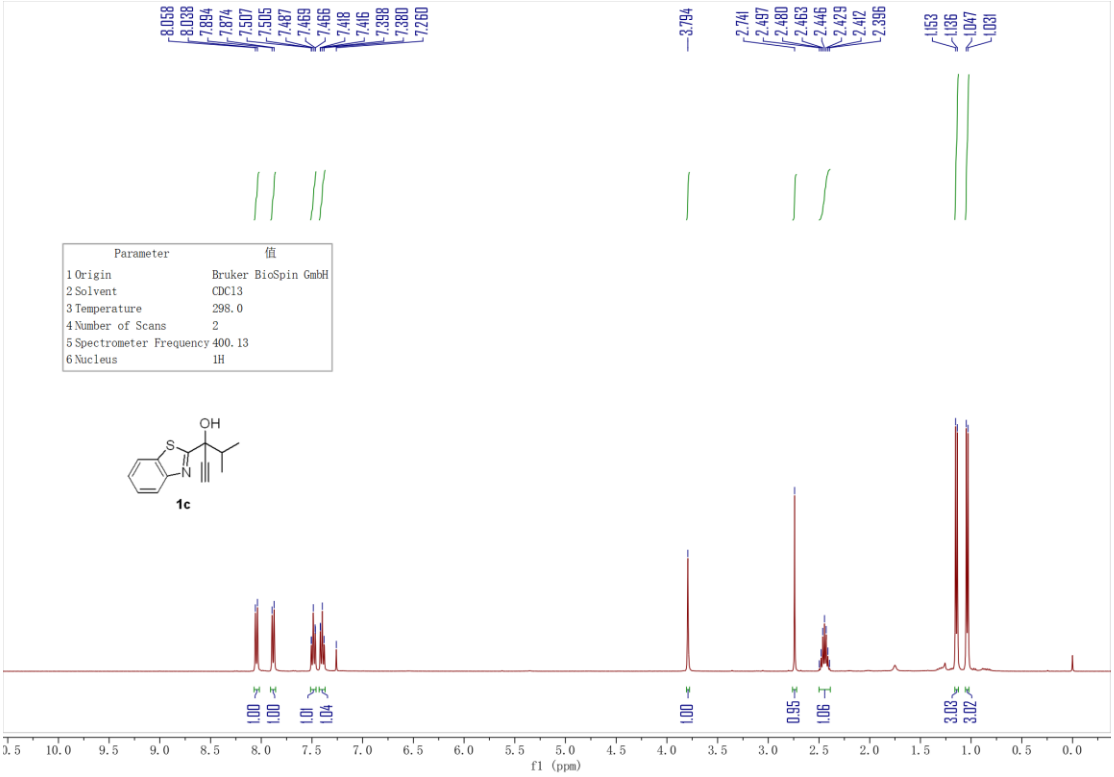
8.058
8.038
7.894
7.874
7.507
7.505
7.487
7.469
7.466
7.418
7.416
7.398
7.380
7.260
3.794
2.741
2.497
2.480
2.463
2.446
2.429
2.412
2.396
1.153
1.136
1.047
1.031
Parameter 值
1 Origin Bruker BioSpin GmbH
2 Solvent CDCl3
3 Temperature 298.0
4 Number of Scans 2
5 Spectrometer Frequency 400.13
6 Nucleus 1H
OH
S
N
1c
1.00
1.00
1.01
1.04
1.00
0.95
1.06
3.03
3.02
f1 (ppm)

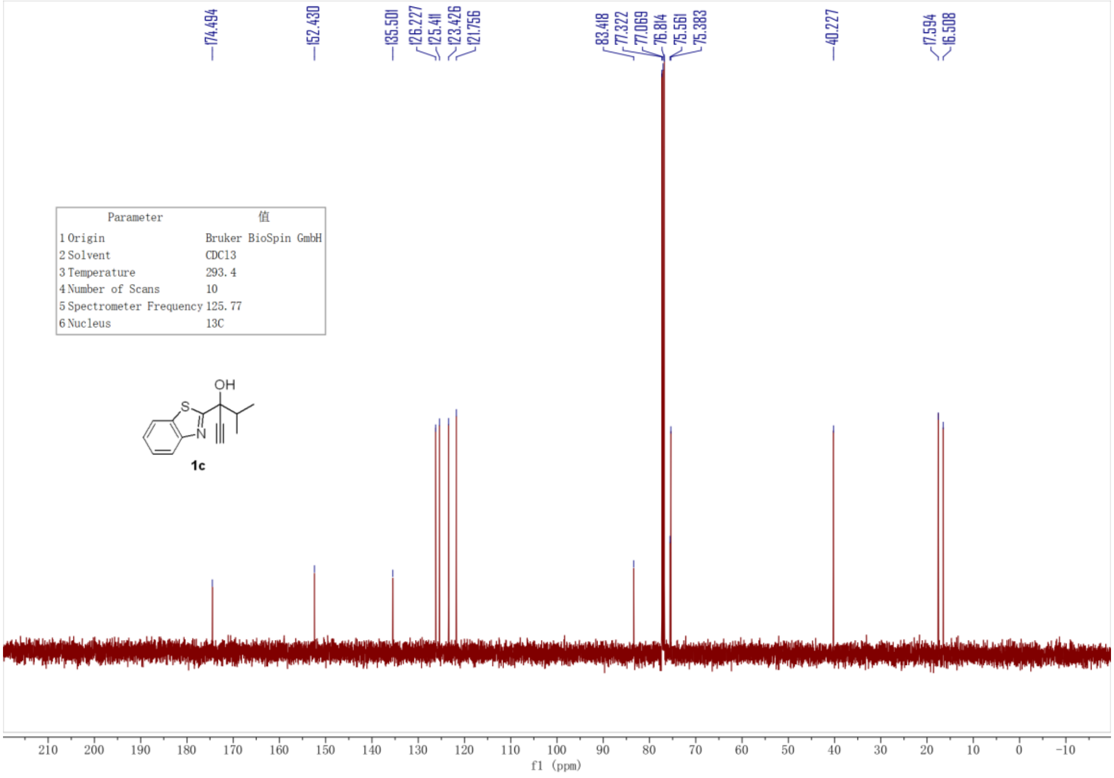
174.494
152.430
135.501
126.227
125.411
123.426
121.756
83.418
77.322
77.069
76.814
75.561
75.383
40.227
17.594
16.508
Parameter 值
1 Origin Bruker BioSpin GmbH
2 Solvent CDCl3
3 Temperature 293.4
4 Number of Scans 10
5 Spectrometer Frequency 125.77
6 Nucleus 13C
OH
S
N
1c
f1 (ppm)

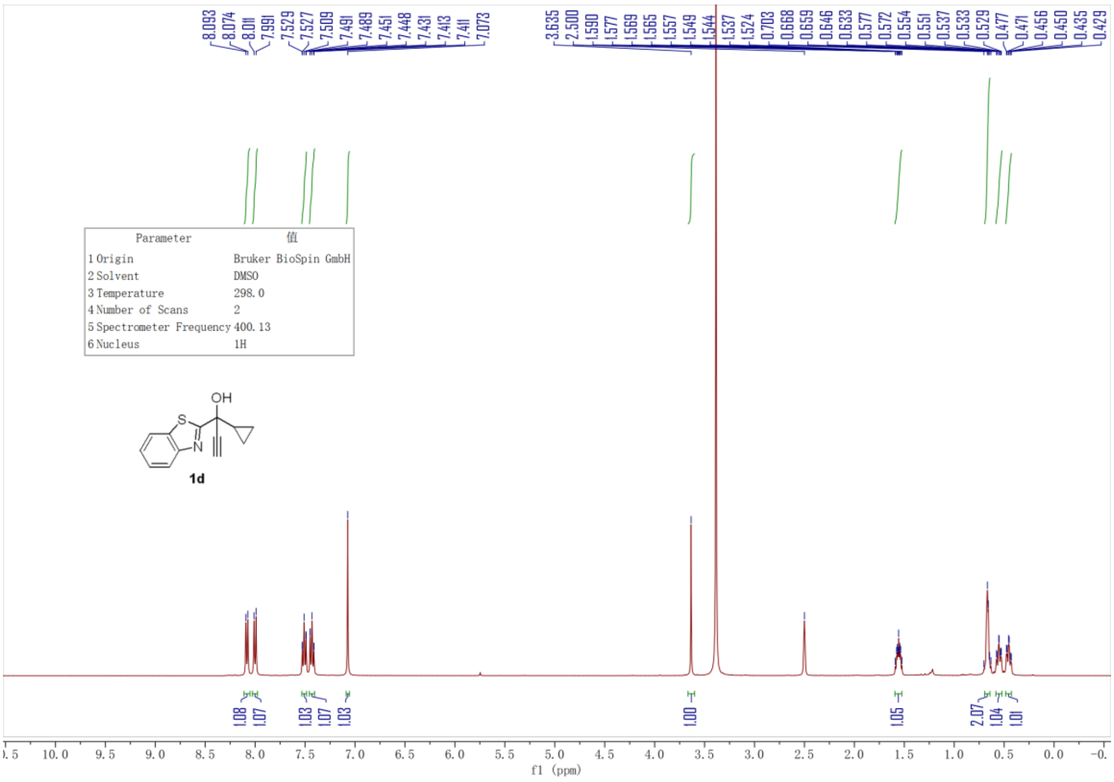
8.093
8.074
8.011
7.991
7.529
7.527
7.509
7.491
7.489
7.451
7.448
7.431
7.413
7.411
7.073
3.635
2.500
1.590
1.577
1.569
1.565
1.557
1.549
1.544
1.537
1.524
0.703
0.668
0.659
0.646
0.633
0.577
0.572
0.554
0.551
0.537
0.533
0.529
0.477
0.471
0.456
0.450
0.435
0.429
Parameter 值
1 Origin Bruker BioSpin GmbH
2 Solvent DMSO
3 Temperature 298.0
4 Number of Scans 2
5 Spectrometer Frequency 400.13
6 Nucleus 1H
OH
S
N
1d
1.08
1.07
1.03
1.07
1.03
1.00
1.05
2.07
1.04
1.01
f1 (ppm)

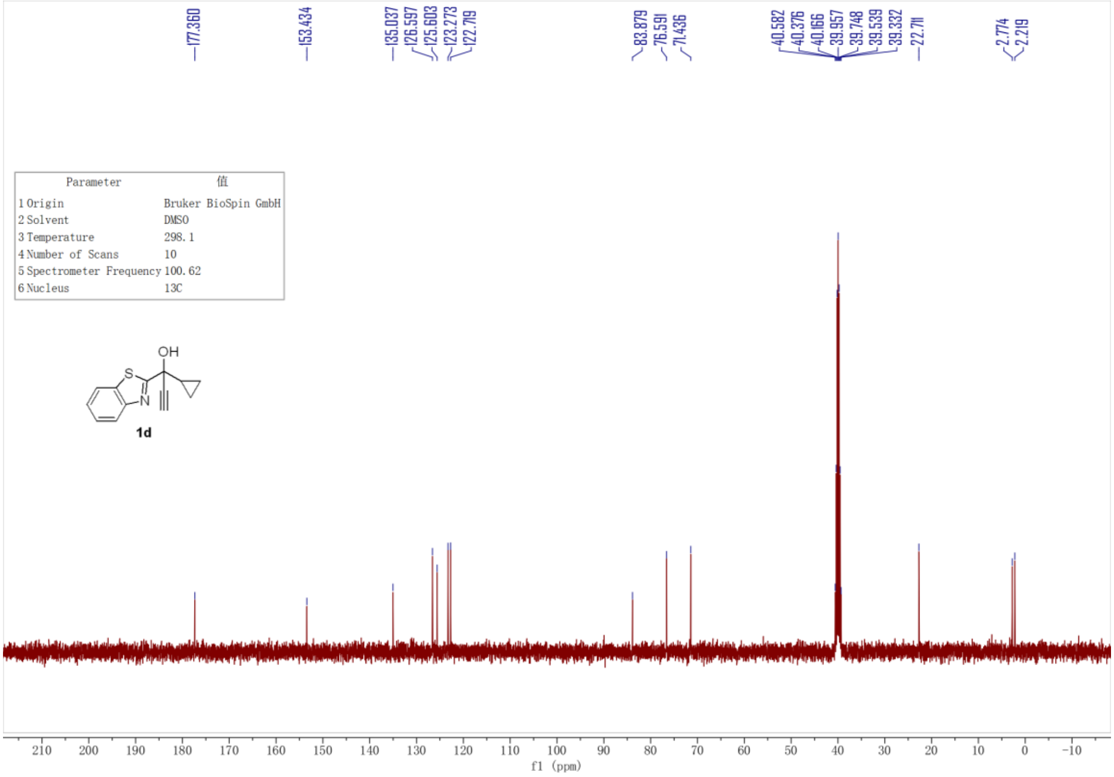
177.360
153.434
135.037
126.597
125.603
123.273
122.719
83.879
76.591
71.436
40.582
40.376
40.166
39.957
39.748
39.539
39.332
22.711
2.774
2.219
Parameter 值
1 Origin Bruker BioSpin GmbH
2 Solvent DMSO
3 Temperature 298.1
4 Number of Scans 10
5 Spectrometer Frequency 100.62
6 Nucleus 13C
OH
S
N
1d
f1 (ppm)

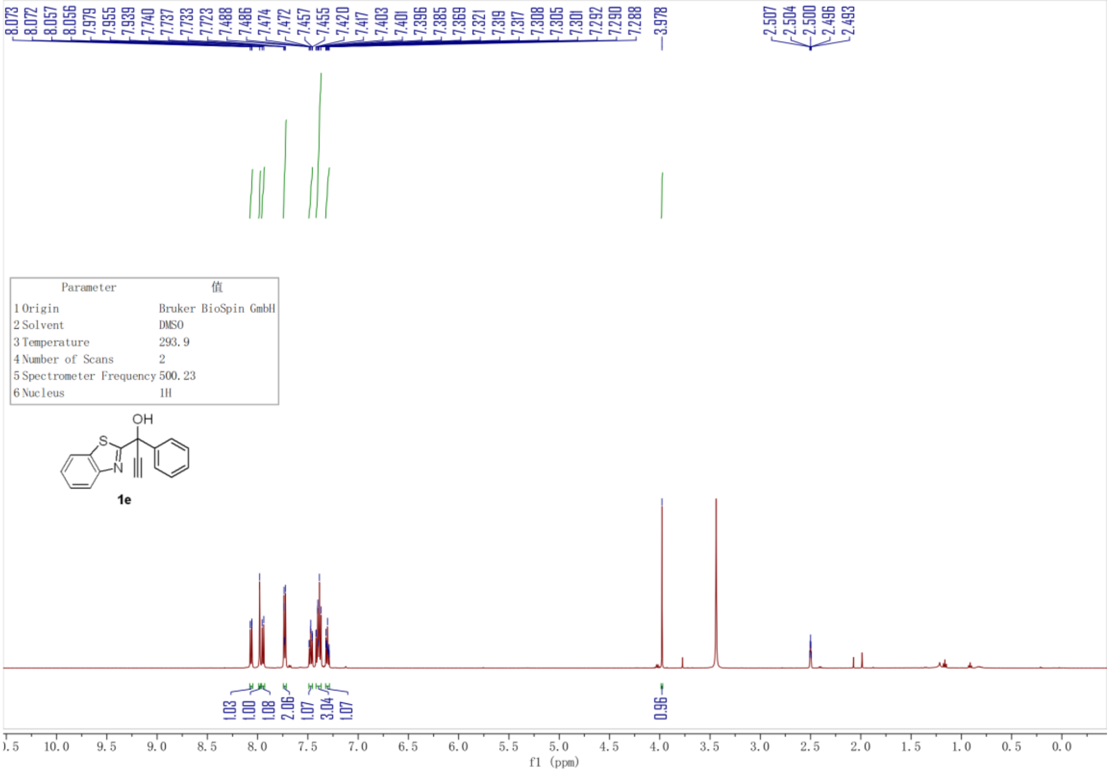
8.073
8.072
8.057
8.056
7.979
7.955
7.939
7.740
7.737
7.733
7.723
7.488
7.486
7.474
7.472
7.457
7.455
7.420
7.417
7.403
7.401
7.396
7.385
7.369
7.321
7.319
7.317
7.308
7.305
7.301
7.292
7.290
7.288
3.978
2.507
2.504
2.500
2.496
2.493
Parameter 值
1 Origin Bruker BioSpin GmbH
2 Solvent DMSO
3 Temperature 293.9
4 Number of Scans 2
5 Spectrometer Frequency 500.23
6 Nucleus 1H
OH
S
N
1e
1.03
1.00
1.08
2.06
1.07
3.04
1.07
0.96
f1 (ppm)

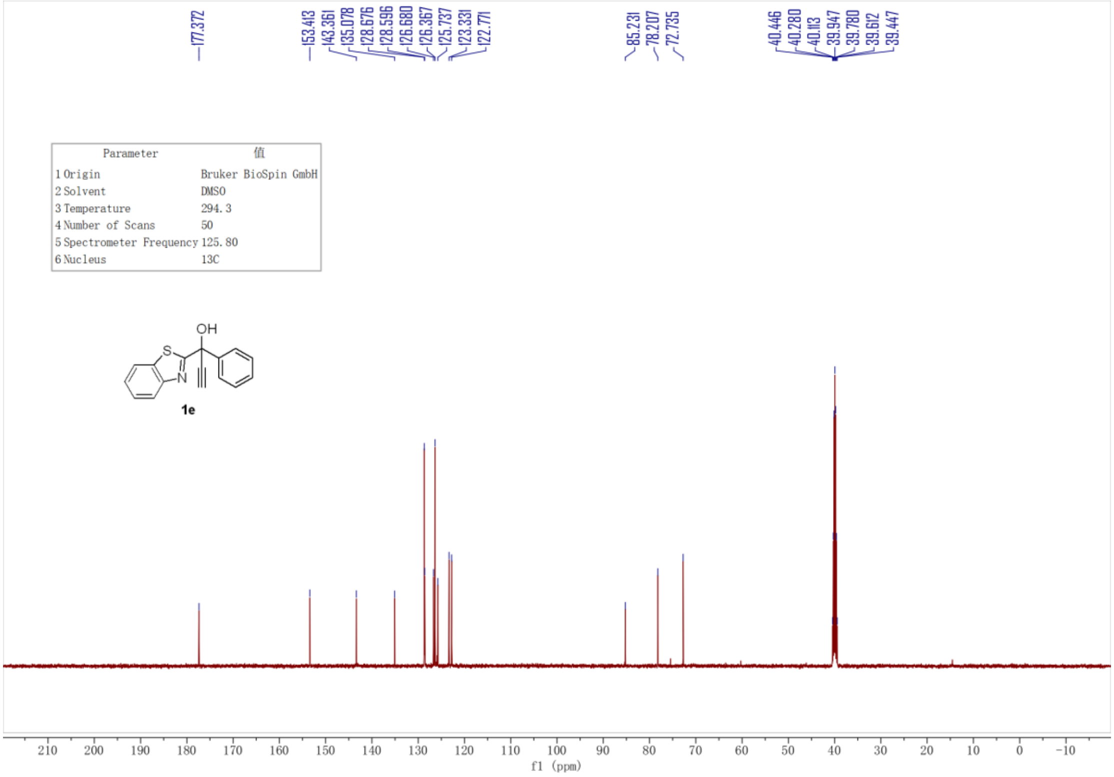
177.372
153.413
143.361
135.078
128.676
128.596
126.680
126.367
125.737
123.331
122.771
85.231
78.207
72.735
40.446
40.280
40.113
39.947
39.780
39.612
39.447
Parameter 值
1 Origin Bruker BioSpin GmbH
2 Solvent DMSO
3 Temperature 294.3
4 Number of Scans 50
5 Spectrometer Frequency 125.80
6 Nucleus 13C
OH
S
N
1e
210 200 190 180 170 160 150 140 130 120 110 100 90 80 70 60 50 40 30 20 10 0 -10
f1 (ppm)

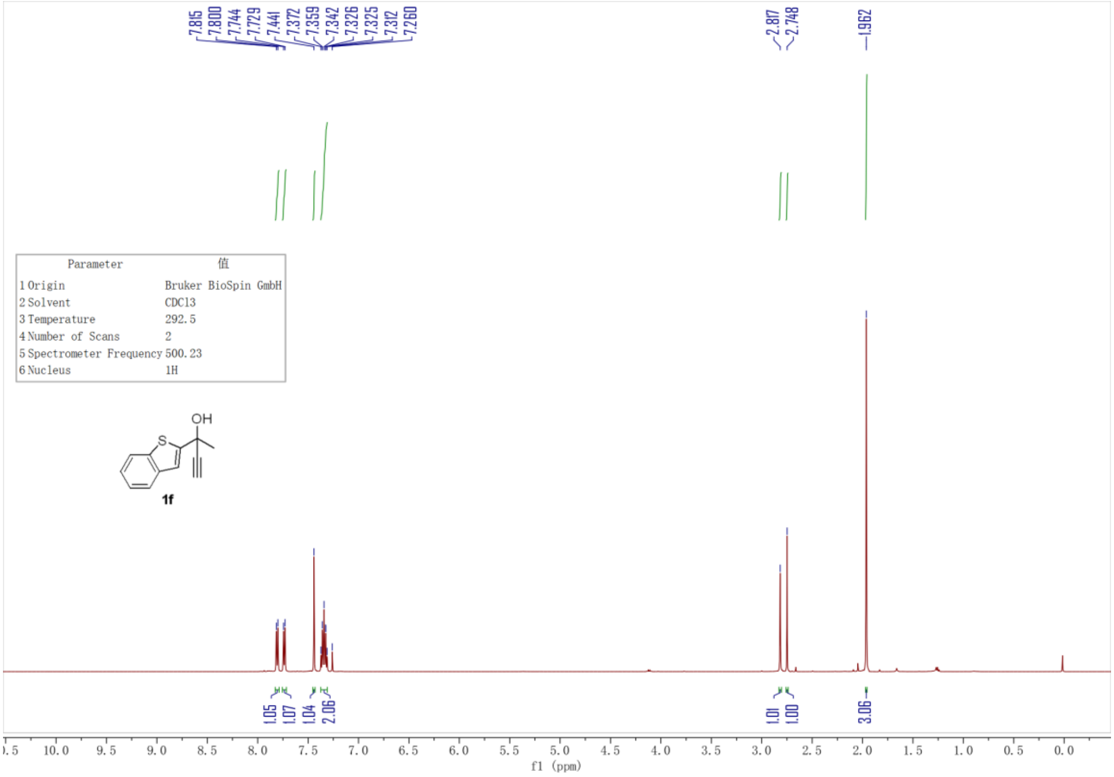
7.815
7.800
7.744
7.729
7.441
7.372
7.359
7.342
7.326
7.325
7.312
7.260
2.817
2.748
1.962
Parameter 值
1 Origin Bruker BioSpin GmbH
2 Solvent CDCl3
3 Temperature 292.5
4 Number of Scans 2
5 Spectrometer Frequency 500.23
6 Nucleus 1H
OH
S
1f
1.05
1.07
1.04
2.06
1.01
1.00
3.06
10.0 9.5 9.0 8.5 8.0 7.5 7.0 6.5 6.0 5.5 5.0 4.5 4.0 3.5 3.0 2.5 2.0 1.5 1.0 0.5 0.0
f1 (ppm)

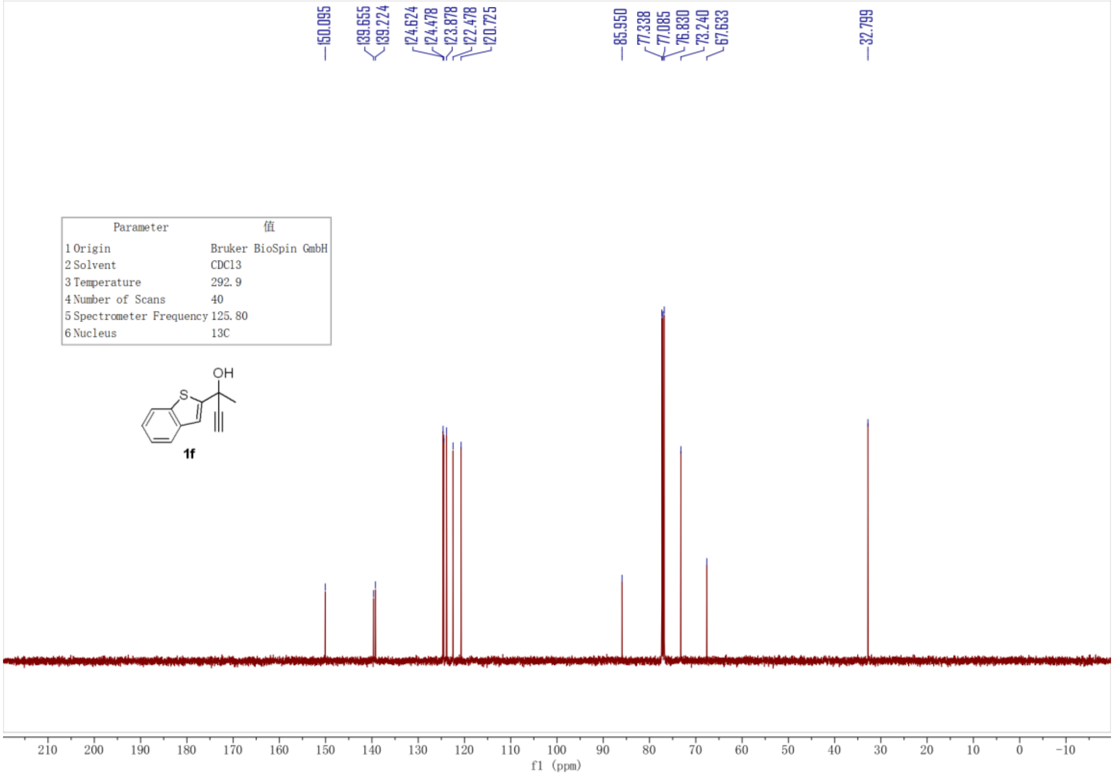
150.095
139.655
139.224
124.624
124.478
123.878
122.478
120.725
85.950
77.338
77.085
76.830
73.240
67.633
32.799
Parameter 值
1 Origin Bruker BioSpin GmbH
2 Solvent CDCl3
3 Temperature 292.9
4 Number of Scans 40
5 Spectrometer Frequency 125.80
6 Nucleus 13C
OH
S
1f
210 200 190 180 170 160 150 140 130 120 110 100 90 80 70 60 50 40 30 20 10 0 -10
f1 (ppm)

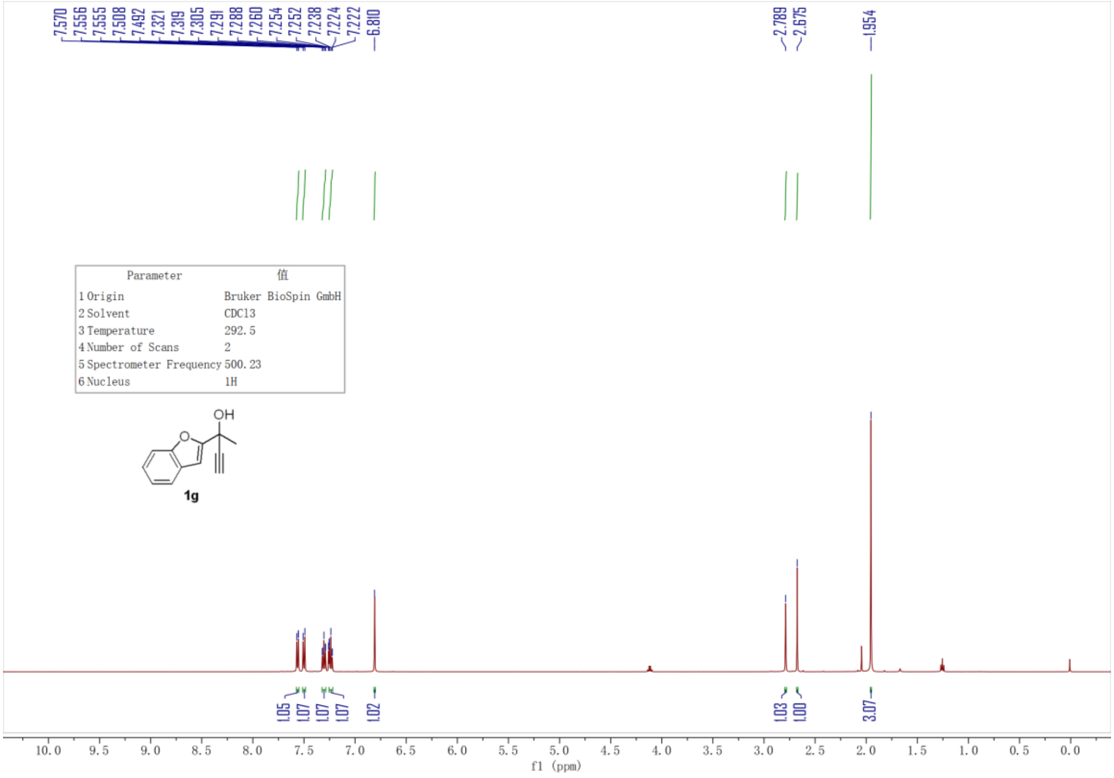
7.570
7.556
7.555
7.508
7.492
7.321
7.319
7.305
7.291
7.288
7.260
7.254
7.252
7.238
7.224
7.222
6.810
2.789
2.675
1.954
Parameter 值
1 Origin Bruker BioSpin GmbH
2 Solvent CDCl3
3 Temperature 292.5
4 Number of Scans 2
5 Spectrometer Frequency 500.23
6 Nucleus 1H
OH
O
1g
1.05
1.07
1.07
1.07
1.02
1.03
1.00
3.07
10.0 9.5 9.0 8.5 8.0 7.5 7.0 6.5 6.0 5.5 5.0 4.5 4.0 3.5 3.0 2.5 2.0 1.5 1.0 0.5 0.0
f1 (ppm)

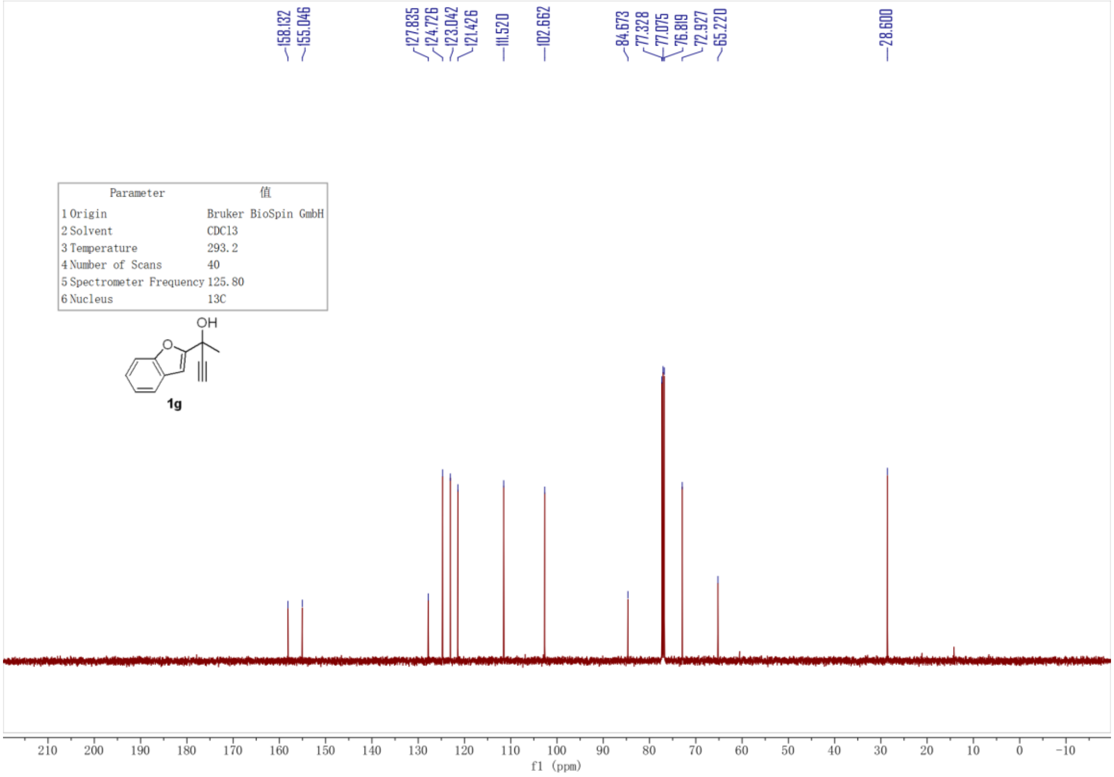
158.132
155.046
127.835
124.726
123.042
121.426
111.520
102.662
84.673
77.328
77.075
76.819
72.927
65.220
28.600
Parameter 值
1 Origin Bruker BioSpin GmbH
2 Solvent CDCl3
3 Temperature 293.2
4 Number of Scans 40
5 Spectrometer Frequency 125.80
6 Nucleus 13C
OH
O
1g
210 200 190 180 170 160 150 140 130 120 110 100 90 80 70 60 50 40 30 20 10 0 -10
f1 (ppm)

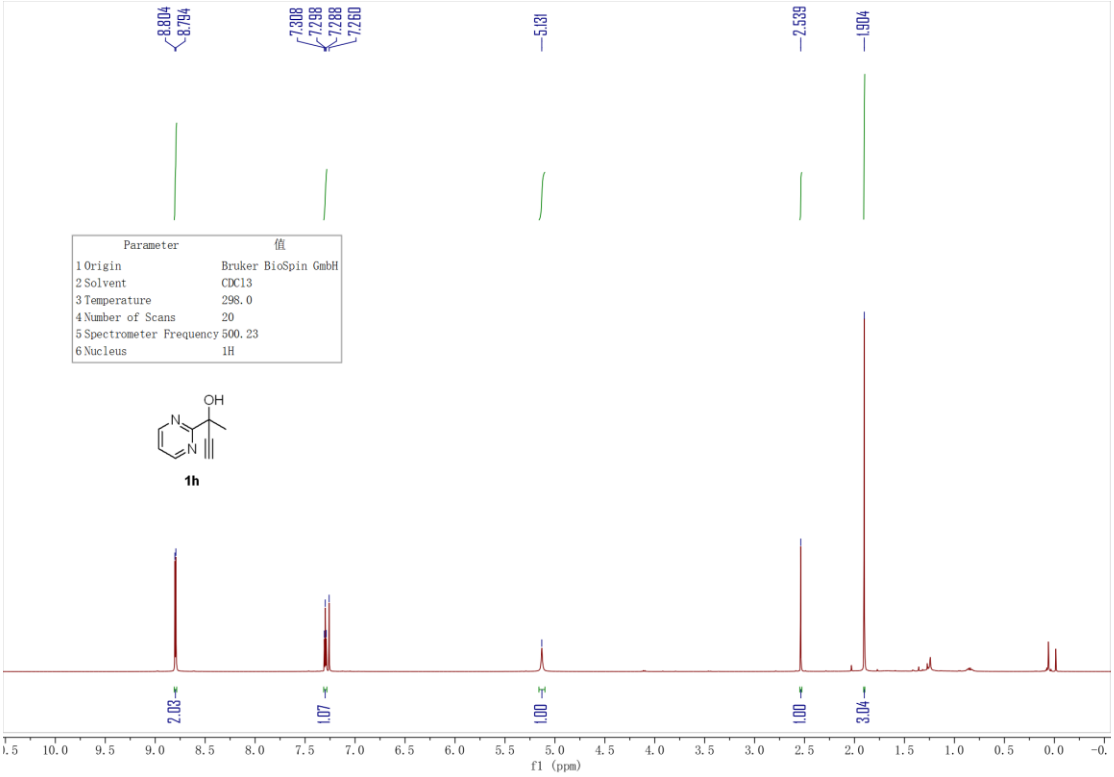
8.804
8.794
7.308
7.298
7.288
7.260
5.131
2.539
1.904
Parameter 值
1 Origin Bruker BioSpin GmbH
2 Solvent CDCl3
3 Temperature 298.0
4 Number of Scans 20
5 Spectrometer Frequency 500.23
6 Nucleus 1H
OH
N
N
1h
2.03
1.07
1.00
1.00
3.04
10.0 9.5 9.0 8.5 8.0 7.5 7.0 6.5 6.0 5.5 5.0 4.5 4.0 3.5 3.0 2.5 2.0 1.5 1.0 0.5 0.0
f1 (ppm)

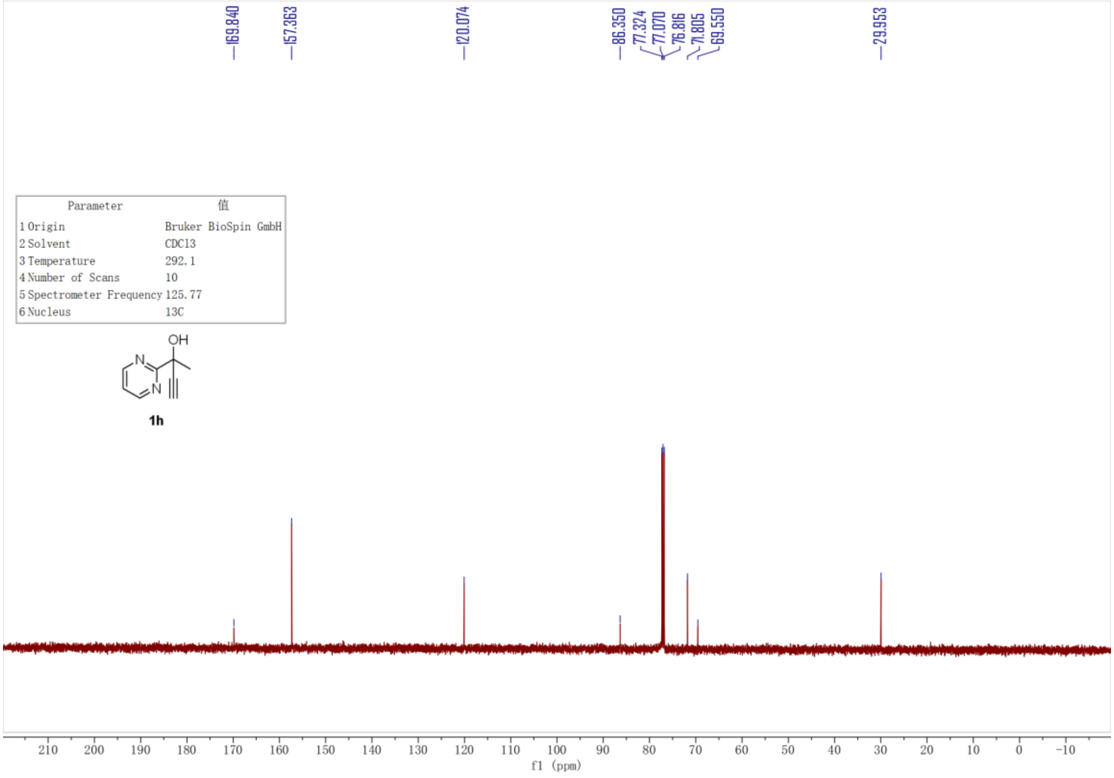

169.840
157.363
120.074
86.350
77.324
77.070
76.816
71.805
69.550
29.953
Parameter 值
1 Origin Bruker BioSpin GmbH
2 Solvent CDCl3
3 Temperature 292.1
4 Number of Scans 10
5 Spectrometer Frequency 125.77
6 Nucleus 13C
OH
N
N
1h
210 200 190 180 170 160 150 140 130 120 110 100 90 80 70 60 50 40 30 20 10 0 -10
f1 (ppm)

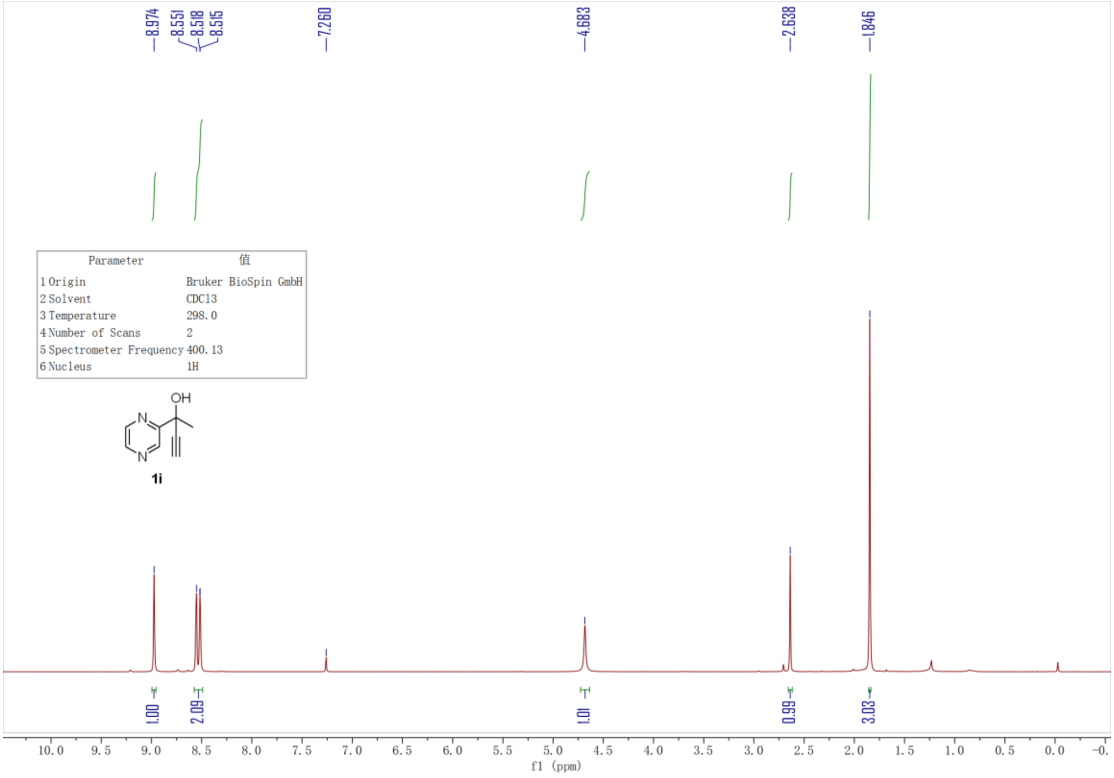

8.974
8.551
8.518
8.515
7.260
4.683
2.638
1.846
Parameter 值
1 Origin Bruker BioSpin GmbH
2 Solvent CDCl3
3 Temperature 298.0
4 Number of Scans 2
5 Spectrometer Frequency 400.13
6 Nucleus 1H
OH
N
N
1i
1.00
2.09
1.01
0.99
3.03
10.0 9.5 9.0 8.5 8.0 7.5 7.0 6.5 6.0 5.5 5.0 4.5 4.0 3.5 3.0 2.5 2.0 1.5 1.0 0.5 0.0
f1 (ppm)

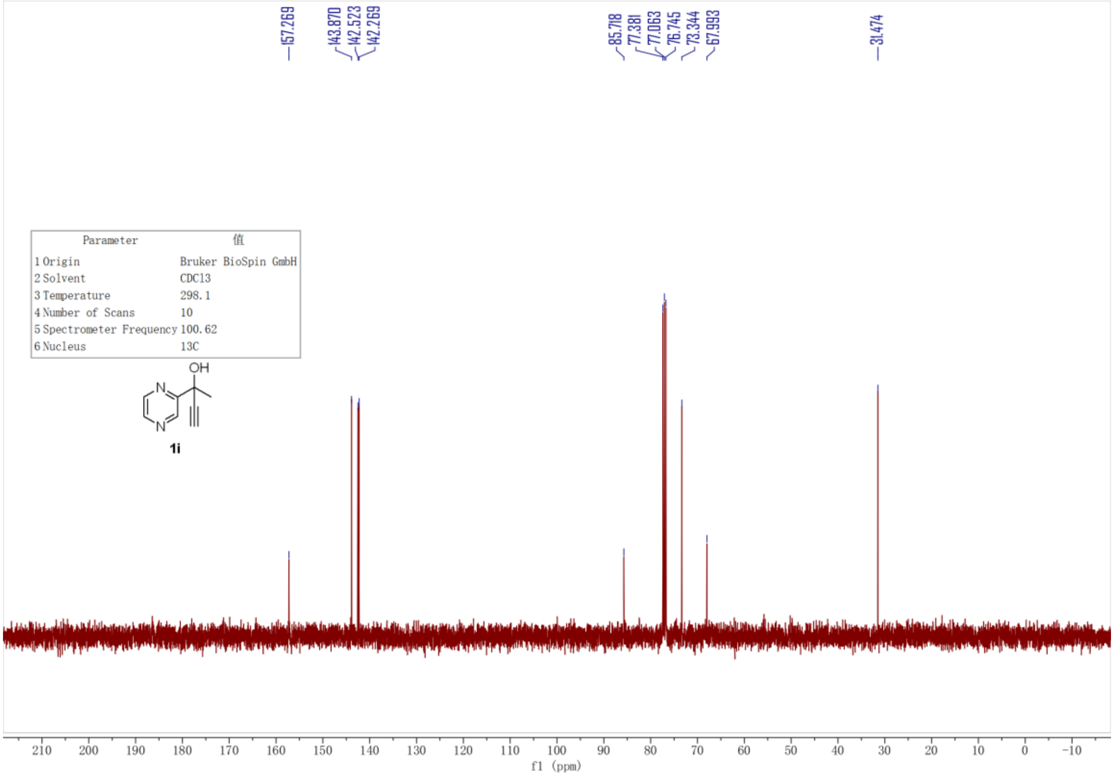
157.269
143.870
142.523
142.269
85.718
77.381
77.063
76.745
73.344
67.993
31.474
Parameter 值
1 Origin Bruker BioSpin GmbH
2 Solvent CDCl3
3 Temperature 298.1
4 Number of Scans 10
5 Spectrometer Frequency 100.62
6 Nucleus 13C
OH
N
N
1i
210 200 190 180 170 160 150 140 130 120 110 100 90 80 70 60 50 40 30 20 10 0 -10
f1 (ppm)

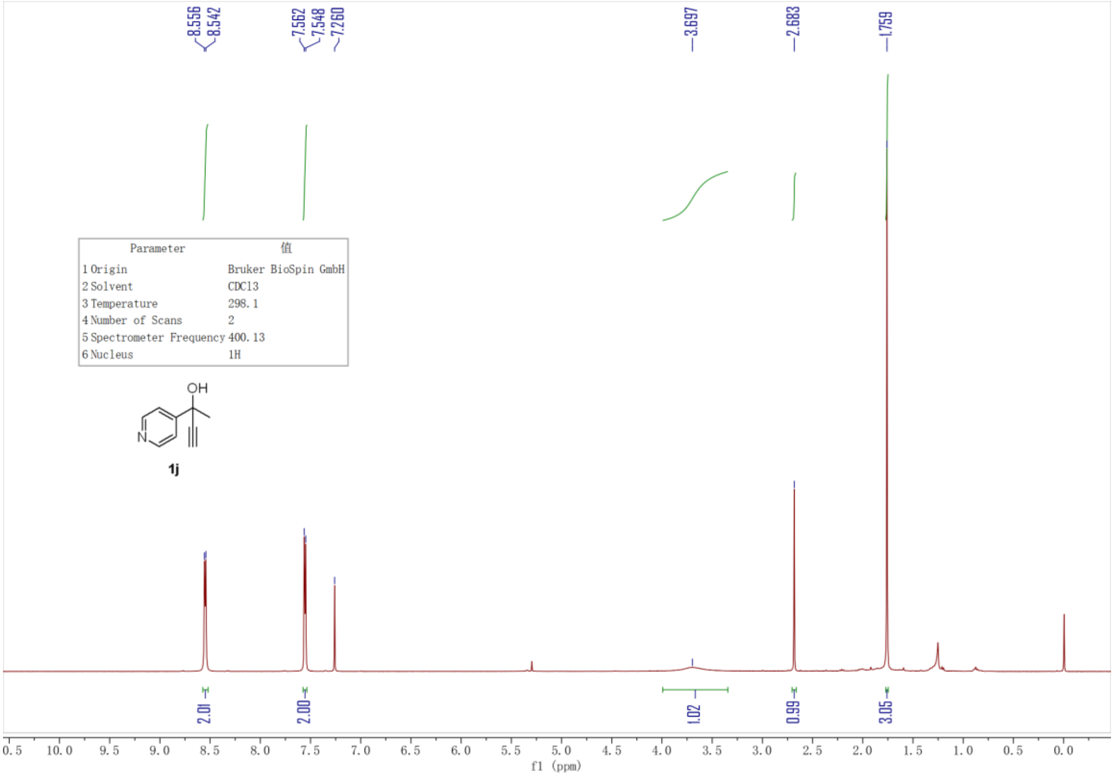
8.556
8.542
7.562
7.548
7.260
3.697
2.683
1.759
Parameter 值
1 Origin Bruker BioSpin GmbH
2 Solvent CDCl3
3 Temperature 298.1
4 Number of Scans 2
5 Spectrometer Frequency 400.13
6 Nucleus 1H
OH
N
1j
2.01
2.00
1.02
0.99
3.05
0.5 10.0 9.5 9.0 8.5 8.0 7.5 7.0 6.5 6.0 5.5 5.0 4.5 4.0 3.5 3.0 2.5 2.0 1.5 1.0 0.5 0.0
f1 (ppm)

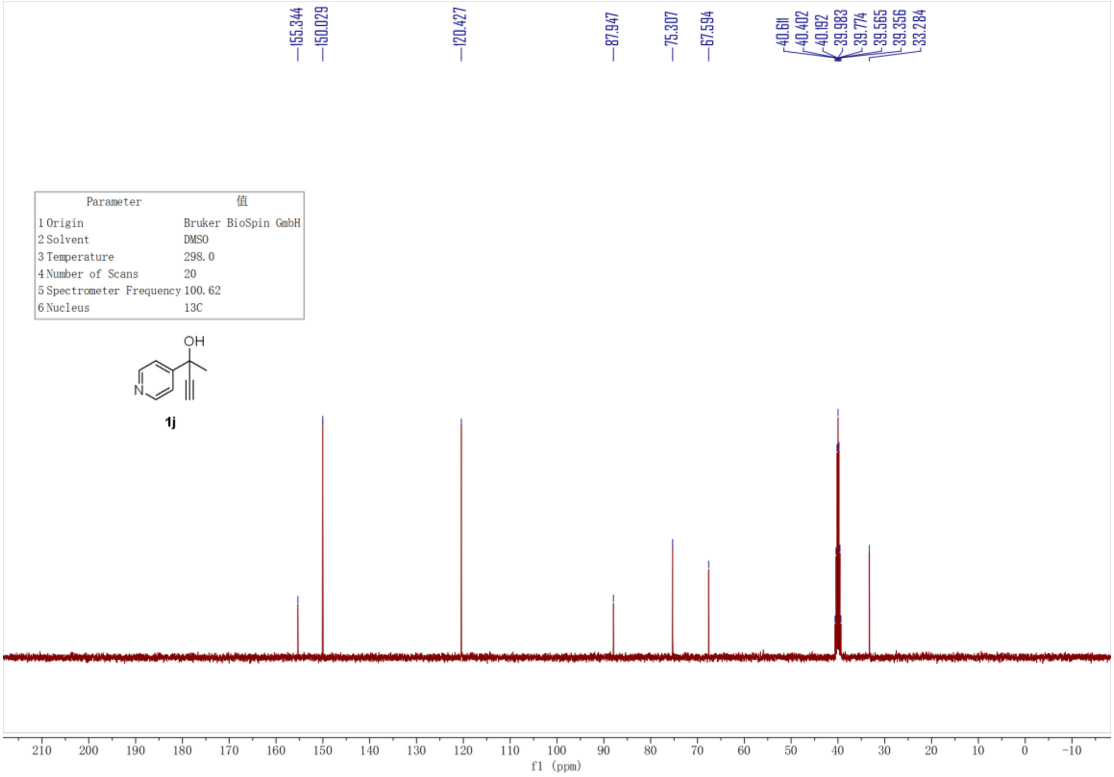

155.344
150.029
120.427
87.947
75.307
67.594
40.611
40.402
40.192
39.983
39.774
39.565
39.356
33.284
Parameter 值
1 Origin Bruker BioSpin GmbH
2 Solvent DMSO
3 Temperature 298.0
4 Number of Scans 20
5 Spectrometer Frequency 100.62
6 Nucleus 13C
OH
N
1j
210 200 190 180 170 160 150 140 130 120 110 100 90 80 70 60 50 40 30 20 10 0 -10
f1 (ppm)

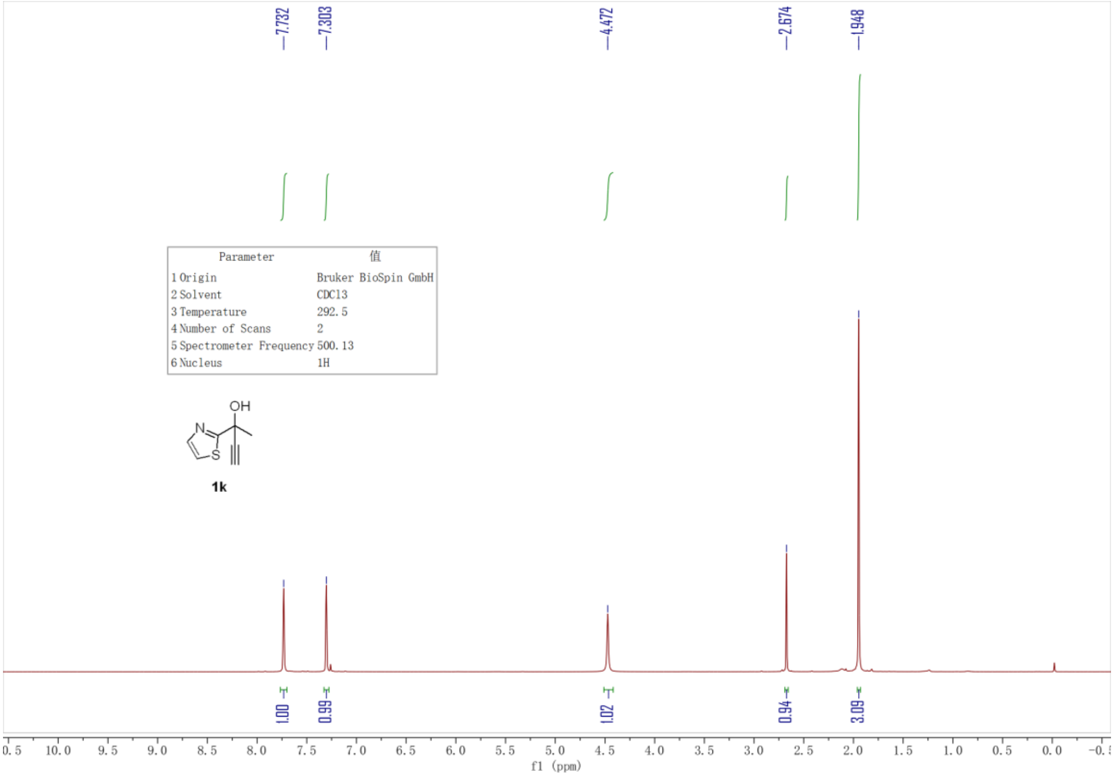

7.732
7.303
4.472
2.674
1.948
Parameter 值
1 Origin Bruker BioSpin GmbH
2 Solvent CDCl3
3 Temperature 292.5
4 Number of Scans 2
5 Spectrometer Frequency 500.13
6 Nucleus 1H
OH
N
S
1k
1.00
0.99
1.02
0.94
3.09
10.0 9.5 9.0 8.5 8.0 7.5 7.0 6.5 6.0 5.5 5.0 4.5 4.0 3.5 3.0 2.5 2.0 1.5 1.0 0.5 0.0
f1 (ppm)

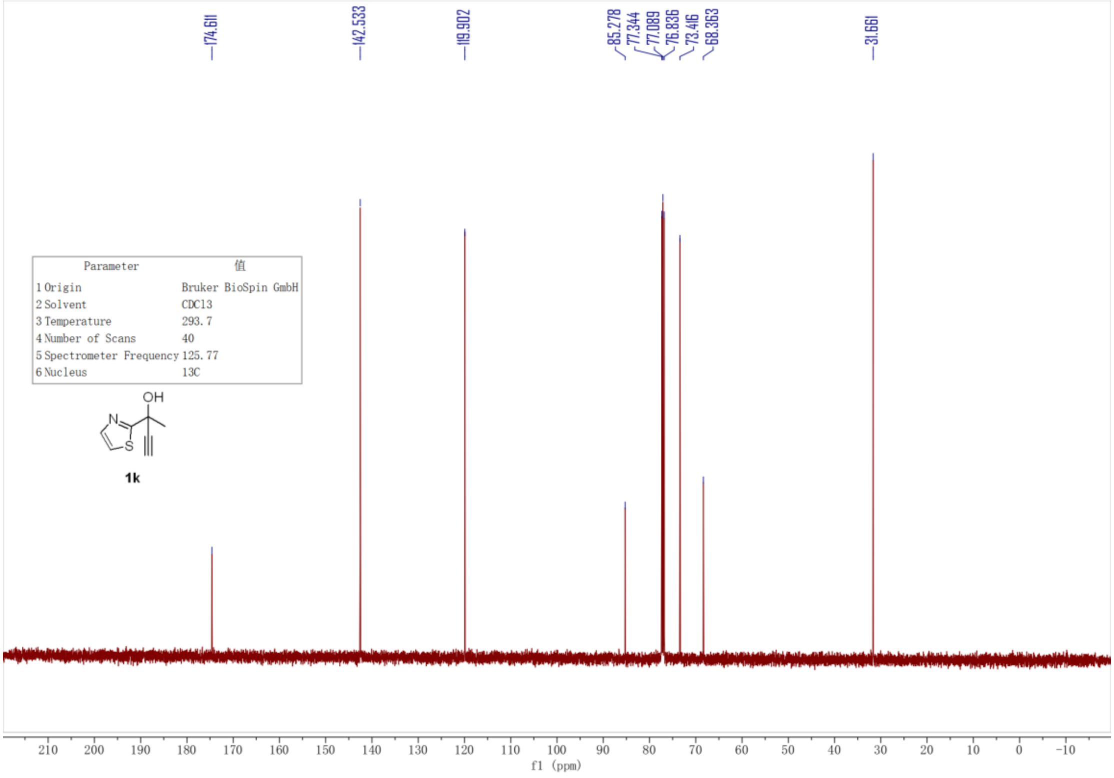
174.611
142.533
119.902
85.278
77.344
77.089
76.836
73.416
68.363
31.661
Parameter 值
1 Origin Bruker BioSpin GmbH
2 Solvent CDCl3
3 Temperature 293.7
4 Number of Scans 40
5 Spectrometer Frequency 125.77
6 Nucleus 13C
OH
N
S
1k
210 200 190 180 170 160 150 140 130 120 110 100 90 80 70 60 50 40 30 20 10 0 -10
f1 (ppm)

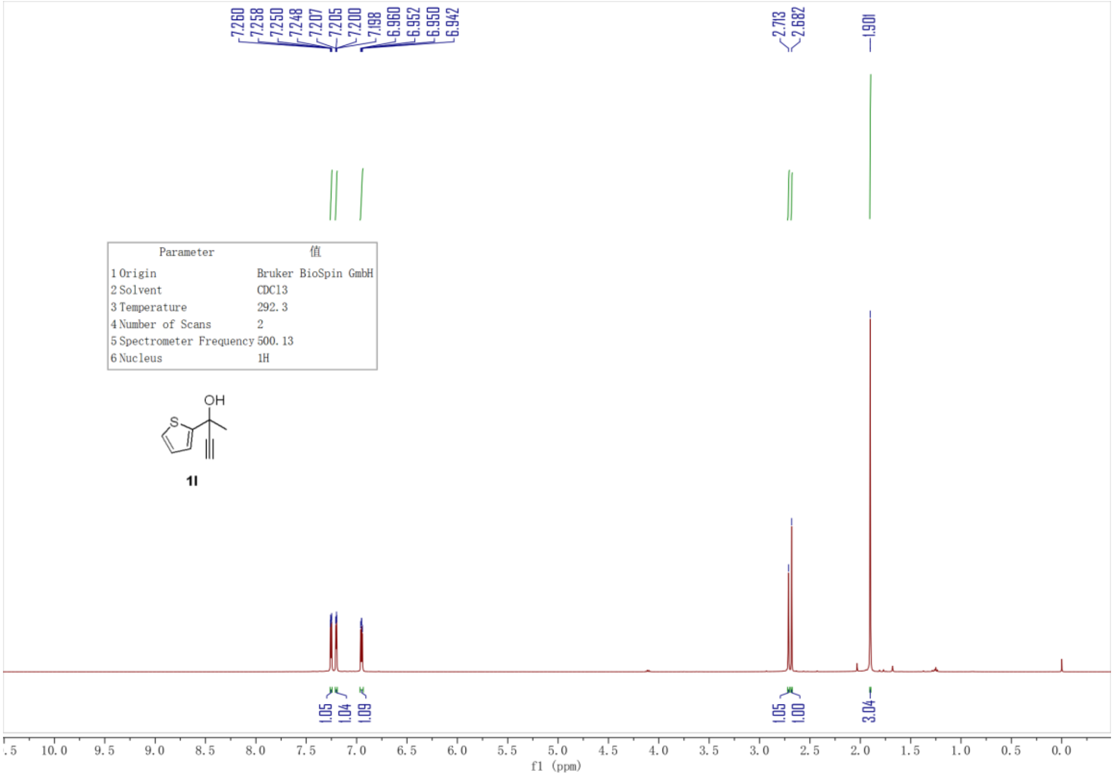
7.260
7.258
7.250
7.248
7.207
7.205
7.200
7.198
6.960
6.952
6.950
6.942
2.713
2.682
1.901
Parameter 值
1 Origin Bruker BioSpin GmbH
2 Solvent CDCl3
3 Temperature 292.3
4 Number of Scans 2
5 Spectrometer Frequency 500.13
6 Nucleus 1H
OH
S
1l
1.05
1.04
1.09
1.05
1.00
3.04
.5 10.0 9.5 9.0 8.5 8.0 7.5 7.0 6.5 6.0 5.5 5.0 4.5 4.0 3.5 3.0 2.5 2.0 1.5 1.0 0.5 0.0
f1 (ppm)

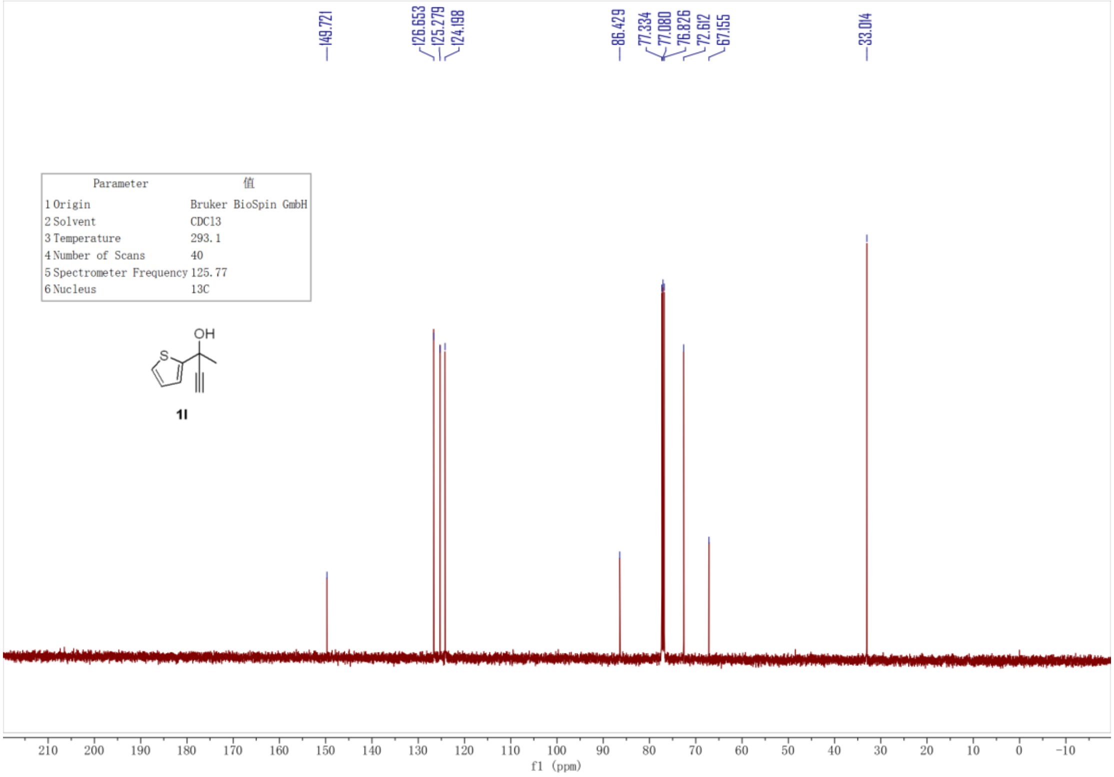
149.721
126.653
125.279
124.198
86.429
77.334
77.080
76.826
72.612
67.155
33.014
Parameter 值
1 Origin Bruker BioSpin GmbH
2 Solvent CDCl3
3 Temperature 293.1
4 Number of Scans 40
5 Spectrometer Frequency 125.77
6 Nucleus 13C
OH
S
1l
210 200 190 180 170 160 150 140 130 120 110 100 90 80 70 60 50 40 30 20 10 0 -10
f1 (ppm)

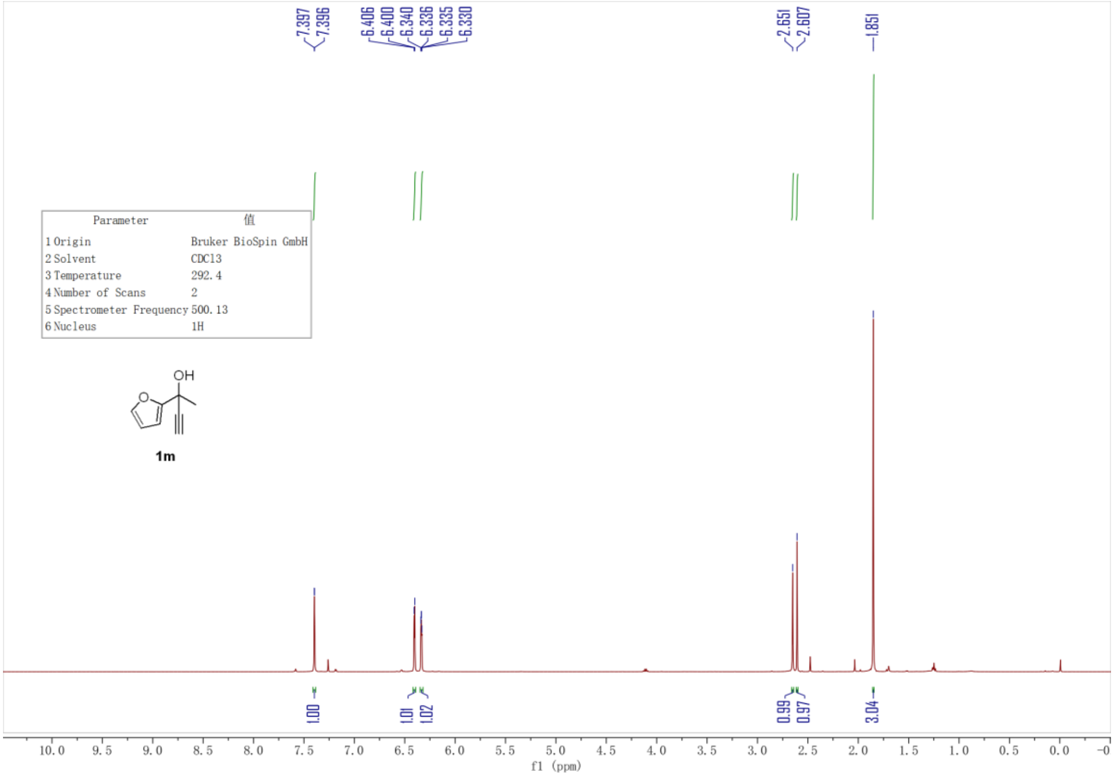
7.397
7.396
6.406
6.400
6.340
6.336
6.335
6.330
2.651
2.607
1.851
Parameter 值
1 Origin Bruker BioSpin GmbH
2 Solvent CDCl3
3 Temperature 292.4
4 Number of Scans 2
5 Spectrometer Frequency 500.13
6 Nucleus 1H
OH
O
1m
1.00
1.01
1.02
0.99
0.97
3.04
10.0 9.5 9.0 8.5 8.0 7.5 7.0 6.5 6.0 5.5 5.0 4.5 4.0 3.5 3.0 2.5 2.0 1.5 1.0 0.5 0.0 -0
f1 (ppm)

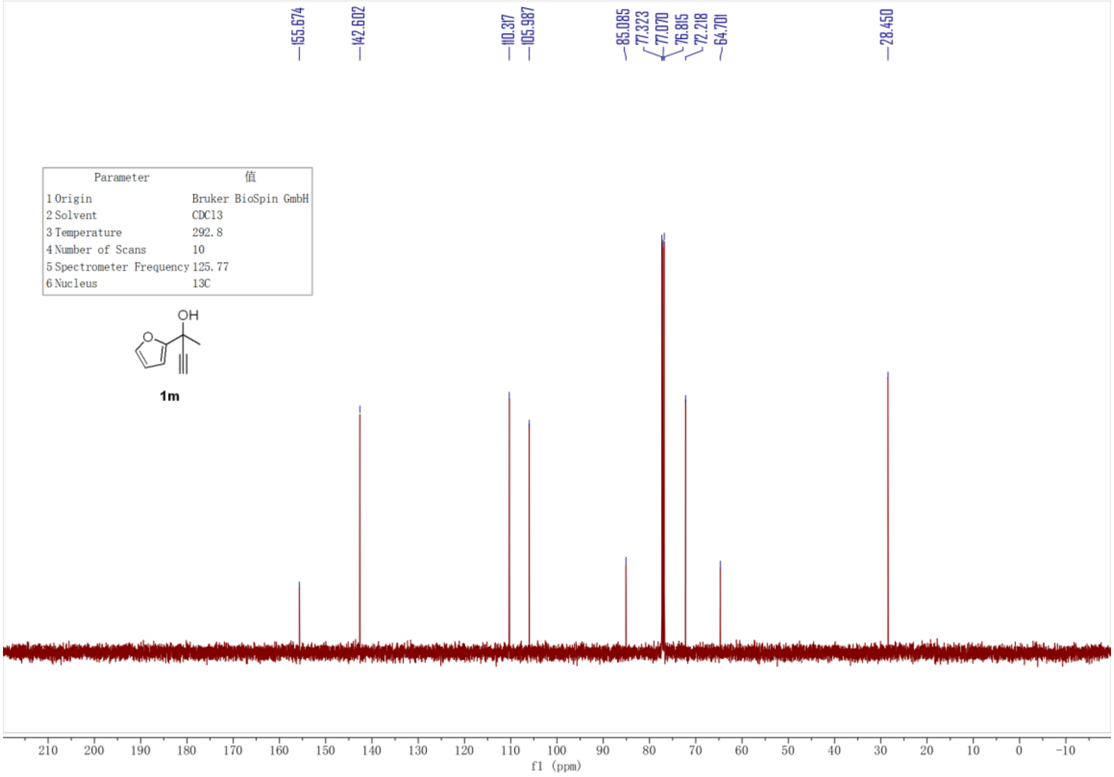
155.674
142.602
110.317
105.987
85.085
77.323
77.070
76.815
72.218
64.701
28.450
Parameter 值
1 Origin Bruker BioSpin GmbH
2 Solvent CDCl3
3 Temperature 292.8
4 Number of Scans 10
5 Spectrometer Frequency 125.77
6 Nucleus 13C
OH
O
1m
f1 (ppm)

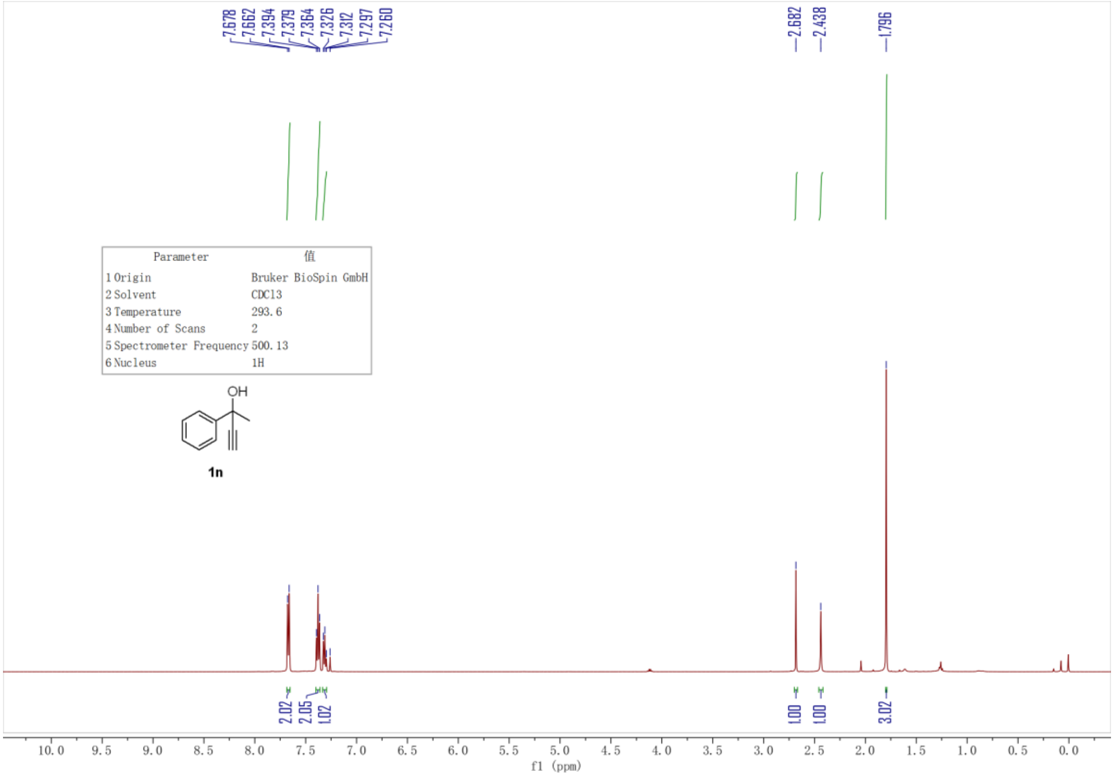
7.678
7.662
7.394
7.379
7.364
7.326
7.312
7.297
7.260
2.682
2.438
1.796
Parameter 值
1 Origin Bruker BioSpin GmbH
2 Solvent CDCl3
3 Temperature 293.6
4 Number of Scans 2
5 Spectrometer Frequency 500.13
6 Nucleus 1H
OH
1n
2.02
2.05
1.02
1.00
1.00
3.02
f1 (ppm)

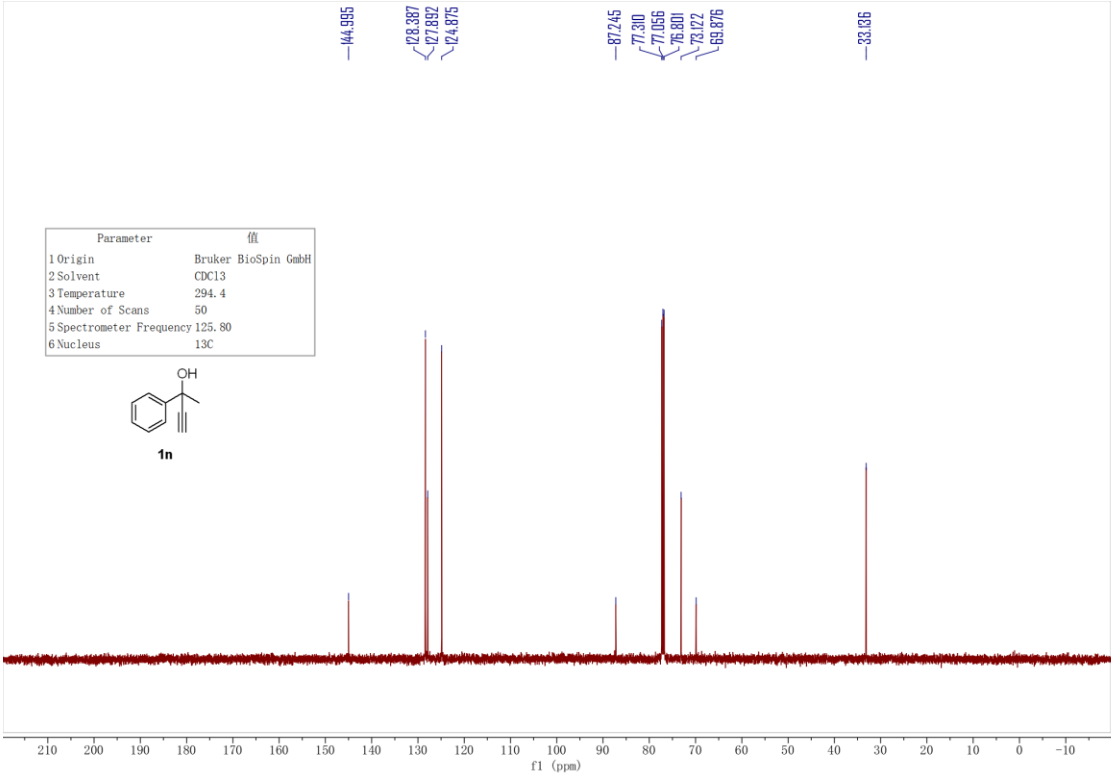
144.995
128.387
127.892
124.875
87.245
77.310
77.056
76.801
73.122
69.876
33.136
Parameter 值
1 Origin Bruker BioSpin GmbH
2 Solvent CDCl3
3 Temperature 294.4
4 Number of Scans 50
5 Spectrometer Frequency 125.80
6 Nucleus 13C
OH
1n
210 200 190 180 170 160 150 140 130 120 110 100 90 80 70 60 50 40 30 20 10 0 -10
f1 (ppm)

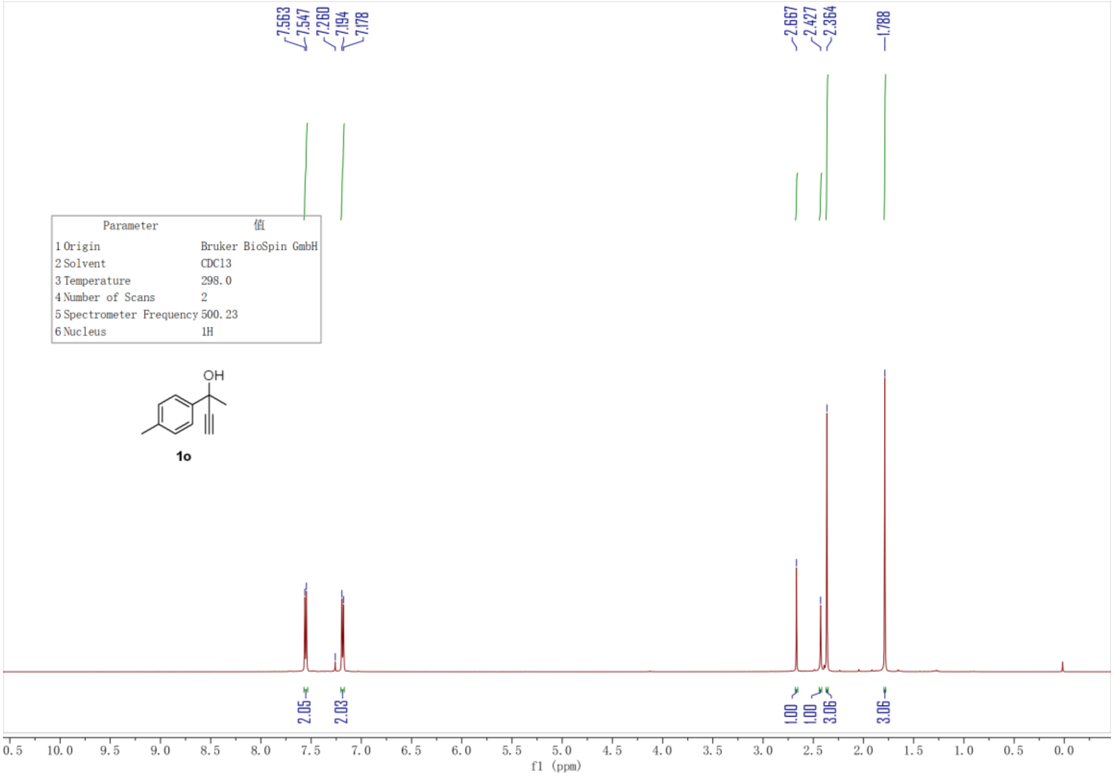
7.563
7.547
7.260
7.194
7.178
2.667
2.427
2.364
1.788
Parameter 值
1 Origin Bruker BioSpin GmbH
2 Solvent CDCl3
3 Temperature 298.0
4 Number of Scans 2
5 Spectrometer Frequency 500.23
6 Nucleus 1H
OH
1o
2.05
2.03
1.00
1.00
3.06
3.06
0.5 10.0 9.5 9.0 8.5 8.0 7.5 7.0 6.5 6.0 5.5 5.0 4.5 4.0 3.5 3.0 2.5 2.0 1.5 1.0 0.5 0.0
f1 (ppm)

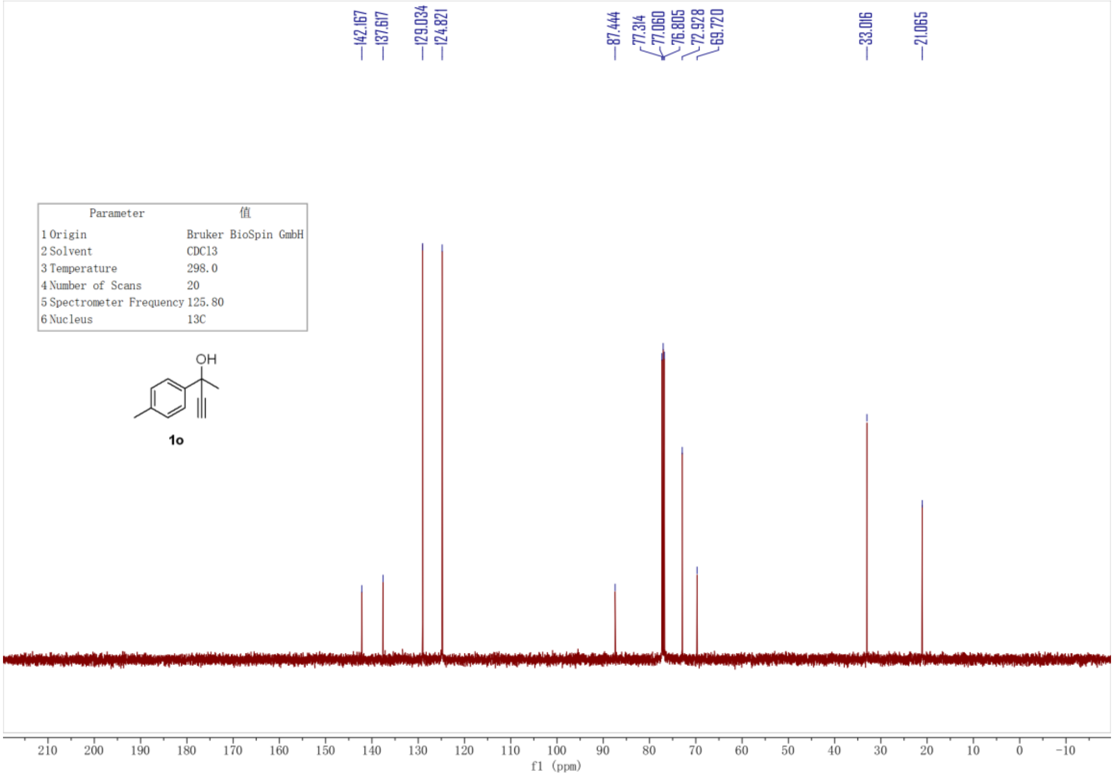
142.167
137.617
129.034
124.821
87.444
77.314
77.060
76.805
72.928
69.720
33.016
21.065
Parameter
值
1 Origin Bruker BioSpin GmbH
2 Solvent CDCl3
3 Temperature 298.0
4 Number of Scans 20
5 Spectrometer Frequency 125.80
6 Nucleus 13C
OH
1o
f1 (ppm)

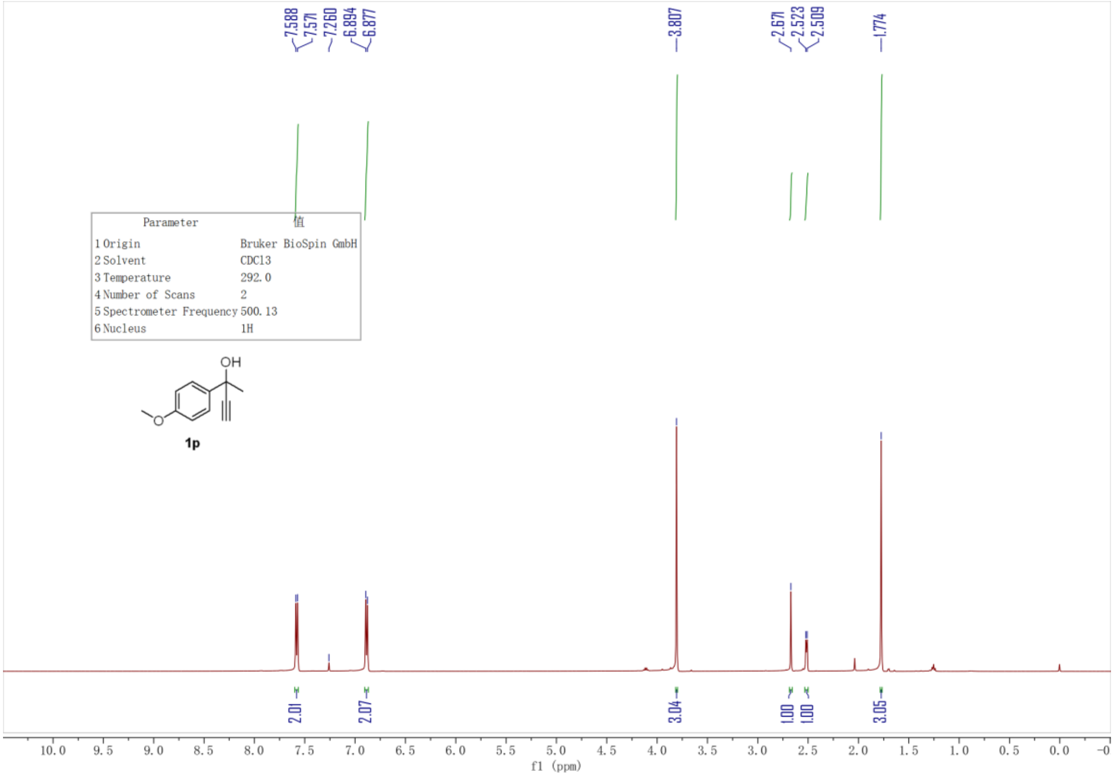
7.588
7.571
7.260
6.894
6.877
3.807
2.671
2.523
2.509
1.774
Parameter
值
1 Origin Bruker BioSpin GmbH
2 Solvent CDCl3
3 Temperature 292.0
4 Number of Scans 2
5 Spectrometer Frequency 500.13
6 Nucleus 1H
OH
O
1p
2.01
2.07
3.04
1.00
1.00
3.05
f1 (ppm)

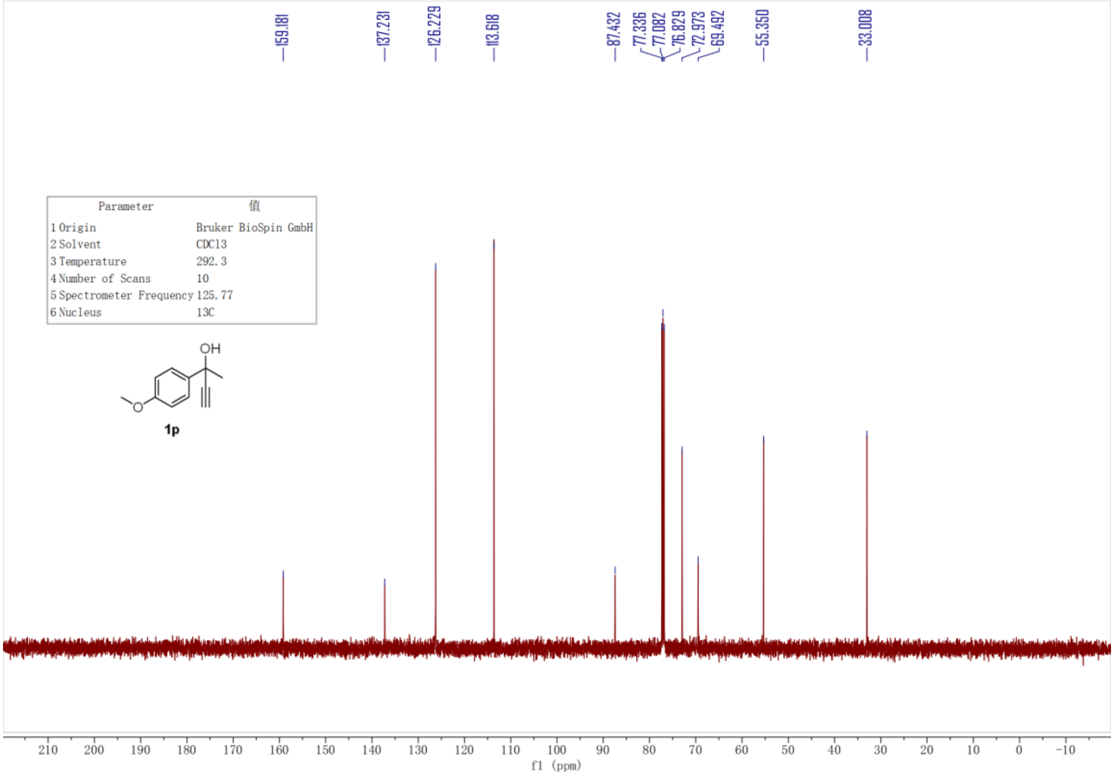

159.181
137.231
126.229
113.618
87.432
77.336
77.082
76.829
72.973
69.492
55.350
33.008
Parameter 值
1 Origin Bruker BioSpin GmbH
2 Solvent CDCl3
3 Temperature 292.3
4 Number of Scans 10
5 Spectrometer Frequency 125.77
6 Nucleus 13C
OH
O
1p
210 200 190 180 170 160 150 140 130 120 110 100 90 80 70 60 50 40 30 20 10 0 -10
f1 (ppm)

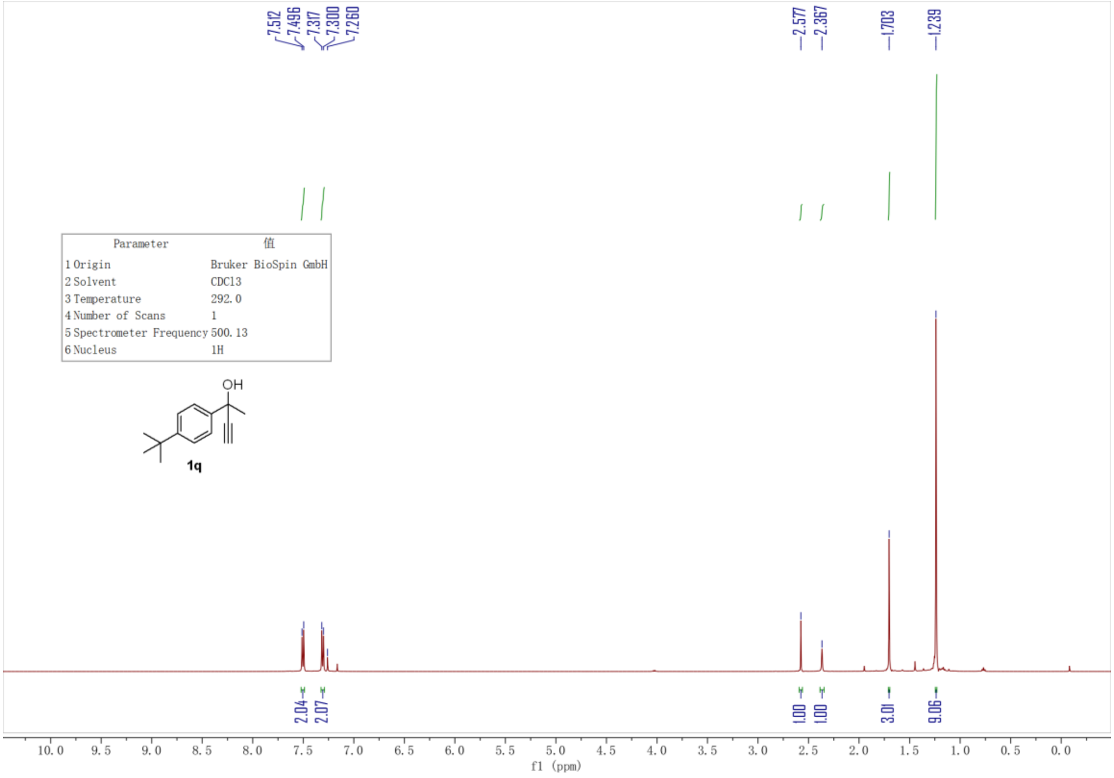

7.512
7.496
7.317
7.300
7.260
2.577
2.367
1.703
1.239
Parameter 值
1 Origin Bruker BioSpin GmbH
2 Solvent CDCl3
3 Temperature 292.0
4 Number of Scans 1
5 Spectrometer Frequency 500.13
6 Nucleus 1H
OH
1q
2.04
2.07
1.00
1.00
3.01
9.06
10.0 9.5 9.0 8.5 8.0 7.5 7.0 6.5 6.0 5.5 5.0 4.5 4.0 3.5 3.0 2.5 2.0 1.5 1.0 0.5 0.0
f1 (ppm)

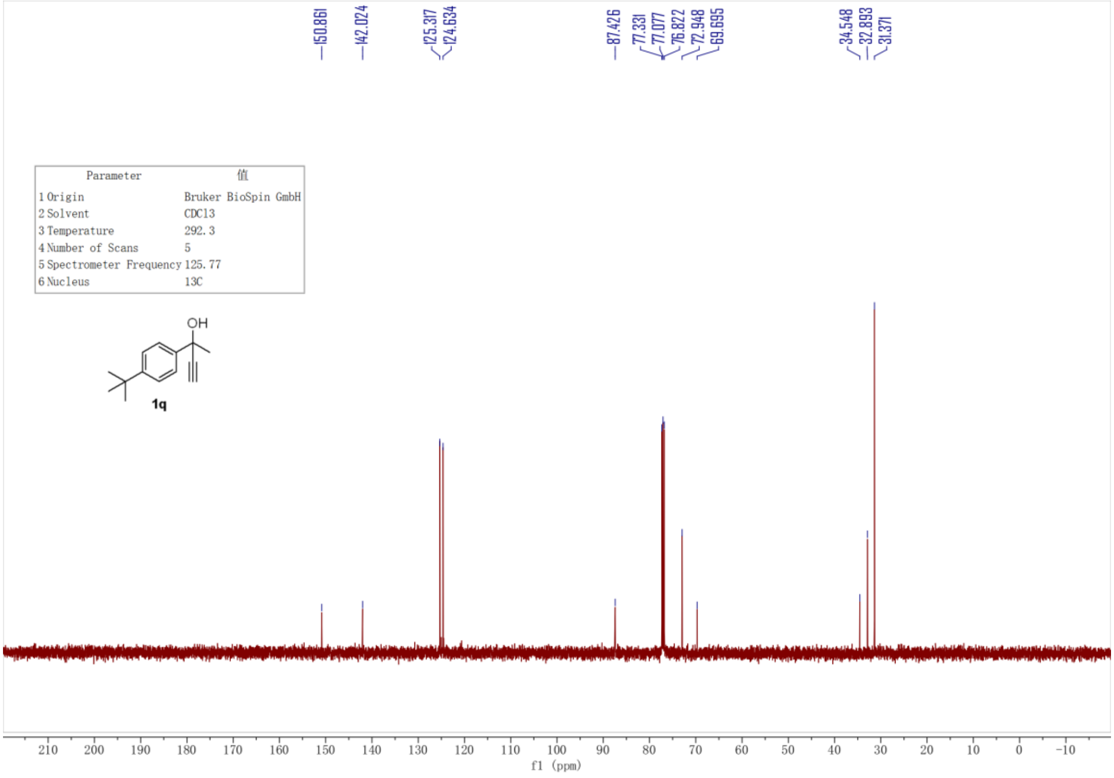

150.861
142.024
125.317
124.634
87.426
77.331
77.077
76.822
72.948
69.695
34.548
32.893
31.371
Parameter 值
1 Origin Bruker BioSpin GmbH
2 Solvent CDCl3
3 Temperature 292.3
4 Number of Scans 5
5 Spectrometer Frequency 125.77
6 Nucleus 13C
OH
1q
210 200 190 180 170 160 150 140 130 120 110 100 90 80 70 60 50 40 30 20 10 0 -10
f1 (ppm)

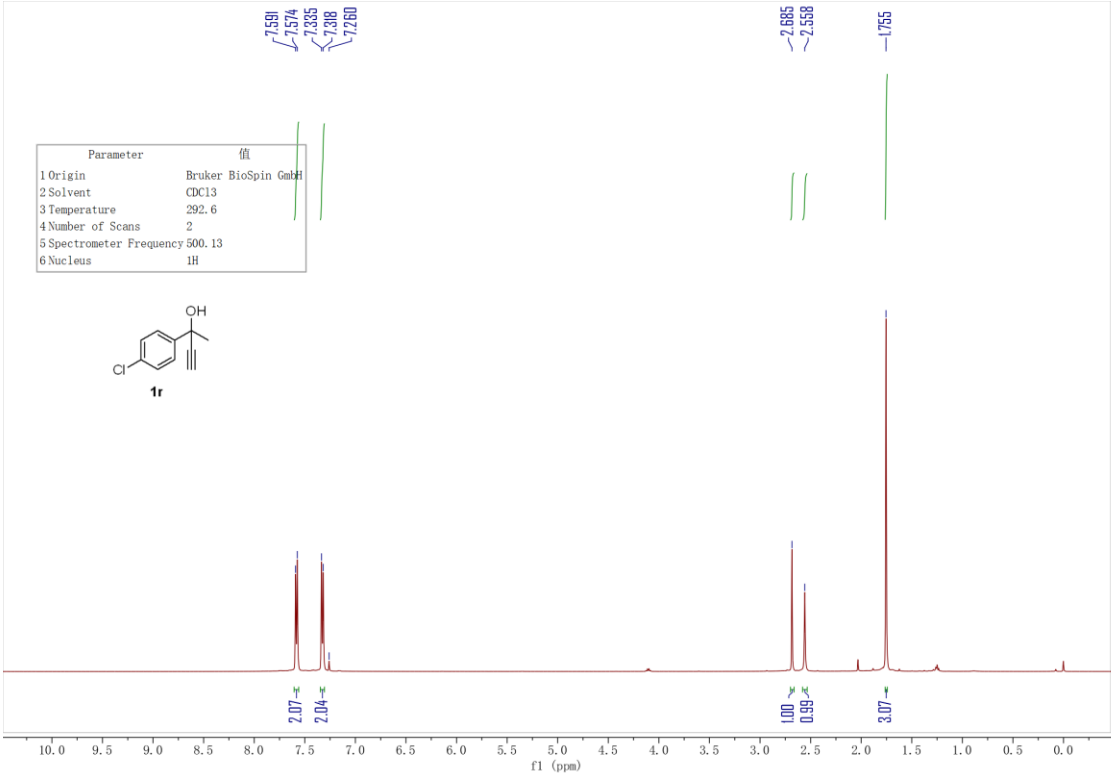

7.591
7.574
7.335
7.318
7.260
2.685
2.558
1.755
Parameter 值
1 Origin Bruker BioSpin GmbH
2 Solvent CDCl3
3 Temperature 292.6
4 Number of Scans 2
5 Spectrometer Frequency 500.13
6 Nucleus 1H
OH
Cl
1r
2.07
2.04
1.00
0.99
3.07
10.0 9.5 9.0 8.5 8.0 7.5 7.0 6.5 6.0 5.5 5.0 4.5 4.0 3.5 3.0 2.5 2.0 1.5 1.0 0.5 0.0
f1 (ppm)

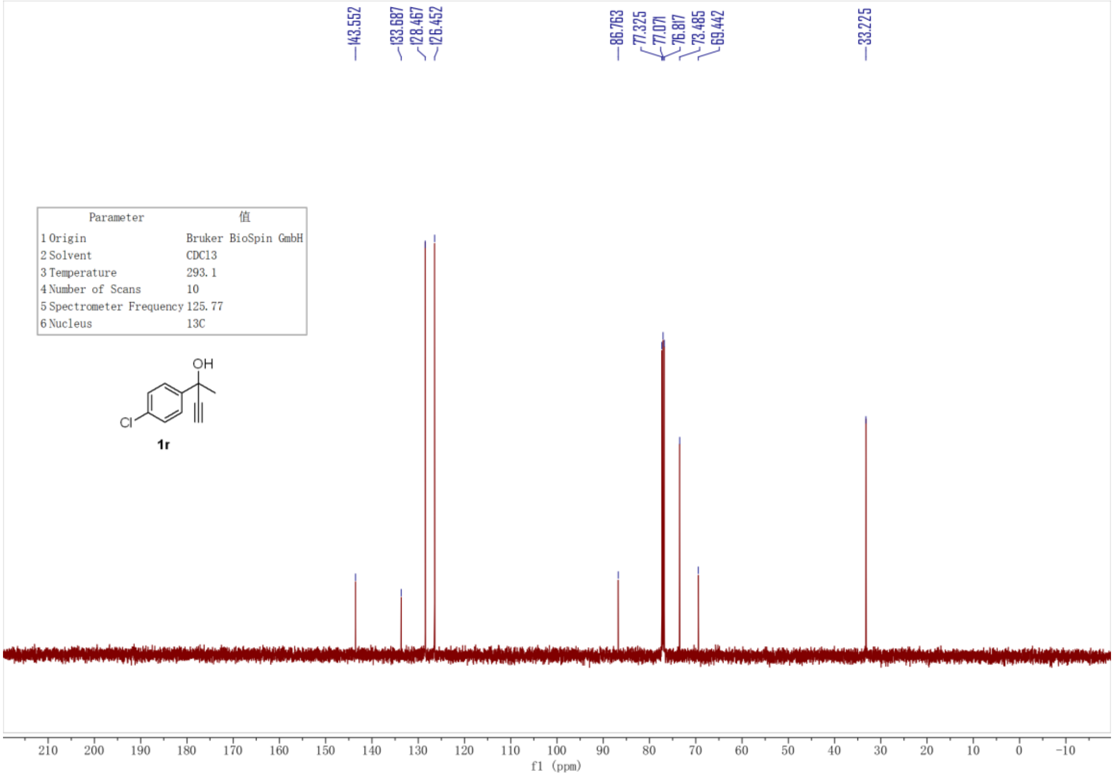
143.552
133.687
128.467
126.452
86.763
77.325
77.071
76.817
73.485
69.442
33.225
Parameter 值
1 Origin Bruker BioSpin GmbH
2 Solvent CDCl3
3 Temperature 293.1
4 Number of Scans 10
5 Spectrometer Frequency 125.77
6 Nucleus 13C
OH
Cl
1r
210 200 190 180 170 160 150 140 130 120 110 100 90 80 70 60 50 40 30 20 10 0 -10
f1 (ppm)

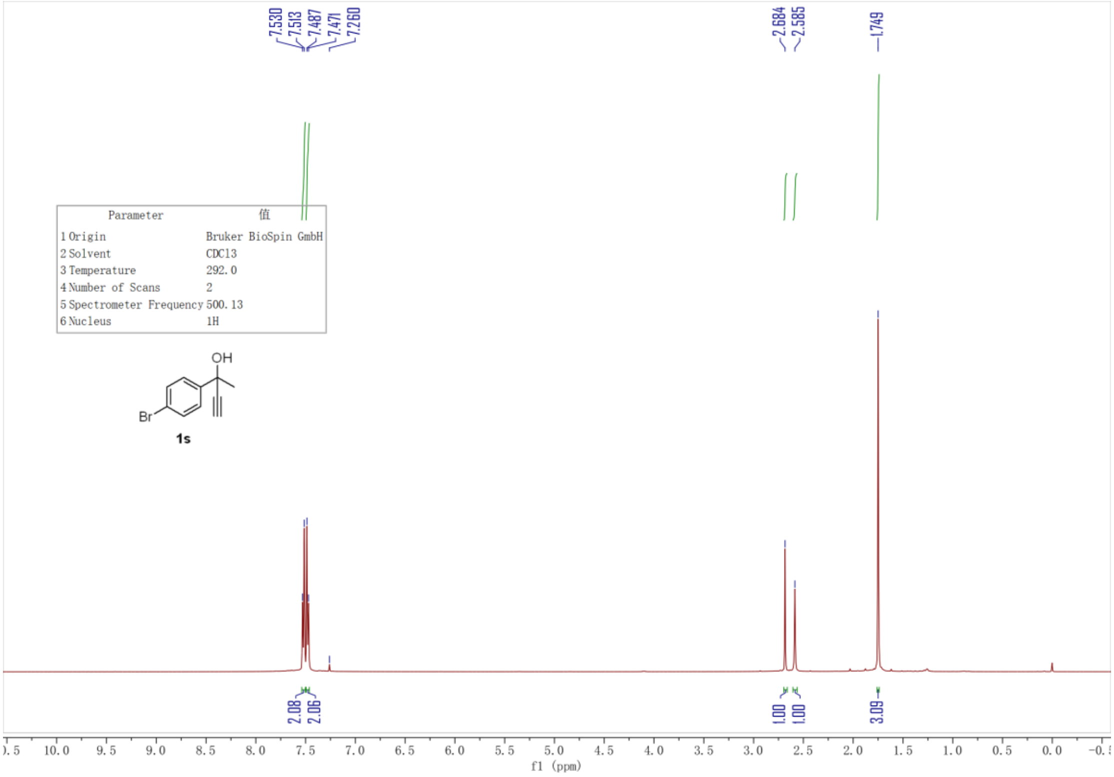
7.530
7.513
7.487
7.471
7.260
2.684
2.585
1.749
Parameter 值
1 Origin Bruker BioSpin GmbH
2 Solvent CDCl3
3 Temperature 292.0
4 Number of Scans 2
5 Spectrometer Frequency 500.13
6 Nucleus 1H
OH
Br
1s
2.08
2.06
1.00
1.00
3.09
10.0 9.5 9.0 8.5 8.0 7.5 7.0 6.5 6.0 5.5 5.0 4.5 4.0 3.5 3.0 2.5 2.0 1.5 1.0 0.5 0.0
f1 (ppm)

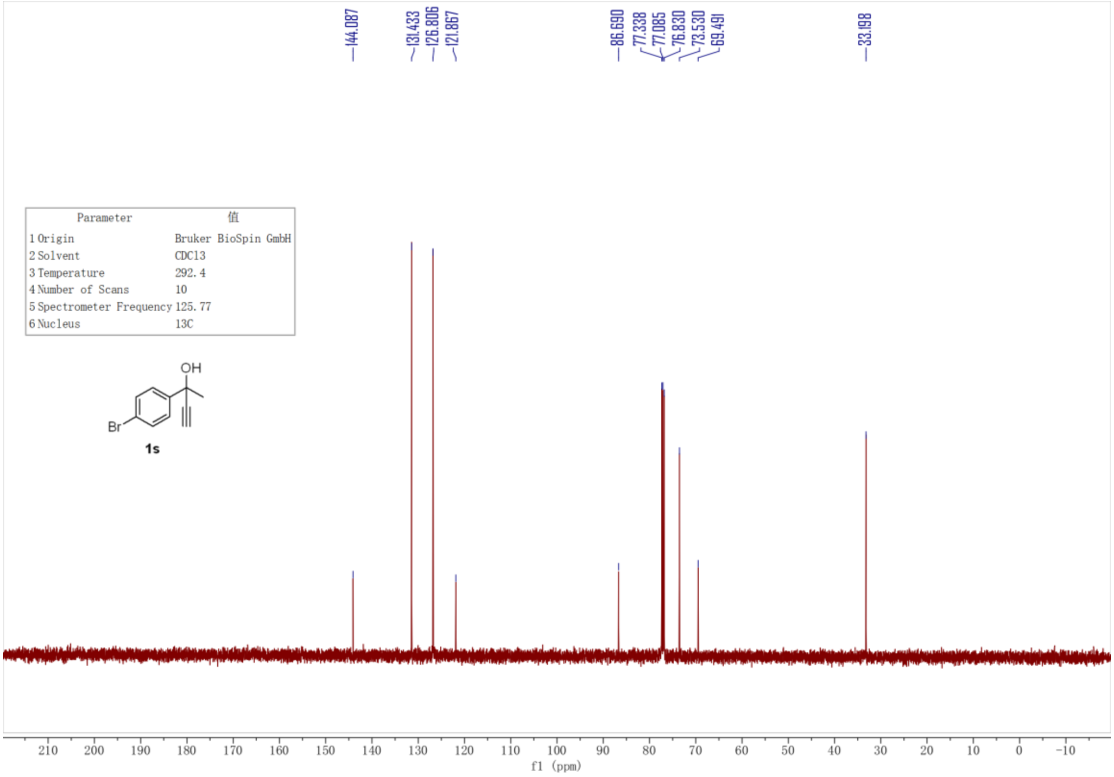
144.087
131.433
126.806
121.867
86.690
77.338
77.085
76.830
73.530
69.491
33.198
Parameter 値
1 Origin Bruker BioSpin GmbH
2 Solvent CDCl3
3 Temperature 292.4
4 Number of Scans 10
5 Spectrometer Frequency 125.77
6 Nucleus 13C
OH
Br
1s
f1 (ppm)

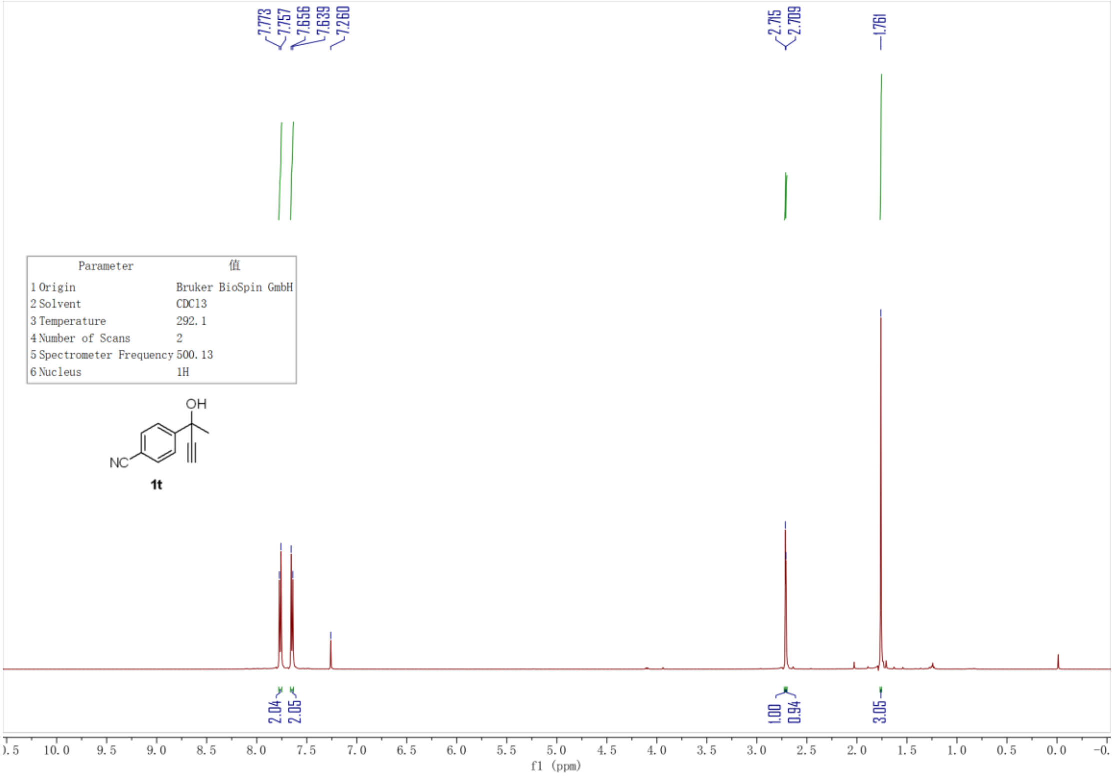
7.773
7.757
7.656
7.639
7.260
2.715
2.709
1.761
Parameter 値
1 Origin Bruker BioSpin GmbH
2 Solvent CDCl3
3 Temperature 292.1
4 Number of Scans 2
5 Spectrometer Frequency 500.13
6 Nucleus 1H
OH
NC
1t
2.04
2.05
1.00
0.94
3.05
f1 (ppm)

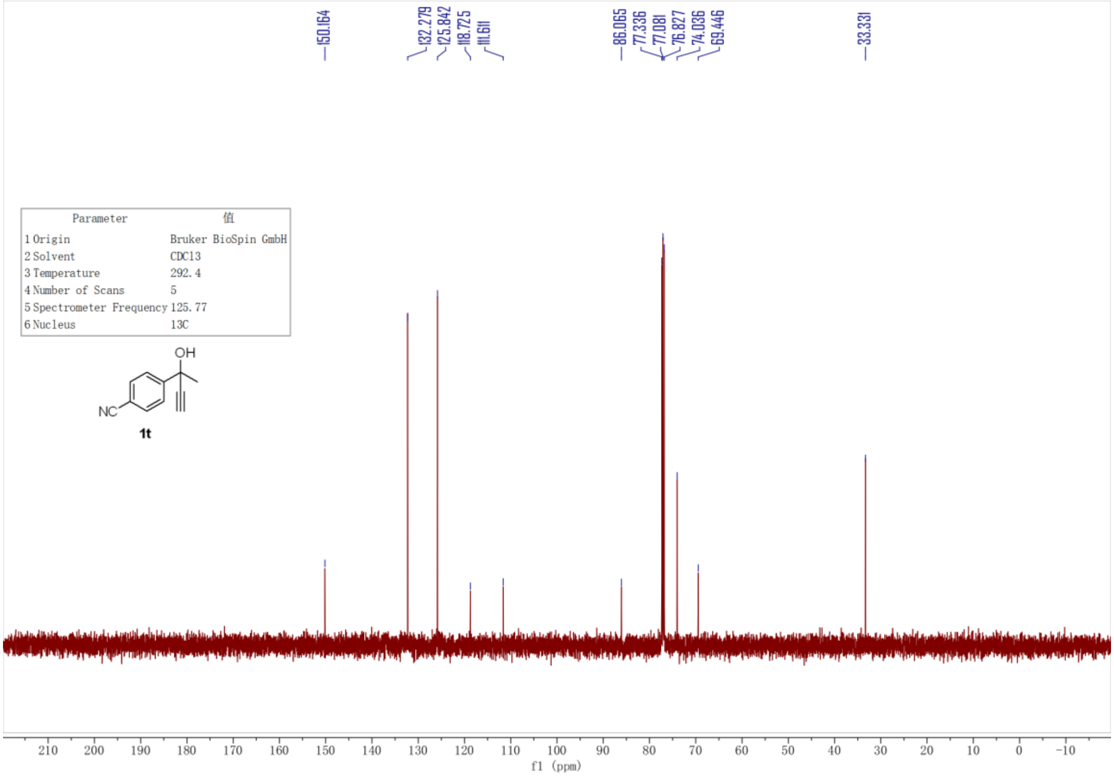
150.164
132.279
125.842
118.725
111.611
86.065
77.336
77.081
76.827
74.036
69.446
33.331
Parameter 值
1 Origin Bruker BioSpin GmbH
2 Solvent CDCl3
3 Temperature 292.4
4 Number of Scans 5
5 Spectrometer Frequency 125.77
6 Nucleus 13C
OH
NC
1t
f1 (ppm)

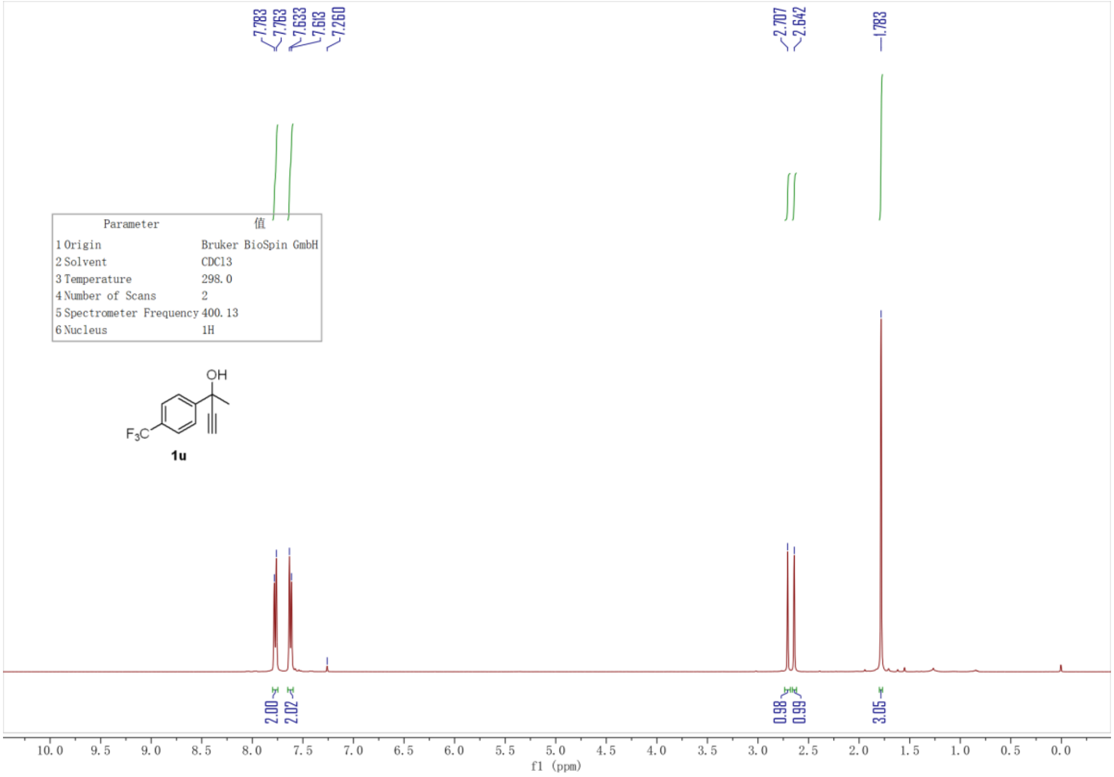
7.783
7.763
7.633
7.613
7.260
2.707
2.642
1.783
Parameter 值
1 Origin Bruker BioSpin GmbH
2 Solvent CDCl3
3 Temperature 298.0
4 Number of Scans 2
5 Spectrometer Frequency 400.13
6 Nucleus 1H
OH
F3C
1u
2.00
2.02
0.98
0.99
3.05
f1 (ppm)

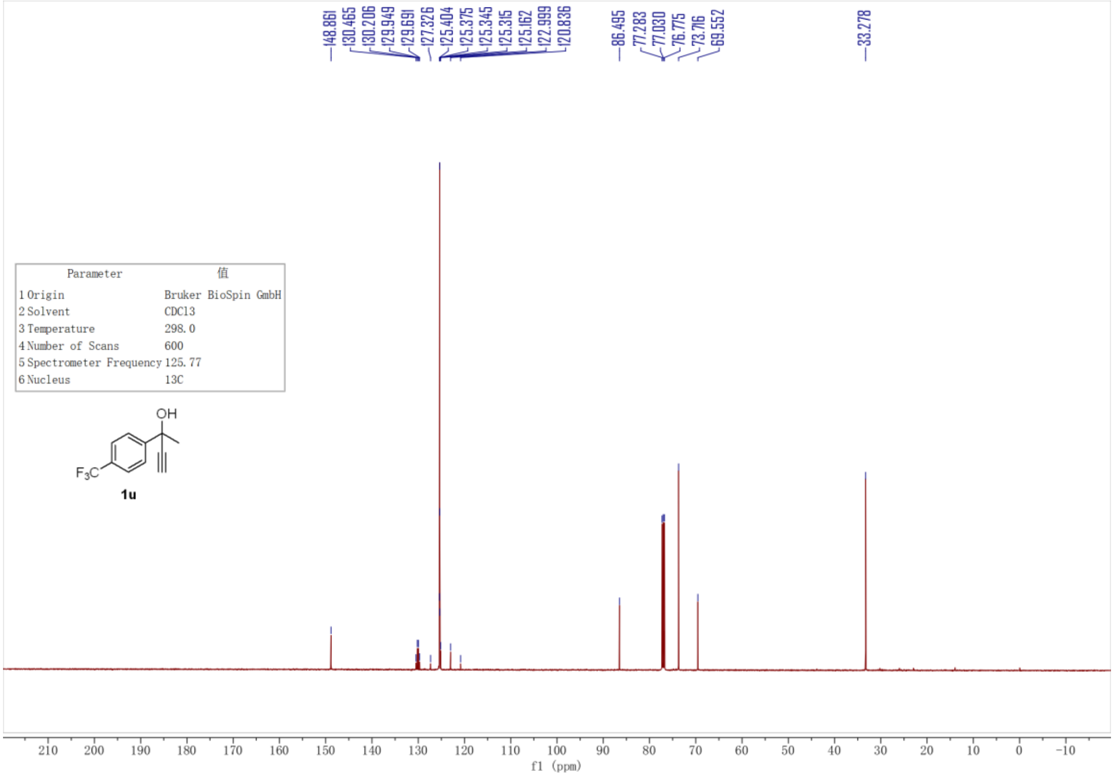

148.861
130.465
130.206
129.949
129.691
127.326
125.404
125.375
125.345
125.315
125.162
122.999
120.836
86.495
77.283
77.030
76.775
73.716
69.552
33.278
Parameter 值
1 Origin Bruker BioSpin GmbH
2 Solvent CDCl3
3 Temperature 298.0
4 Number of Scans 600
5 Spectrometer Frequency 125.77
6 Nucleus 13C
OH
F3C
1u
f1 (ppm)

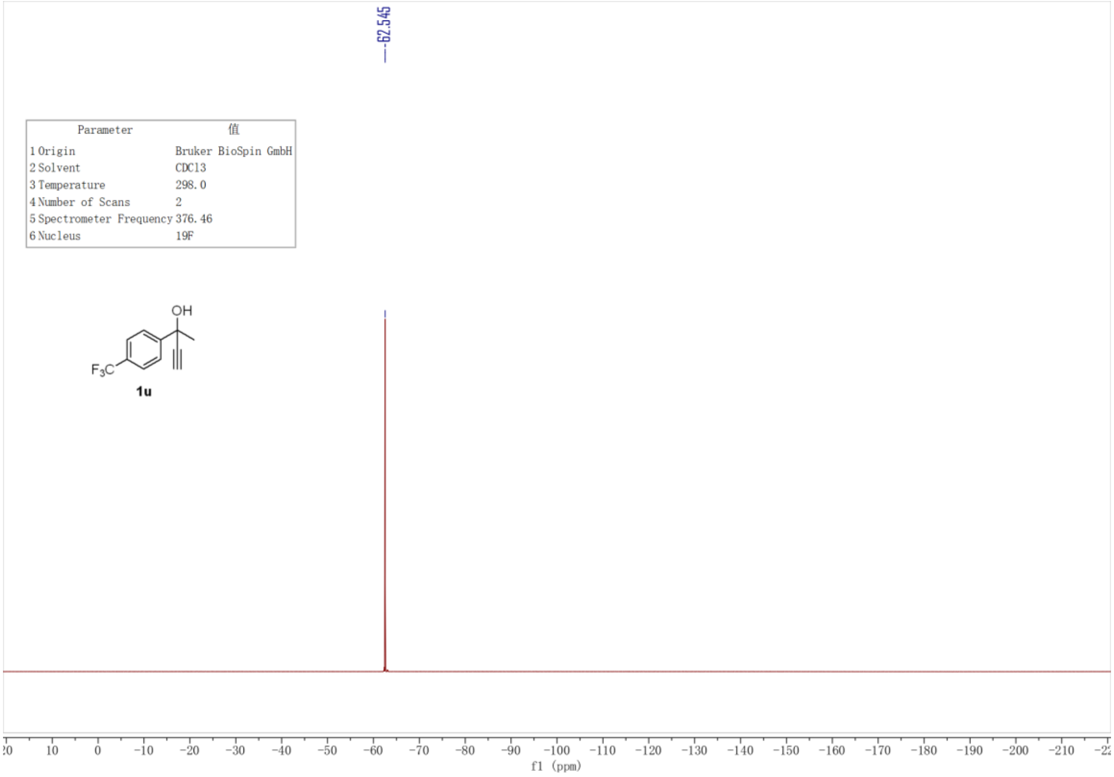

-62.545
Parameter 值
1 Origin Bruker BioSpin GmbH
2 Solvent CDCl3
3 Temperature 298.0
4 Number of Scans 2
5 Spectrometer Frequency 376.46
6 Nucleus 19F
OH
F3C
1u
f1 (ppm)

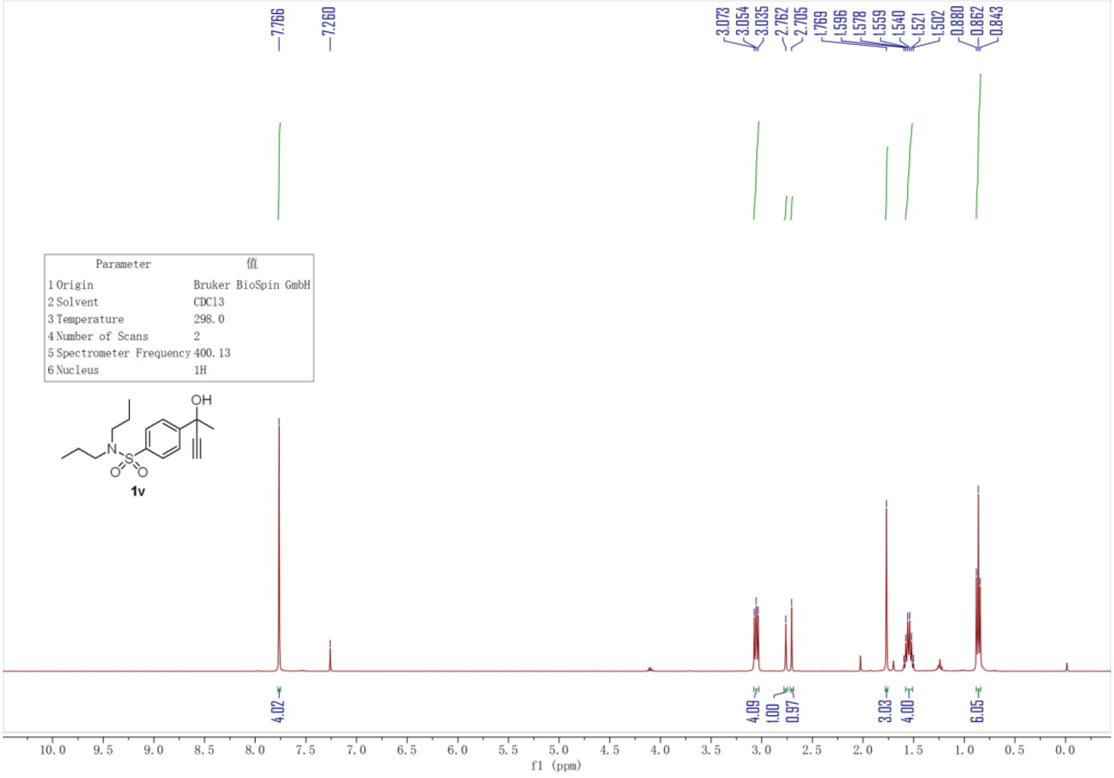

7.766
7.260
3.073
3.054
3.035
2.762
2.705
1.769
1.596
1.578
1.559
1.540
1.521
1.502
0.880
0.862
0.843
Parameter 值
1 Origin Bruker BioSpin GmbH
2 Solvent CDCl3
3 Temperature 298.0
4 Number of Scans 2
5 Spectrometer Frequency 400.13
6 Nucleus 1H
OH
N
S
O O
1v
4.02
4.09
1.00
0.97
3.03
4.00
6.05
10.0 9.5 9.0 8.5 8.0 7.5 7.0 6.5 6.0 5.5 5.0 4.5 4.0 3.5 3.0 2.5 2.0 1.5 1.0 0.5 0.0
f1 (ppm)

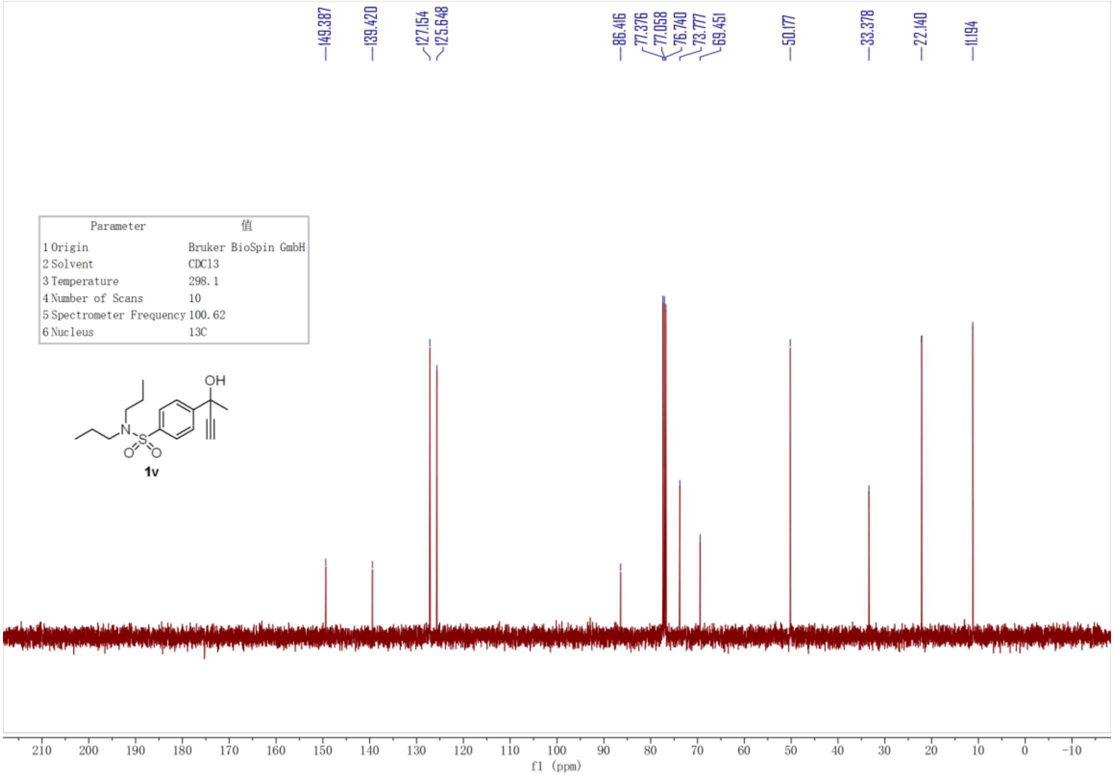

149.387
139.420
127.154
125.648
86.416
77.376
77.058
76.740
73.777
69.451
50.177
33.378
22.140
11.194
Parameter 值
1 Origin Bruker BioSpin GmbH
2 Solvent CDCl3
3 Temperature 298.1
4 Number of Scans 10
5 Spectrometer Frequency 100.62
6 Nucleus 13C
OH
N
S
O O
1v
210 200 190 180 170 160 150 140 130 120 110 100 90 80 70 60 50 40 30 20 10 0 -10
f1 (ppm)

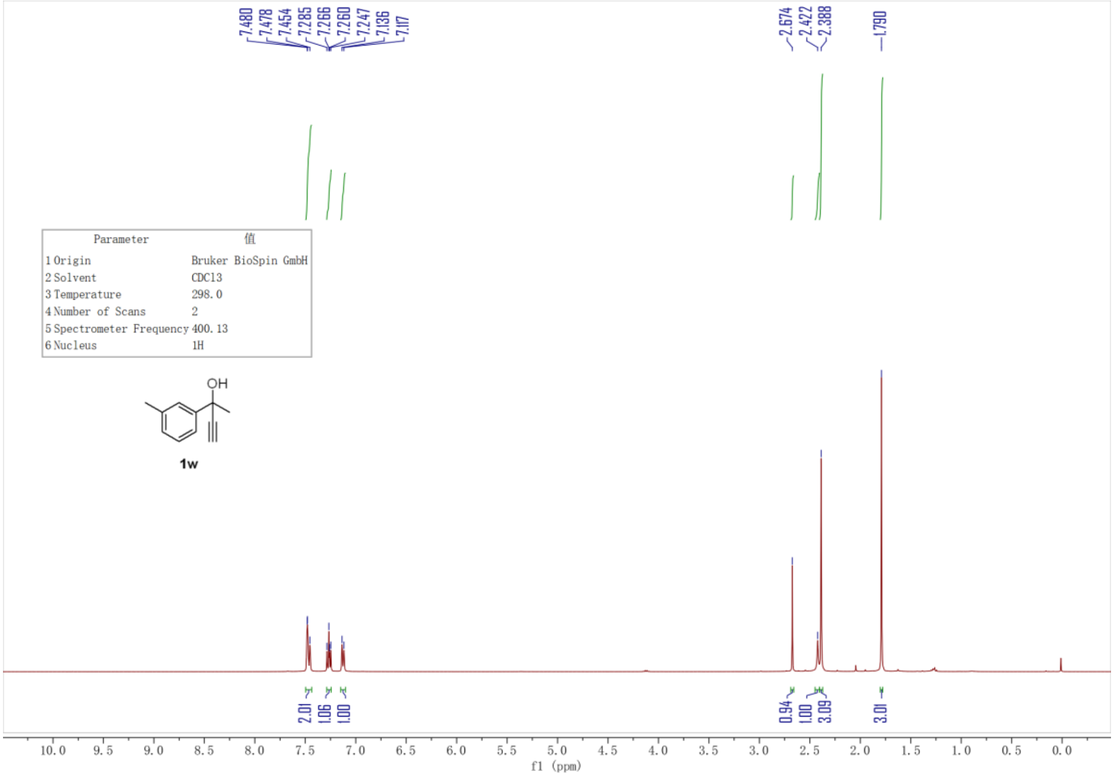

7.480
7.478
7.454
7.285
7.266
7.260
7.247
7.136
7.117
2.674
2.422
2.388
1.790
Parameter
值
1 Origin
Bruker BioSpin GmbH
2 Solvent
CDCl3
3 Temperature
298.0
4 Number of Scans
2
5 Spectrometer Frequency
400.13
6 Nucleus
1H
OH
1w
2.01
1.06
1.00
0.94
1.00
3.09
3.01
f1 (ppm)

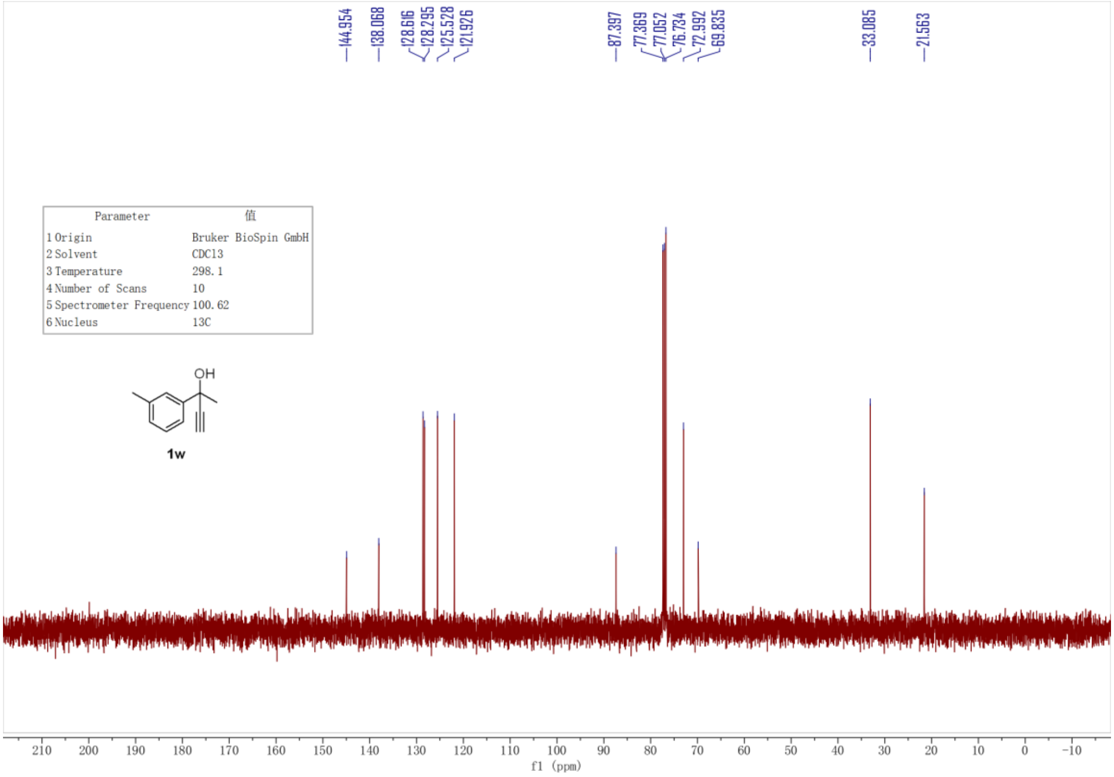

144.954
138.068
128.616
128.295
125.528
121.926
87.397
77.369
77.052
76.734
72.992
69.835
33.085
21.563
Parameter
值
1 Origin
Bruker BioSpin GmbH
2 Solvent
CDCl3
3 Temperature
298.1
4 Number of Scans
10
5 Spectrometer Frequency
100.62
6 Nucleus
13C
OH
1w
f1 (ppm)

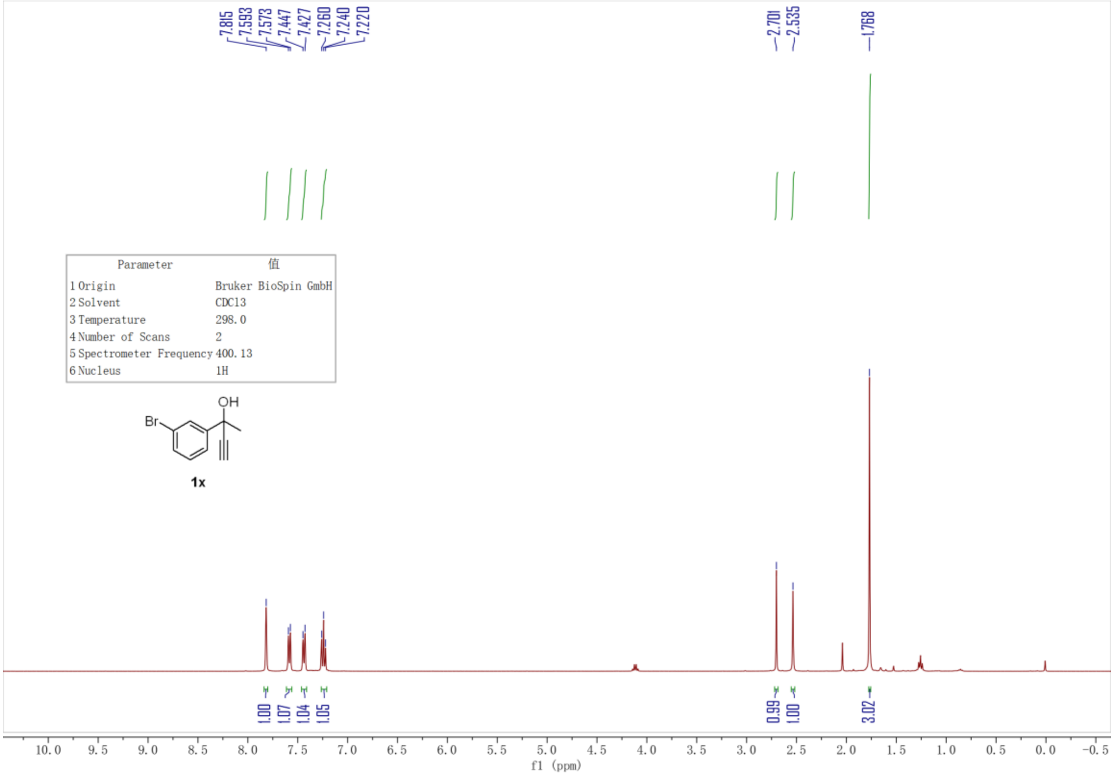
7.815
7.593
7.573
7.447
7.427
7.260
7.240
7.220
2.701
2.535
1.768
Parameter 值
1 Origin Bruker BioSpin GmbH
2 Solvent CDCl3
3 Temperature 298.0
4 Number of Scans 2
5 Spectrometer Frequency 400.13
6 Nucleus 1H
OH
Br
1x
1.00
1.07
1.04
1.05
0.99
1.00
3.02
f1 (ppm)

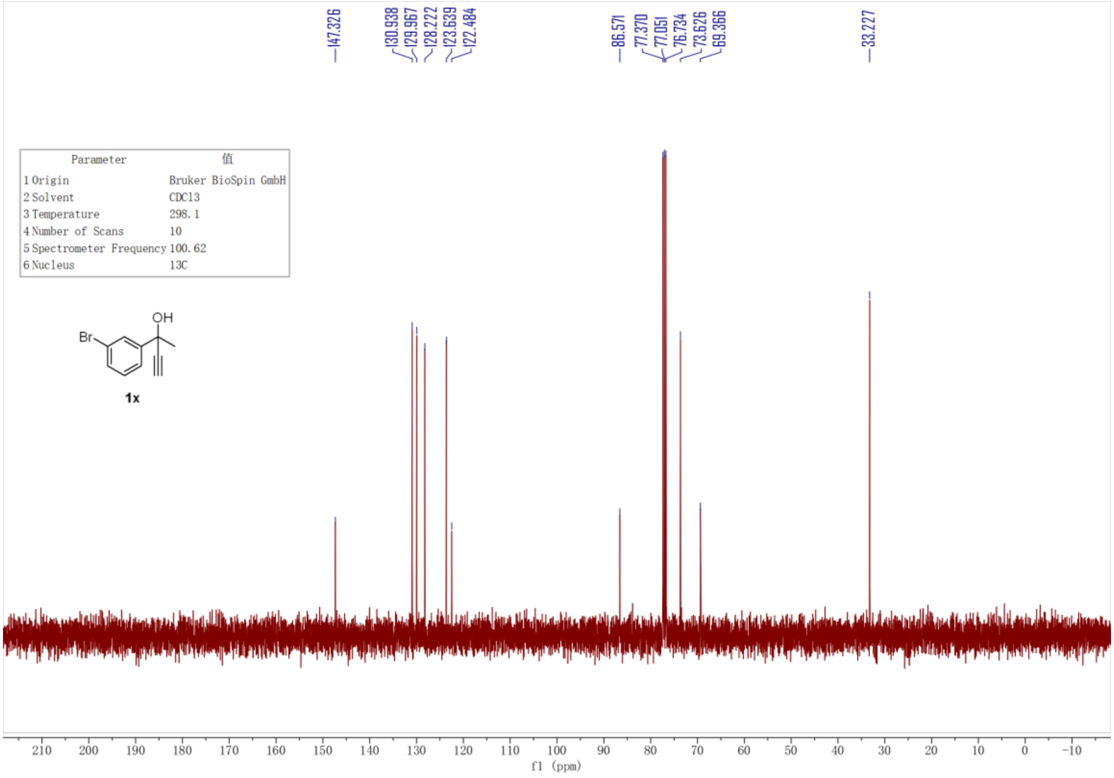
147.326
130.938
129.967
128.222
123.639
122.484
86.571
77.370
77.051
76.734
73.626
69.366
33.227
Parameter 值
1 Origin Bruker BioSpin GmbH
2 Solvent CDCl3
3 Temperature 298.1
4 Number of Scans 10
5 Spectrometer Frequency 100.62
6 Nucleus 13C
OH
Br
1x
f1 (ppm)

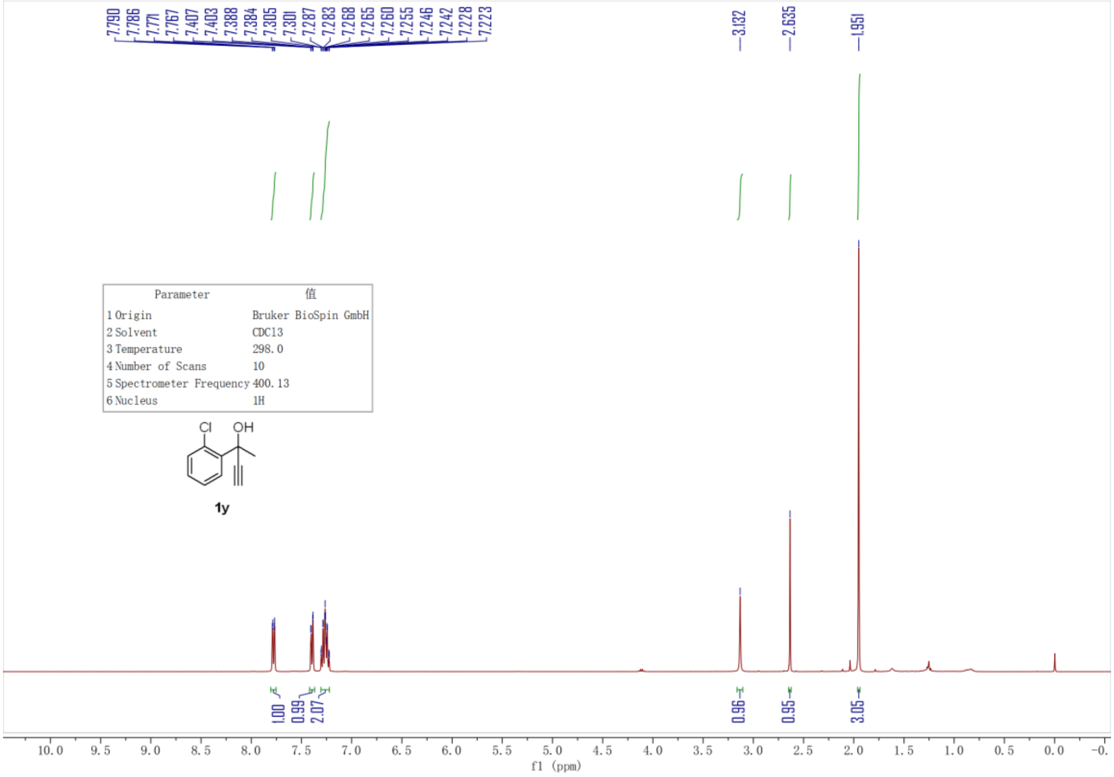

Parameter	值
1 Origin	Bruker BioSpin GmbH
2 Solvent	CDCl3
3 Temperature	298.0
4 Number of Scans	10
5 Spectrometer Frequency	400.13
6 Nucleus	1H

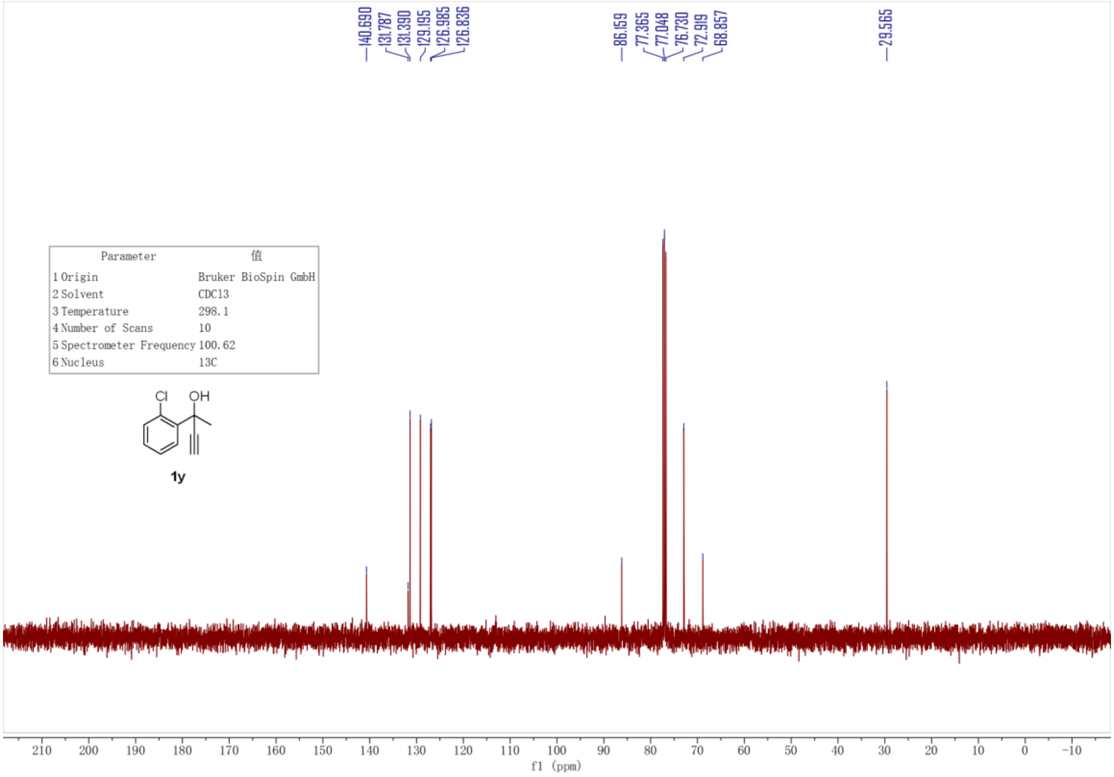

Parameter	值
1 Origin	Bruker BioSpin GmbH
2 Solvent	CDCl3
3 Temperature	298.1
4 Number of Scans	10
5 Spectrometer Frequency	100.62
6 Nucleus	13C

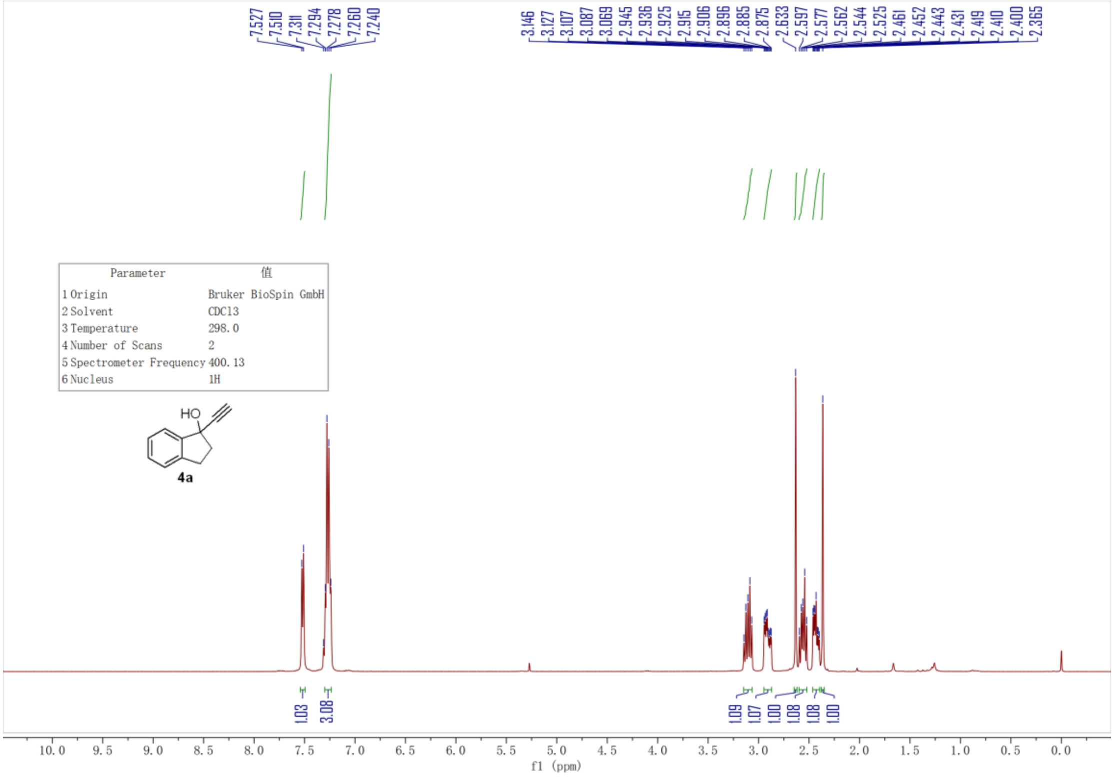
7.527
7.510
7.311
7.294
7.278
7.260
7.240
3.146
3.127
3.107
3.087
3.069
2.945
2.936
2.925
2.915
2.906
2.896
2.885
2.875
2.633
2.597
2.577
2.562
2.544
2.525
2.461
2.452
2.443
2.431
2.419
2.410
2.400
2.365
Parameter 值
1 Origin Bruker BioSpin GmbH
2 Solvent CDCl3
3 Temperature 298.0
4 Number of Scans 2
5 Spectrometer Frequency 400.13
6 Nucleus 1H
HO
4a
1.03
3.08
1.09
1.07
1.00
1.08
1.08
1.00
f1 (ppm)

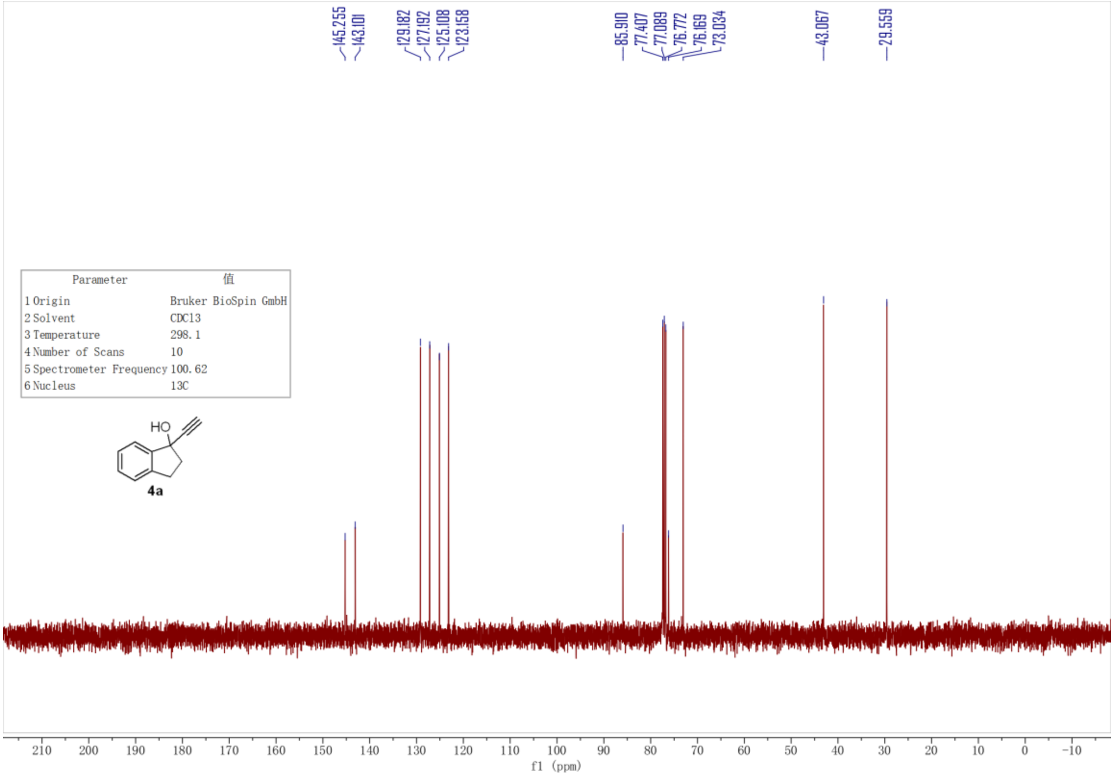
145.255
143.101
129.182
127.192
125.108
123.158
85.910
77.407
77.089
76.772
76.169
73.034
43.067
29.559
Parameter 值
1 Origin Bruker BioSpin GmbH
2 Solvent CDCl3
3 Temperature 298.1
4 Number of Scans 10
5 Spectrometer Frequency 100.62
6 Nucleus 13C
HO
4a
f1 (ppm)

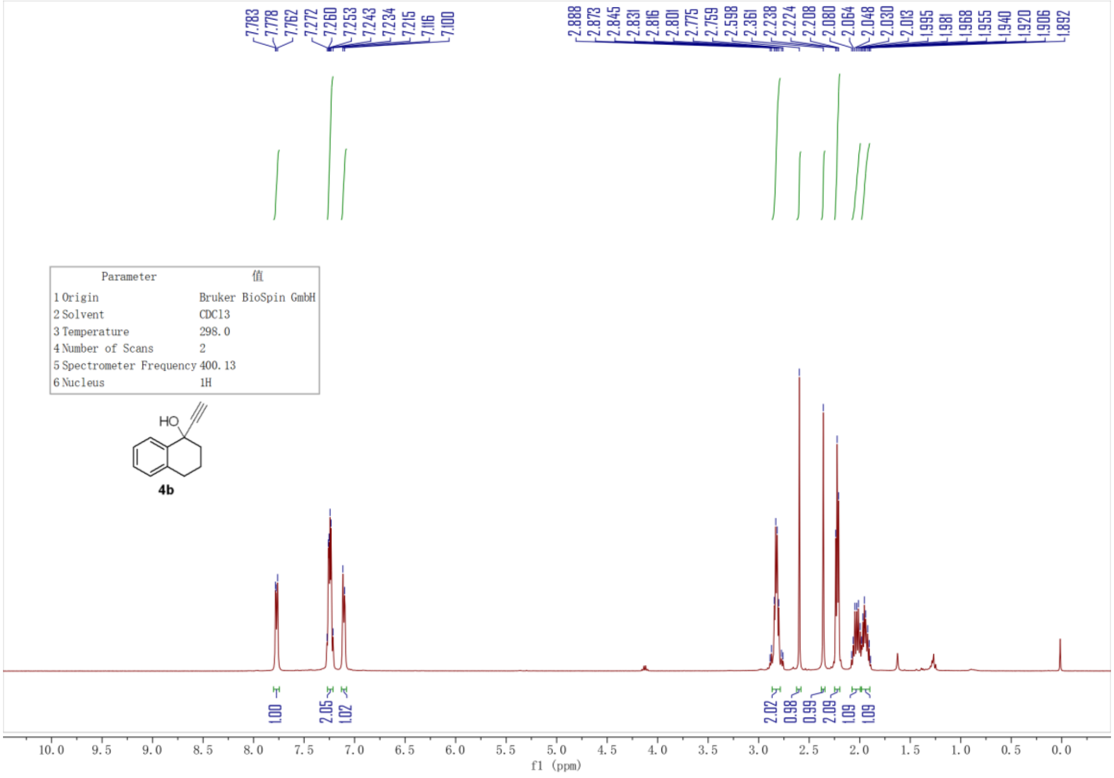

Parameter	值
1 Origin	Bruker BioSpin GmbH
2 Solvent	CDCl3
3 Temperature	298.0
4 Number of Scans	2
5 Spectrometer Frequency	400.13
6 Nucleus	1H

4b

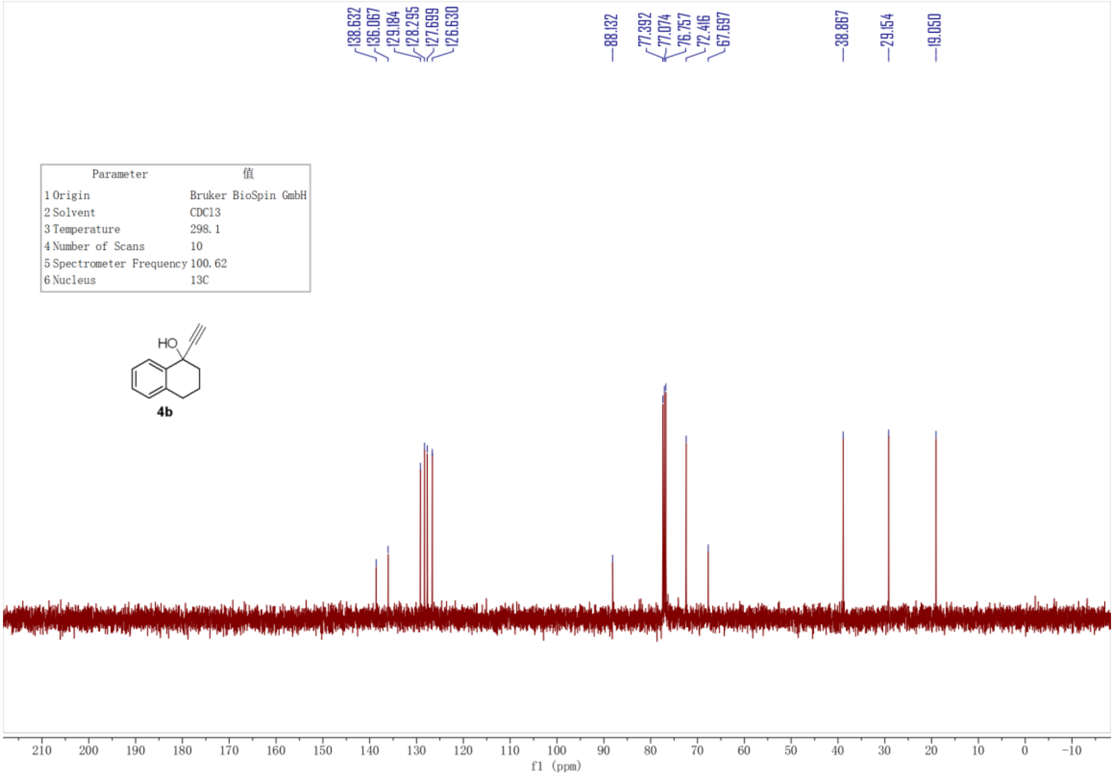

Parameter	值
1 Origin	Bruker BioSpin GmbH
2 Solvent	CDCl3
3 Temperature	298.1
4 Number of Scans	10
5 Spectrometer Frequency	100.62
6 Nucleus	13C

4b

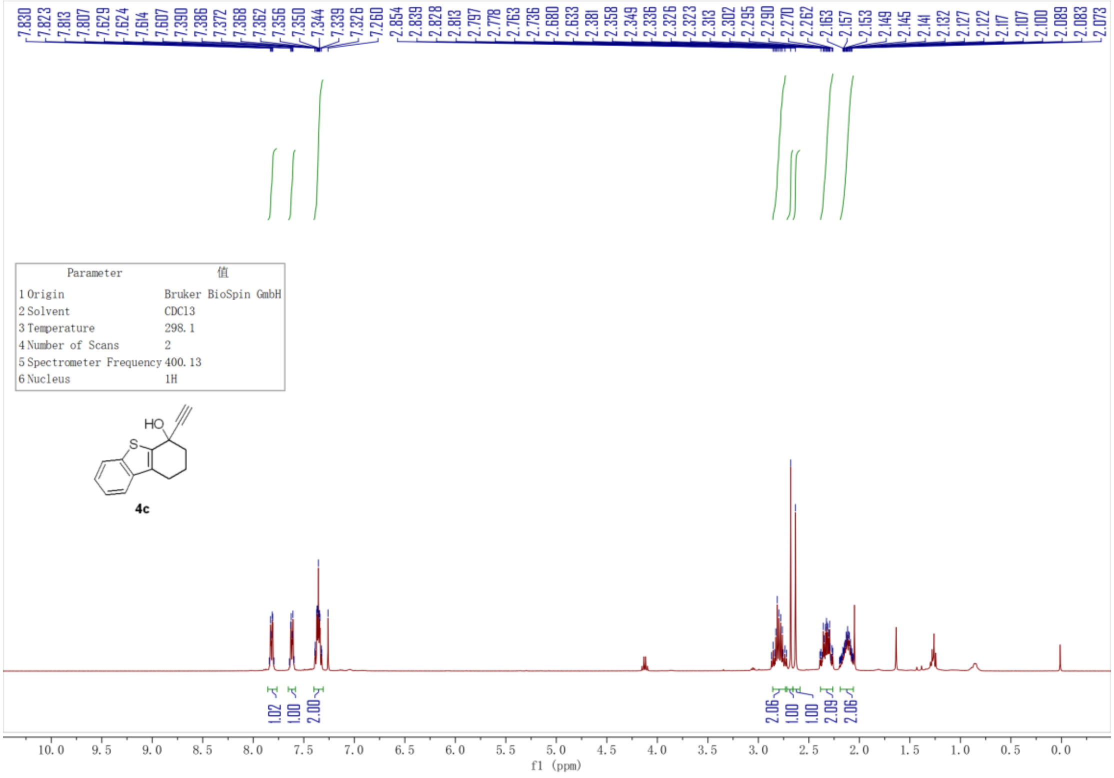

7.830 7.823 7.813 7.807 7.629 7.624 7.614 7.607 7.390 7.386 7.372 7.368 7.362 7.356 7.350 7.344 7.339 7.326 7.260 2.854 2.839 2.828 2.813 2.797 2.778 2.763 2.736 2.680 2.633 2.381 2.358 2.349 2.336 2.326 2.323 2.313 2.302 2.295 2.290 2.270 2.262 2.163 2.157 2.153 2.149 2.145 2.141 2.132 2.127 2.122 2.117 2.107 2.100 2.089 2.083 2.073
Parameter 值
1 Origin Bruker BioSpin GmbH
2 Solvent CDCl3
3 Temperature 298.1
4 Number of Scans 2
5 Spectrometer Frequency 400.13
6 Nucleus 1H
HO
S
4c
1.02 1.00 2.00 2.06 1.00 1.00 2.09 2.06
10.0 9.5 9.0 8.5 8.0 7.5 7.0 6.5 6.0 5.5 5.0 4.5 4.0 3.5 3.0 2.5 2.0 1.5 1.0 0.5 0.0
f1 (ppm)

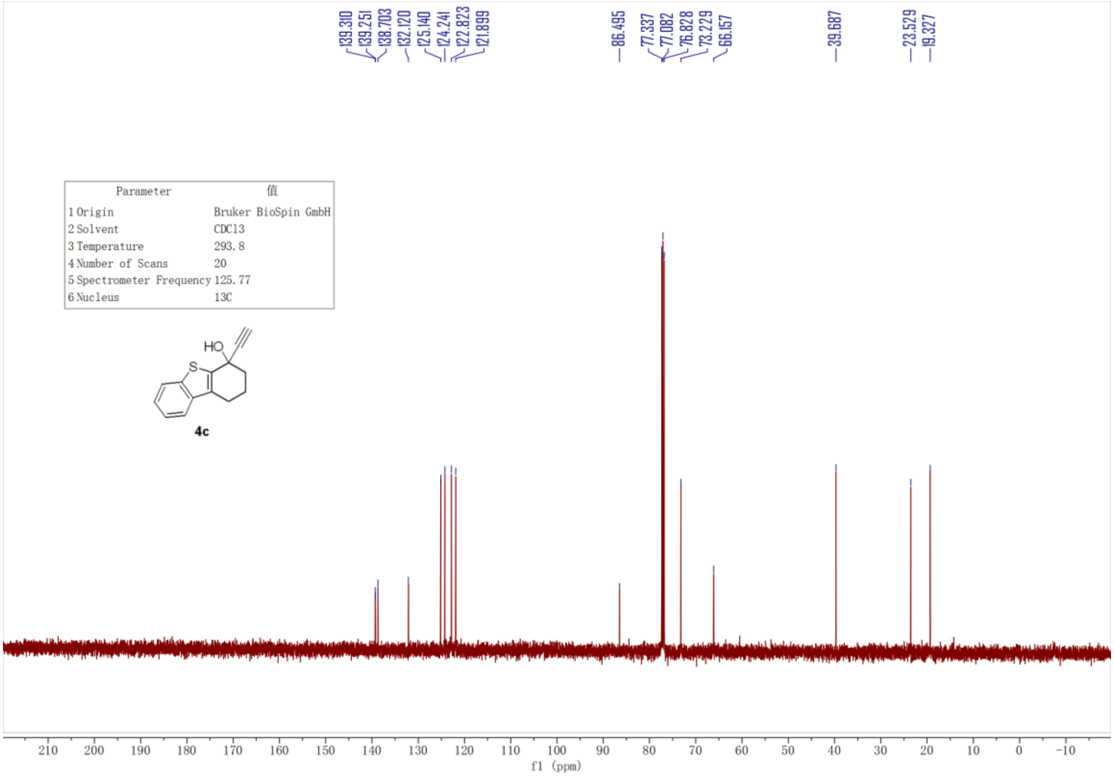

139.310 139.251 138.703 132.120 125.140 124.241 122.823 121.899 86.495 77.337 77.082 76.828 73.229 66.157 39.687 23.529 19.327
Parameter 值
1 Origin Bruker BioSpin GmbH
2 Solvent CDCl3
3 Temperature 293.8
4 Number of Scans 20
5 Spectrometer Frequency 125.77
6 Nucleus 13C
HO
S
4c
210 200 190 180 170 160 150 140 130 120 110 100 90 80 70 60 50 40 30 20 10 0 -10
f1 (ppm)

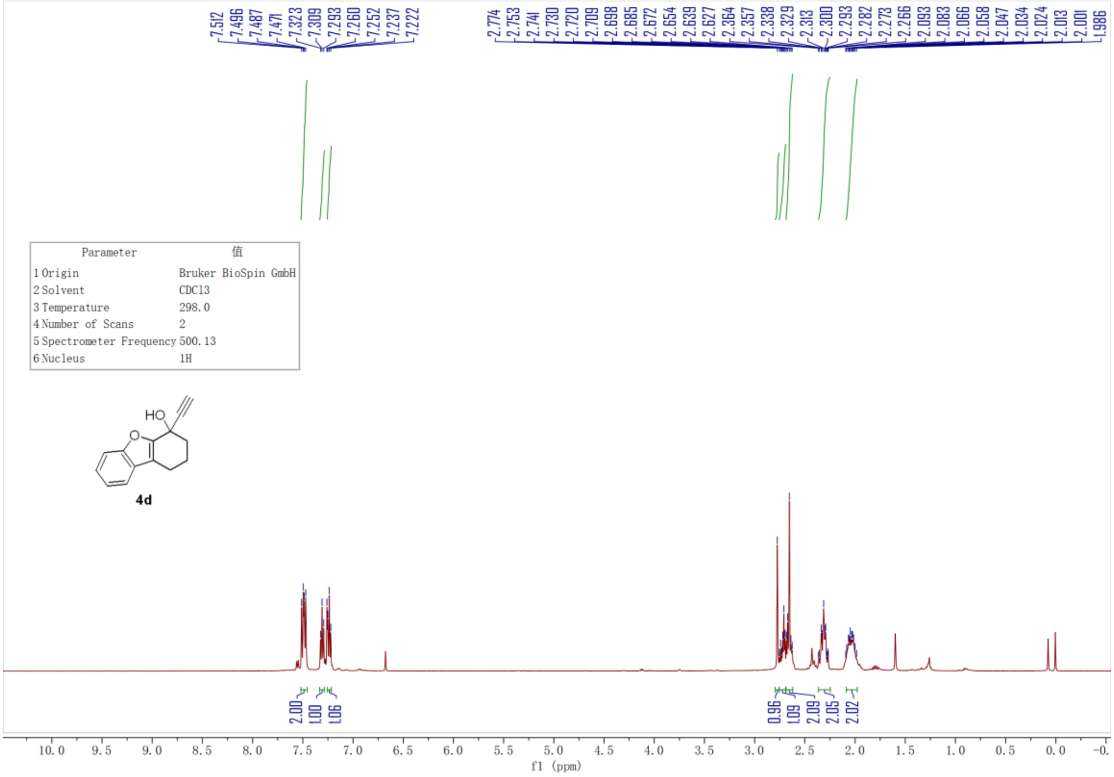
7.512
7.496
7.487
7.471
7.323
7.309
7.293
7.260
7.252
7.237
7.222
2.774
2.753
2.741
2.730
2.720
2.709
2.698
2.685
2.672
2.654
2.639
2.627
2.364
2.357
2.338
2.329
2.313
2.300
2.293
2.282
2.273
2.266
2.093
2.083
2.066
2.058
2.047
2.034
2.024
2.013
2.001
1.986
Parameter 值
1 Origin Bruker BioSpin GmbH
2 Solvent CDCl3
3 Temperature 298.0
4 Number of Scans 2
5 Spectrometer Frequency 500.13
6 Nucleus 1H
HO
O
4d
2.00
1.00
1.06
0.96
1.09
2.09
2.05
2.02
f1 (ppm)

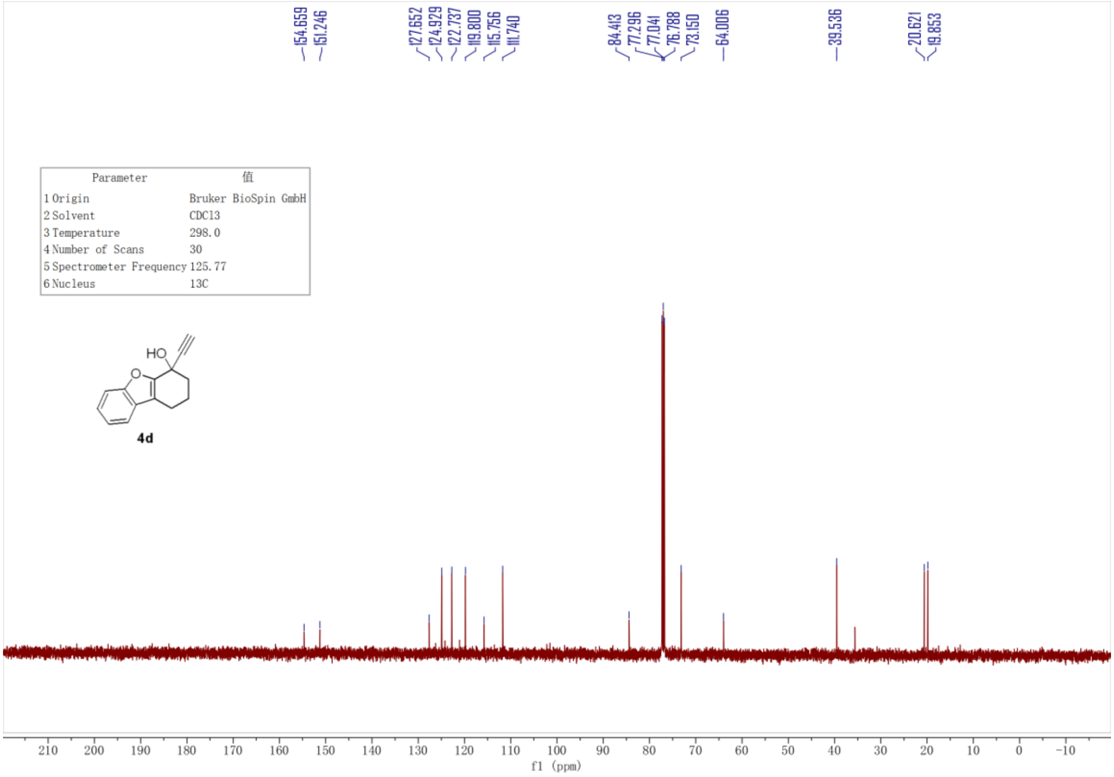
154.659
151.246
127.652
124.929
122.737
119.800
115.756
111.740
84.413
77.296
77.041
76.788
73.150
64.006
39.536
20.621
19.853
Parameter 值
1 Origin Bruker BioSpin GmbH
2 Solvent CDCl3
3 Temperature 298.0
4 Number of Scans 30
5 Spectrometer Frequency 125.77
6 Nucleus 13C
HO
O
4d
f1 (ppm)

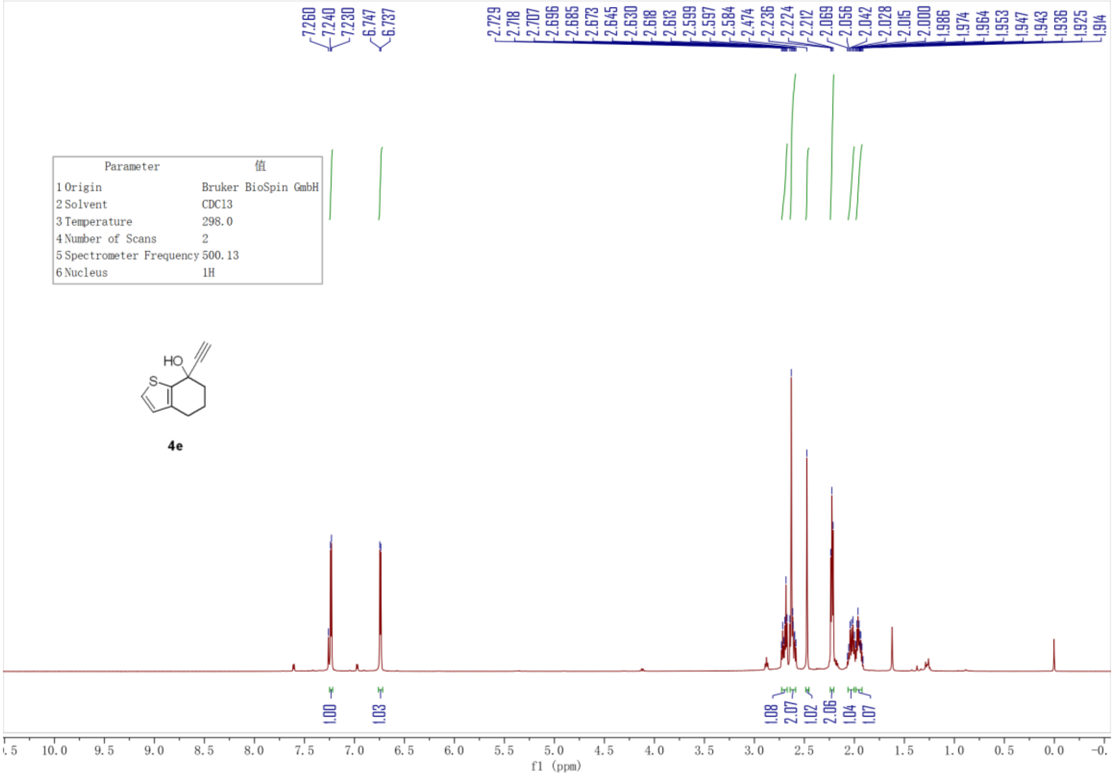

Parameter 值
1 Origin Bruker BioSpin GmbH
2 Solvent CDCl3
3 Temperature 298.0
4 Number of Scans 2
5 Spectrometer Frequency 500.13
6 Nucleus 1H
HO
S
4e
7.260
7.240
7.230
6.747
6.737
2.729
2.718
2.707
2.696
2.685
2.673
2.645
2.630
2.618
2.613
2.599
2.597
2.584
2.474
2.236
2.224
2.212
2.069
2.056
2.042
2.028
2.015
2.000
1.986
1.974
1.964
1.953
1.947
1.943
1.936
1.925
1.914
1.00
1.03
1.08
2.07
1.02
2.06
1.04
1.07
f1 (ppm)

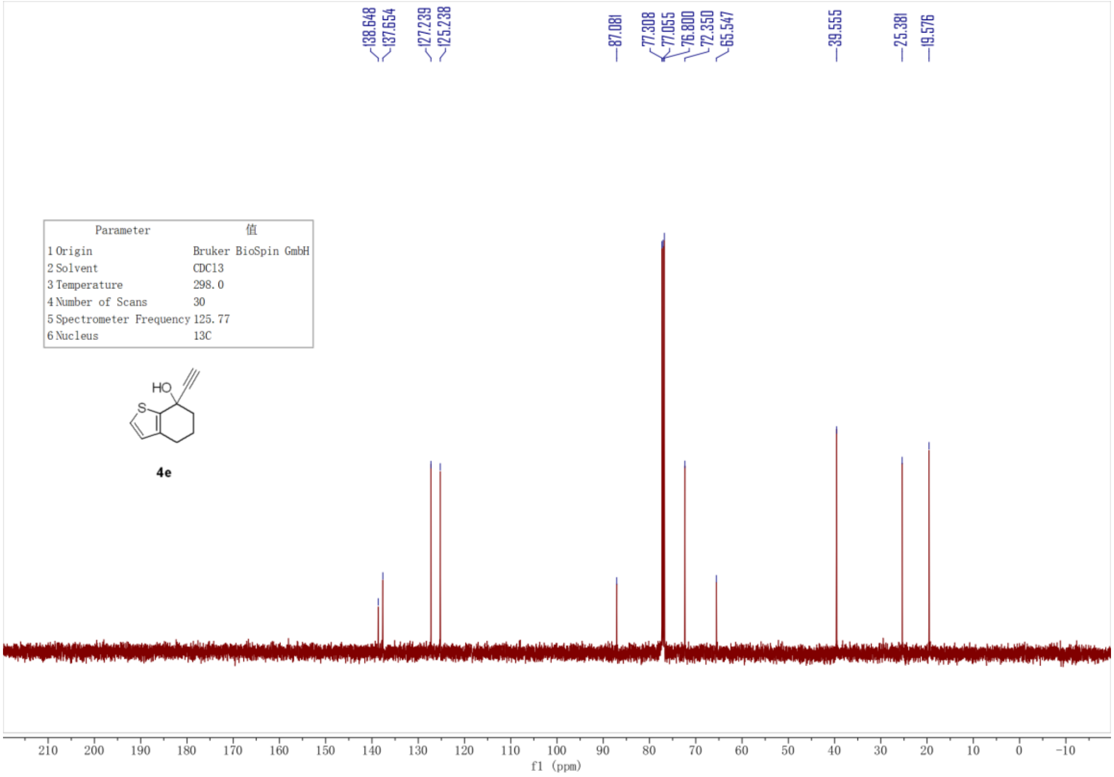

Parameter 值
1 Origin Bruker BioSpin GmbH
2 Solvent CDCl3
3 Temperature 298.0
4 Number of Scans 30
5 Spectrometer Frequency 125.77
6 Nucleus 13C
HO
S
4e
138.648
137.654
127.239
125.238
87.081
77.308
77.055
76.800
72.350
65.547
39.555
25.381
19.576
f1 (ppm)

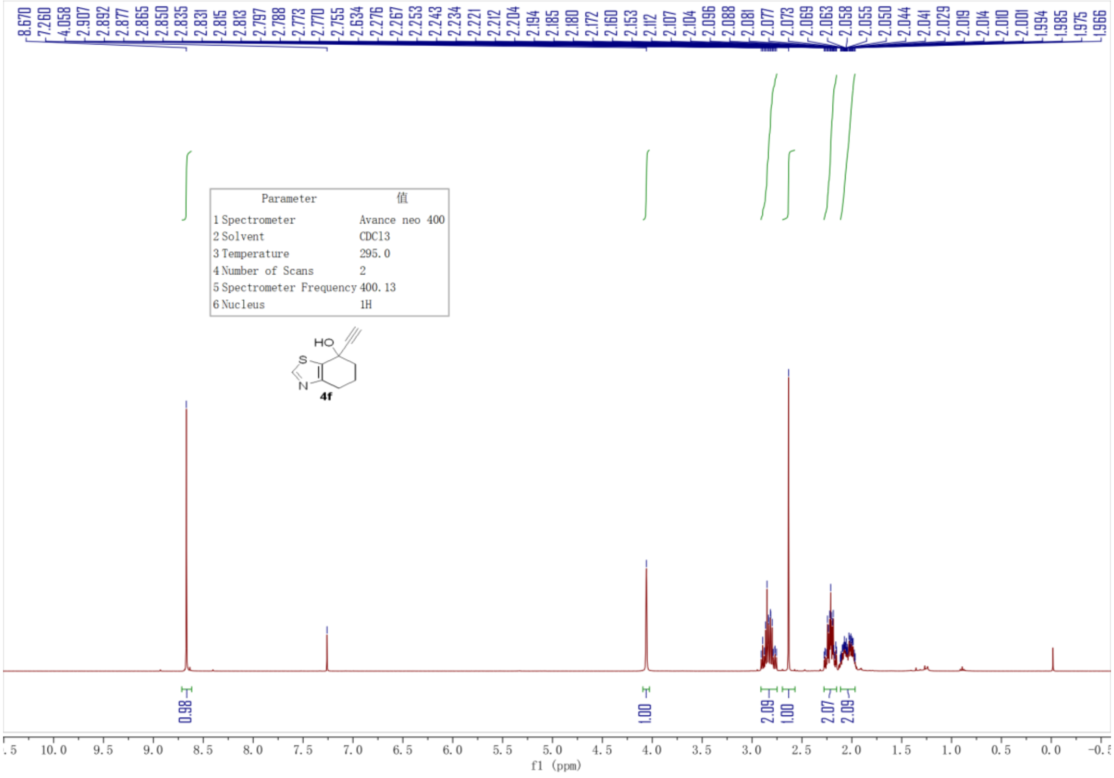

8.670 7.260 4.058 2.907 2.892 2.877 2.865 2.850 2.835 2.831 2.815 2.813 2.797 2.788 2.773 2.770 2.755 2.634 2.276 2.267 2.253 2.243 2.234 2.221 2.212 2.204 2.194 2.185 2.180 2.172 2.160 2.153 2.112 2.107 2.104 2.096 2.088 2.081 2.077 2.073 2.069 2.063 2.058 2.055 2.050 2.044 2.041 2.029 2.019 2.014 2.010 2.001 1.994 1.985 1.975 1.966
Parameter 值
1 Spectrometer Avance neo 400
2 Solvent CDCl3
3 Temperature 295.0
4 Number of Scans 2
5 Spectrometer Frequency 400.13
6 Nucleus 1H
HO
S
N
4f
0.98 1.00 2.09 1.00 2.07 2.09
f1 (ppm)

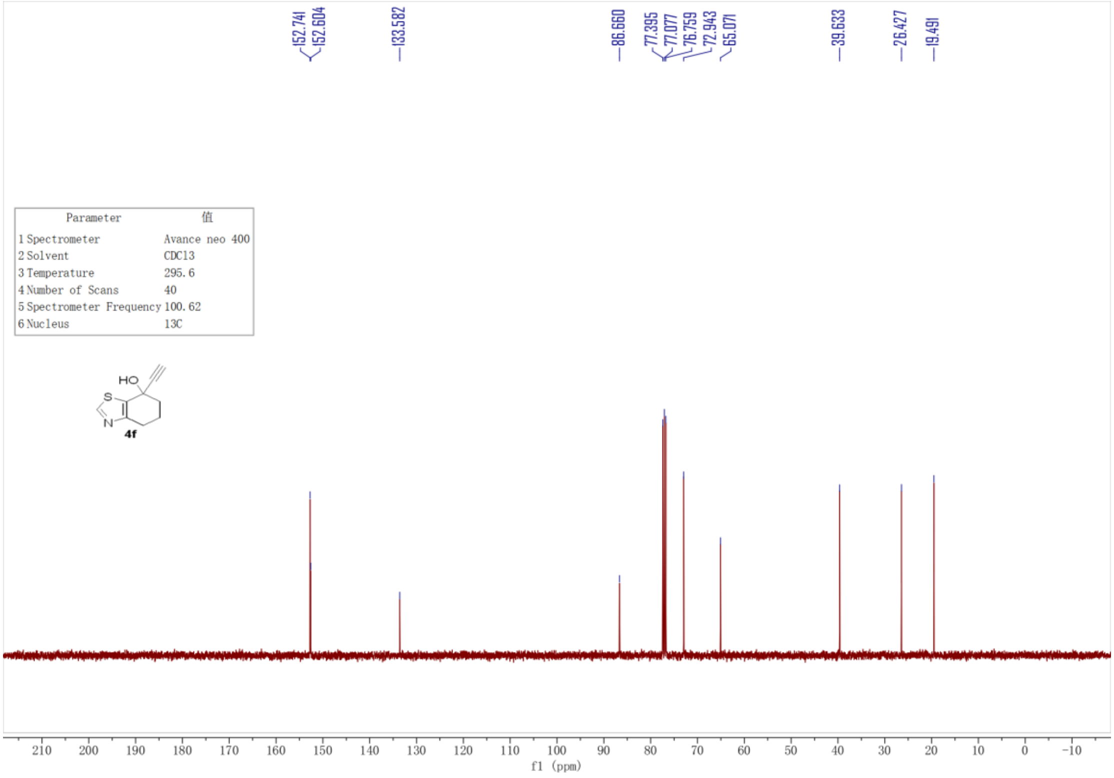

152.741 152.604 133.582 86.660 77.395 77.077 76.759 72.943 65.071 39.633 26.427 19.491
Parameter 值
1 Spectrometer Avance neo 400
2 Solvent CDCl3
3 Temperature 295.6
4 Number of Scans 40
5 Spectrometer Frequency 100.62
6 Nucleus 13C
HO
S
N
4f
f1 (ppm)

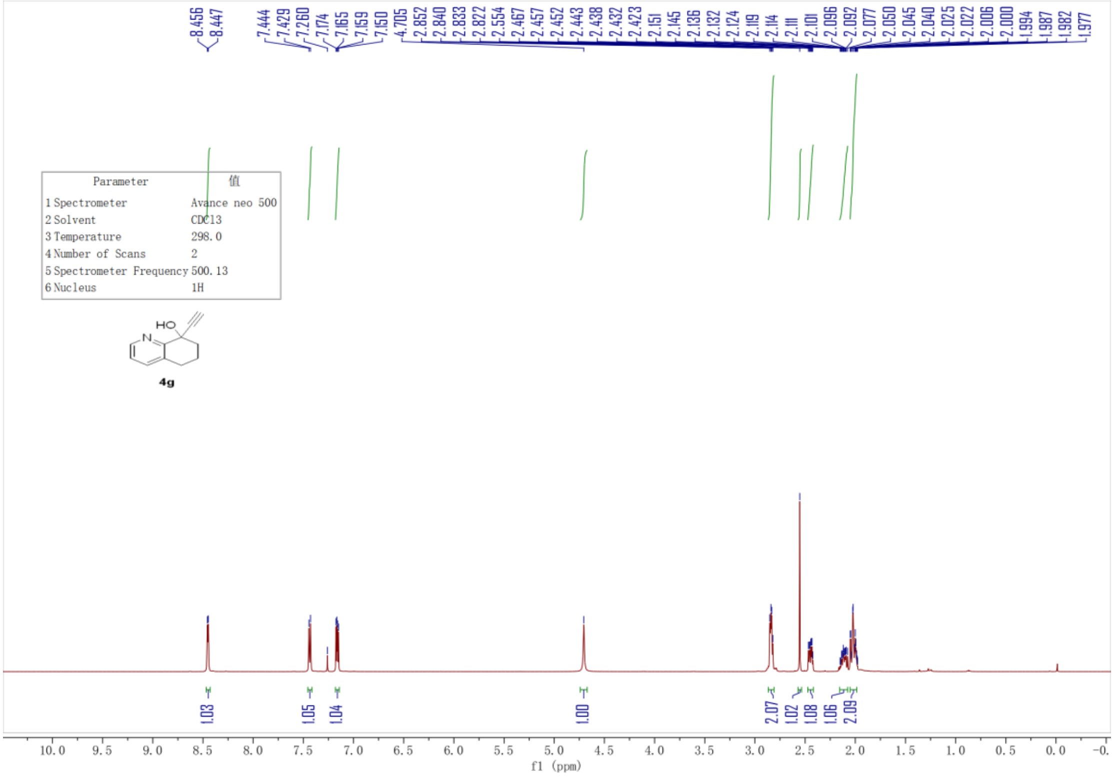
Parameter 值
1 Spectrometer Avance neo 500
2 Solvent CDCl3
3 Temperature 298.0
4 Number of Scans 2
5 Spectrometer Frequency 500.13
6 Nucleus 1H
HO
N
4g
f1 (ppm)

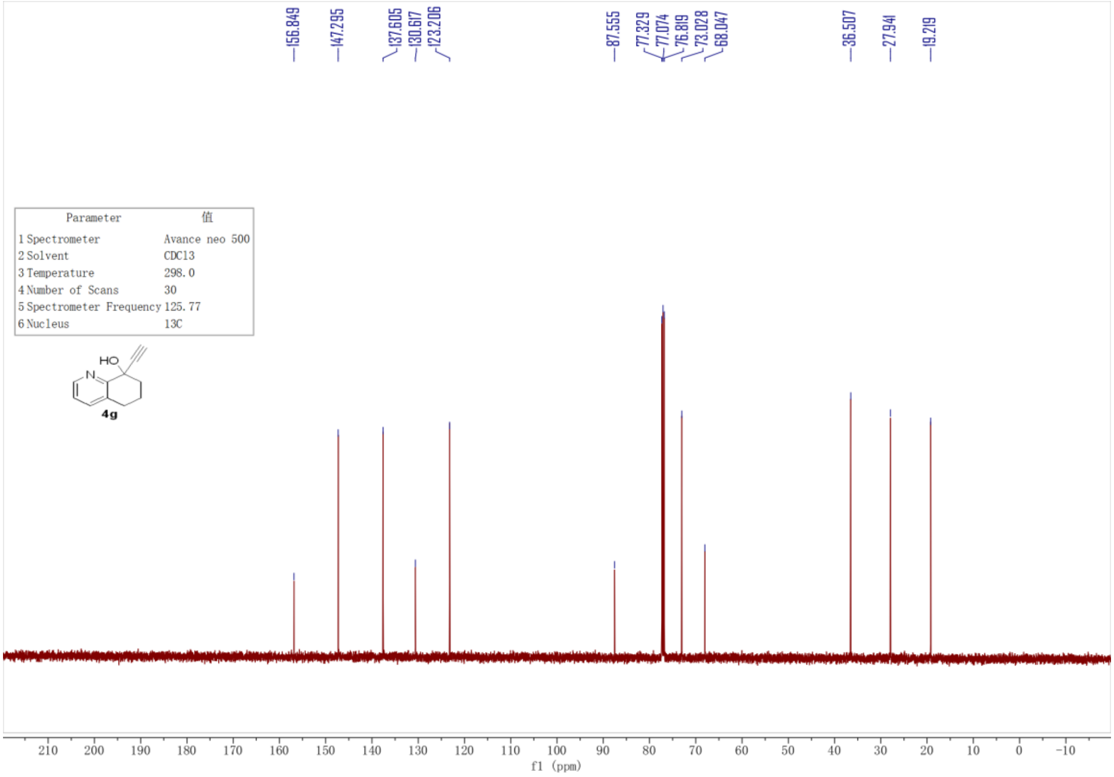
Parameter 值
1 Spectrometer Avance neo 500
2 Solvent CDCl3
3 Temperature 298.0
4 Number of Scans 30
5 Spectrometer Frequency 125.77
6 Nucleus 13C
HO
N
4g
f1 (ppm)

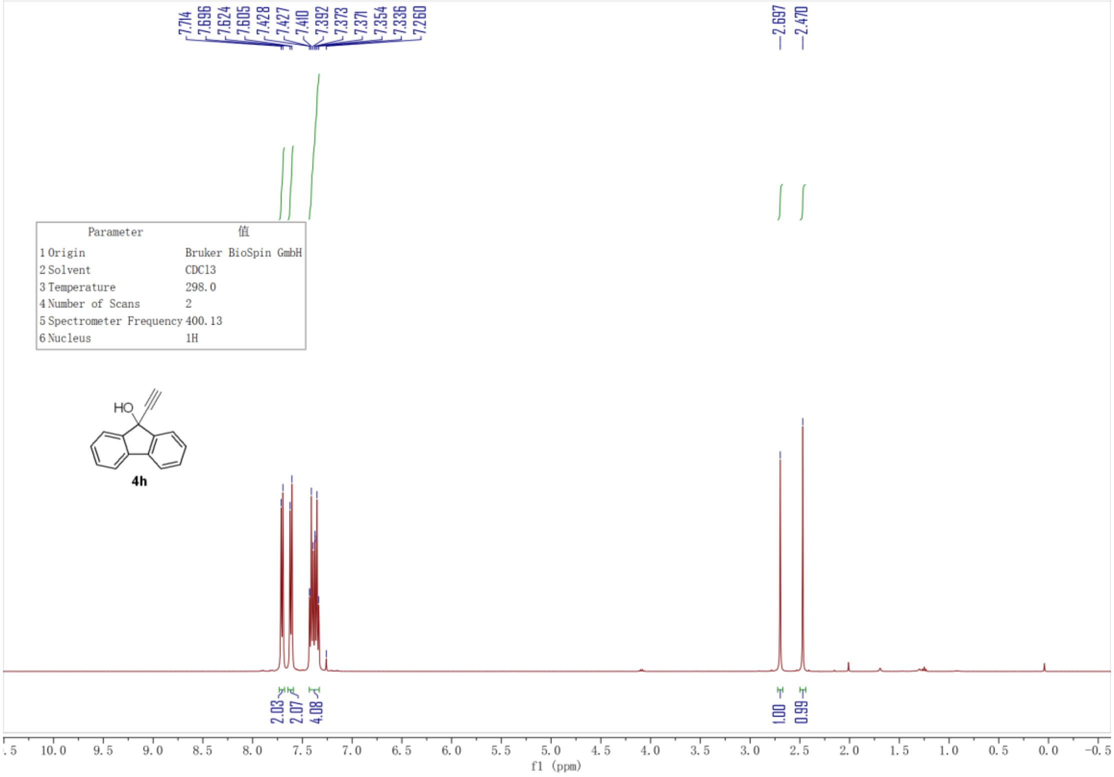
7.714
7.696
7.624
7.605
7.428
7.427
7.410
7.392
7.373
7.371
7.354
7.336
7.260
2.697
2.470
Parameter 值
1 Origin Bruker BioSpin GmbH
2 Solvent CDCl3
3 Temperature 298.0
4 Number of Scans 2
5 Spectrometer Frequency 400.13
6 Nucleus 1H
HO
4h
2.03
2.07
4.08
1.00
0.99
f1 (ppm)

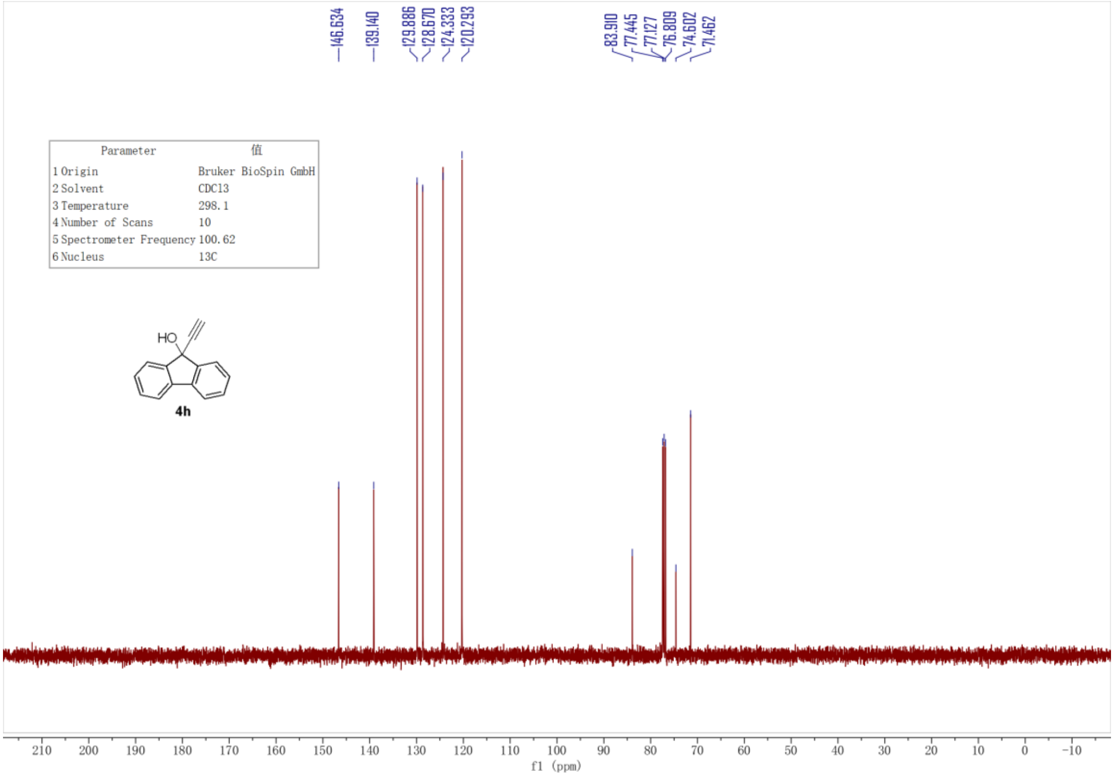
146.634
139.140
129.886
128.670
124.333
120.293
83.910
77.445
77.127
76.809
74.602
71.462
Parameter 值
1 Origin Bruker BioSpin GmbH
2 Solvent CDCl3
3 Temperature 298.1
4 Number of Scans 10
5 Spectrometer Frequency 100.62
6 Nucleus 13C
HO
4h
f1 (ppm)

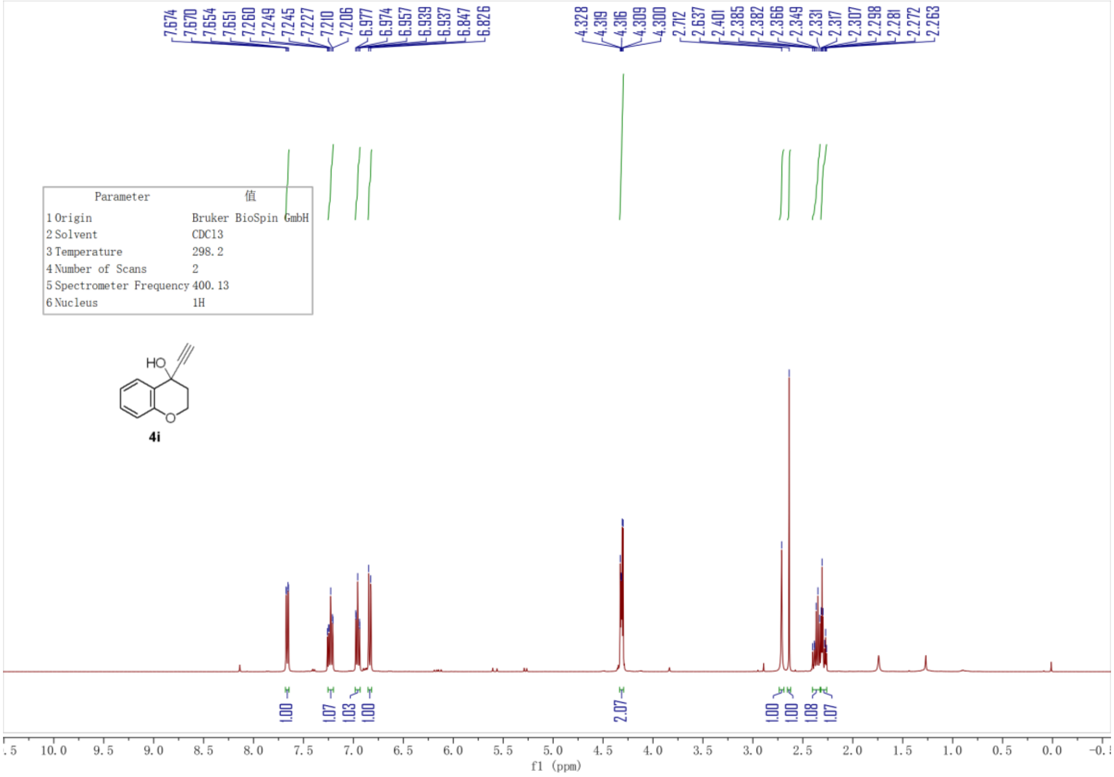
Parameter 值
1 Origin Bruker BioSpin GmbH
2 Solvent CDCl3
3 Temperature 298.2
4 Number of Scans 2
5 Spectrometer Frequency 400.13
6 Nucleus 1H
HO
O
4i
f1 (ppm)

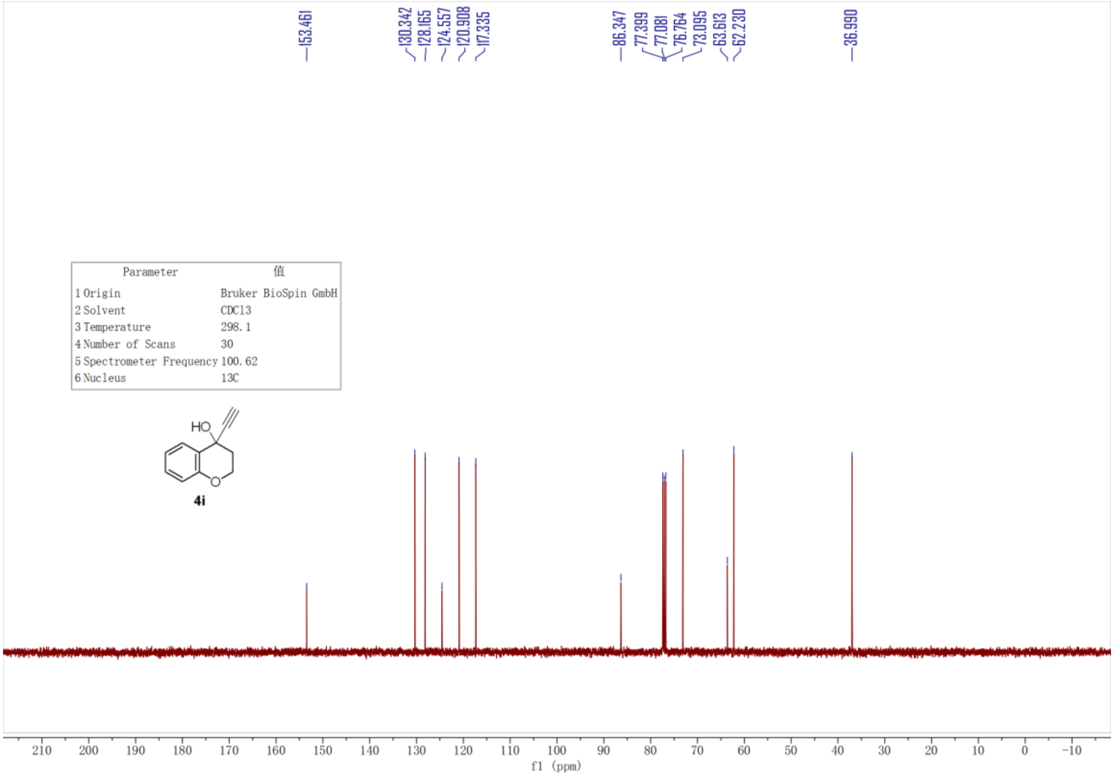
Parameter 值
1 Origin Bruker BioSpin GmbH
2 Solvent CDCl3
3 Temperature 298.1
4 Number of Scans 30
5 Spectrometer Frequency 100.62
6 Nucleus 13C
HO
O
4i
f1 (ppm)

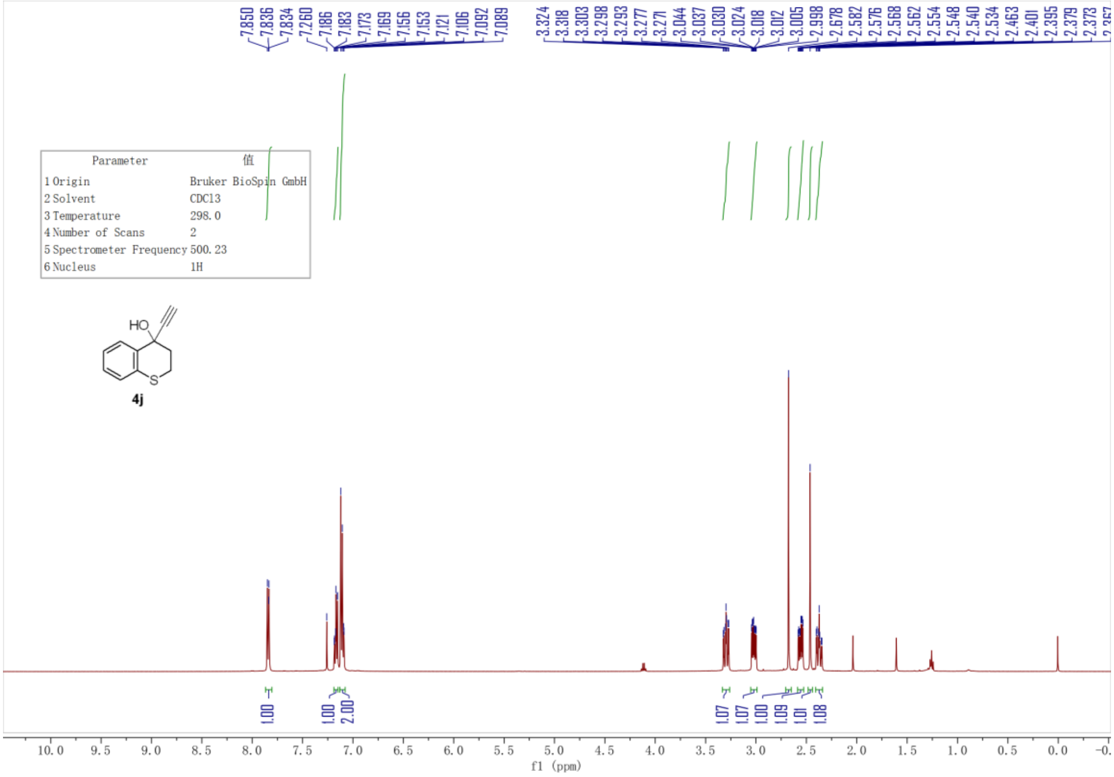
7.850
7.836
7.834
7.260
7.186
7.183
7.173
7.169
7.156
7.153
7.121
7.106
7.092
7.089
3.324
3.318
3.303
3.298
3.293
3.277
3.271
3.044
3.037
3.030
3.024
3.018
3.012
3.005
2.998
2.678
2.582
2.576
2.568
2.562
2.554
2.548
2.540
2.534
2.463
2.401
2.395
2.379
2.373
Parameter 值
1 Origin Bruker BioSpin GmbH
2 Solvent CDCl3
3 Temperature 298.0
4 Number of Scans 2
5 Spectrometer Frequency 500.23
6 Nucleus 1H
HO
S
4j
1.00
1.00
2.00
1.07
1.07
1.00
1.09
1.01
1.08
f1 (ppm)

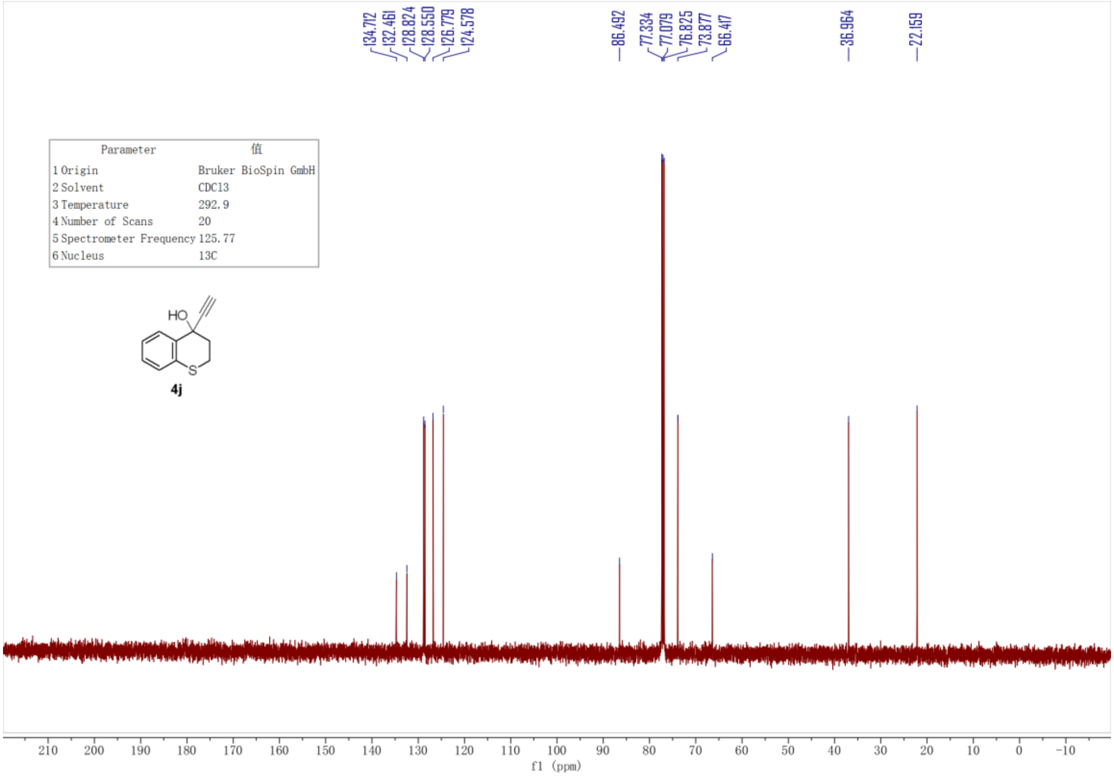
134.712
132.461
128.824
128.550
126.779
124.578
86.492
77.334
77.079
76.825
73.877
66.417
36.964
22.159
Parameter 值
1 Origin Bruker BioSpin GmbH
2 Solvent CDCl3
3 Temperature 292.9
4 Number of Scans 20
5 Spectrometer Frequency 125.77
6 Nucleus 13C
HO
S
4j
f1 (ppm)

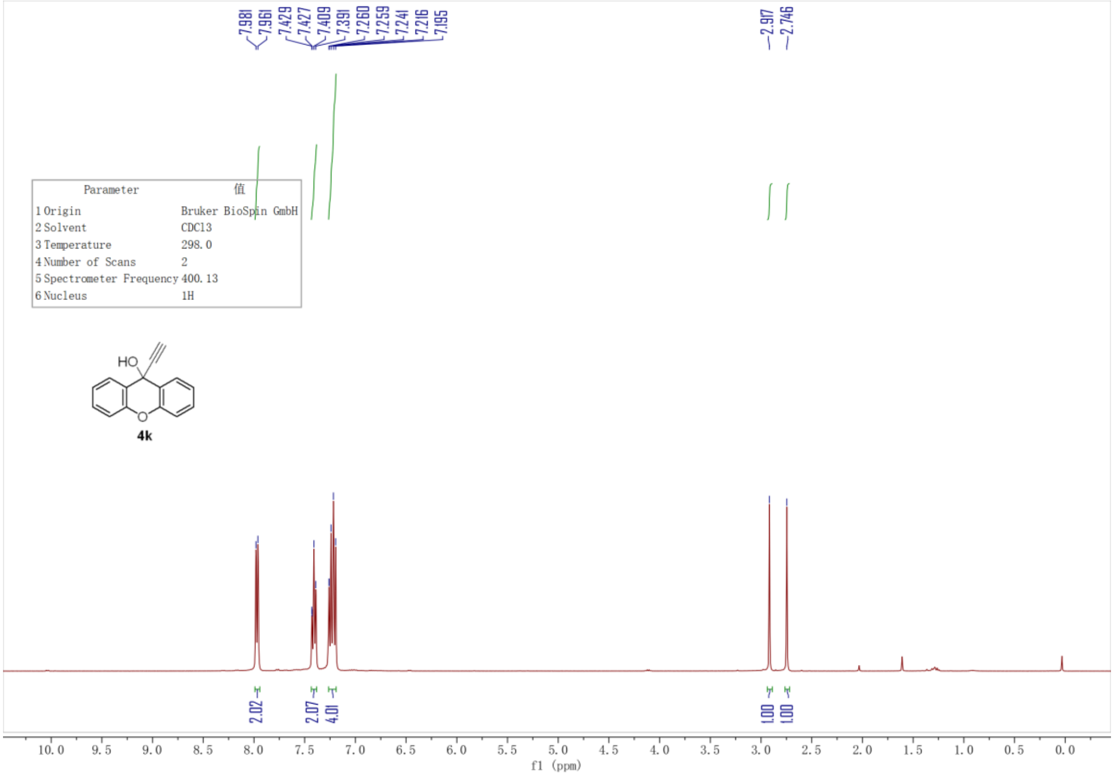
7.981
7.961
7.429
7.427
7.409
7.391
7.260
7.259
7.241
7.216
7.195
2.917
2.746
Parameter 值
1 Origin Bruker BioSpin GmbH
2 Solvent CDCl3
3 Temperature 298.0
4 Number of Scans 2
5 Spectrometer Frequency 400.13
6 Nucleus 1H
HO
O
4k
2.02
2.07
4.01
1.00
1.00
10.0 9.5 9.0 8.5 8.0 7.5 7.0 6.5 6.0 5.5 5.0 4.5 4.0 3.5 3.0 2.5 2.0 1.5 1.0 0.5 0.0
f1 (ppm)

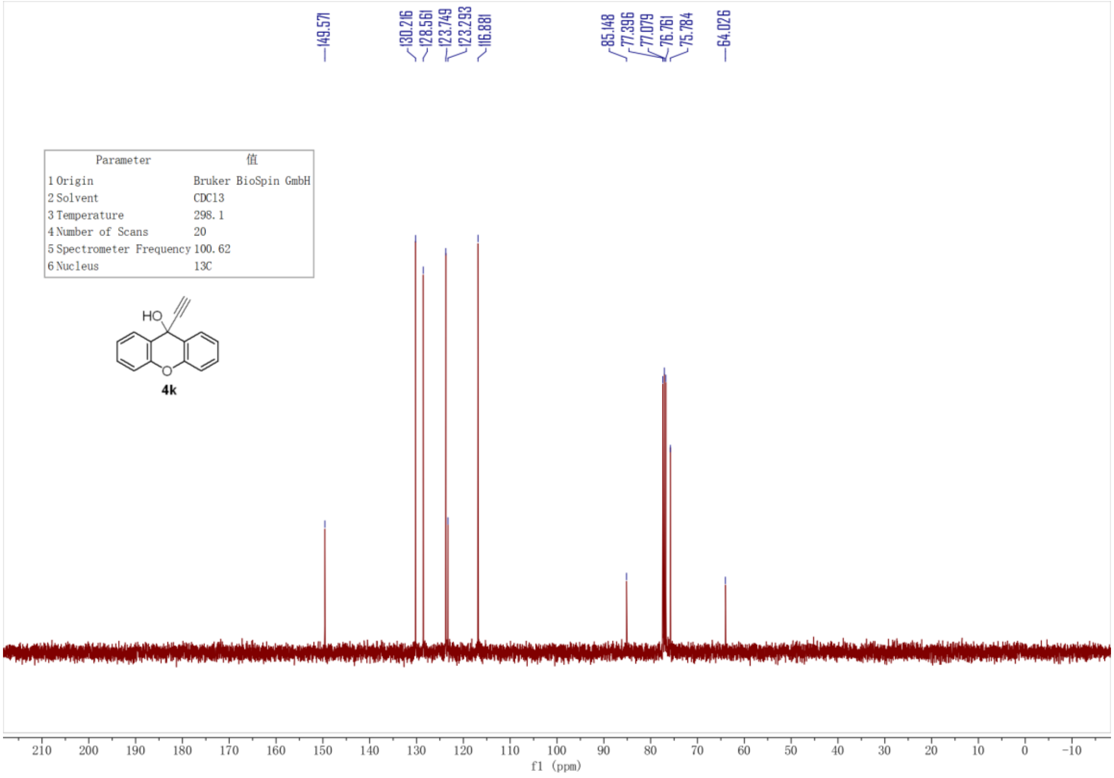
149.571
130.216
128.561
123.749
123.293
116.881
85.148
77.396
77.079
76.761
75.784
64.026
Parameter 值
1 Origin Bruker BioSpin GmbH
2 Solvent CDCl3
3 Temperature 298.1
4 Number of Scans 20
5 Spectrometer Frequency 100.62
6 Nucleus 13C
HO
O
4k
210 200 190 180 170 160 150 140 130 120 110 100 90 80 70 60 50 40 30 20 10 0 -10
f1 (ppm)

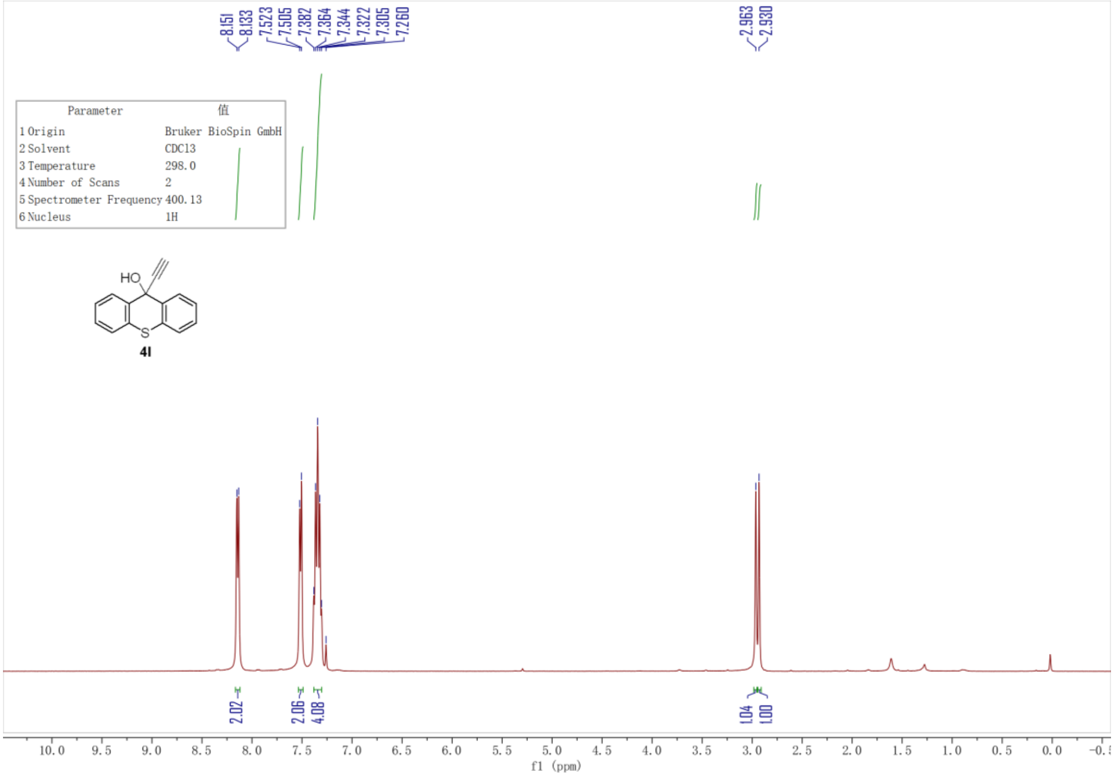
8.151
8.133
7.523
7.505
7.382
7.364
7.344
7.322
7.305
7.260
2.963
2.930
Parameter 值
1 Origin Bruker BioSpin GmbH
2 Solvent CDCl3
3 Temperature 298.0
4 Number of Scans 2
5 Spectrometer Frequency 400.13
6 Nucleus 1H
HO
S
4l
2.02
2.06
4.08
1.04
1.00
f1 (ppm)

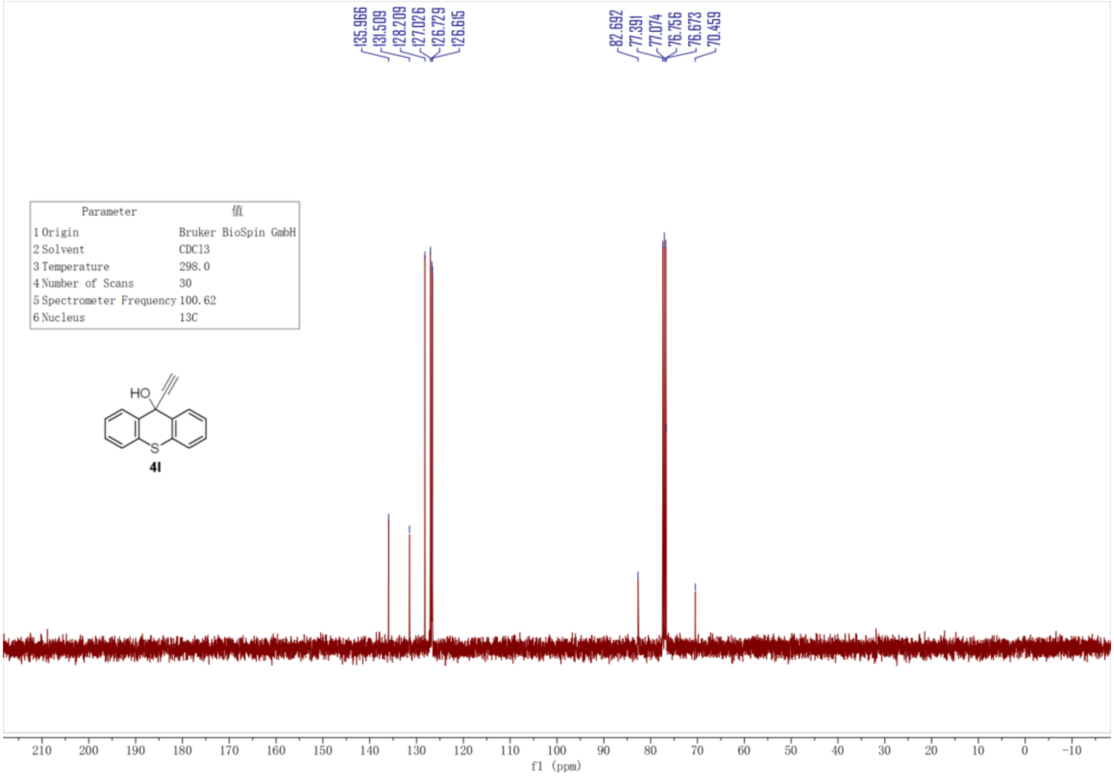
135.966
131.509
128.209
127.026
126.729
126.615
82.692
77.391
77.074
76.756
76.673
70.459
Parameter 值
1 Origin Bruker BioSpin GmbH
2 Solvent CDCl3
3 Temperature 298.0
4 Number of Scans 30
5 Spectrometer Frequency 100.62
6 Nucleus 13C
HO
S
4l
f1 (ppm)

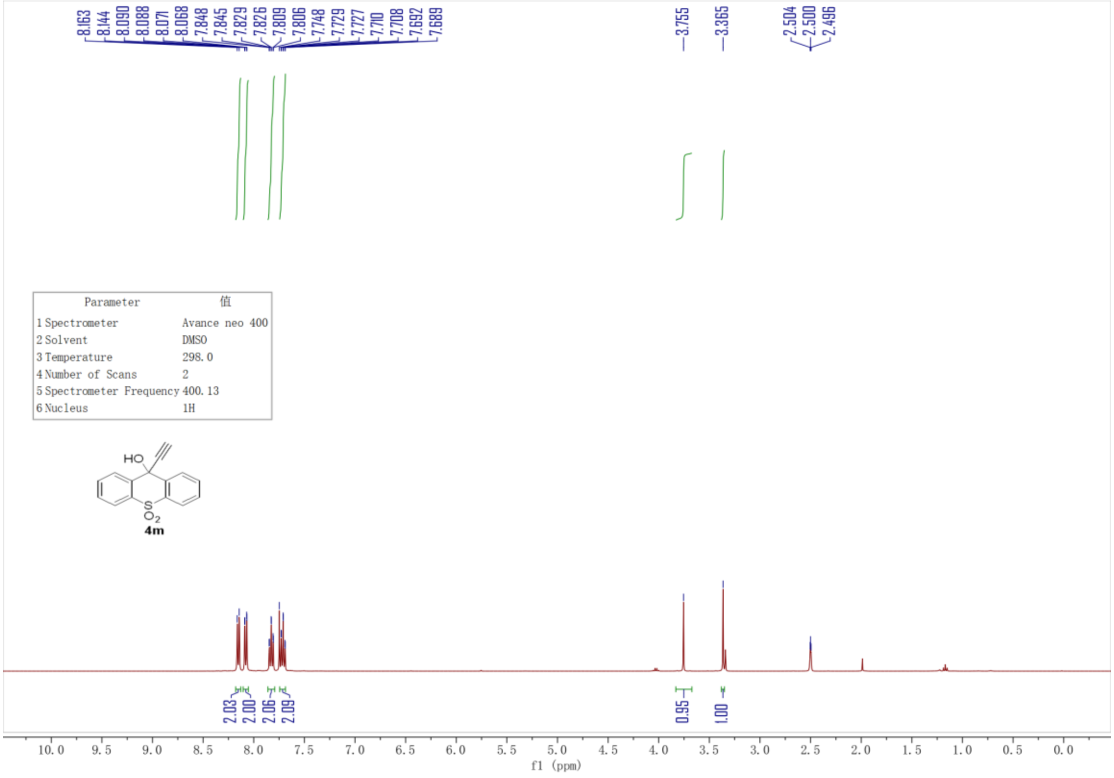

Parameter	值
1 Spectrometer	Avance neo 400
2 Solvent	DMSO
3 Temperature	298.0
4 Number of Scans	2
5 Spectrometer Frequency	400.13
6 Nucleus	1H

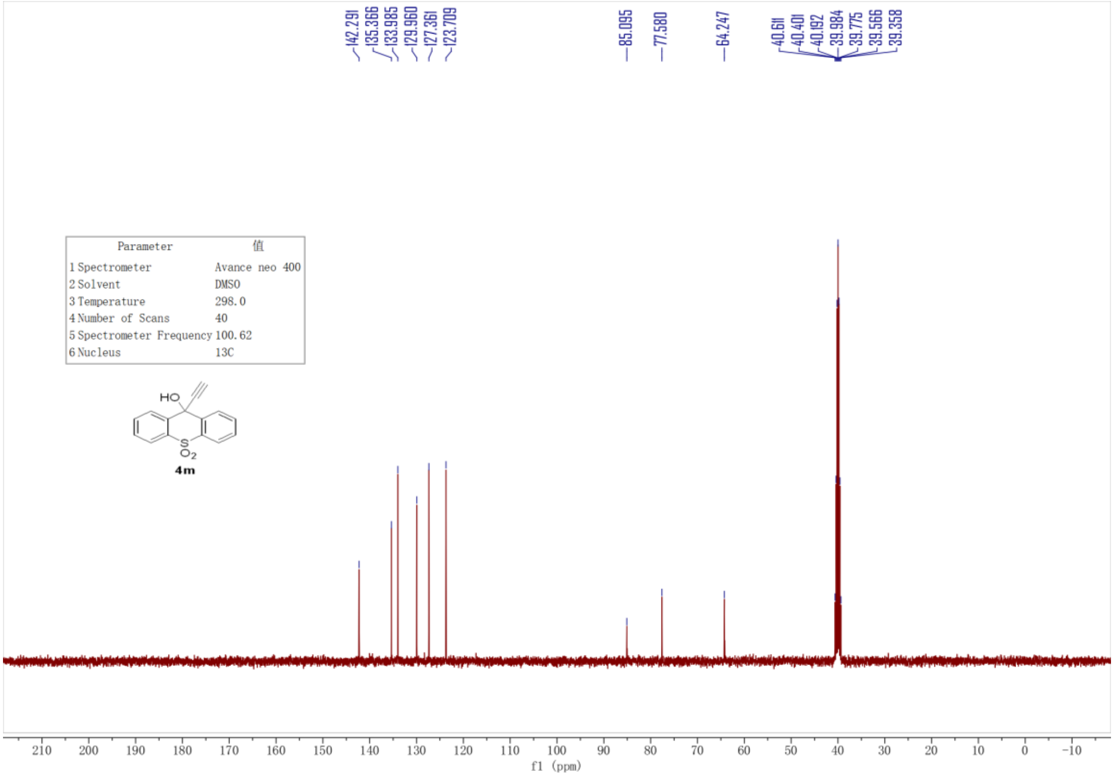

Parameter	值
1 Spectrometer	Avance neo 400
2 Solvent	DMSO
3 Temperature	298.0
4 Number of Scans	40
5 Spectrometer Frequency	100.62
6 Nucleus	13C

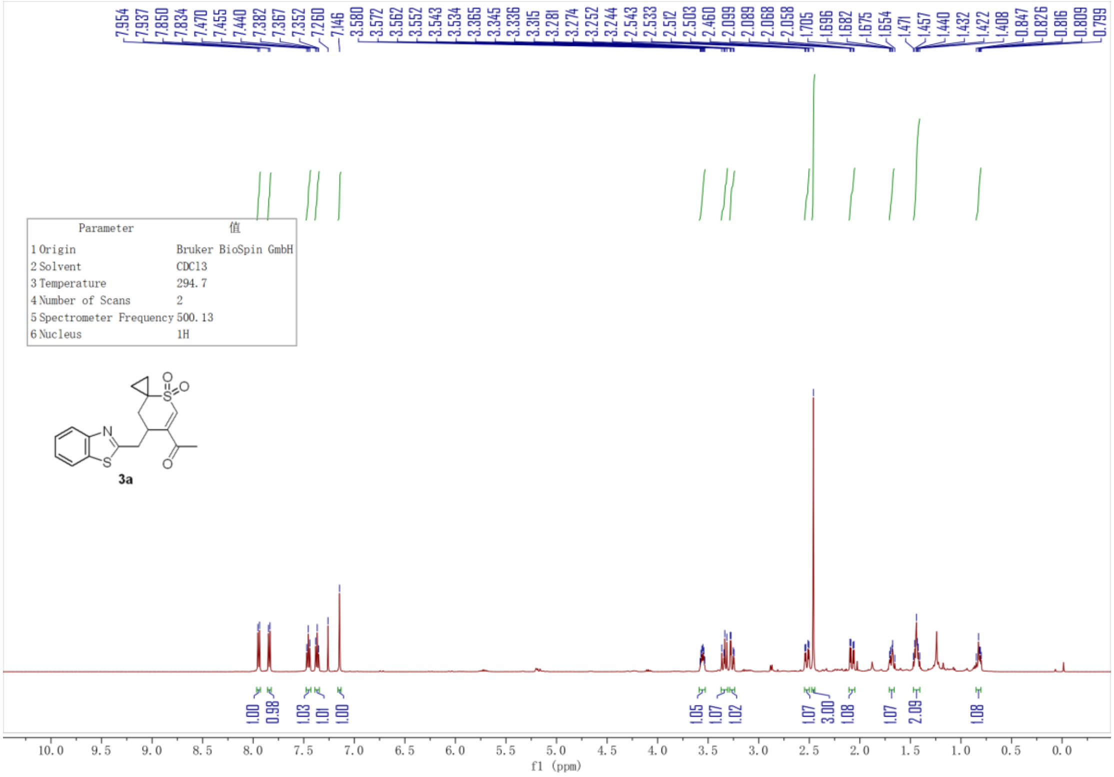
Parameter 值
1 Origin Bruker BioSpin GmbH
2 Solvent CDCl3
3 Temperature 294.7
4 Number of Scans 2
5 Spectrometer Frequency 500.13
6 Nucleus 1H
3a
f1 (ppm)

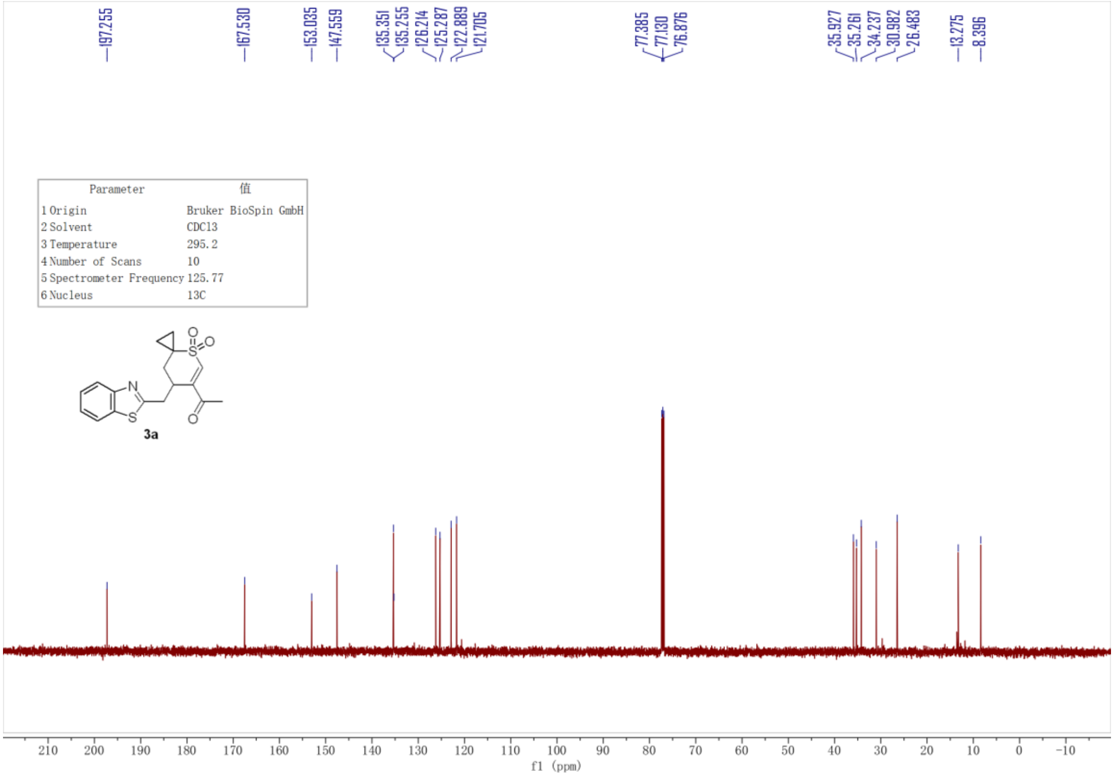
Parameter 值
1 Origin Bruker BioSpin GmbH
2 Solvent CDCl3
3 Temperature 295.2
4 Number of Scans 10
5 Spectrometer Frequency 125.77
6 Nucleus 13C
3a
f1 (ppm)

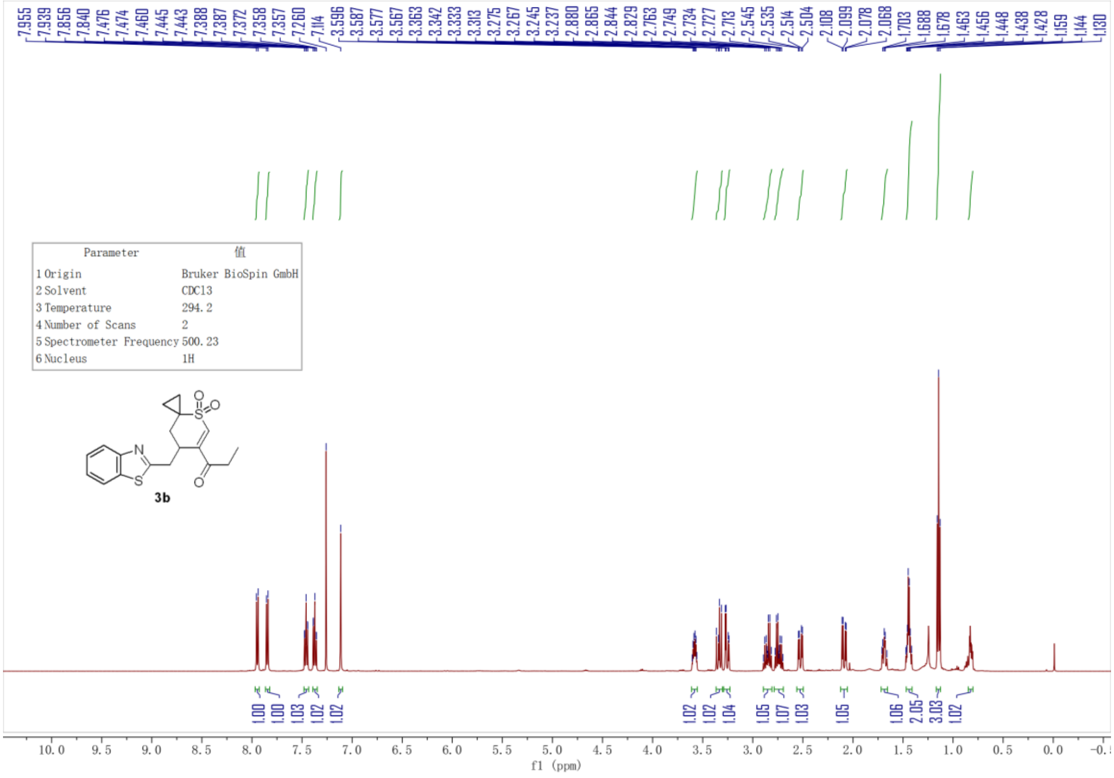
7.955
7.939
7.856
7.840
7.476
7.474
7.460
7.445
7.443
7.388
7.387
7.372
7.358
7.357
7.260
7.114
3.596
3.587
3.577
3.567
3.363
3.342
3.333
3.313
3.275
3.267
3.245
3.237
2.880
2.865
2.844
2.829
2.763
2.749
2.734
2.727
2.713
2.545
2.535
2.514
2.504
2.108
2.099
2.078
2.068
1.703
1.688
1.678
1.463
1.456
1.448
1.438
1.428
1.159
1.144
1.130
Parameter 值
1 Origin Bruker BioSpin GmbH
2 Solvent CDCl3
3 Temperature 294.2
4 Number of Scans 2
5 Spectrometer Frequency 500.23
6 Nucleus 1H
N
S
O
O
S
O
3b
1.00
1.00
1.03
1.02
1.02
1.02
1.02
1.04
1.05
1.07
1.03
1.05
1.06
2.05
3.03
1.02
10.0
9.5
9.0
8.5
8.0
7.5
7.0
6.5
6.0
5.5
5.0
4.5
4.0
3.5
3.0
2.5
2.0
1.5
1.0
0.5
0.0
f1 (ppm)

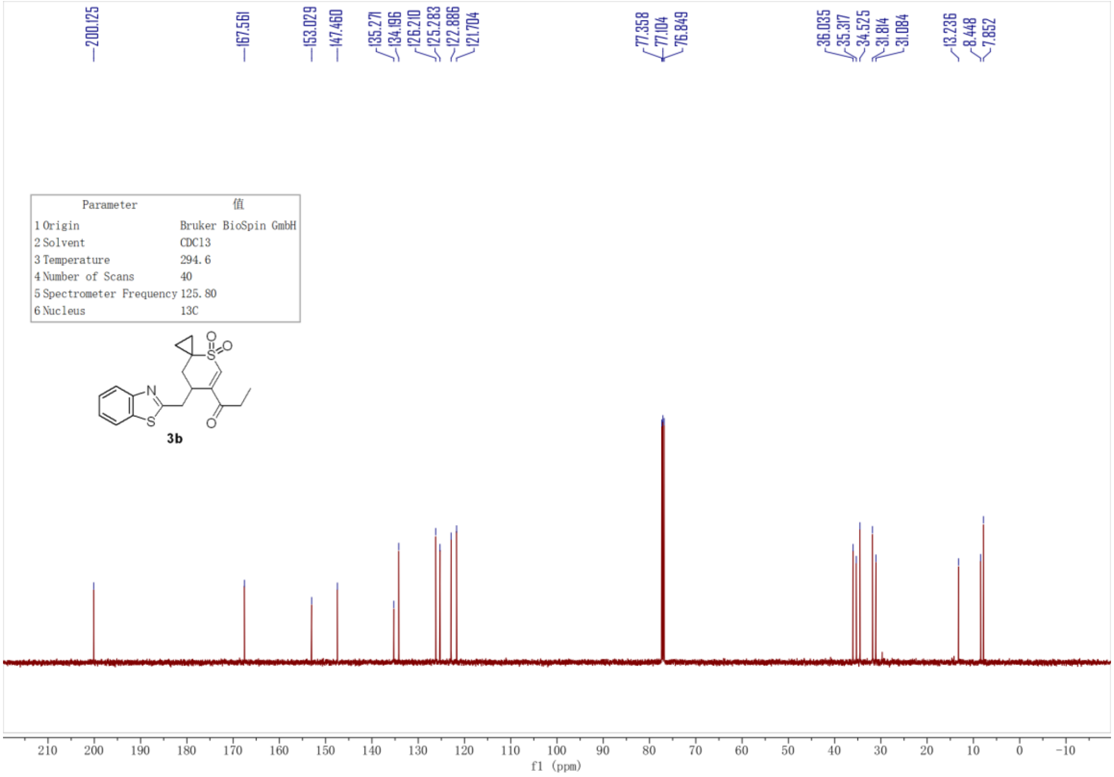
200.125
167.561
153.029
147.460
135.271
134.196
126.210
125.283
122.886
121.704
77.358
77.104
76.849
36.035
35.317
34.525
31.814
31.084
13.236
8.448
7.852
Parameter 值
1 Origin Bruker BioSpin GmbH
2 Solvent CDCl3
3 Temperature 294.6
4 Number of Scans 40
5 Spectrometer Frequency 125.80
6 Nucleus 13C
N
S
O
O
S
O
3b
210
200
190
180
170
160
150
140
130
120
110
100
90
80
70
60
50
40
30
20
10
0
-10
f1 (ppm)

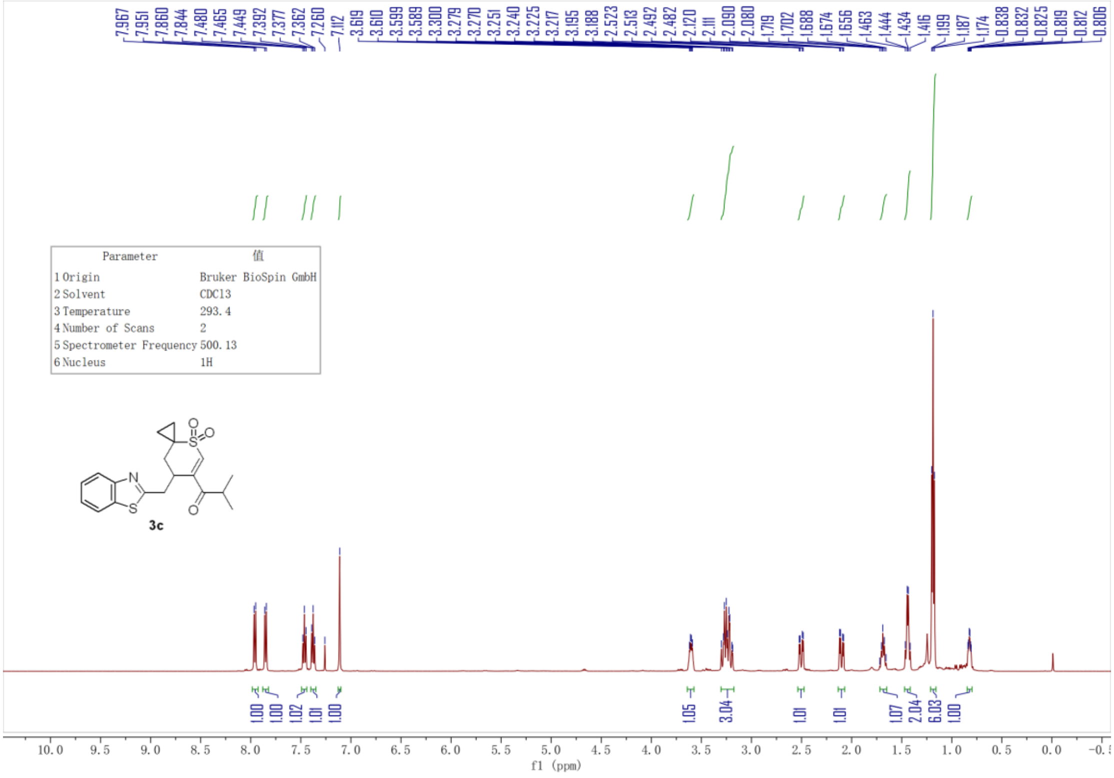

Parameter
值
1 Origin
Bruker BioSpin GmbH
2 Solvent
CDCl3
3 Temperature
293.4
4 Number of Scans
2
5 Spectrometer Frequency
500.13
6 Nucleus
1H
3c
f1 (ppm)

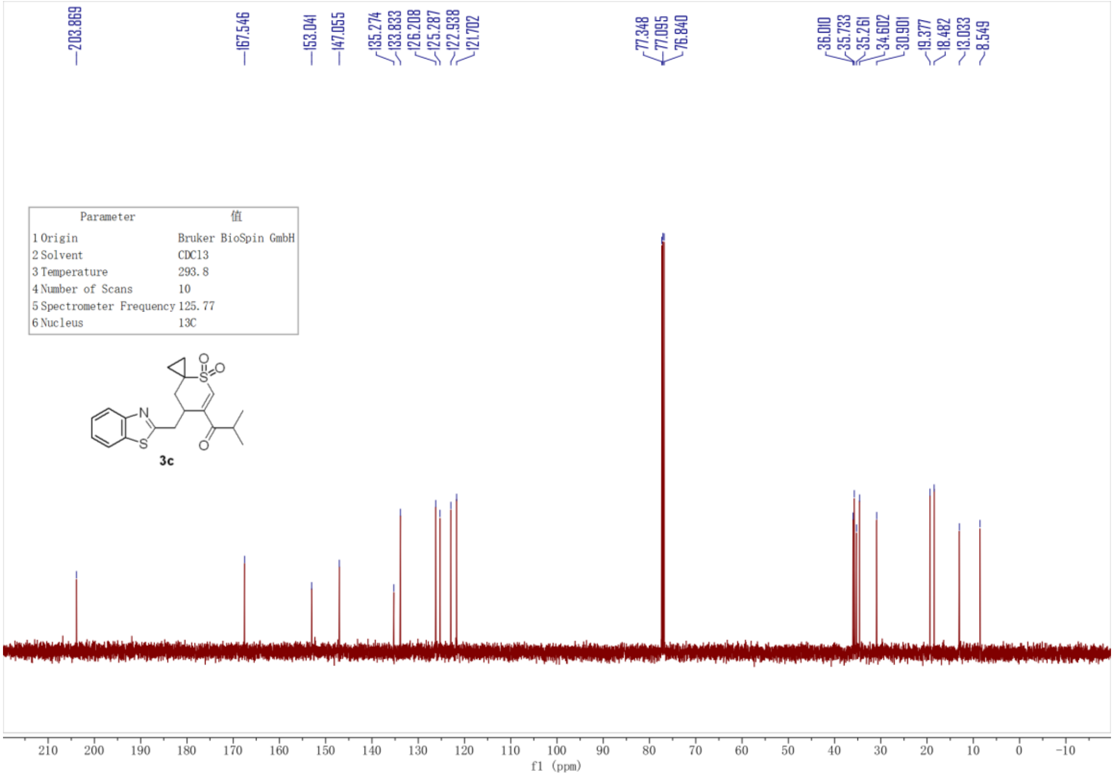

Parameter
值
1 Origin
Bruker BioSpin GmbH
2 Solvent
CDCl3
3 Temperature
293.8
4 Number of Scans
10
5 Spectrometer Frequency
125.77
6 Nucleus
13C
3c
f1 (ppm)

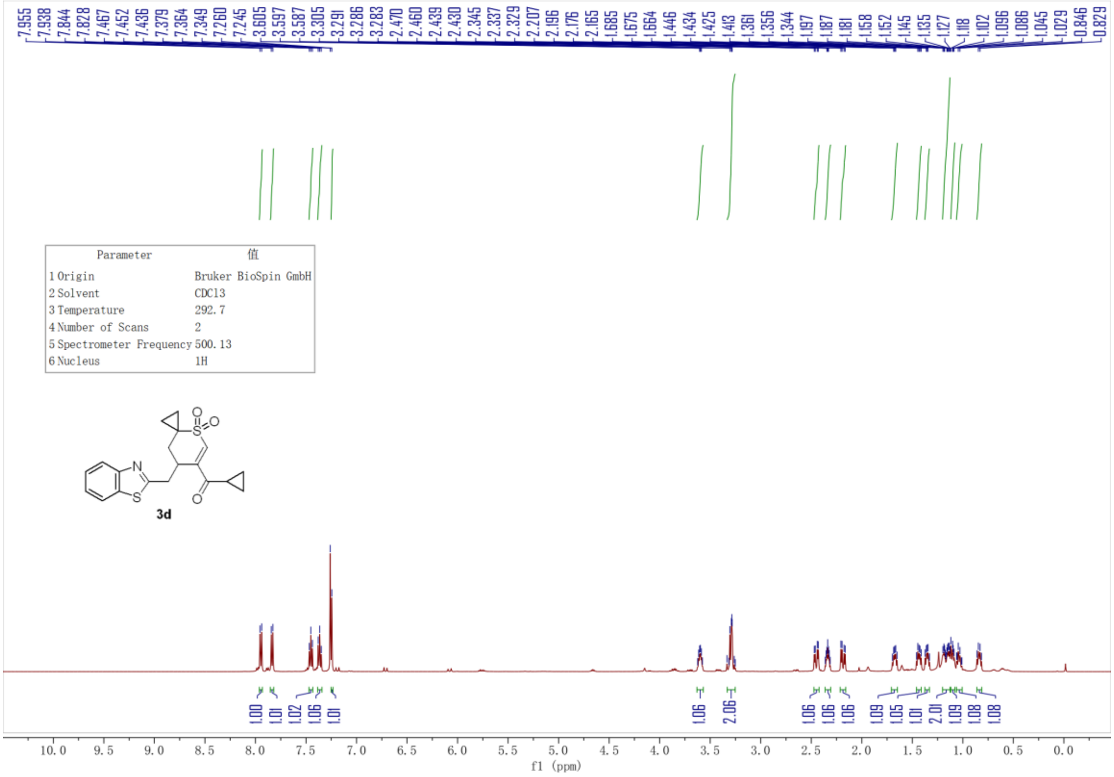

Parameter 值
1 Origin Bruker BioSpin GmbH
2 Solvent CDCl3
3 Temperature 292.7
4 Number of Scans 2
5 Spectrometer Frequency 500.13
6 Nucleus 1H
3d
f1 (ppm)

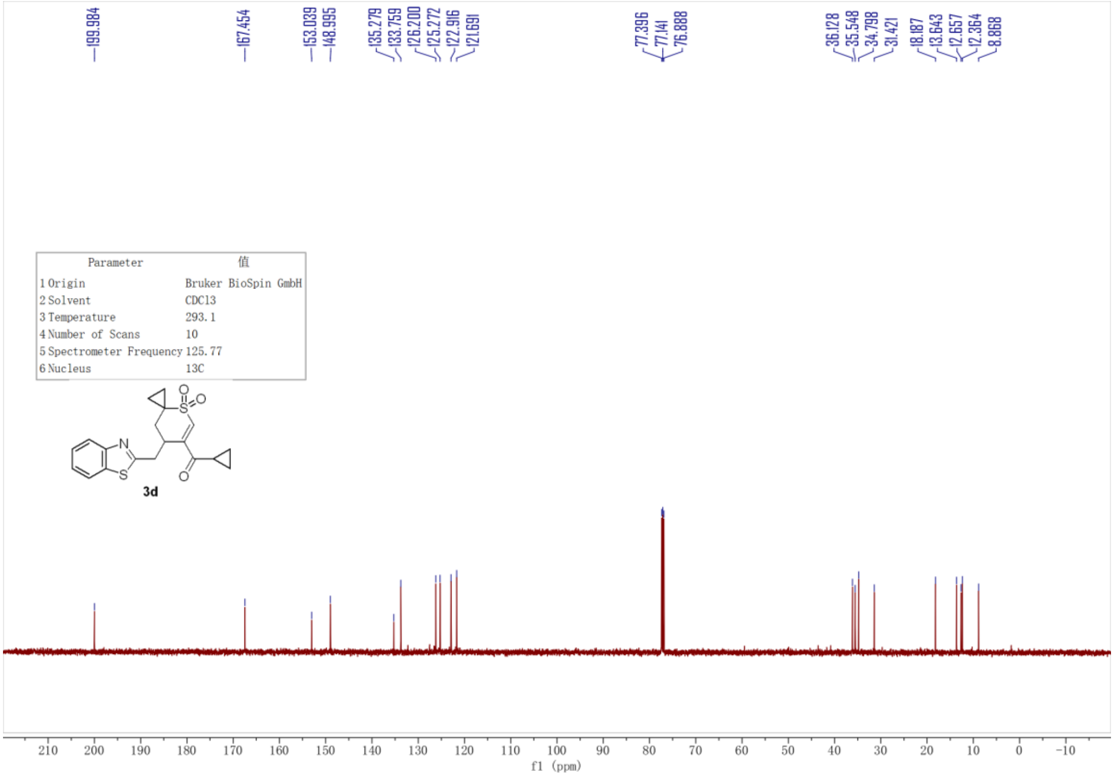

Parameter 值
1 Origin Bruker BioSpin GmbH
2 Solvent CDCl3
3 Temperature 293.1
4 Number of Scans 10
5 Spectrometer Frequency 125.77
6 Nucleus 13C
3d
f1 (ppm)

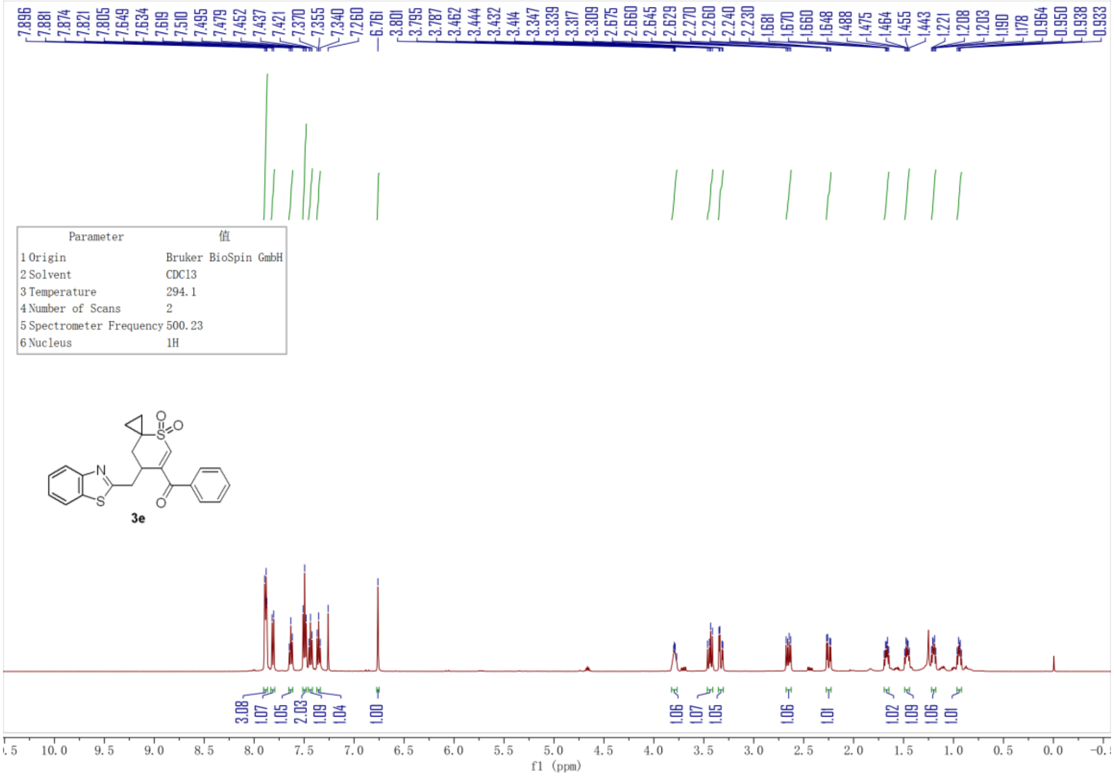

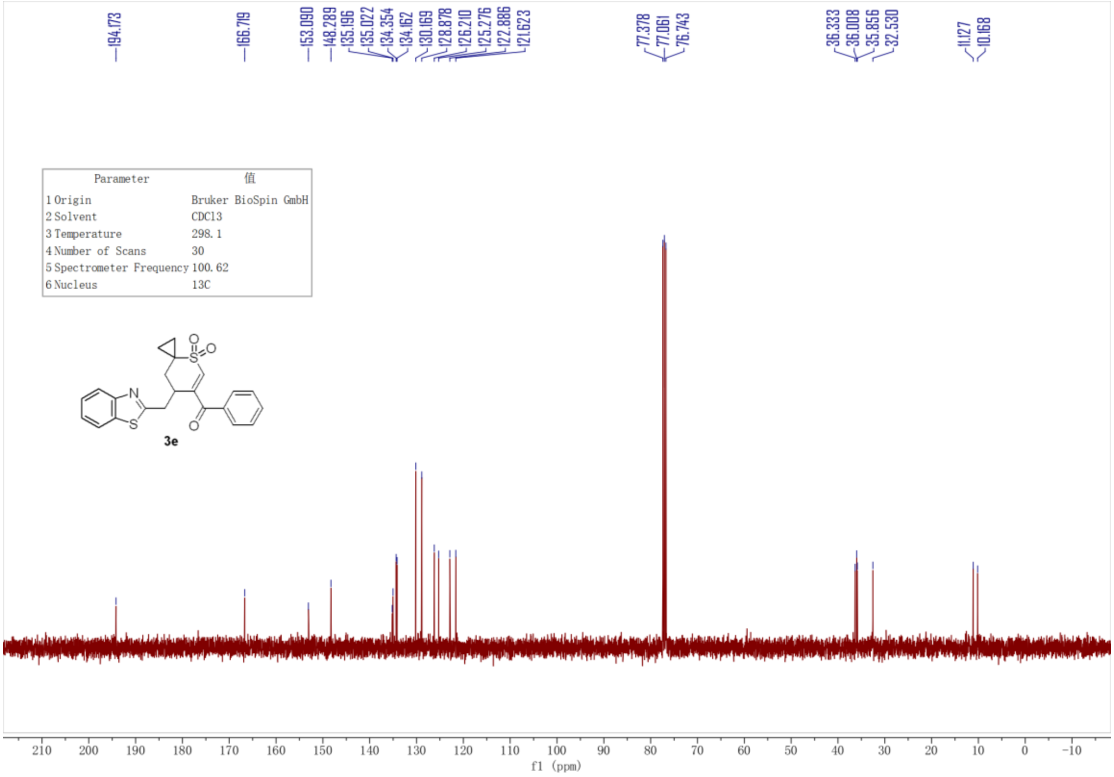

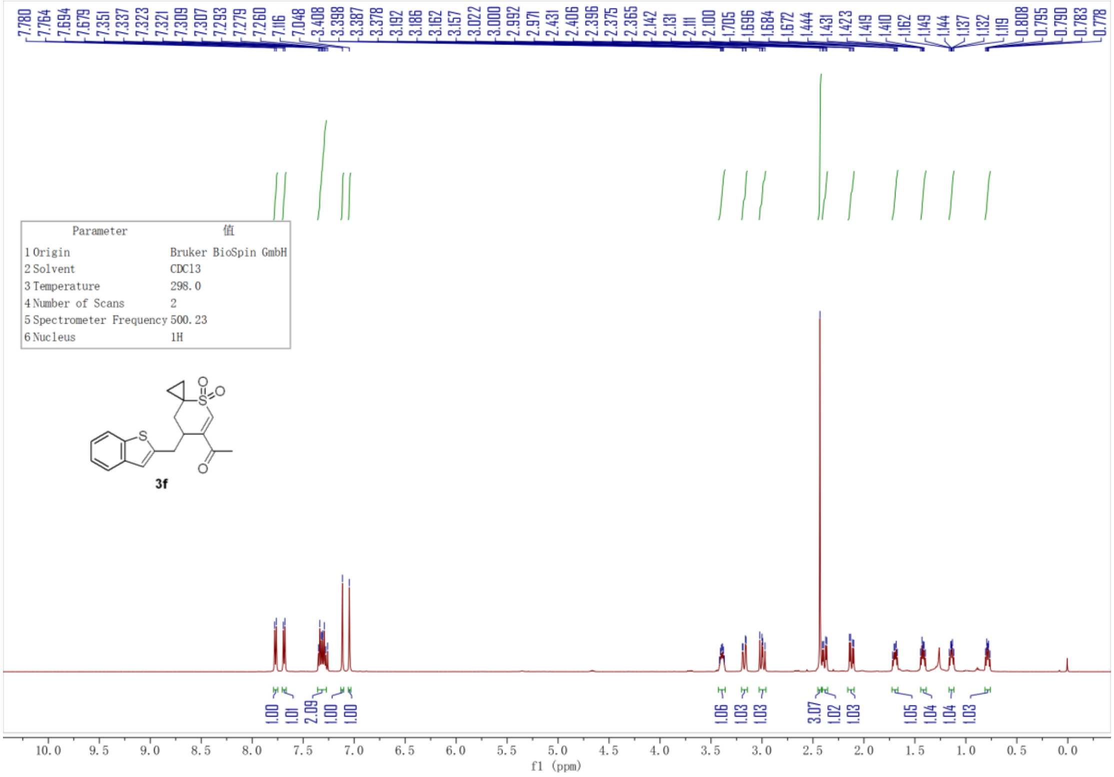
7.780 7.764 7.694 7.679 7.351 7.337 7.323 7.321 7.309 7.307 7.293 7.279 7.260 7.116 7.048 3.408 3.398 3.387 3.378 3.192 3.186 3.162 3.157 3.022 3.000 2.992 2.971 2.431 2.406 2.396 2.375 2.365 2.142 2.131 2.111 2.100 1.705 1.696 1.684 1.672 1.444 1.431 1.423 1.419 1.410 1.162 1.149 1.144 1.137 1.132 1.119 0.808 0.795 0.790 0.783 0.778
Parameter 值
1 Origin Bruker BioSpin GmbH
2 Solvent CDCl3
3 Temperature 298.0
4 Number of Scans 2
5 Spectrometer Frequency 500.23
6 Nucleus 1H
3f
1.00 1.01 2.09 1.00 1.00 1.06 1.03 1.03 3.07 1.02 1.03 1.05 1.04 1.04 1.03
10.0 9.5 9.0 8.5 8.0 7.5 7.0 6.5 6.0 5.5 5.0 4.5 4.0 3.5 3.0 2.5 2.0 1.5 1.0 0.5 0.0
f1 (ppm)

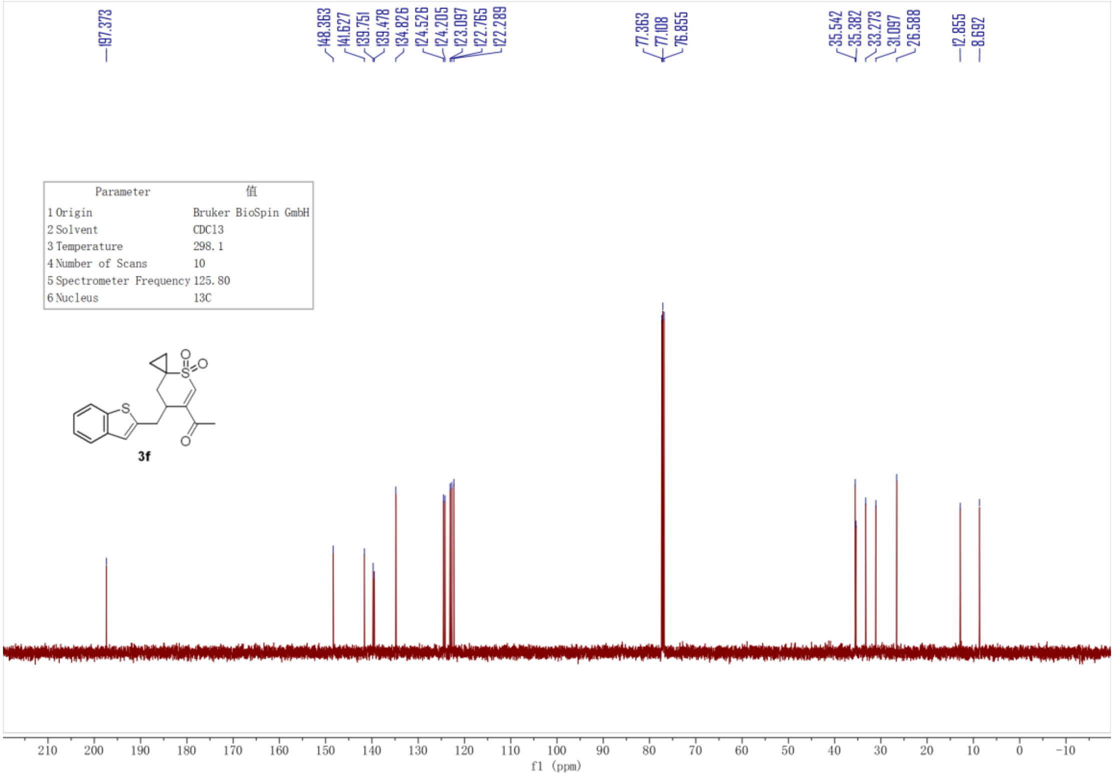
197.373
148.363 141.627 139.751 139.478 134.826 124.526 124.205 123.097 122.765 122.289
77.363 77.108 76.855
35.542 35.382 33.273 31.097 26.588 12.855 8.692
Parameter 值
1 Origin Bruker BioSpin GmbH
2 Solvent CDCl3
3 Temperature 298.1
4 Number of Scans 10
5 Spectrometer Frequency 125.80
6 Nucleus 13C
3f
210 200 190 180 170 160 150 140 130 120 110 100 90 80 70 60 50 40 30 20 10 0 -10
f1 (ppm)

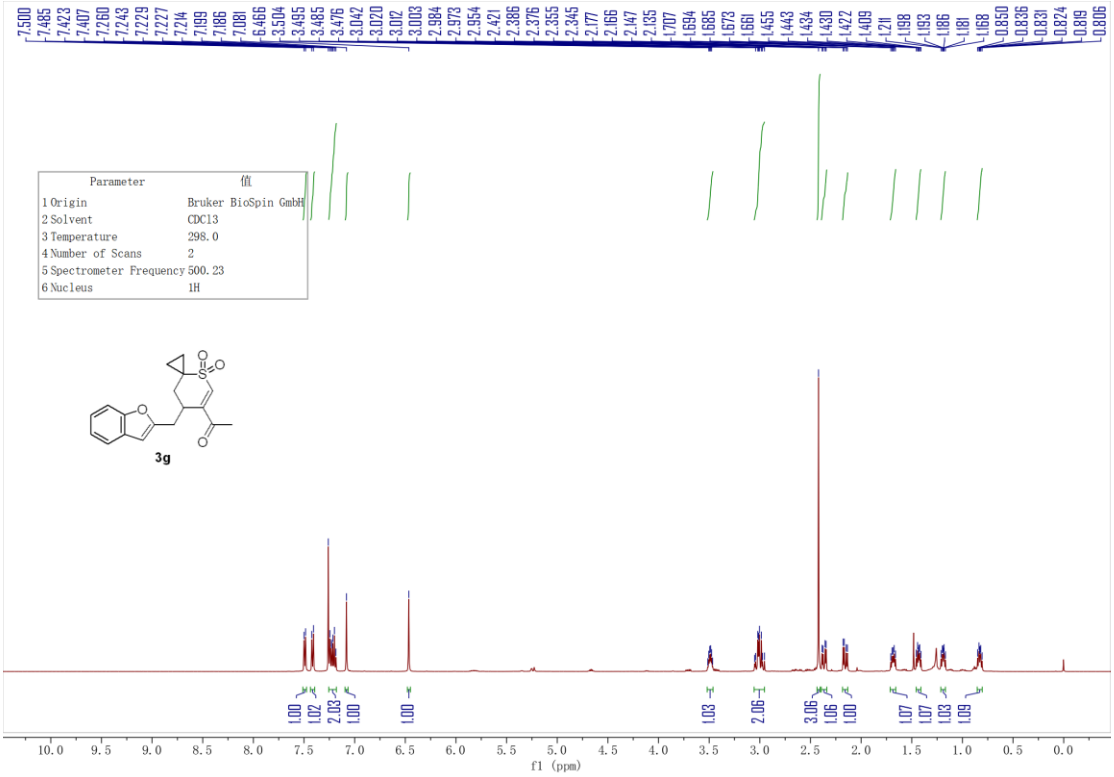
7.500 7.485 7.423 7.407 7.260 7.243 7.229 7.227 7.214 7.199 7.186 7.081 6.466 3.504 3.495 3.485 3.476 3.042 3.020 3.012 3.003 2.984 2.973 2.954 2.421 2.386 2.376 2.355 2.345 2.177 2.166 2.147 2.135 1.707 1.694 1.685 1.673 1.661 1.455 1.443 1.434 1.430 1.422 1.409 1.211 1.198 1.193 1.186 1.181 1.168 0.850 0.836 0.831 0.824 0.819 0.806
Parameter 值
1 Origin Bruker BioSpin GmbH
2 Solvent CDCl3
3 Temperature 298.0
4 Number of Scans 2
5 Spectrometer Frequency 500.23
6 Nucleus 1H
3g
1.00 1.02 2.03 1.00 1.00 1.03 2.06 3.06 1.06 1.00 1.07 1.07 1.03 1.09
10.0 9.5 9.0 8.5 8.0 7.5 7.0 6.5 6.0 5.5 5.0 4.5 4.0 3.5 3.0 2.5 2.0 1.5 1.0 0.5 0.0
f1 (ppm)

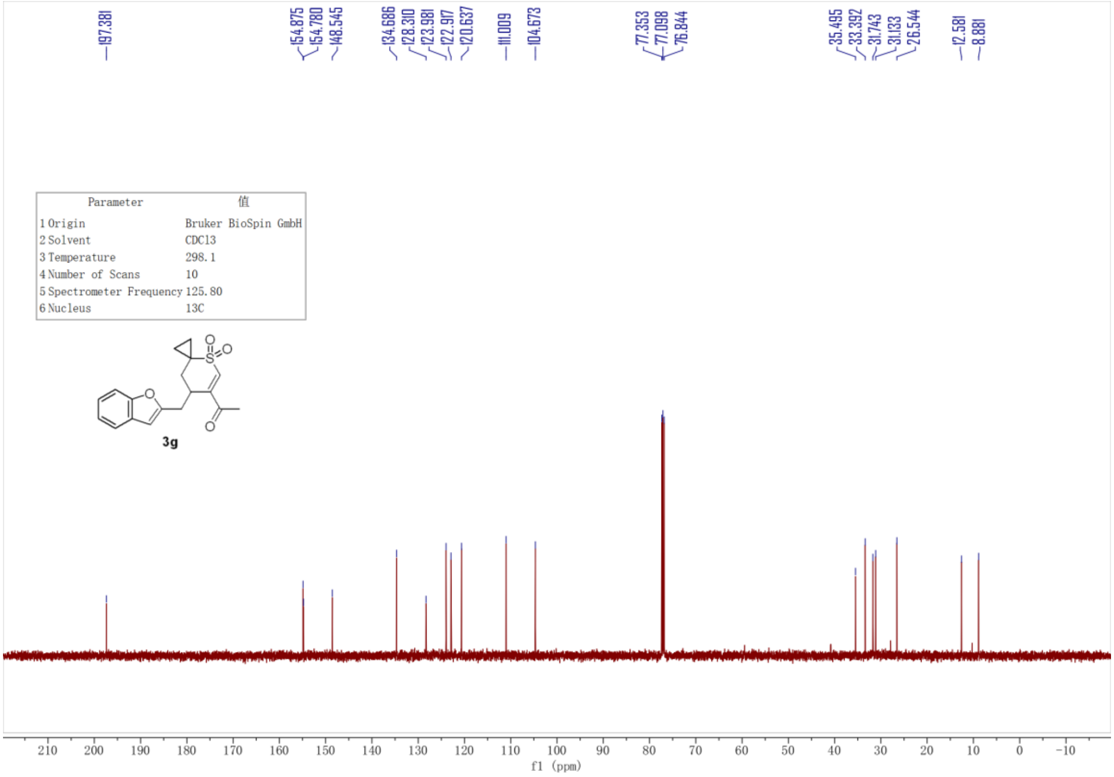
197.381 154.875 154.780 148.545 134.686 128.310 123.981 122.917 120.637 111.009 104.673 77.353 77.098 76.844 35.495 33.392 31.743 31.133 26.544 12.581 8.881
Parameter 值
1 Origin Bruker BioSpin GmbH
2 Solvent CDCl3
3 Temperature 298.1
4 Number of Scans 10
5 Spectrometer Frequency 125.80
6 Nucleus 13C
3g
210 200 190 180 170 160 150 140 130 120 110 100 90 80 70 60 50 40 30 20 10 0 -10
f1 (ppm)

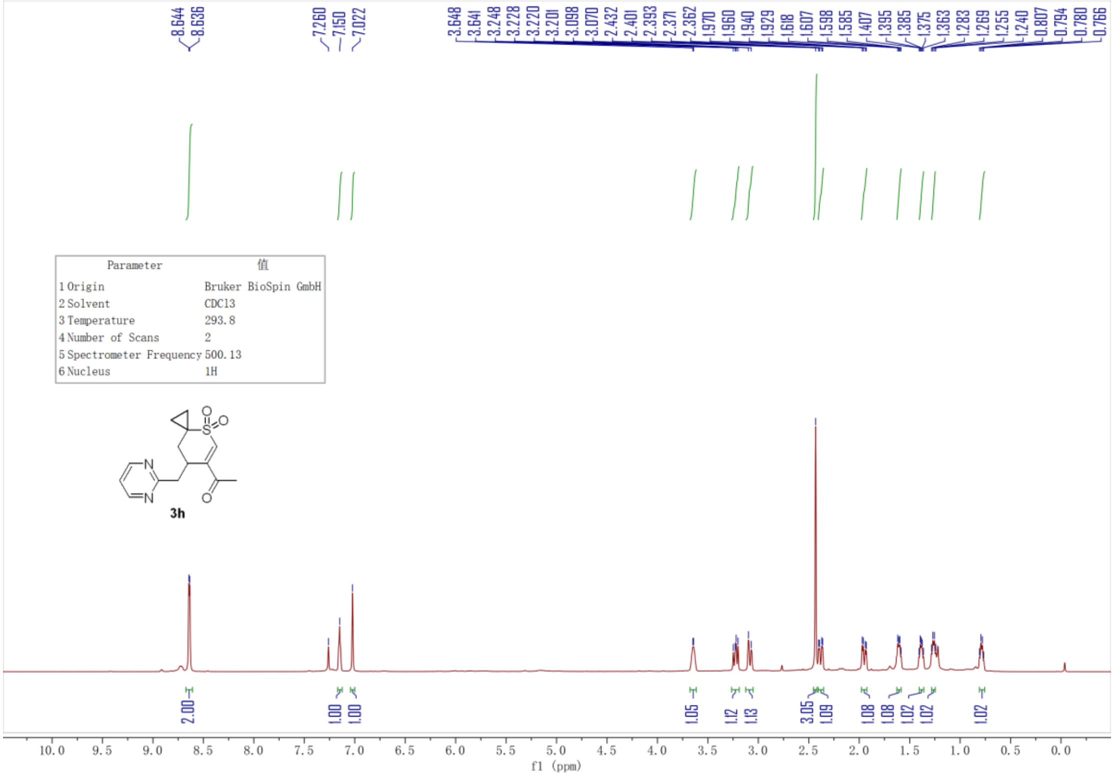
8.644
8.636
7.260
7.150
7.022
3.648
3.641
3.248
3.228
3.220
3.201
3.098
3.070
2.432
2.401
2.393
2.371
2.362
1.970
1.960
1.940
1.929
1.618
1.607
1.598
1.585
1.407
1.395
1.385
1.375
1.363
1.283
1.269
1.255
1.240
0.807
0.794
0.780
0.766
Parameter
值
1 Origin
Bruker BioSpin GmbH
2 Solvent
CDCl3
3 Temperature
293.8
4 Number of Scans
2
5 Spectrometer Frequency
500.13
6 Nucleus
1H
3h
2.00
1.00
1.00
1.05
1.12
1.13
3.05
1.09
1.08
1.08
1.02
1.02
1.02
f1 (ppm)

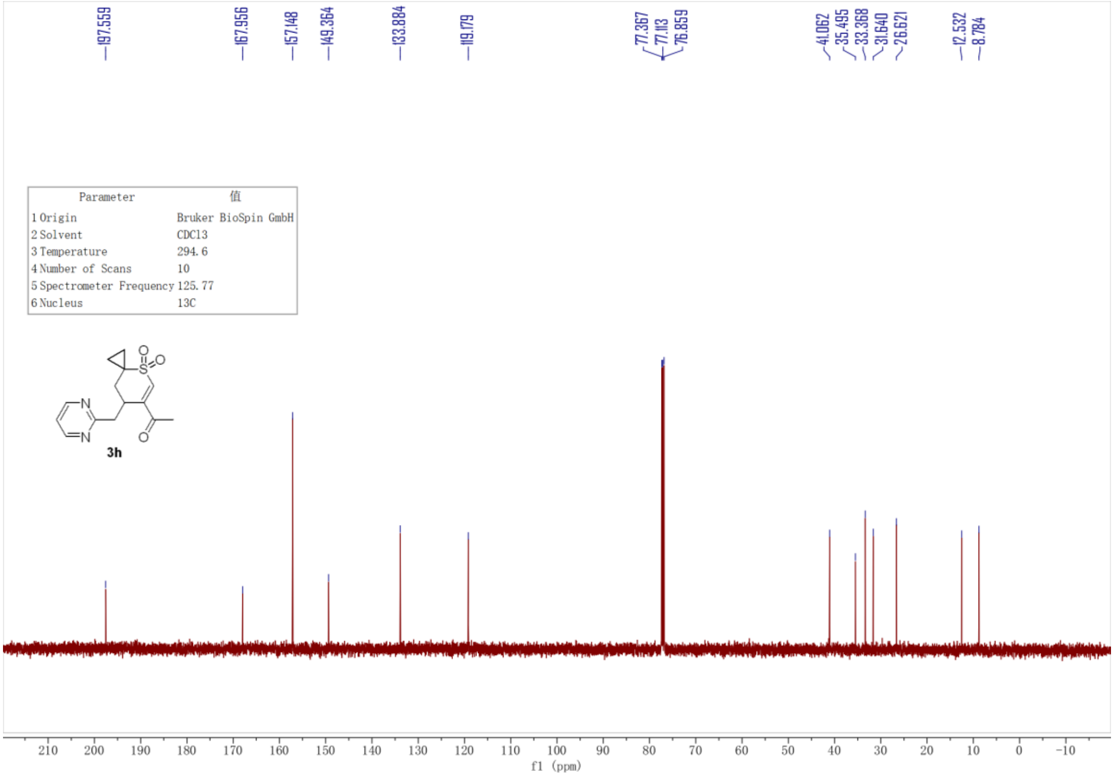
197.559
167.956
157.148
149.364
133.884
119.179
77.367
77.113
76.859
41.062
35.495
33.368
31.640
26.621
12.532
8.784
Parameter
值
1 Origin
Bruker BioSpin GmbH
2 Solvent
CDCl3
3 Temperature
294.6
4 Number of Scans
10
5 Spectrometer Frequency
125.77
6 Nucleus
13C
3h
f1 (ppm)

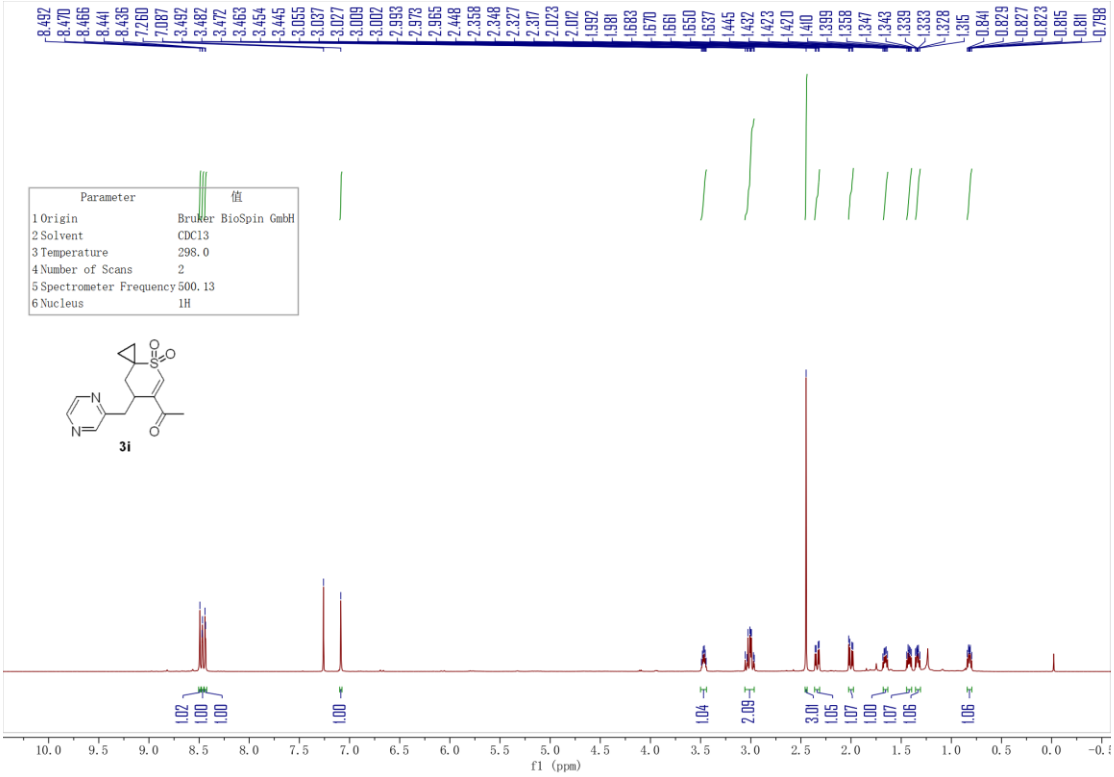
Parameter 值
1 Origin Bruker BioSpin GmbH
2 Solvent CDCl3
3 Temperature 298.0
4 Number of Scans 2
5 Spectrometer Frequency 500.13
6 Nucleus 1H
3i
f1 (ppm)

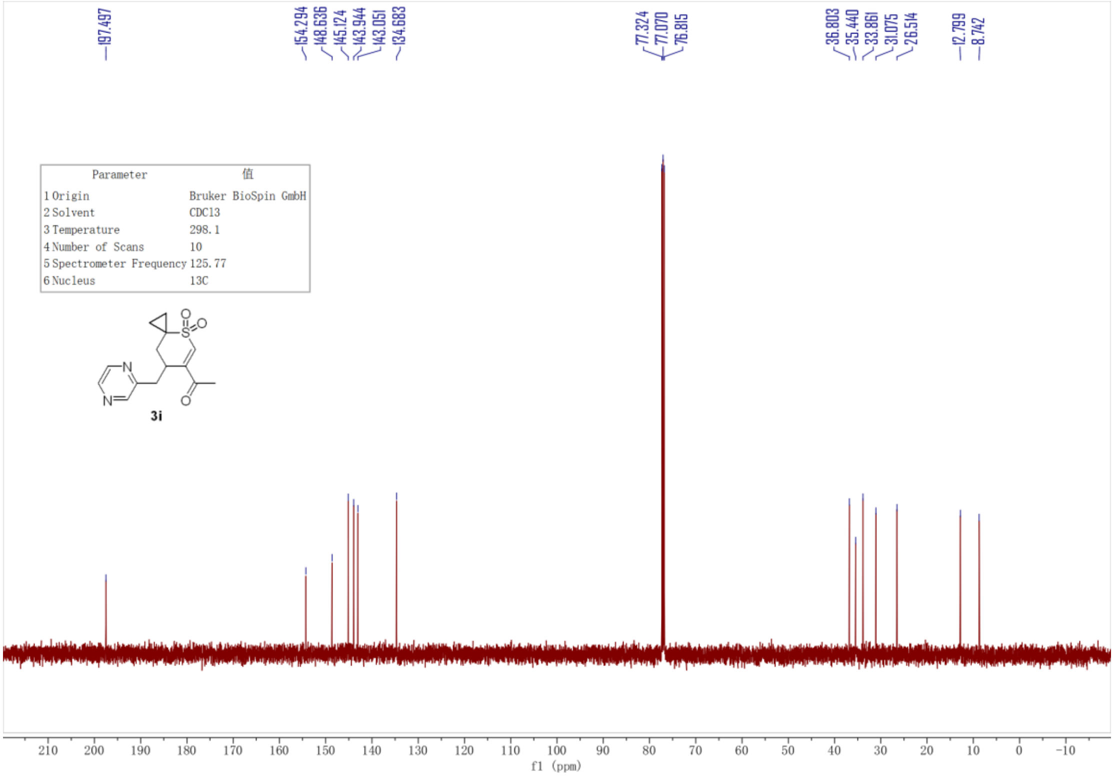
Parameter 值
1 Origin Bruker BioSpin GmbH
2 Solvent CDCl3
3 Temperature 298.1
4 Number of Scans 10
5 Spectrometer Frequency 125.77
6 Nucleus 13C
3i
f1 (ppm)

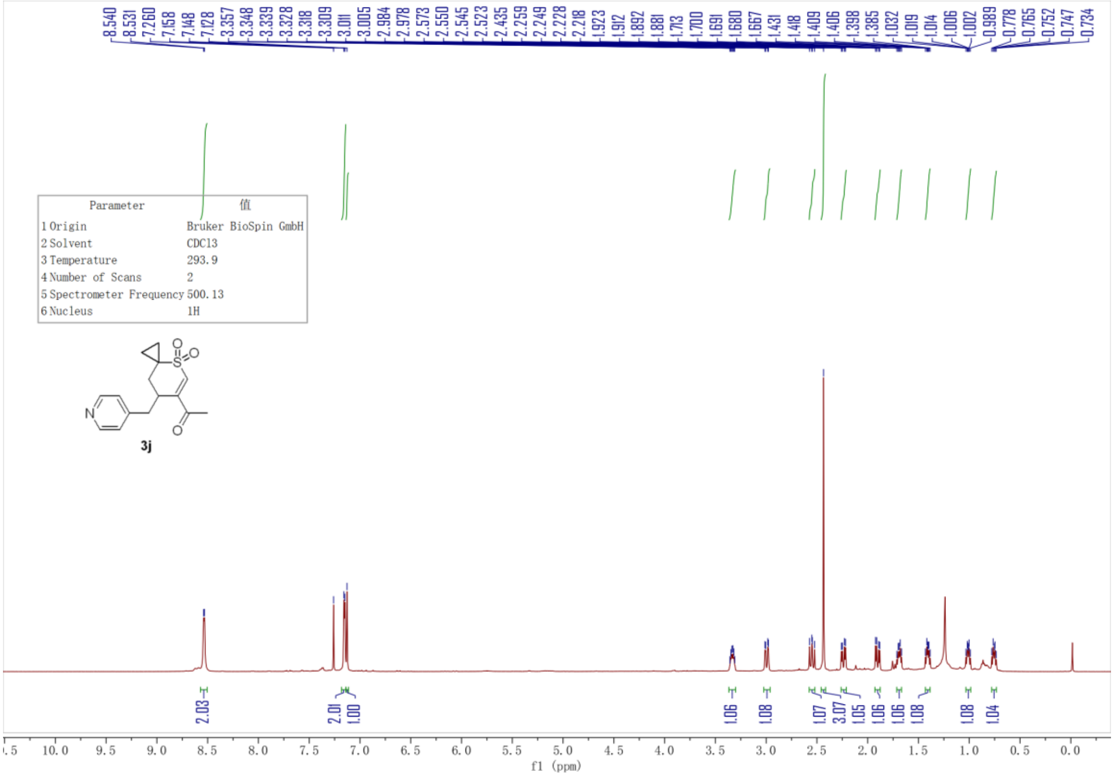

Parameter
值
1 Origin
Bruker BioSpin GmbH
2 Solvent
CDCl3
3 Temperature
293.9
4 Number of Scans
2
5 Spectrometer Frequency
500.13
6 Nucleus
1H
3j
f1 (ppm)

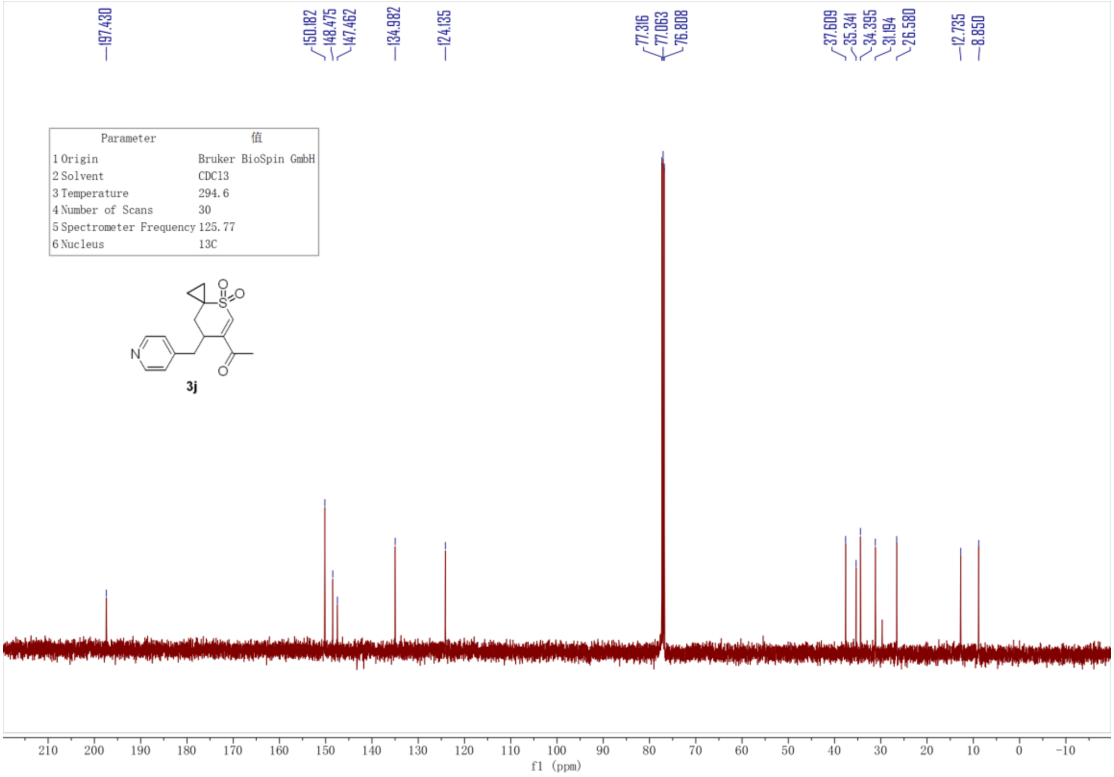

Parameter
值
1 Origin
Bruker BioSpin GmbH
2 Solvent
CDCl3
3 Temperature
294.6
4 Number of Scans
30
5 Spectrometer Frequency
125.77
6 Nucleus
13C
3j
197.430
150.182
148.475
147.462
134.982
124.135
77.316
77.063
76.808
37.609
35.341
34.395
31.194
26.580
12.735
8.850
f1 (ppm)

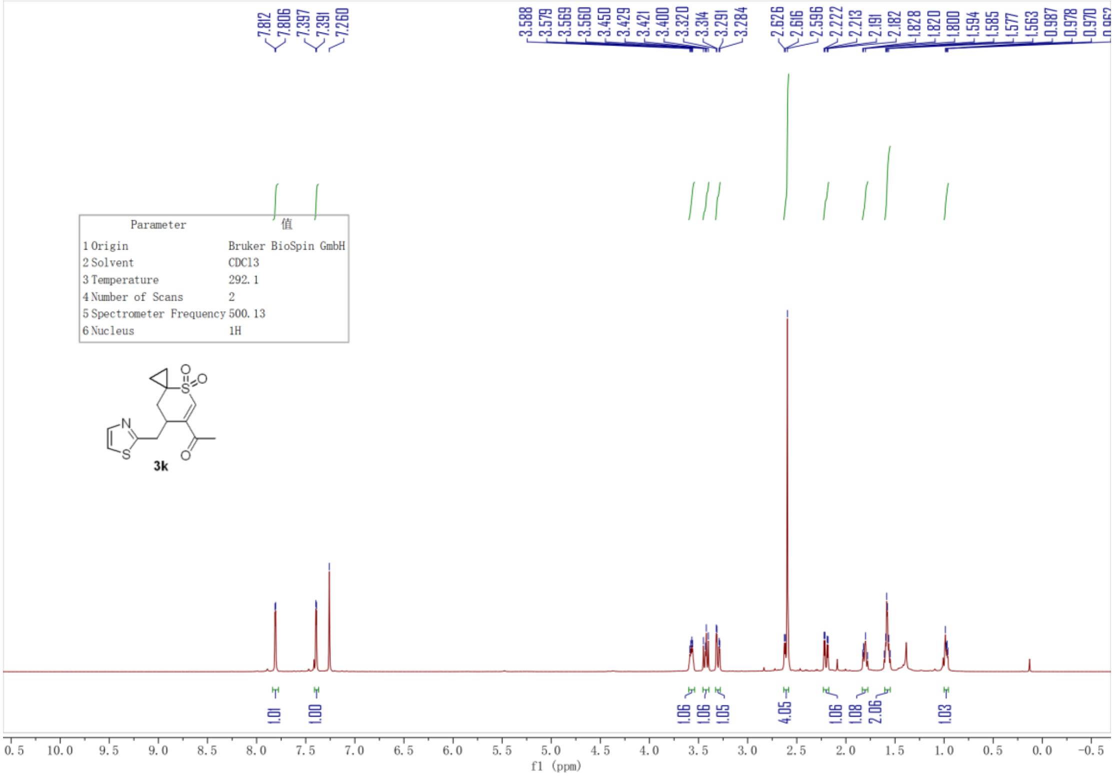

Parameter 值
1 Origin Bruker BioSpin GmbH
2 Solvent CDCl3
3 Temperature 292.1
4 Number of Scans 2
5 Spectrometer Frequency 500.13
6 Nucleus 1H
3k
f1 (ppm)

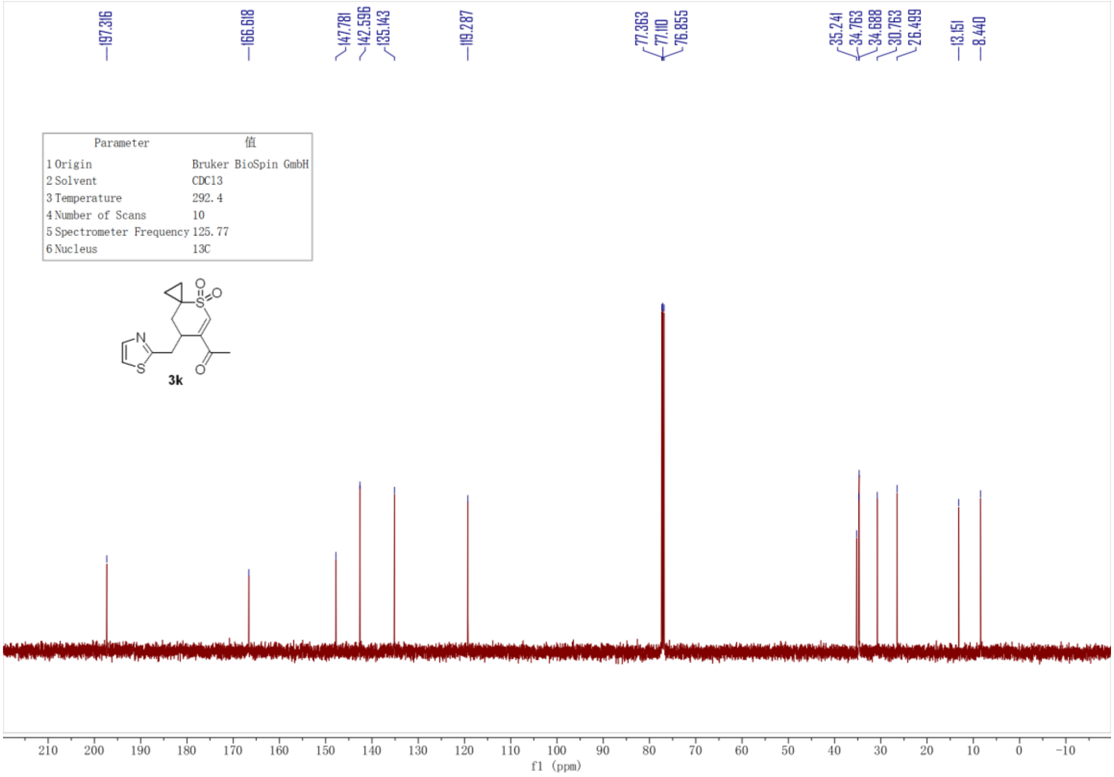

197.316
166.618
147.781
142.596
135.143
119.287
77.363
77.110
76.855
35.241
34.763
34.688
30.763
26.499
13.151
8.440
Parameter 值
1 Origin Bruker BioSpin GmbH
2 Solvent CDCl3
3 Temperature 292.4
4 Number of Scans 10
5 Spectrometer Frequency 125.77
6 Nucleus 13C
3k
f1 (ppm)

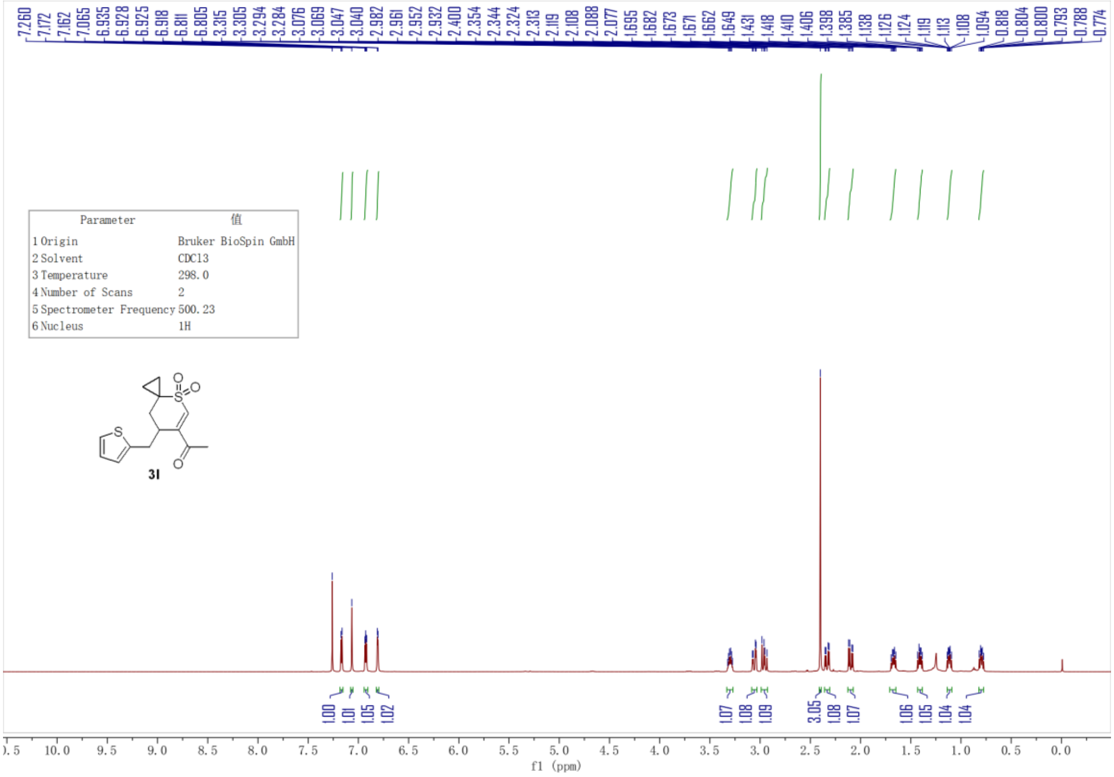
Parameter
值
1 Origin Bruker BioSpin GmbH
2 Solvent CDCl3
3 Temperature 298.0
4 Number of Scans 2
5 Spectrometer Frequency 500.23
6 Nucleus 1H
3l
f1 (ppm)

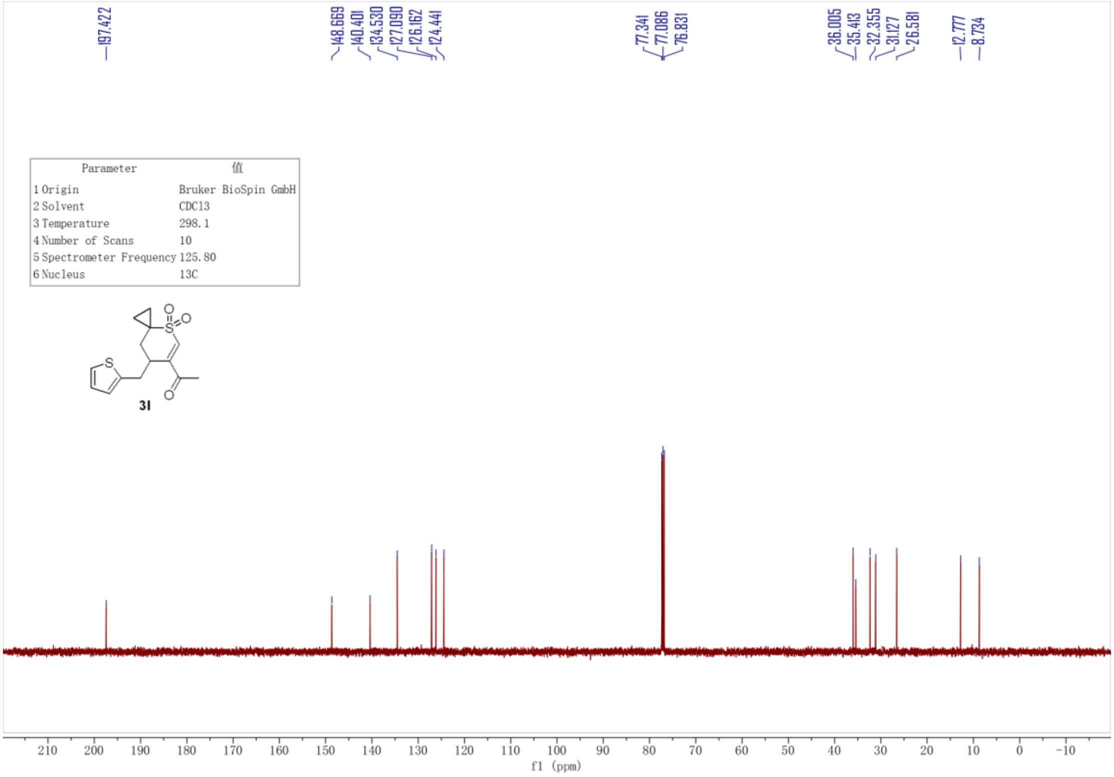
Parameter
值
1 Origin Bruker BioSpin GmbH
2 Solvent CDCl3
3 Temperature 298.1
4 Number of Scans 10
5 Spectrometer Frequency 125.80
6 Nucleus 13C
3l
f1 (ppm)

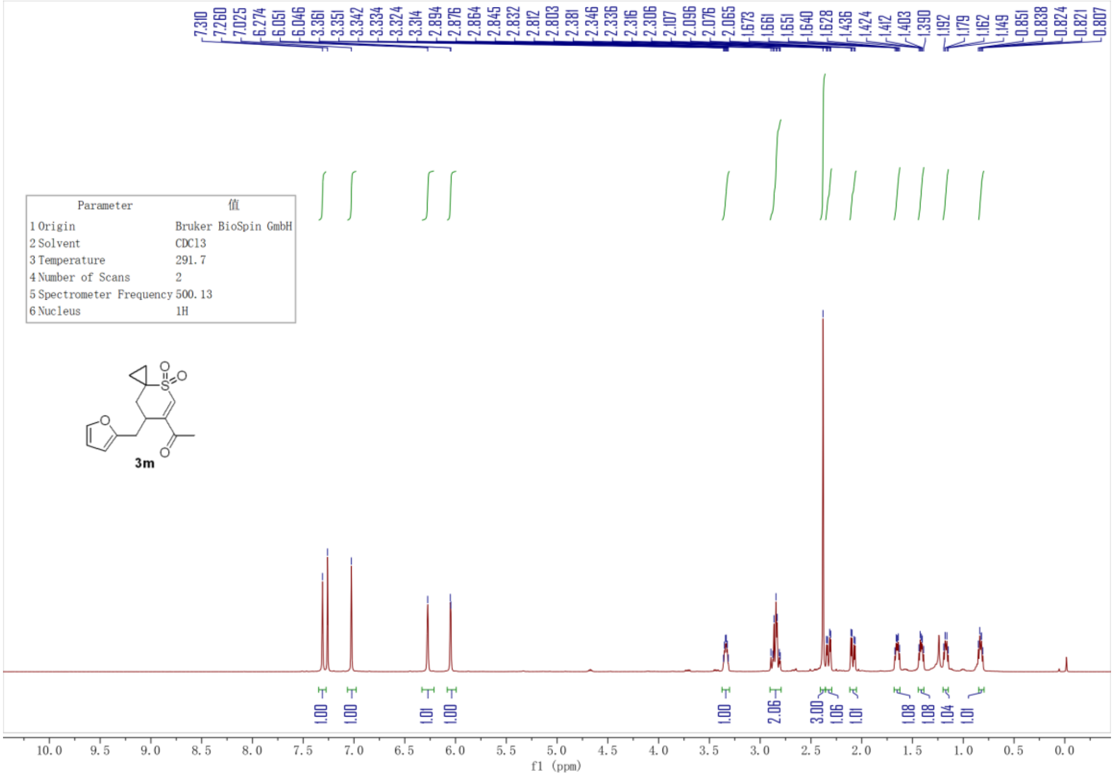
7.310 7.260 7.025 6.274 6.051 6.046 3.361 3.351 3.342 3.334 3.324 3.314 2.894 2.876 2.864 2.845 2.832 2.812 2.803 2.381 2.346 2.336 2.316 2.306 2.107 2.096 2.076 2.065 1.673 1.661 1.651 1.640 1.628 1.436 1.424 1.412 1.403 1.390 1.192 1.179 1.162 1.149 0.851 0.838 0.824 0.821 0.807
Parameter 值
1 Origin Bruker BioSpin GmbH
2 Solvent CDCl3
3 Temperature 291.7
4 Number of Scans 2
5 Spectrometer Frequency 500.13
6 Nucleus 1H
3m
1.00 1.00 1.01 1.00 1.00 2.06 3.00 1.06 1.01 1.08 1.08 1.04 1.01
f1 (ppm)

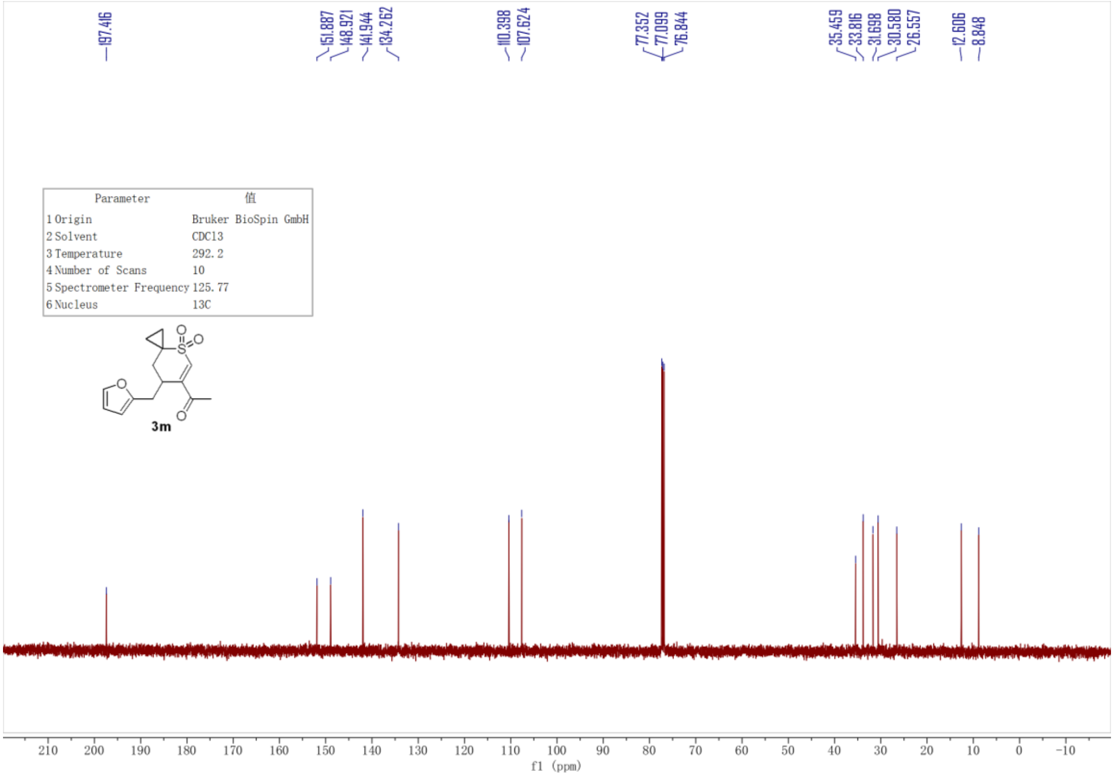
197.416
151.887 148.921 141.944 134.262
110.398 107.624
77.352 77.099 76.844
35.459 33.816 31.698 30.580 26.557
12.606 8.848
Parameter 值
1 Origin Bruker BioSpin GmbH
2 Solvent CDCl3
3 Temperature 292.2
4 Number of Scans 10
5 Spectrometer Frequency 125.77
6 Nucleus 13C
3m
f1 (ppm)

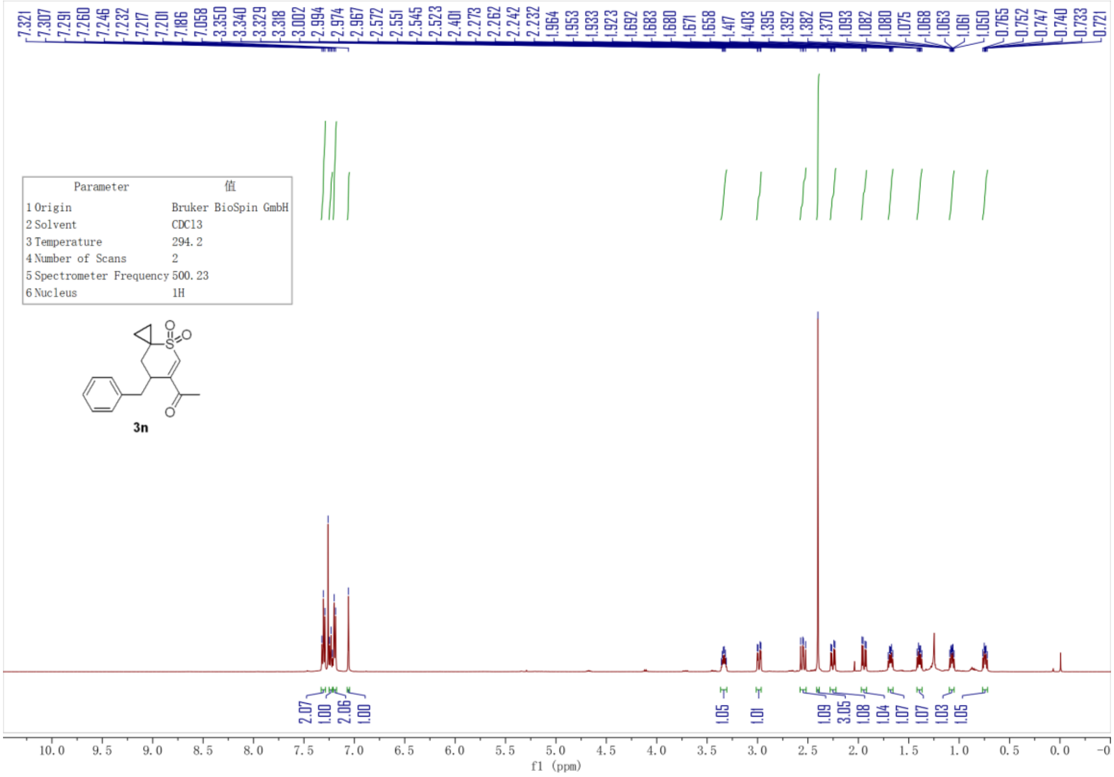
7.321 7.307 7.291 7.260 7.246 7.232 7.217 7.201 7.186 7.058 3.350 3.340 3.329 3.318 3.002 2.994 2.974 2.967 2.572 2.551 2.545 2.523 2.401 2.273 2.262 2.242 2.232 1.964 1.953 1.933 1.923 1.692 1.683 1.680 1.671 1.658 1.417 1.403 1.395 1.392 1.382 1.370 1.093 1.082 1.080 1.075 1.068 1.063 1.061 1.050 0.765 0.752 0.747 0.740 0.733 0.721
Parameter 值
1 Origin Bruker BioSpin GmbH
2 Solvent CDCl3
3 Temperature 294.2
4 Number of Scans 2
5 Spectrometer Frequency 500.23
6 Nucleus 1H
3n
2.07 1.00 2.06 1.00 1.05 1.01 1.09 3.05 1.08 1.04 1.07 1.07 1.03 1.05
f1 (ppm)

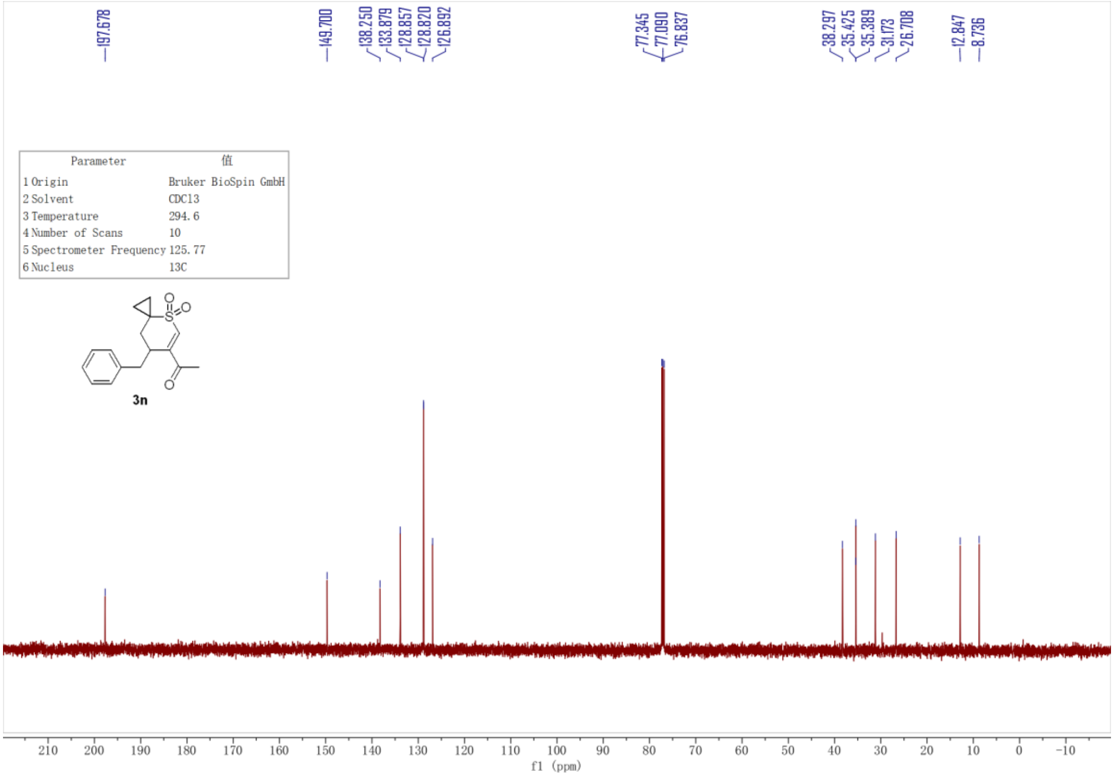
197.678
149.700 138.250 133.879 128.857 128.820 126.892
77.345 77.090 76.837
38.297 35.425 35.389 31.173 26.708 12.847 8.736
Parameter 值
1 Origin Bruker BioSpin GmbH
2 Solvent CDCl3
3 Temperature 294.6
4 Number of Scans 10
5 Spectrometer Frequency 125.77
6 Nucleus 13C
3n
f1 (ppm)

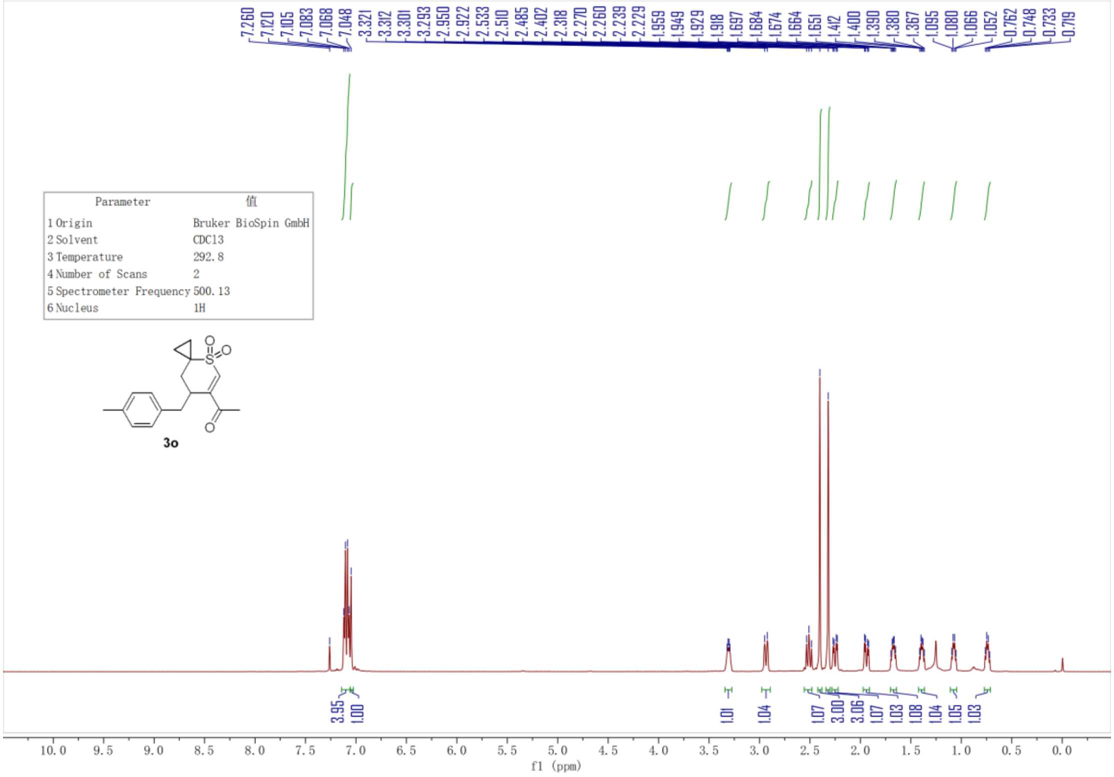
7.260
7.120
7.105
7.083
7.068
7.048
3.321
3.312
3.301
3.293
2.950
2.922
2.533
2.510
2.485
2.402
2.318
2.270
2.260
2.239
2.229
1.959
1.949
1.929
1.918
1.697
1.684
1.674
1.664
1.651
1.412
1.400
1.390
1.380
1.367
1.095
1.080
1.066
1.052
0.762
0.748
0.733
0.719
Parameter
值
1 Origin
Bruker BioSpin GmbH
2 Solvent
CDCl3
3 Temperature
292.8
4 Number of Scans
2
5 Spectrometer Frequency
500.13
6 Nucleus
1H
3o
3.95
1.00
1.01
1.04
1.07
3.00
3.06
1.07
1.03
1.08
1.04
1.05
1.03
f1 (ppm)

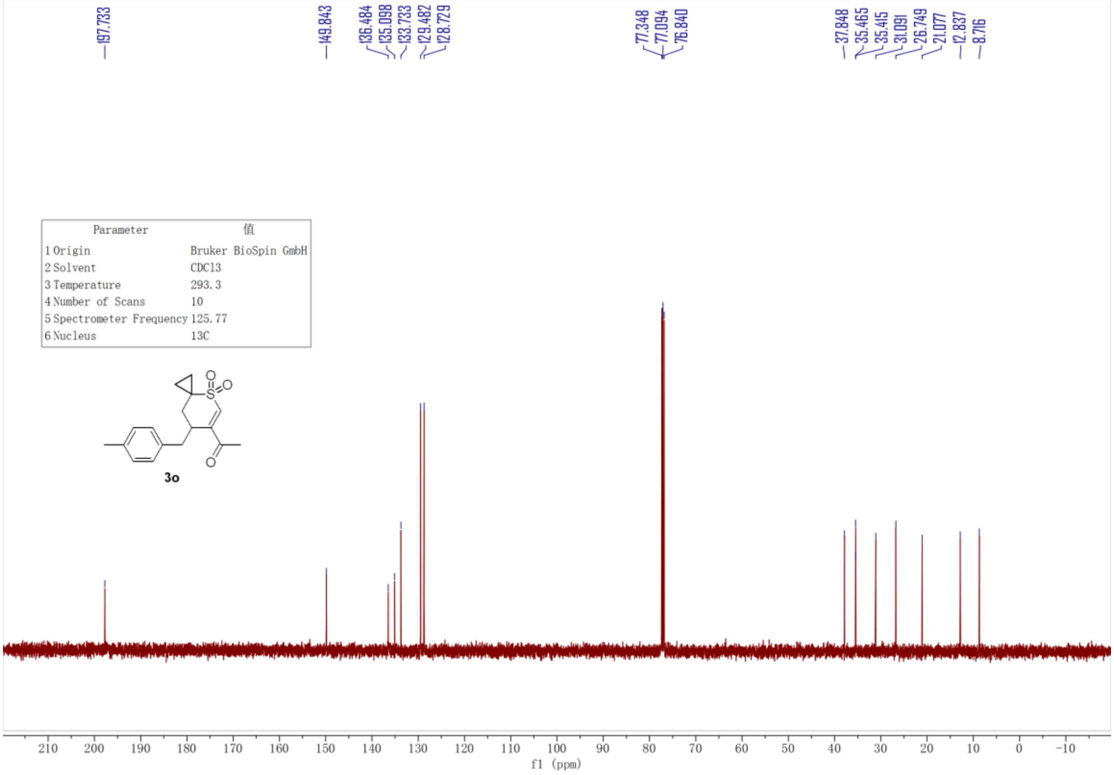
197.733
149.843
136.484
135.098
133.733
129.482
128.729
77.348
77.094
76.840
37.848
35.465
35.415
31.091
26.749
21.077
12.837
8.716
Parameter
值
1 Origin
Bruker BioSpin GmbH
2 Solvent
CDCl3
3 Temperature
293.3
4 Number of Scans
10
5 Spectrometer Frequency
125.77
6 Nucleus
13C
3o
f1 (ppm)

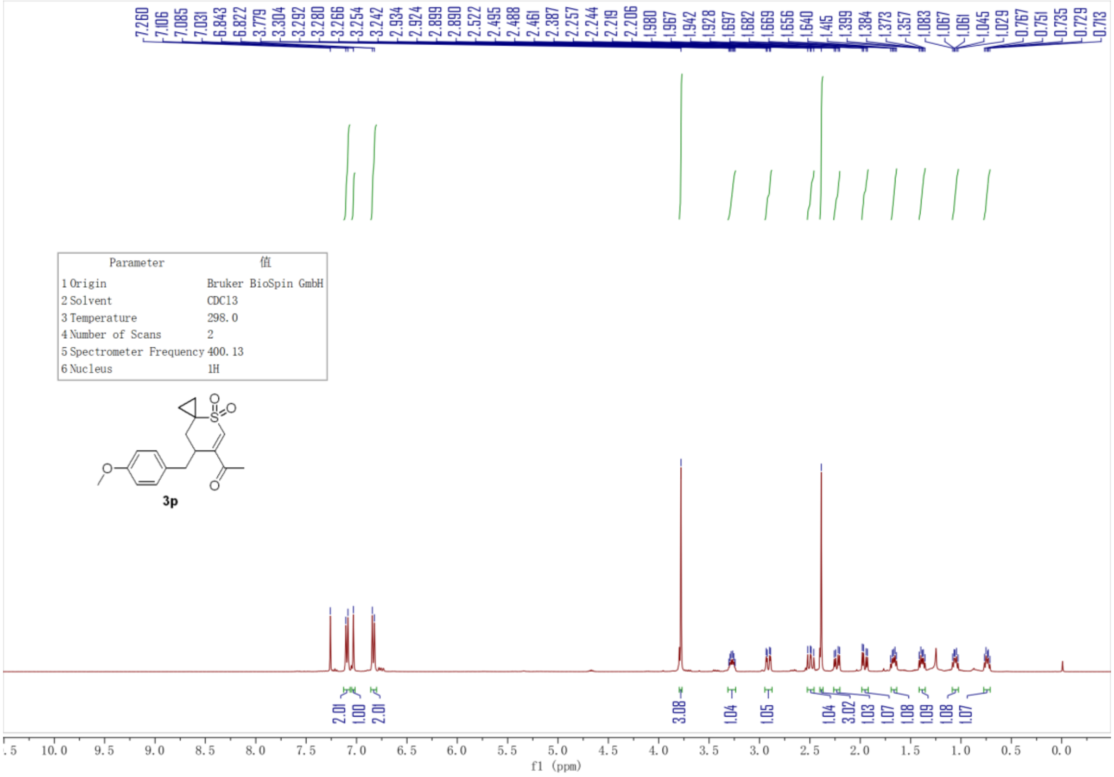
7.260
7.106
7.085
7.031
6.843
6.822
3.779
3.304
3.292
3.280
3.266
3.254
3.242
2.934
2.924
2.899
2.890
2.522
2.495
2.488
2.461
2.387
2.257
2.244
2.219
2.206
1.980
1.967
1.942
1.928
1.697
1.682
1.669
1.656
1.640
1.415
1.399
1.384
1.373
1.357
1.083
1.067
1.061
1.045
1.029
0.767
0.751
0.735
0.729
0.713
Parameter 值
1 Origin Bruker BioSpin GmbH
2 Solvent CDCl3
3 Temperature 298.0
4 Number of Scans 2
5 Spectrometer Frequency 400.13
6 Nucleus 1H
3p
2.01
1.00
2.01
3.08
1.04
1.05
1.04
3.02
1.03
1.07
1.08
1.09
1.08
1.07
f1 (ppm)

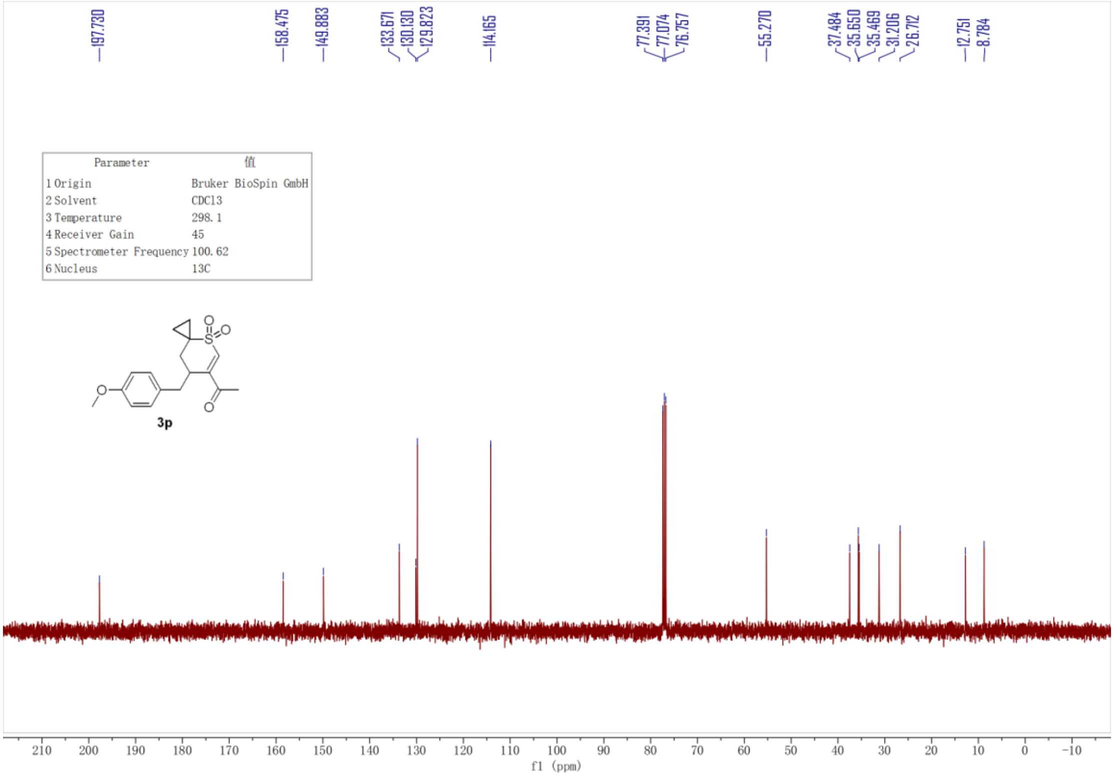
197.730
158.475
149.883
133.671
130.130
129.823
114.165
77.391
77.074
76.757
55.270
37.484
35.650
35.469
31.206
26.712
12.751
8.784
Parameter 值
1 Origin Bruker BioSpin GmbH
2 Solvent CDCl3
3 Temperature 298.1
4 Receiver Gain 45
5 Spectrometer Frequency 100.62
6 Nucleus 13C
3p
f1 (ppm)

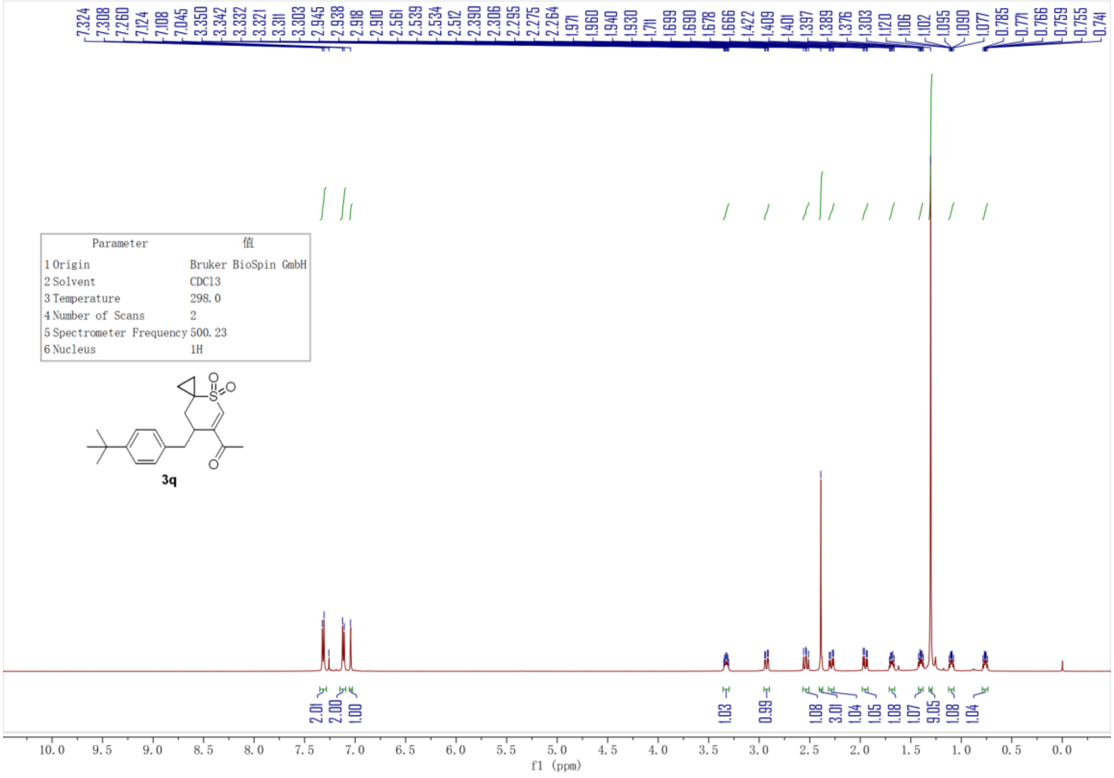

Parameter 值
1 Origin Bruker BioSpin GmbH
2 Solvent CDCl3
3 Temperature 298.0
4 Number of Scans 2
5 Spectrometer Frequency 500.23
6 Nucleus 1H
3q
f1 (ppm)

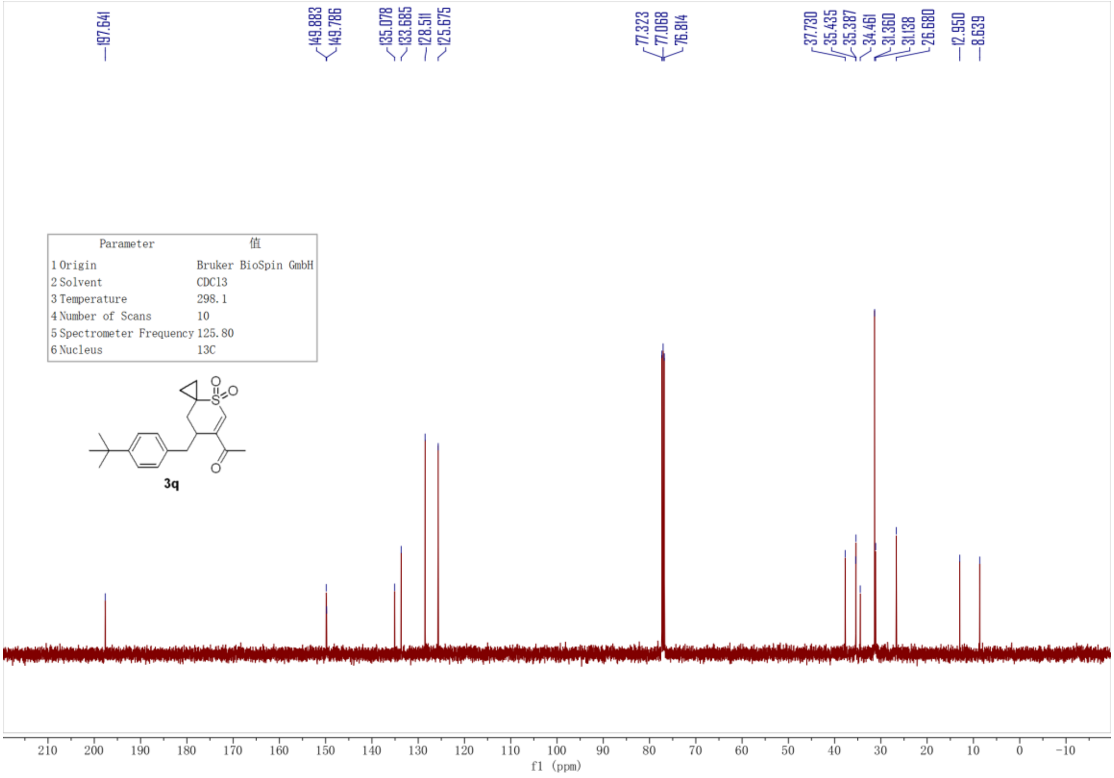

Parameter 值
1 Origin Bruker BioSpin GmbH
2 Solvent CDCl3
3 Temperature 298.1
4 Number of Scans 10
5 Spectrometer Frequency 125.80
6 Nucleus 13C
3q
f1 (ppm)

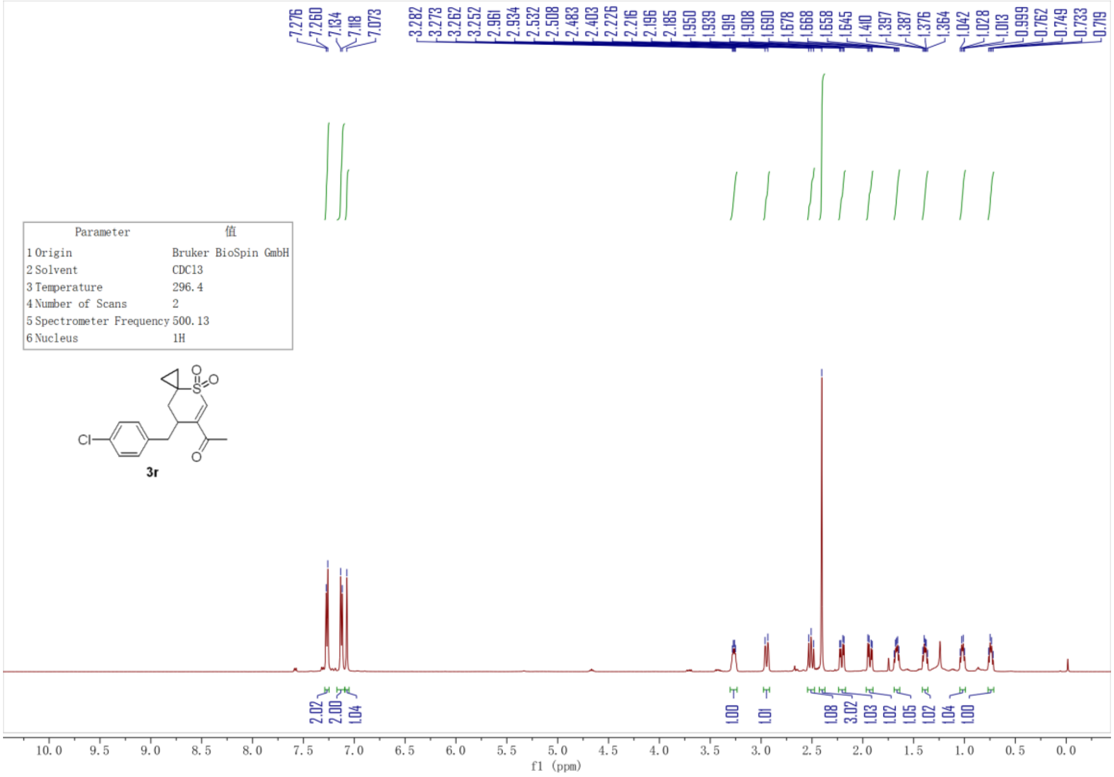

Parameter
值
1 Origin
Bruker BioSpin GmbH
2 Solvent
CDCl3
3 Temperature
296.4
4 Number of Scans
2
5 Spectrometer Frequency
500.13
6 Nucleus
1H
3r
f1 (ppm)

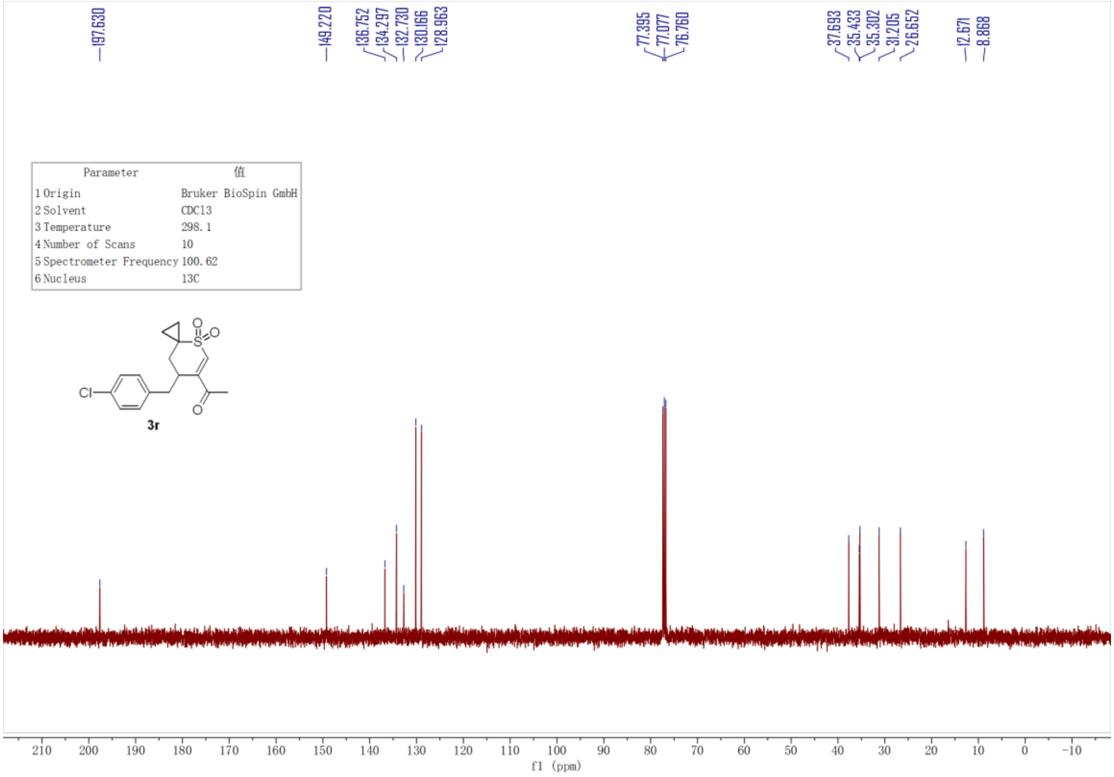

Parameter
值
1 Origin
Bruker BioSpin GmbH
2 Solvent
CDCl3
3 Temperature
298.1
4 Number of Scans
10
5 Spectrometer Frequency
100.62
6 Nucleus
13C
3r
f1 (ppm)

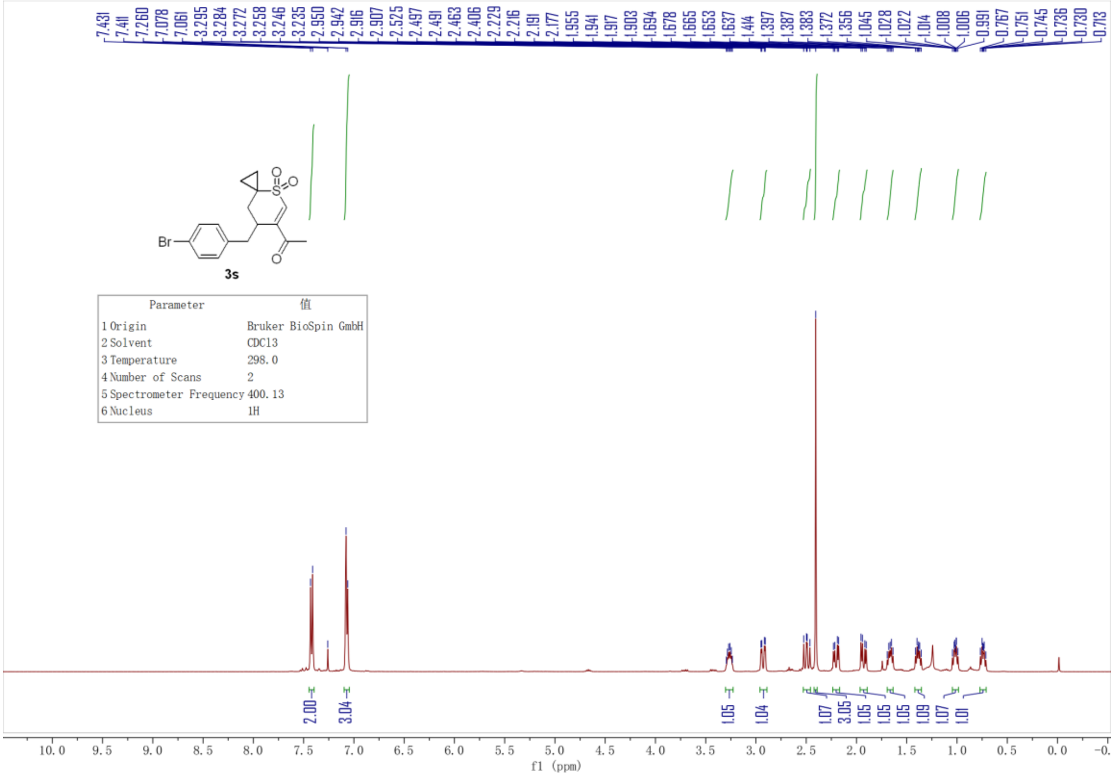
7.431 7.411 7.260 7.078 7.061 3.295 3.284 3.272 3.258 3.246 3.235 2.950 2.942 2.916 2.907 2.525 2.497 2.491 2.463 2.406 2.229 2.216 2.191 2.177 1.955 1.941 1.917 1.903 1.694 1.678 1.665 1.653 1.637 1.414 1.397 1.387 1.383 1.372 1.356 1.045 1.028 1.022 1.014 1.008 1.006 0.991 0.767 0.751 0.745 0.736 0.730 0.713
Br
3s
Parameter 值
1 Origin Bruker BioSpin GmbH
2 Solvent CDCl3
3 Temperature 298.0
4 Number of Scans 2
5 Spectrometer Frequency 400.13
6 Nucleus 1H
2.00 3.04 1.05 1.04 1.07 3.05 1.05 1.05 1.05 1.09 1.07 1.01
f1 (ppm)

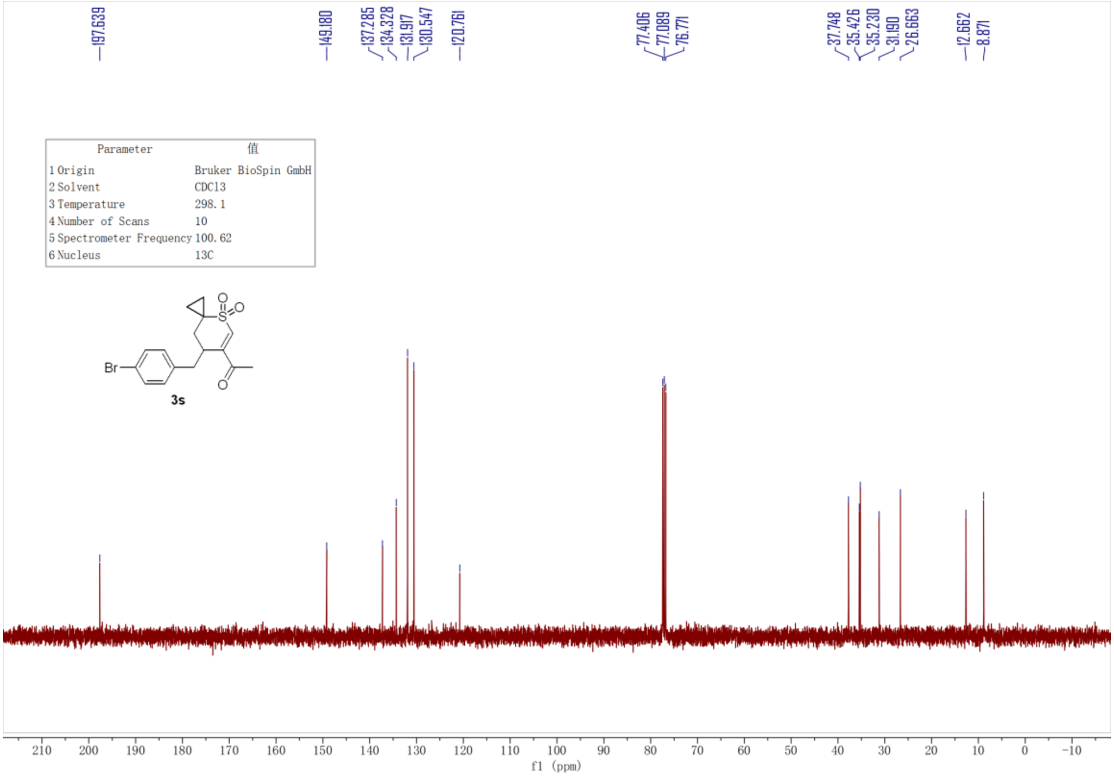
197.639 149.180 137.285 134.328 131.917 130.547 120.761 77.406 77.089 76.771 37.748 35.426 35.230 31.190 26.663 12.662 8.871
Parameter 值
1 Origin Bruker BioSpin GmbH
2 Solvent CDCl3
3 Temperature 298.1
4 Number of Scans 10
5 Spectrometer Frequency 100.62
6 Nucleus 13C
Br
3s
f1 (ppm)

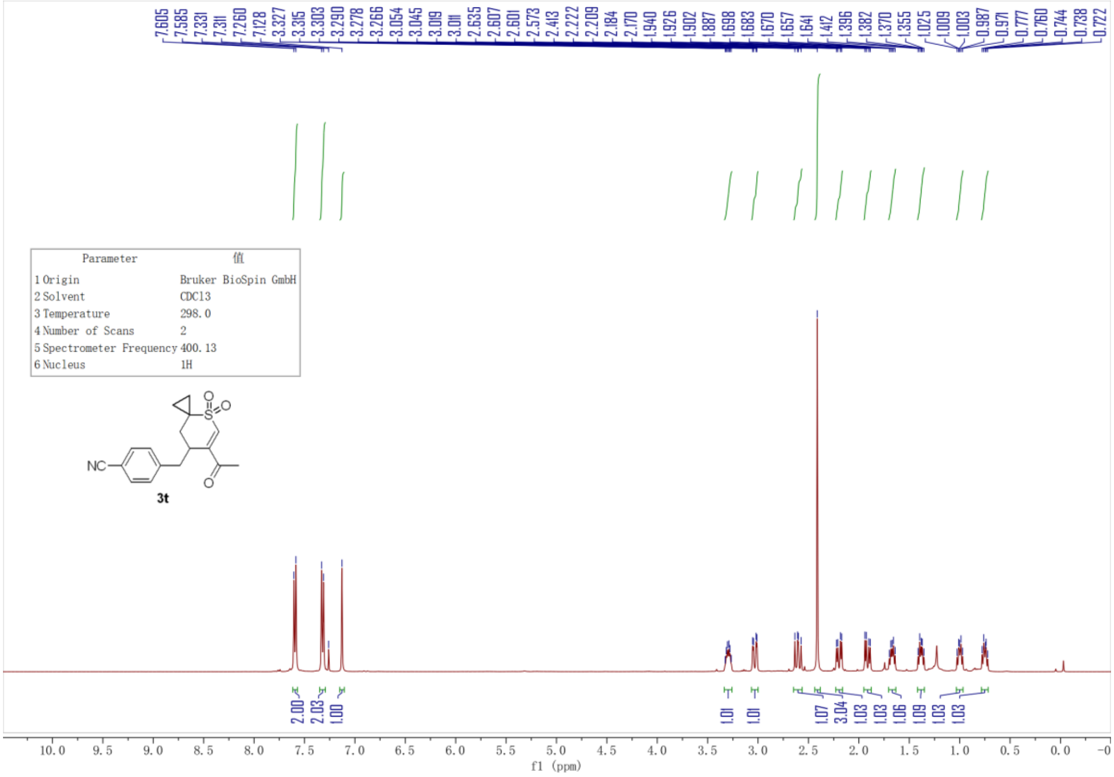
7.605 7.585 7.331 7.311 7.260 7.128 3.327 3.315 3.303 3.290 3.278 3.266 3.054 3.045 3.019 3.011 2.635 2.607 2.601 2.573 2.413 2.222 2.209 2.184 2.170 1.940 1.926 1.902 1.887 1.698 1.683 1.670 1.657 1.641 1.412 1.396 1.382 1.370 1.355 1.025 1.009 1.003 0.987 0.971 0.777 0.760 0.744 0.738 0.722
Parameter 值
1 Origin Bruker BioSpin GmbH
2 Solvent CDCl3
3 Temperature 298.0
4 Number of Scans 2
5 Spectrometer Frequency 400.13
6 Nucleus 1H
NC
3t
2.00 2.03 1.00 1.01 1.01 1.07 3.04 1.03 1.03 1.06 1.09 1.03 1.03
10.0 9.5 9.0 8.5 8.0 7.5 7.0 6.5 6.0 5.5 5.0 4.5 4.0 3.5 3.0 2.5 2.0 1.5 1.0 0.5 0.0 -0
f1 (ppm)

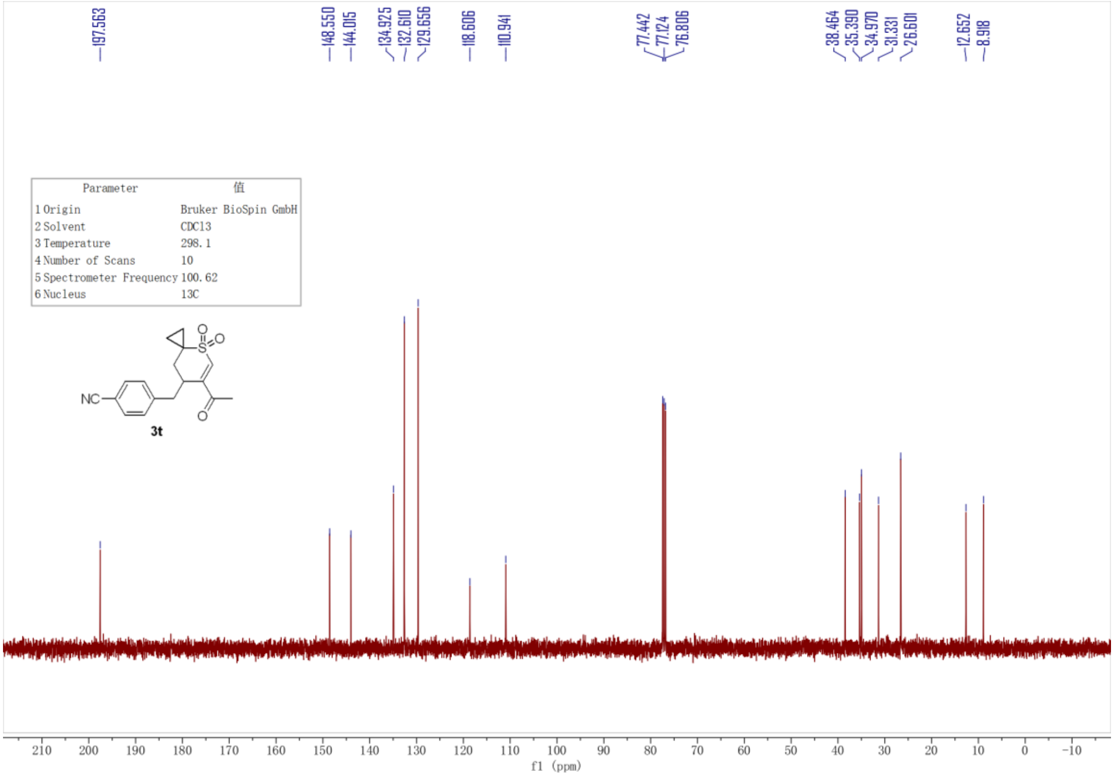
197.563 148.550 144.015 134.925 132.610 129.656 118.606 110.941 77.442 77.124 76.806 38.464 35.390 34.970 31.331 26.601 12.652 8.918
Parameter 值
1 Origin Bruker BioSpin GmbH
2 Solvent CDCl3
3 Temperature 298.1
4 Number of Scans 10
5 Spectrometer Frequency 100.62
6 Nucleus 13C
NC
3t
210 200 190 180 170 160 150 140 130 120 110 100 90 80 70 60 50 40 30 20 10 0 -10
f1 (ppm)

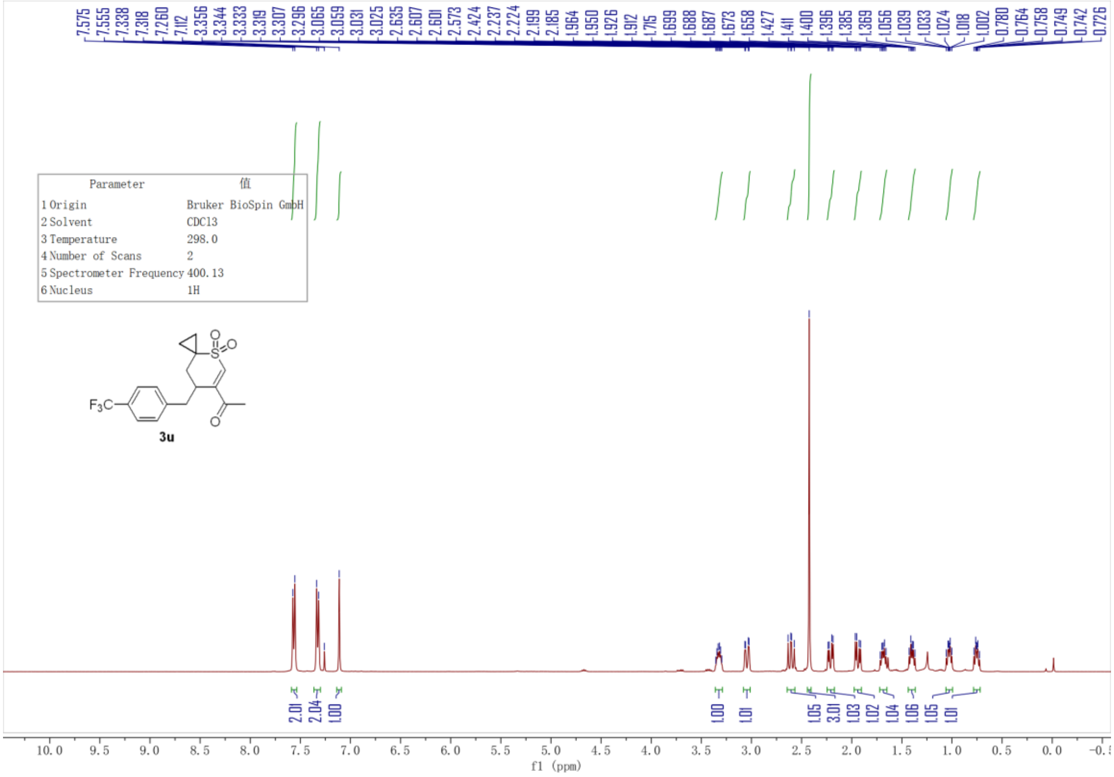
7.575
7.555
7.338
7.318
7.260
7.112
3.356
3.344
3.333
3.319
3.307
3.296
3.065
3.059
3.031
3.025
2.635
2.607
2.601
2.573
2.424
2.237
2.224
2.199
2.185
1.964
1.950
1.926
1.912
1.715
1.699
1.688
1.687
1.673
1.658
1.427
1.411
1.400
1.396
1.385
1.369
1.056
1.039
1.033
1.024
1.018
1.002
0.780
0.764
0.758
0.749
0.742
0.726
Parameter 值
1 Origin Bruker BioSpin GmbH
2 Solvent CDCl3
3 Temperature 298.0
4 Number of Scans 2
5 Spectrometer Frequency 400.13
6 Nucleus 1H
F_3C
3u
2.01
2.04
1.00
1.00
1.01
1.05
3.01
1.03
1.02
1.04
1.06
1.05
1.01
f1 (ppm)

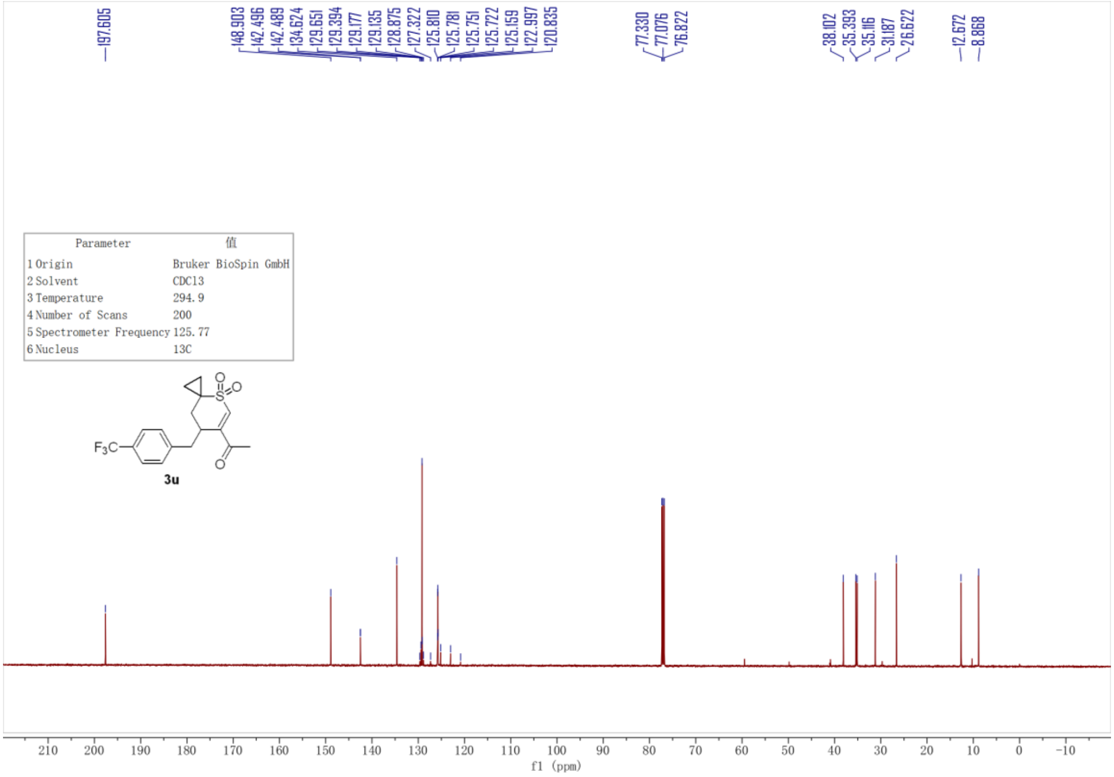
197.605
148.903
142.496
142.489
134.624
129.651
129.394
129.177
129.135
128.875
127.322
125.810
125.781
125.751
125.722
125.159
122.997
120.835
77.330
77.076
76.822
38.102
35.393
35.116
31.187
26.622
12.672
8.868
Parameter 值
1 Origin Bruker BioSpin GmbH
2 Solvent CDCl3
3 Temperature 294.9
4 Number of Scans 200
5 Spectrometer Frequency 125.77
6 Nucleus 13C
F_3C
3u
f1 (ppm)

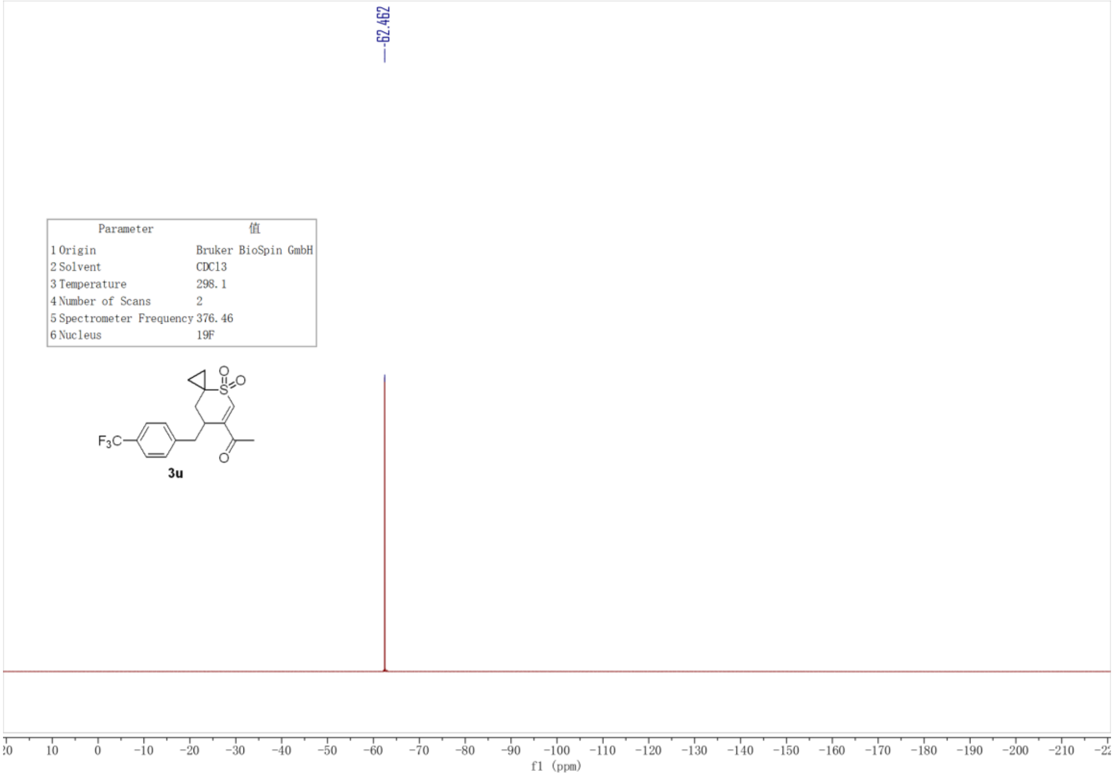

-62.462
Parameter 值
1 Origin Bruker BioSpin GmbH
2 Solvent CDCl3
3 Temperature 298.1
4 Number of Scans 2
5 Spectrometer Frequency 376.46
6 Nucleus 19F
3u
f1 (ppm)

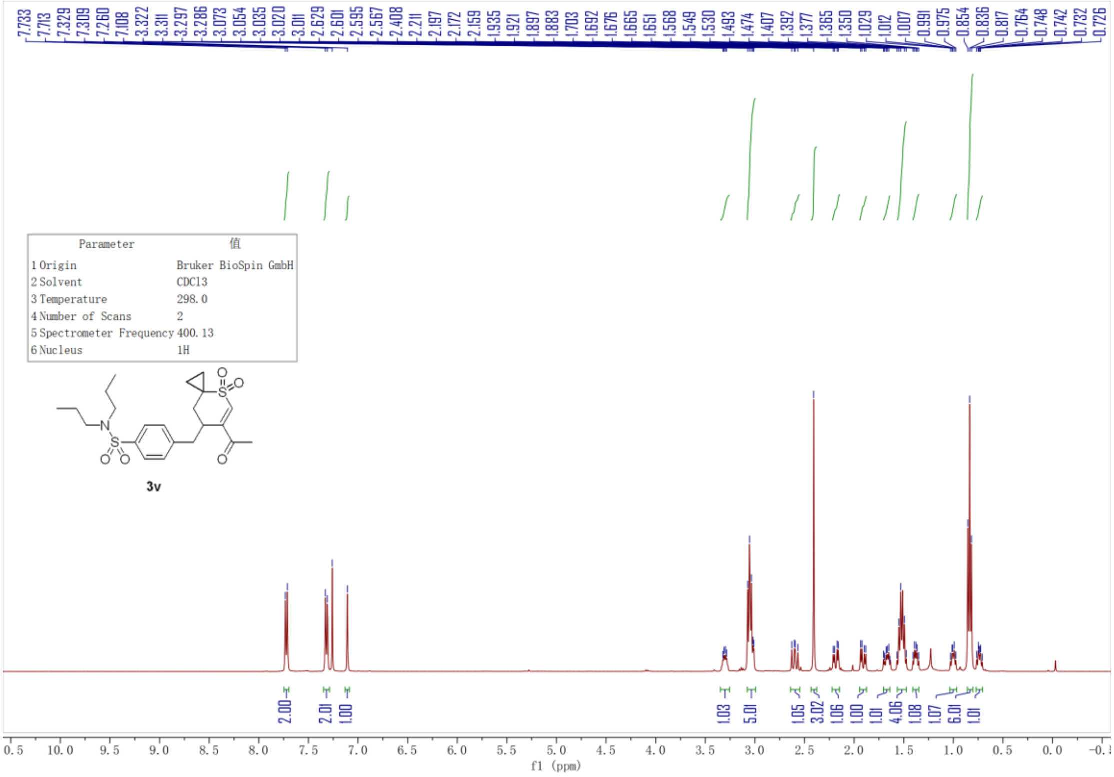

7.733 7.713 7.329 7.309 7.260 7.108 3.322 3.311 3.297 3.286 3.073 3.054 3.035 3.020 3.011 2.629 2.601 2.595 2.567 2.408 2.211 2.197 2.172 2.159 1.935 1.921 1.897 1.883 1.703 1.692 1.676 1.665 1.651 1.568 1.549 1.530 1.493 1.474 1.407 1.392 1.377 1.365 1.350 1.029 1.012 1.007 0.991 0.975 0.854 0.836 0.817 0.764 0.748 0.742 0.732 0.726
Parameter 值
1 Origin Bruker BioSpin GmbH
2 Solvent CDCl3
3 Temperature 298.0
4 Number of Scans 2
5 Spectrometer Frequency 400.13
6 Nucleus 1H
3v
2.00 2.01 1.00 1.03 5.01 1.05 3.02 1.06 1.00 1.01 4.06 1.08 1.07 6.01 1.01
f1 (ppm)

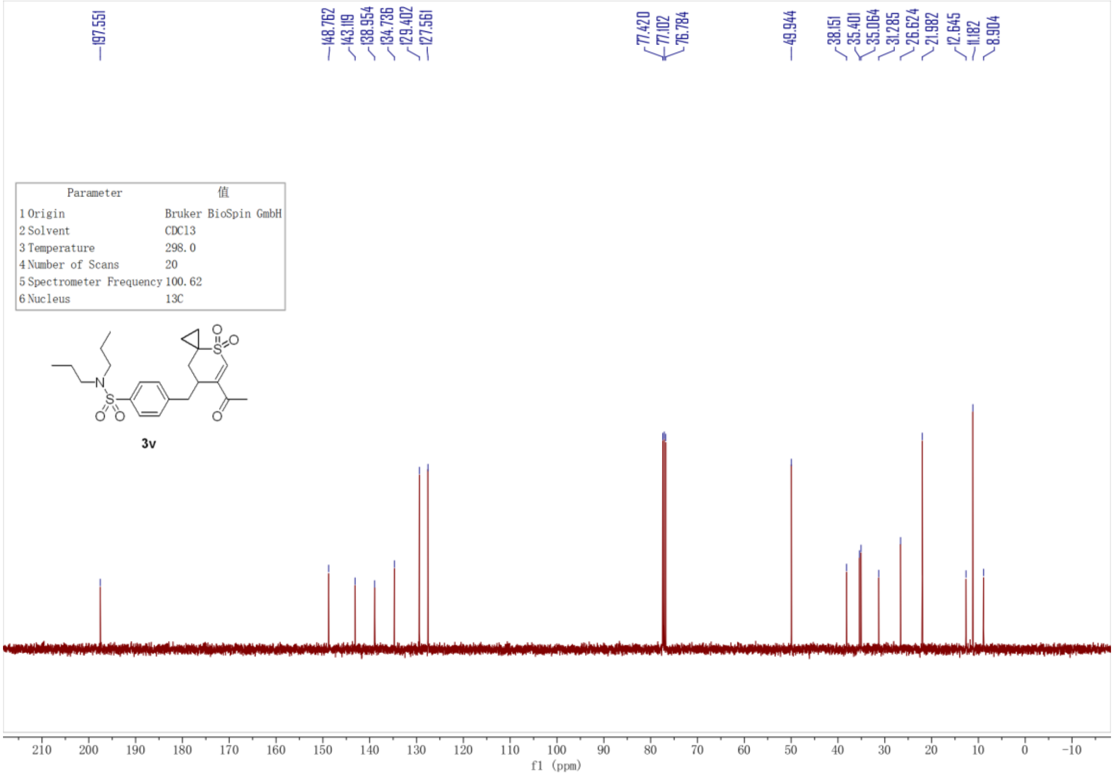
197.551
148.762
143.119
138.954
134.736
129.402
127.561
77.420
77.102
76.784
49.944
38.151
35.401
35.064
31.285
26.624
21.982
12.645
11.182
8.904
Parameter 值
1 Origin Bruker BioSpin GmbH
2 Solvent CDCl3
3 Temperature 298.0
4 Number of Scans 20
5 Spectrometer Frequency 100.62
6 Nucleus 13C
3v
f1 (ppm)

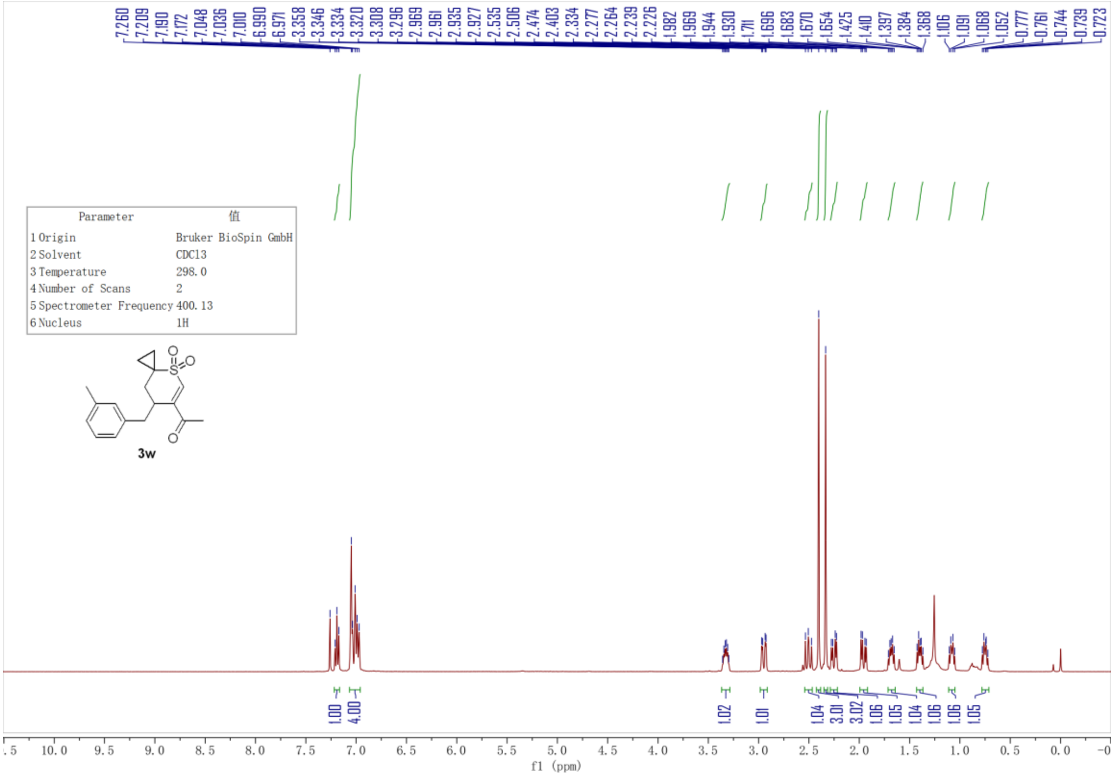
7.260
7.209
7.190
7.172
7.048
7.036
7.010
6.990
6.971
3.358
3.346
3.334
3.320
3.308
3.296
2.969
2.961
2.935
2.927
2.535
2.506
2.474
2.403
2.334
2.277
2.264
2.239
2.226
1.982
1.969
1.944
1.930
1.711
1.696
1.683
1.670
1.654
1.425
1.410
1.397
1.384
1.368
1.106
1.091
1.068
1.052
0.777
0.761
0.744
0.739
0.723
Parameter 值
1 Origin Bruker BioSpin GmbH
2 Solvent CDCl3
3 Temperature 298.0
4 Number of Scans 2
5 Spectrometer Frequency 400.13
6 Nucleus 1H
3w
1.00
4.00
1.02
1.01
1.04
3.01
3.02
1.06
1.05
1.04
1.06
1.05
f1 (ppm)

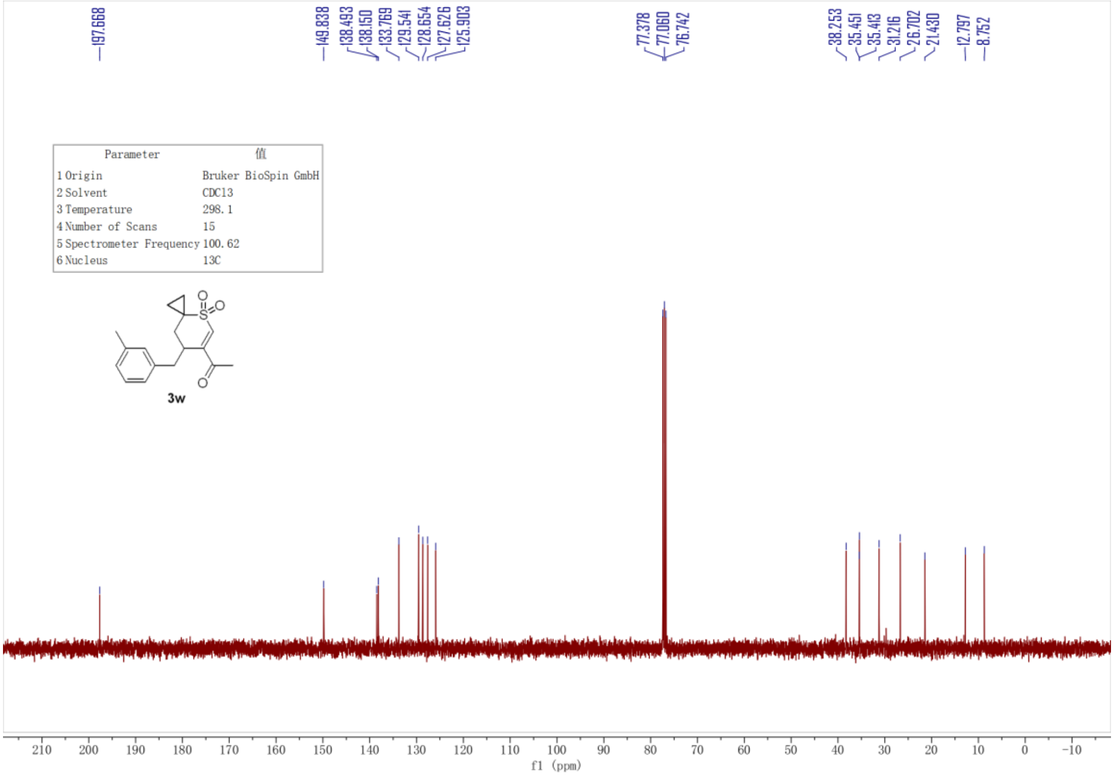
197.668
149.838
138.493
138.150
133.769
129.541
128.654
127.626
125.903
77.378
77.060
76.742
38.253
35.451
35.413
31.216
26.702
21.430
12.797
8.752
Parameter 值
1 Origin Bruker BioSpin GmbH
2 Solvent CDCl3
3 Temperature 298.1
4 Number of Scans 15
5 Spectrometer Frequency 100.62
6 Nucleus 13C
3w
f1 (ppm)

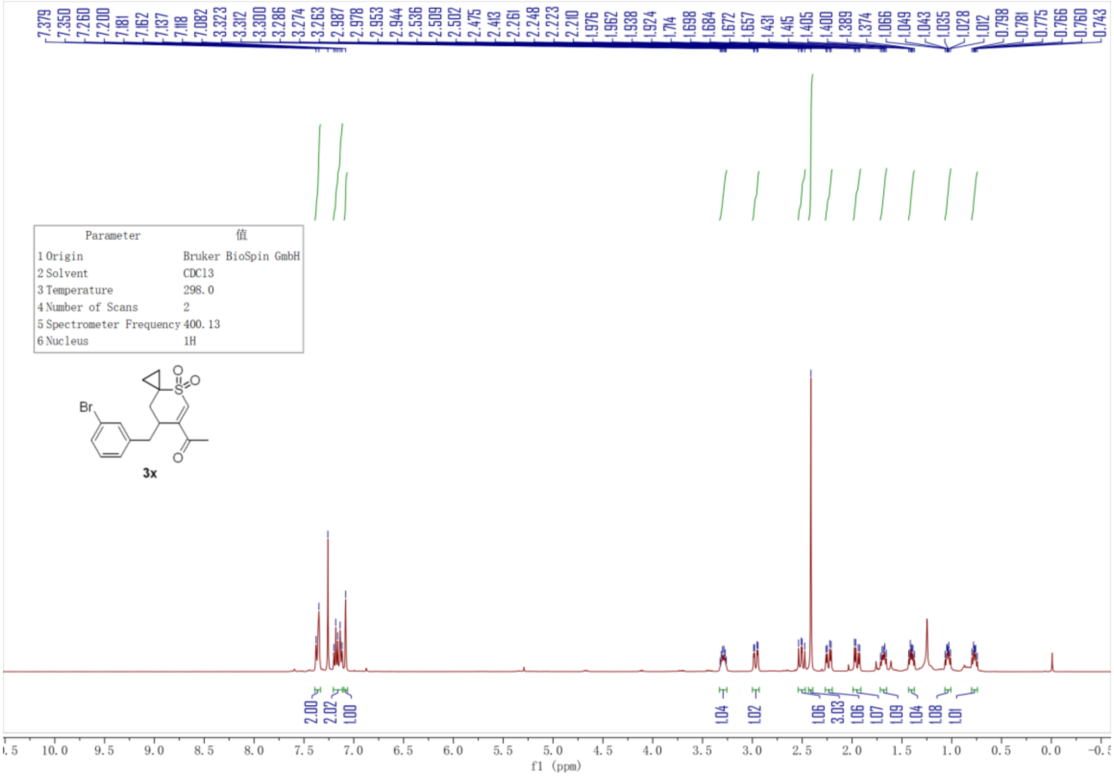
7.379
7.350
7.260
7.200
7.181
7.162
7.137
7.118
7.082
3.323
3.312
3.300
3.286
3.274
3.263
2.987
2.978
2.953
2.944
2.536
2.509
2.502
2.475
2.413
2.261
2.248
2.223
2.210
1.976
1.962
1.938
1.924
1.714
1.698
1.684
1.672
1.657
1.431
1.415
1.405
1.400
1.389
1.374
1.066
1.049
1.043
1.035
1.028
1.012
0.798
0.781
0.775
0.766
0.760
0.743
Parameter 值
1 Origin Bruker BioSpin GmbH
2 Solvent CDCl3
3 Temperature 298.0
4 Number of Scans 2
5 Spectrometer Frequency 400.13
6 Nucleus 1H
Br
3x
2.00
2.02
1.00
1.04
1.02
1.06
3.03
1.06
1.07
1.09
1.04
1.08
1.01
f1 (ppm)

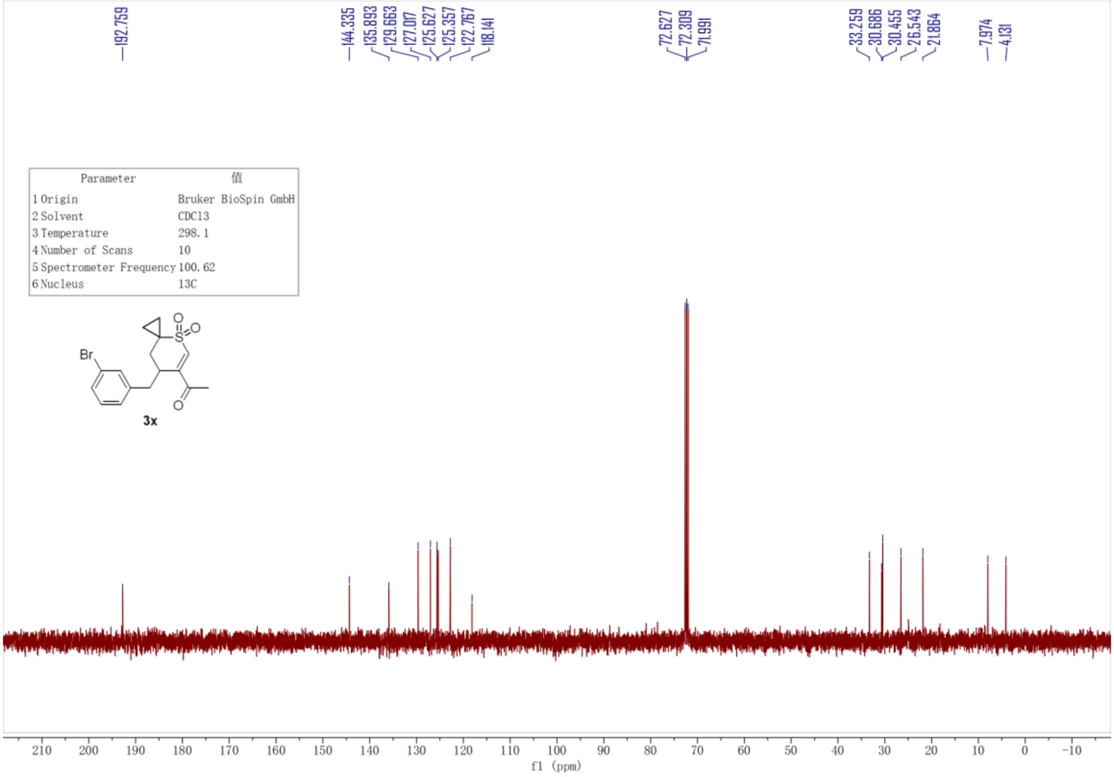
192.759
144.335
135.893
129.663
127.017
125.627
125.357
122.767
118.141
72.627
72.309
71.991
33.259
30.686
30.455
26.543
21.864
7.974
4.131
Parameter 值
1 Origin Bruker BioSpin GmbH
2 Solvent CDCl3
3 Temperature 298.1
4 Number of Scans 10
5 Spectrometer Frequency 100.62
6 Nucleus 13C
Br
S
O
O
O
3x
f1 (ppm)

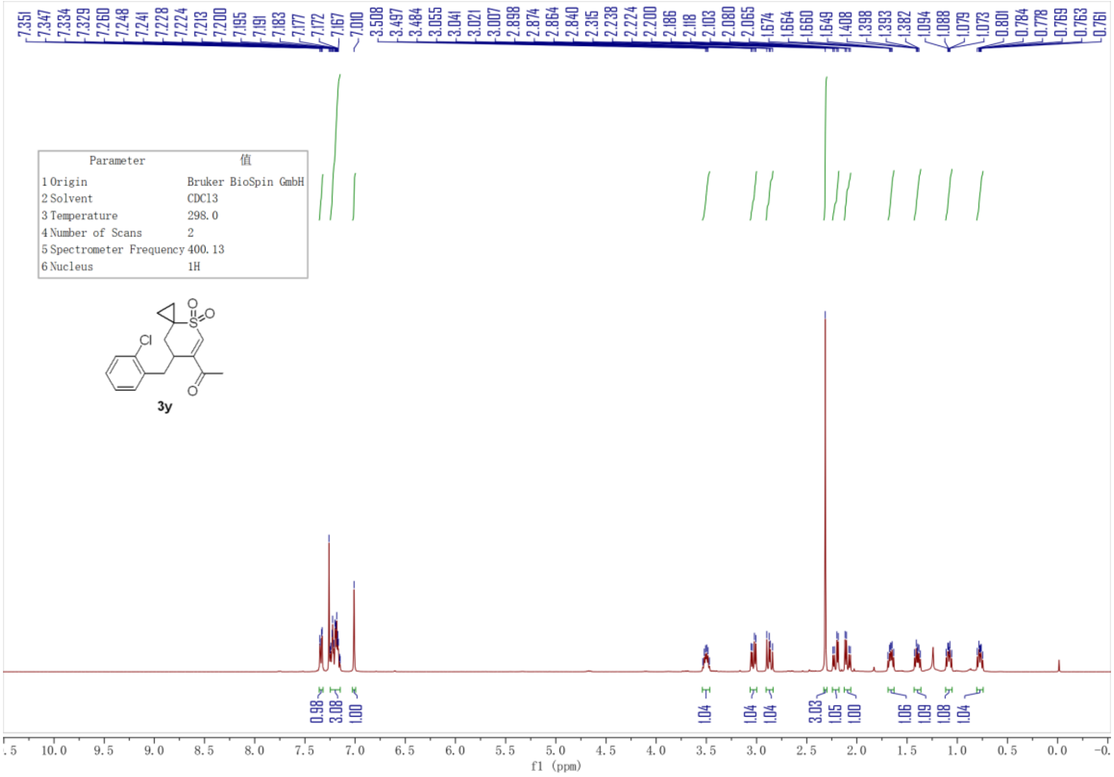
7.351
7.347
7.334
7.329
7.260
7.248
7.241
7.228
7.224
7.213
7.200
7.195
7.191
7.183
7.177
7.172
7.167
7.010
3.508
3.497
3.484
3.055
3.041
3.021
3.007
2.898
2.874
2.864
2.840
2.315
2.238
2.224
2.200
2.186
2.118
2.103
2.080
2.065
1.674
1.664
1.660
1.649
1.408
1.398
1.393
1.382
1.094
1.088
1.079
1.073
0.801
0.784
0.778
0.769
0.763
0.761
Parameter 值
1 Origin Bruker BioSpin GmbH
2 Solvent CDCl3
3 Temperature 298.0
4 Number of Scans 2
5 Spectrometer Frequency 400.13
6 Nucleus 1H
Cl
S
O
O
O
3y
0.98
3.08
1.00
1.04
1.04
1.04
3.03
1.05
1.00
1.06
1.09
1.08
1.04
f1 (ppm)

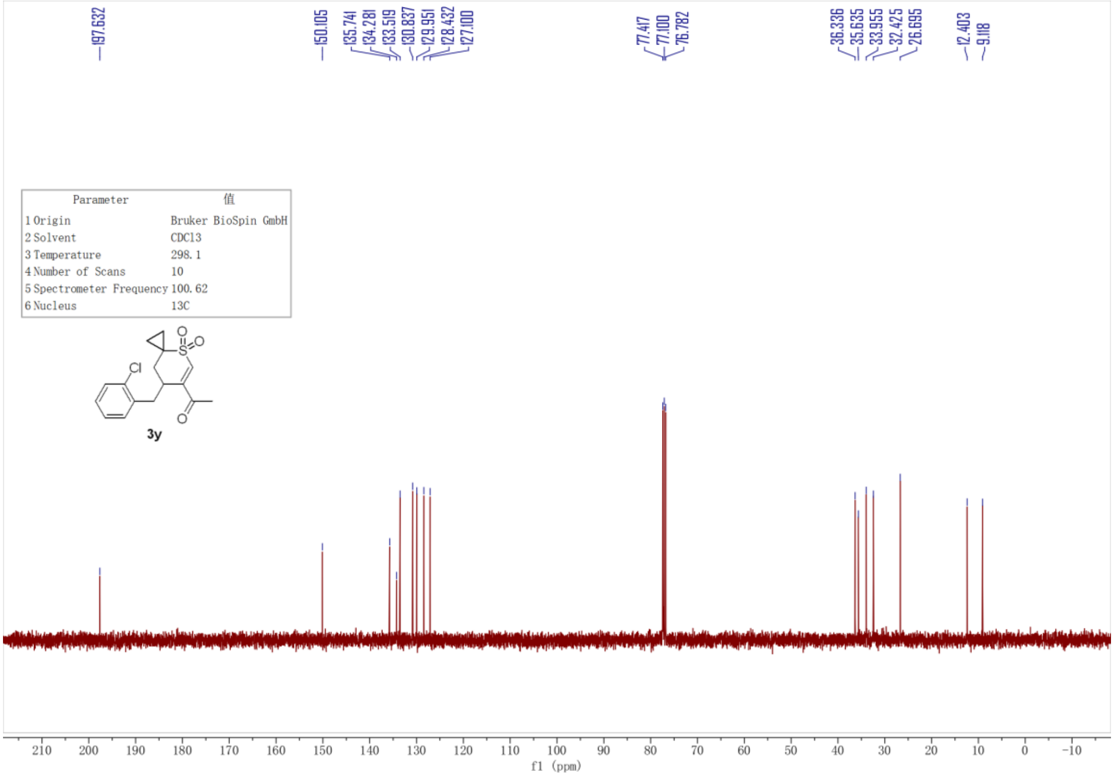

197.632
150.105
135.741
134.281
133.519
130.837
129.951
128.432
127.100
77.417
77.100
76.782
36.336
35.635
33.955
32.425
26.695
12.403
9.118
Parameter 值
1 Origin Bruker BioSpin GmbH
2 Solvent CDCl3
3 Temperature 298.1
4 Number of Scans 10
5 Spectrometer Frequency 100.62
6 Nucleus 13C
Cl
O
S
O
O
3y
f1 (ppm)

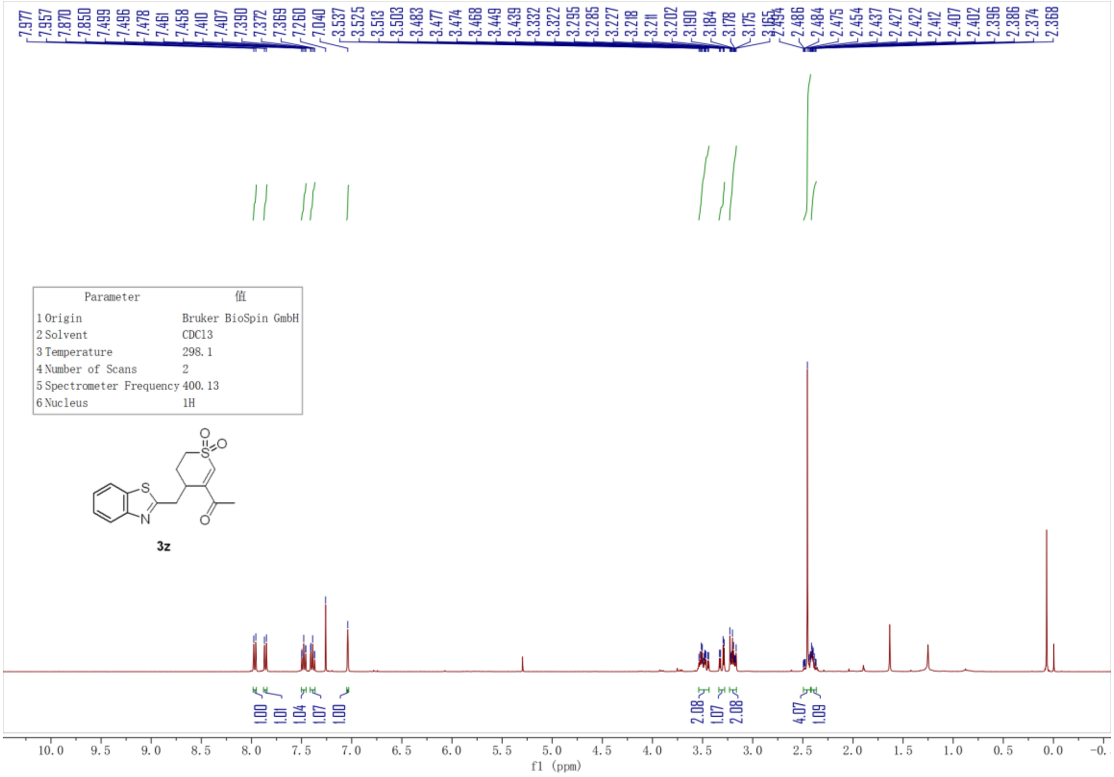

Parameter 值
1 Origin Bruker BioSpin GmbH
2 Solvent CDCl3
3 Temperature 298.1
4 Number of Scans 2
5 Spectrometer Frequency 400.13
6 Nucleus 1H
S
N
O
S
O
O
3z
1.00
1.01
1.04
1.07
1.00
2.08
1.07
2.08
4.07
1.09
f1 (ppm)

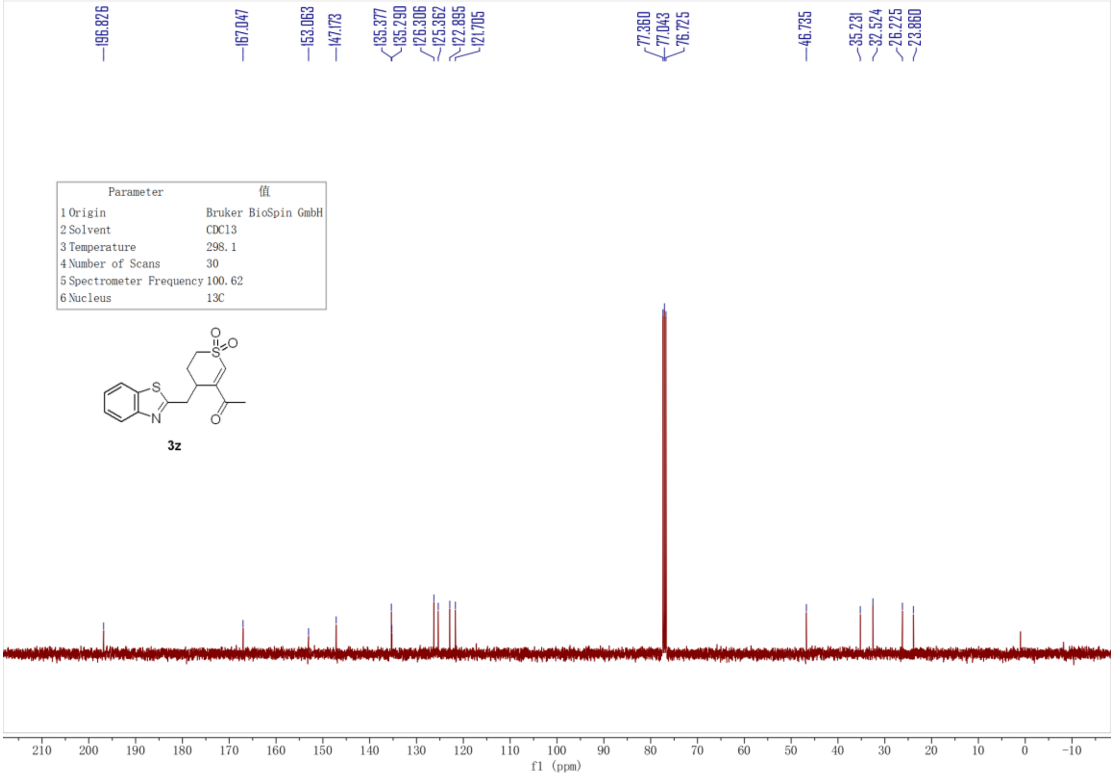
196.826
167.047
153.063
147.173
135.377
135.290
126.306
125.362
122.895
121.705
77.360
77.043
76.725
46.735
35.231
32.524
26.225
23.860
Parameter 值
1 Origin Bruker BioSpin GmbH
2 Solvent CDCl3
3 Temperature 298.1
4 Number of Scans 30
5 Spectrometer Frequency 100.62
6 Nucleus 13C
3z
210 200 190 180 170 160 150 140 130 120 110 100 90 80 70 60 50 40 30 20 10 0 -10
f1 (ppm)

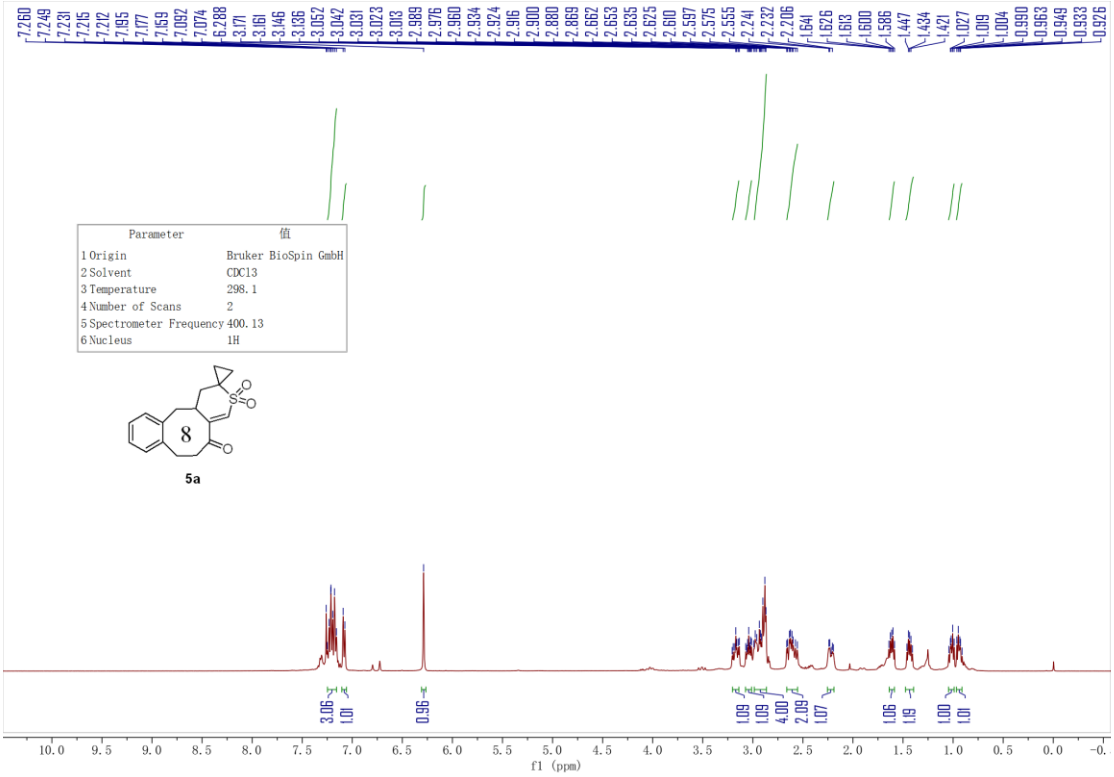
7.260
7.249
7.231
7.215
7.212
7.195
7.177
7.159
7.092
7.074
6.288
3.171
3.161
3.146
3.136
3.052
3.042
3.031
3.023
3.013
2.989
2.976
2.960
2.934
2.924
2.916
2.900
2.880
2.869
2.662
2.653
2.635
2.625
2.610
2.597
2.575
2.555
2.241
2.232
2.206
1.641
1.626
1.613
1.600
1.586
1.447
1.434
1.421
1.027
1.019
1.004
0.990
0.963
0.949
0.933
0.926
Parameter 值
1 Origin Bruker BioSpin GmbH
2 Solvent CDCl3
3 Temperature 298.1
4 Number of Scans 2
5 Spectrometer Frequency 400.13
6 Nucleus 1H
5a
3.06
1.01
0.96
1.09
1.09
4.00
2.09
1.07
1.06
1.19
1.00
1.01
10.0 9.5 9.0 8.5 8.0 7.5 7.0 6.5 6.0 5.5 5.0 4.5 4.0 3.5 3.0 2.5 2.0 1.5 1.0 0.5 0.0 -0.
f1 (ppm)

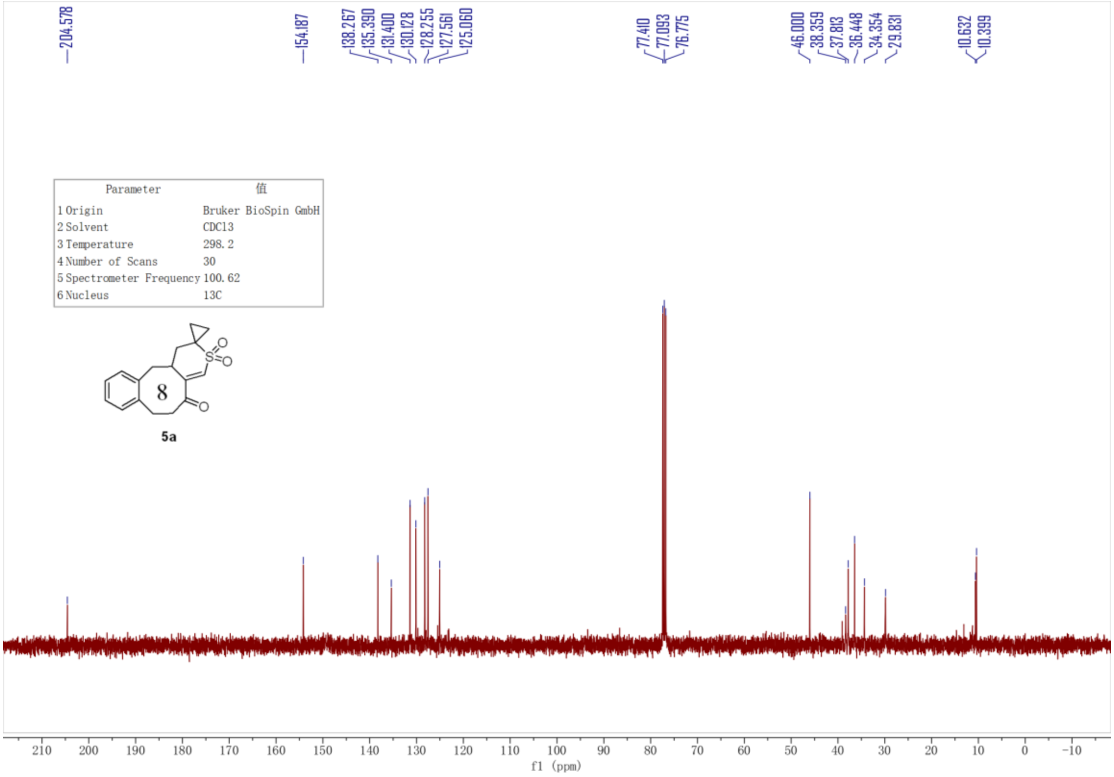

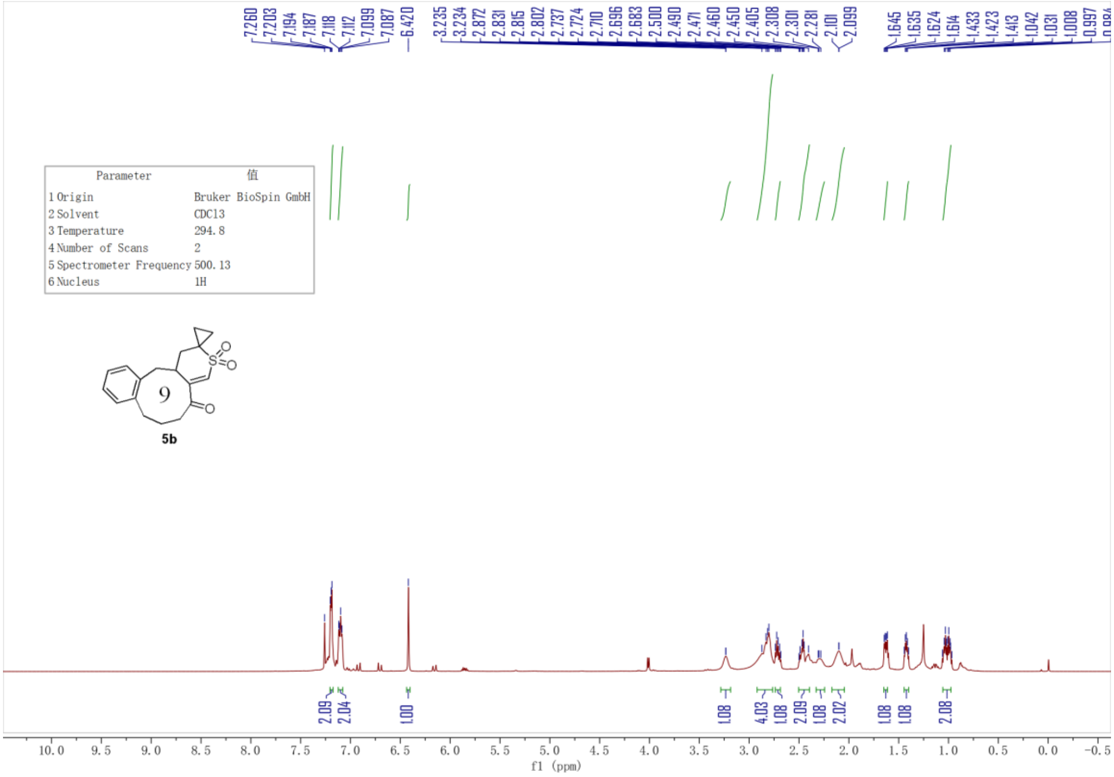

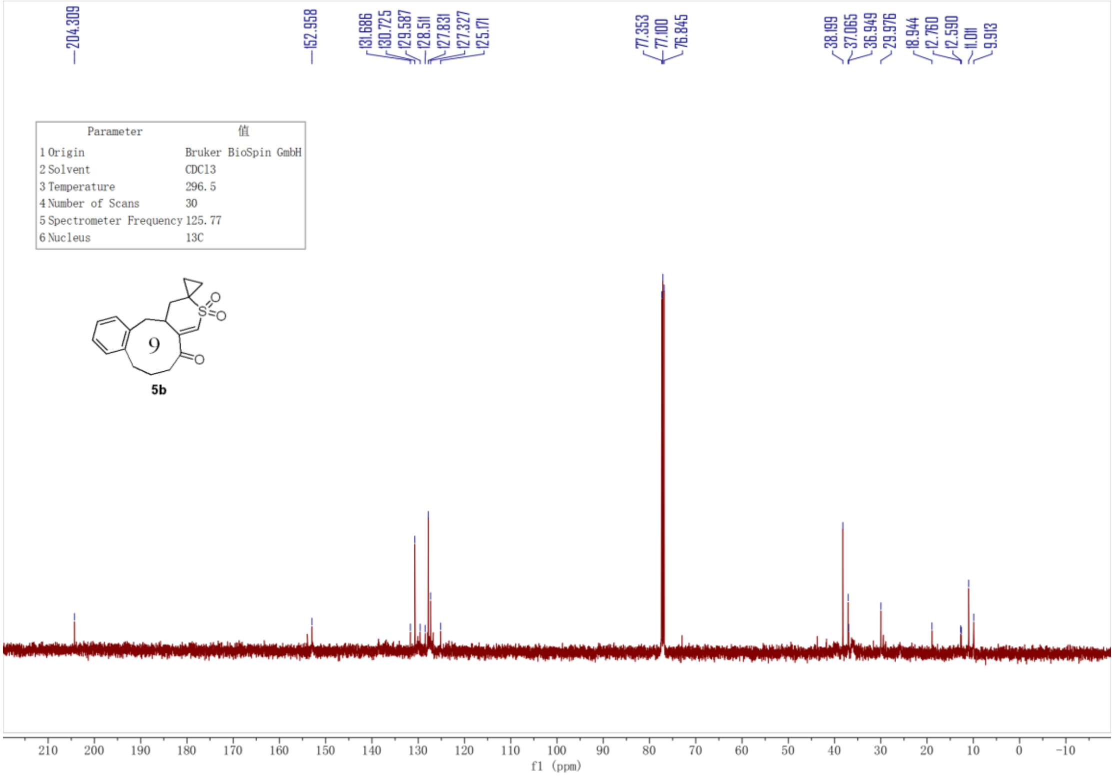
204.309
152.958
131.686
130.725
129.587
128.511
127.831
127.327
125.171
77.353
77.100
76.845
38.199
37.065
36.949
29.976
18.944
12.760
12.590
11.011
9.913
Parameter 值
1 Origin Bruker BioSpin GmbH
2 Solvent CDCl3
3 Temperature 296.5
4 Number of Scans 30
5 Spectrometer Frequency 125.77
6 Nucleus 13C
9
5b
f1 (ppm)

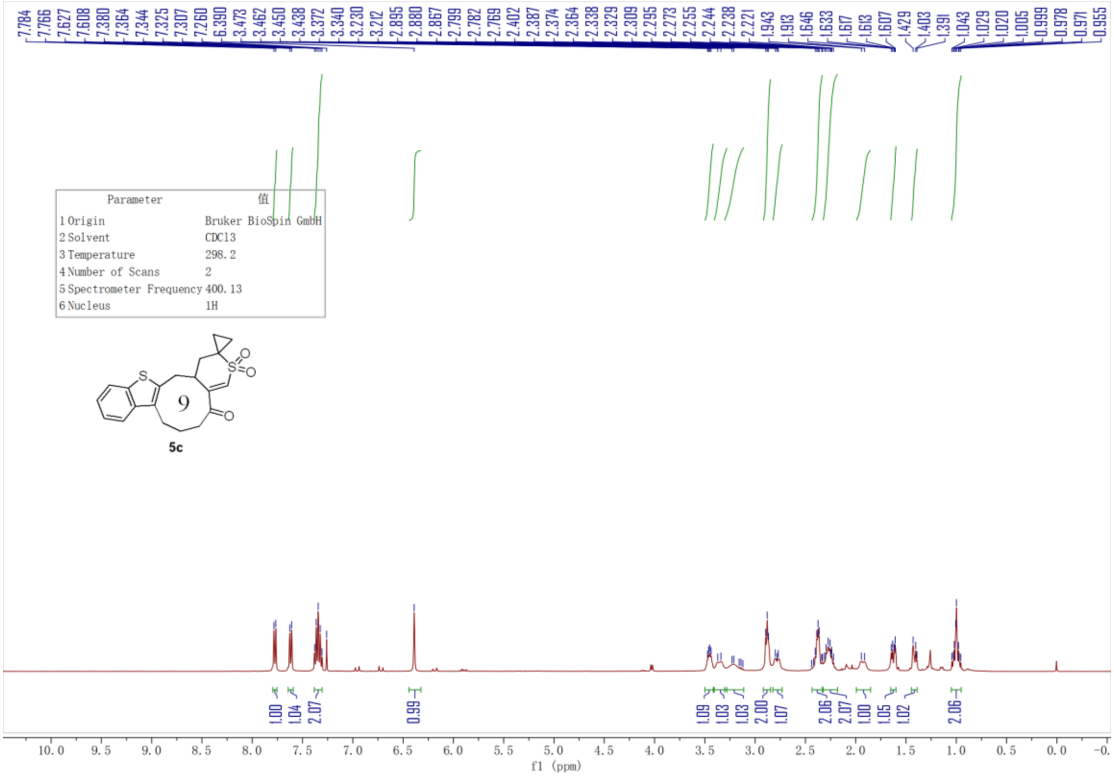
7.784
7.766
7.627
7.608
7.380
7.364
7.344
7.325
7.307
7.260
6.390
3.473
3.462
3.450
3.438
3.372
3.340
3.230
3.212
2.895
2.880
2.867
2.799
2.782
2.769
2.402
2.387
2.374
2.364
2.338
2.329
2.309
2.295
2.273
2.255
2.244
2.238
2.221
1.943
1.913
1.646
1.633
1.617
1.613
1.607
1.429
1.403
1.391
1.043
1.029
1.020
1.005
0.999
0.978
0.971
0.955
Parameter 值
1 Origin Bruker BioSpin GmbH
2 Solvent CDCl3
3 Temperature 298.2
4 Number of Scans 2
5 Spectrometer Frequency 400.13
6 Nucleus 1H
9
5c
1.00
1.04
2.07
0.99
1.09
1.03
1.03
2.00
1.07
2.06
2.07
1.00
1.05
1.02
2.06
f1 (ppm)

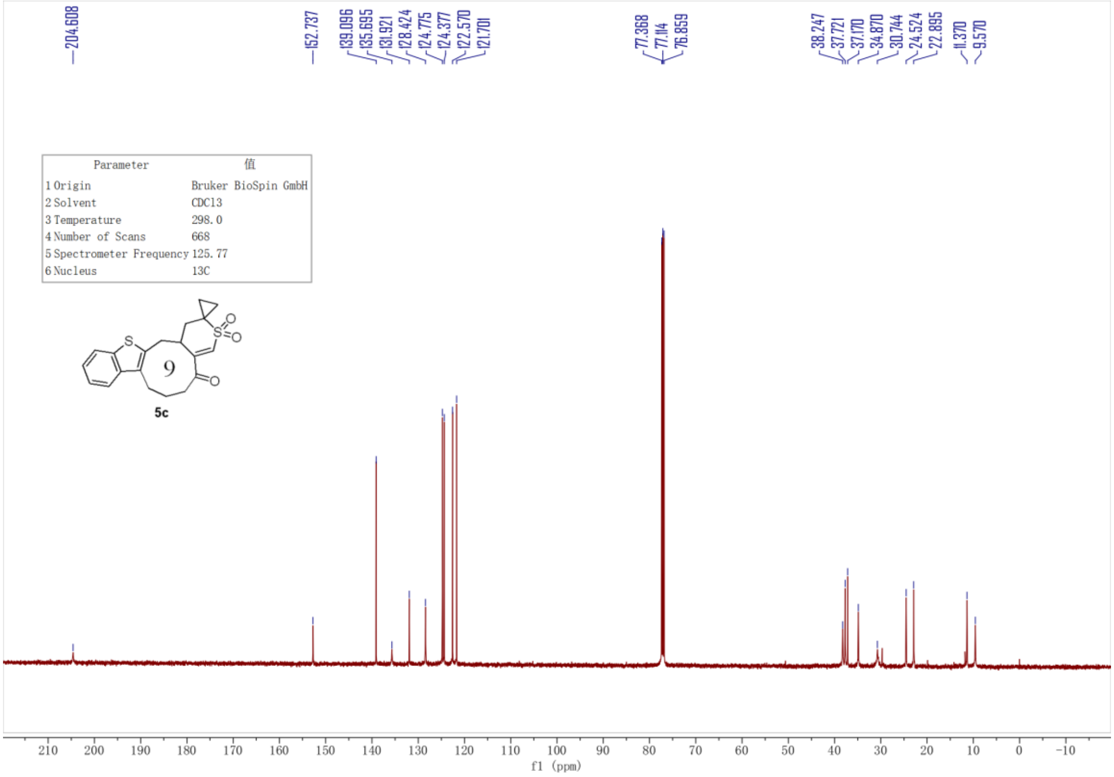
Parameter
值
1 Origin
Bruker BioSpin GmbH
2 Solvent
CDCl3
3 Temperature
298.0
4 Number of Scans
668
5 Spectrometer Frequency
125.77
6 Nucleus
13C
5c
f1 (ppm)

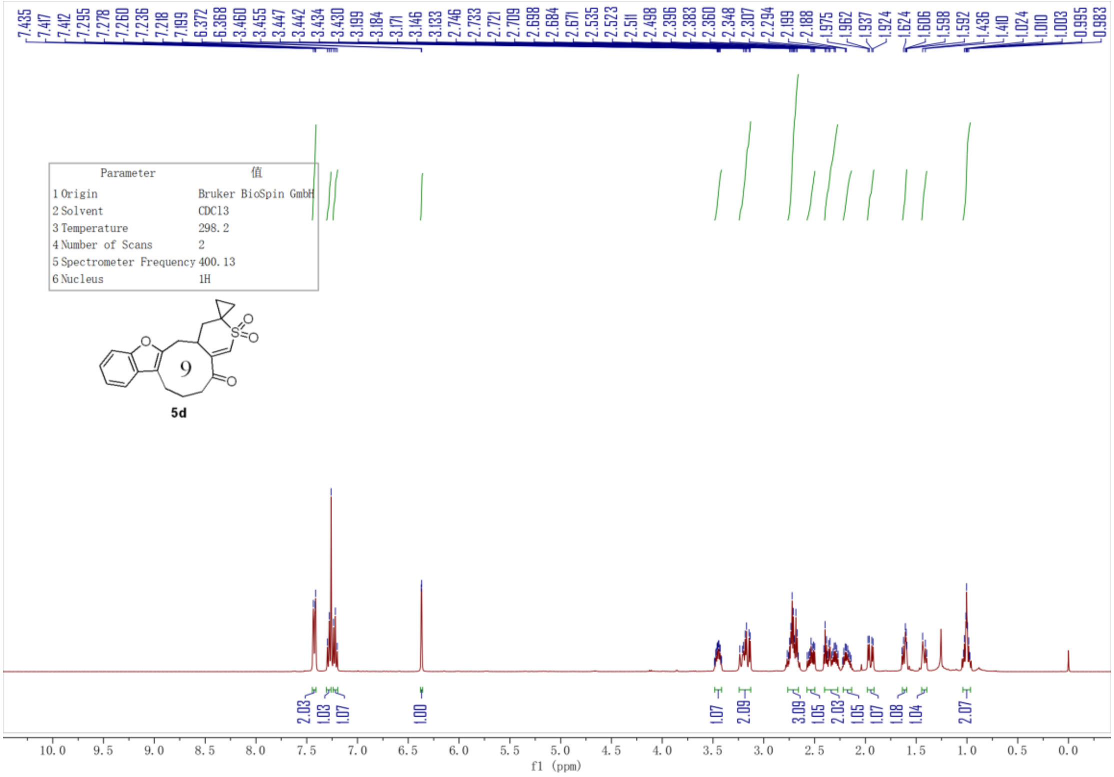
Parameter
值
1 Origin
Bruker BioSpin GmbH
2 Solvent
CDCl3
3 Temperature
298.2
4 Number of Scans
2
5 Spectrometer Frequency
400.13
6 Nucleus
1H
5d
f1 (ppm)

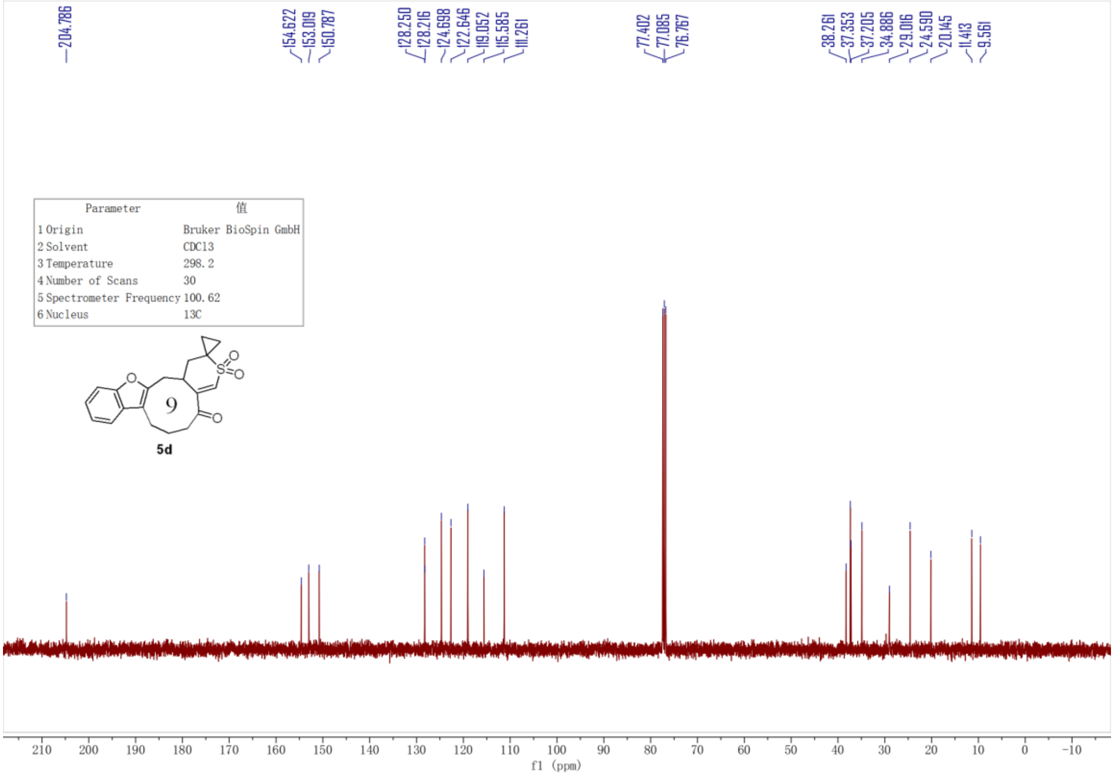
Parameter 值
1 Origin Bruker BioSpin GmbH
2 Solvent CDCl3
3 Temperature 298.2
4 Number of Scans 30
5 Spectrometer Frequency 100.62
6 Nucleus 13C
5d
f1 (ppm)

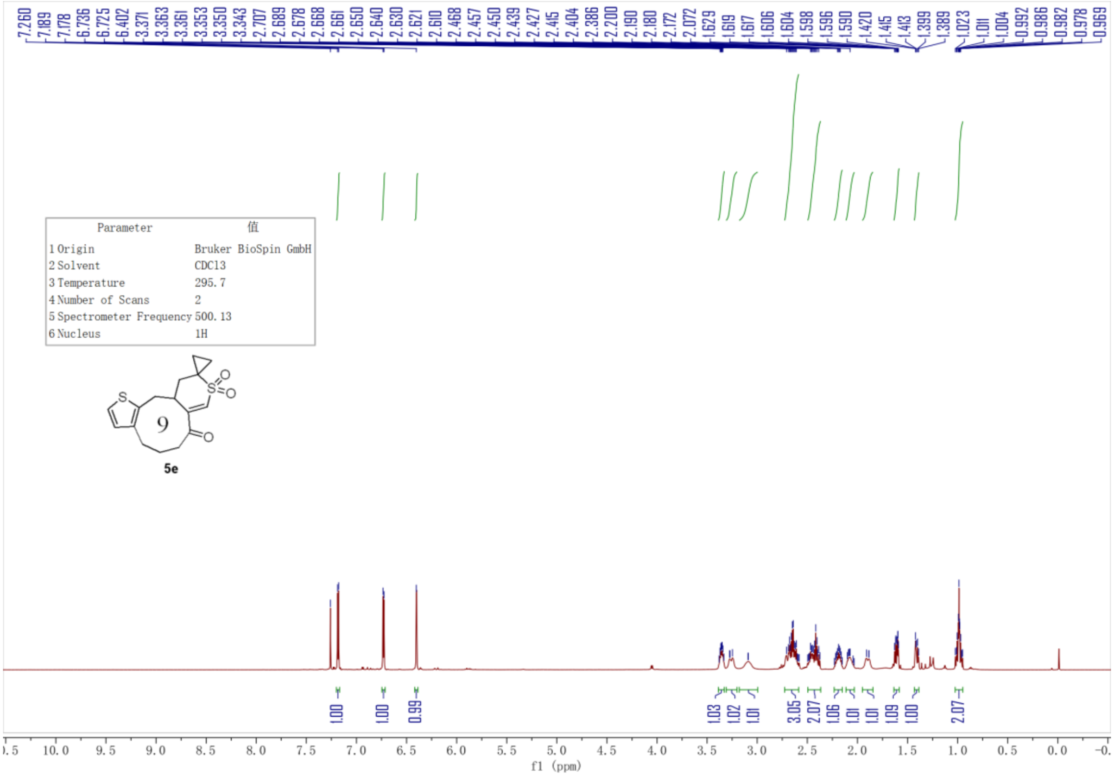
Parameter 值
1 Origin Bruker BioSpin GmbH
2 Solvent CDCl3
3 Temperature 295.7
4 Number of Scans 2
5 Spectrometer Frequency 500.13
6 Nucleus 1H
5e
f1 (ppm)

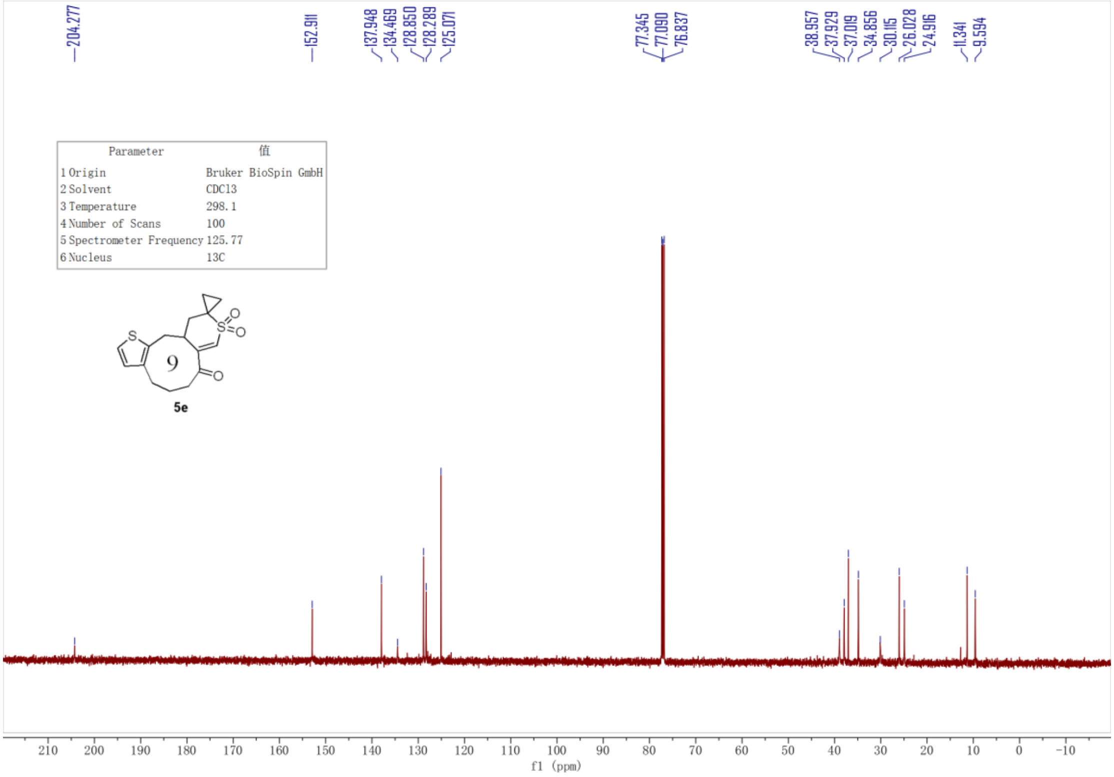
204.277
152.911
137.948
134.469
128.850
128.289
125.071
77.345
77.090
76.837
38.957
37.929
37.019
34.856
30.115
26.028
24.916
11.341
9.594
Parameter 值
1 Origin Bruker BioSpin GmbH
2 Solvent CDCl3
3 Temperature 298.1
4 Number of Scans 100
5 Spectrometer Frequency 125.77
6 Nucleus 13C
S
9
O
S
O
O
5e
210 200 190 180 170 160 150 140 130 120 110 100 90 80 70 60 50 40 30 20 10 0 -10
f1 (ppm)

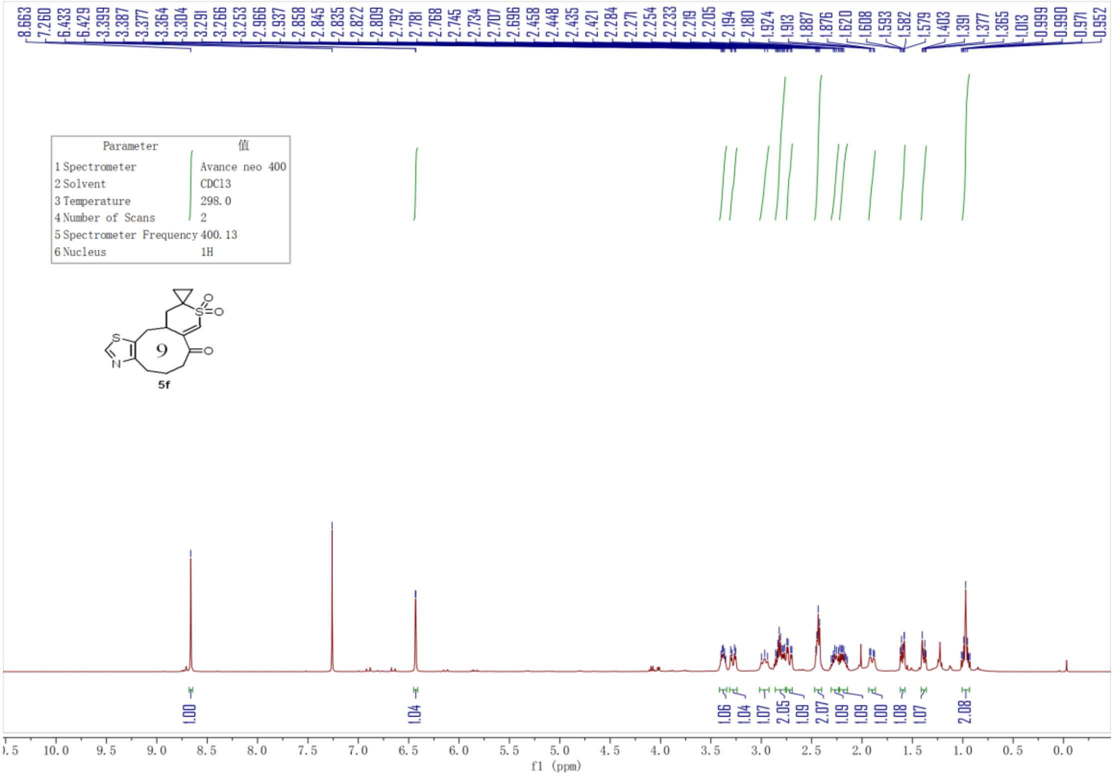
8.663
7.260
6.433
6.429
3.399
3.387
3.377
3.364
3.304
3.291
3.266
3.253
2.966
2.937
2.858
2.845
2.835
2.822
2.809
2.792
2.781
2.768
2.745
2.734
2.707
2.696
2.458
2.448
2.435
2.421
2.284
2.271
2.254
2.233
2.219
2.205
2.194
2.180
1.924
1.913
1.887
1.876
1.620
1.608
1.593
1.582
1.579
1.403
1.391
1.377
1.365
1.013
0.999
0.990
0.971
0.952
Parameter 值
1 Spectrometer Avance neo 400
2 Solvent CDCl3
3 Temperature 298.0
4 Number of Scans 2
5 Spectrometer Frequency 400.13
6 Nucleus 1H
S
N
9
O
S
O
O
5f
1.00
1.04
1.06
1.04
1.07
2.05
1.09
2.07
1.09
1.09
1.00
1.08
1.07
2.08
10.0 9.5 9.0 8.5 8.0 7.5 7.0 6.5 6.0 5.5 5.0 4.5 4.0 3.5 3.0 2.5 2.0 1.5 1.0 0.5 0.0
f1 (ppm)

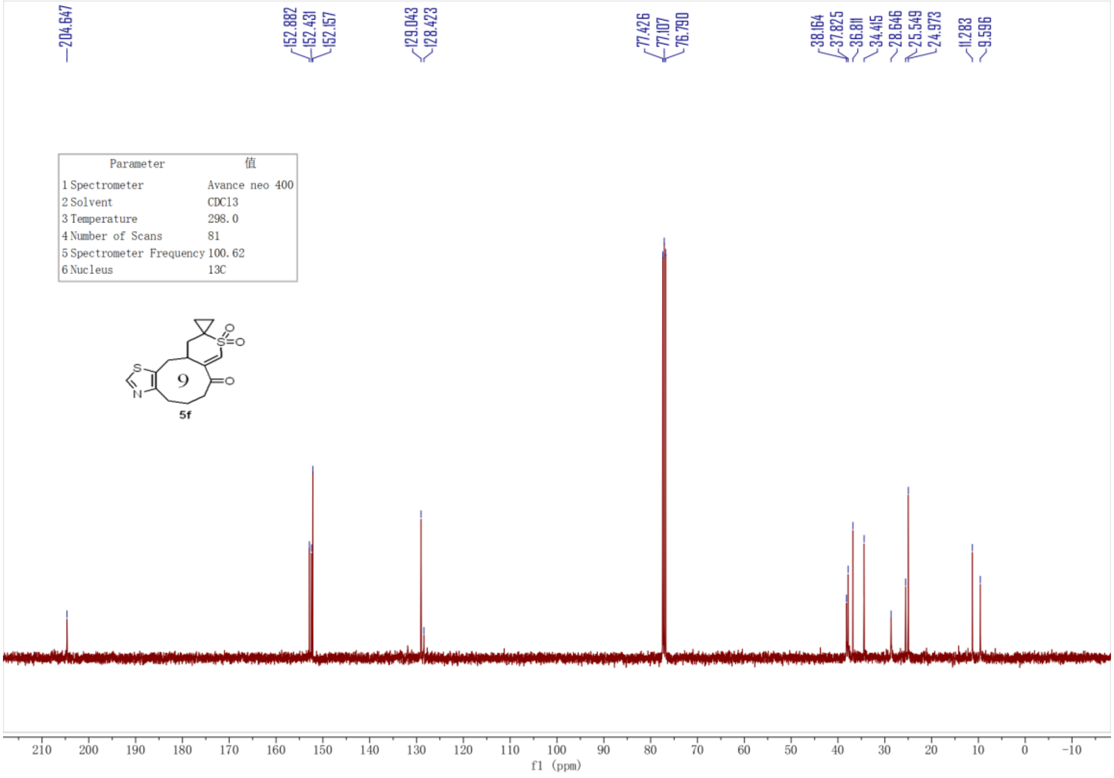
204.647
152.882
152.431
152.157
129.043
128.423
77.426
77.107
76.790
38.164
37.825
36.811
34.415
28.646
25.549
24.973
11.283
9.596
Parameter 值
1 Spectrometer Avance neo 400
2 Solvent CDCl3
3 Temperature 298.0
4 Number of Scans 81
5 Spectrometer Frequency 100.62
6 Nucleus 13C
5f
f1 (ppm)

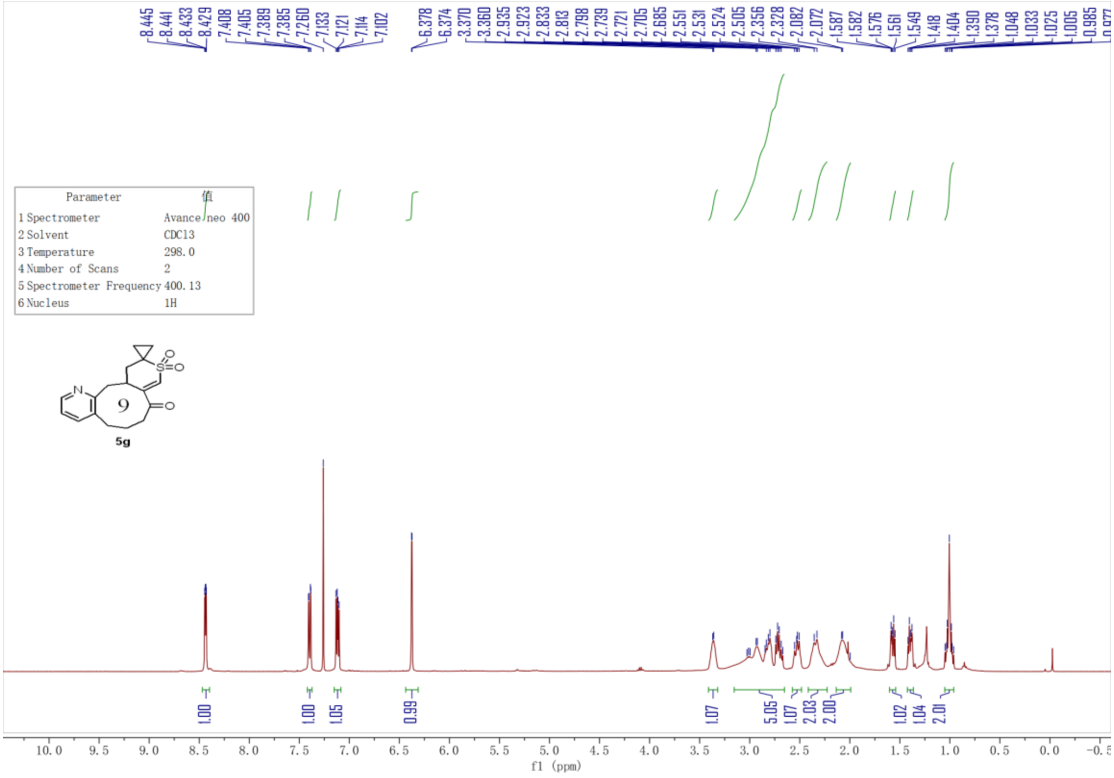
8.445
8.441
8.433
8.429
7.408
7.405
7.389
7.385
7.260
7.133
7.121
7.114
7.102
6.378
6.374
3.370
3.360
2.935
2.923
2.833
2.813
2.798
2.739
2.721
2.705
2.685
2.551
2.531
2.524
2.505
2.356
2.328
2.082
2.072
1.587
1.582
1.576
1.561
1.549
1.418
1.404
1.390
1.378
1.048
1.033
1.025
1.005
0.985
Parameter 值
1 Spectrometer Avance neo 400
2 Solvent CDCl3
3 Temperature 298.0
4 Number of Scans 2
5 Spectrometer Frequency 400.13
6 Nucleus 1H
5g
1.00
1.00
1.05
0.99
1.07
5.05
1.07
2.03
2.00
1.02
1.04
2.01
f1 (ppm)

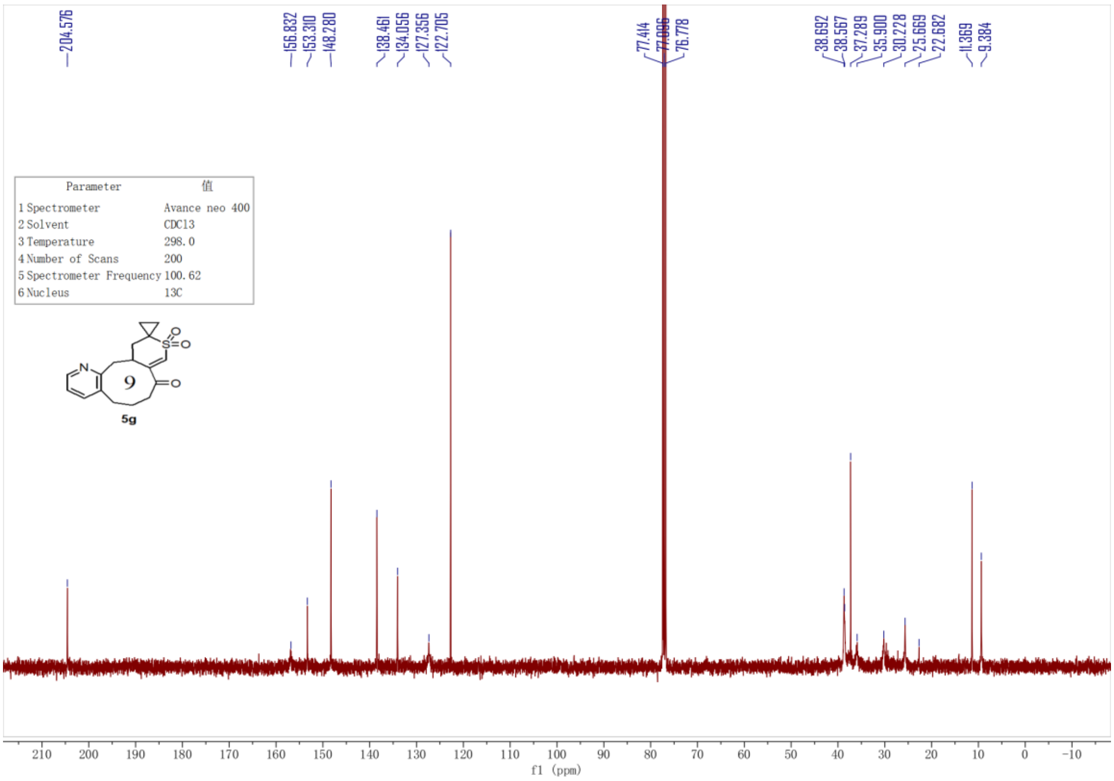
204.576
156.832
153.310
148.280
138.461
134.056
127.356
122.705
77.414
77.096
76.778
38.692
38.567
37.289
35.900
30.228
25.669
22.682
11.369
9.384
Parameter 值
1 Spectrometer Avance neo 400
2 Solvent CDCl3
3 Temperature 298.0
4 Number of Scans 200
5 Spectrometer Frequency 100.62
6 Nucleus 13C
N
9
S
O
5g
f1 (ppm)

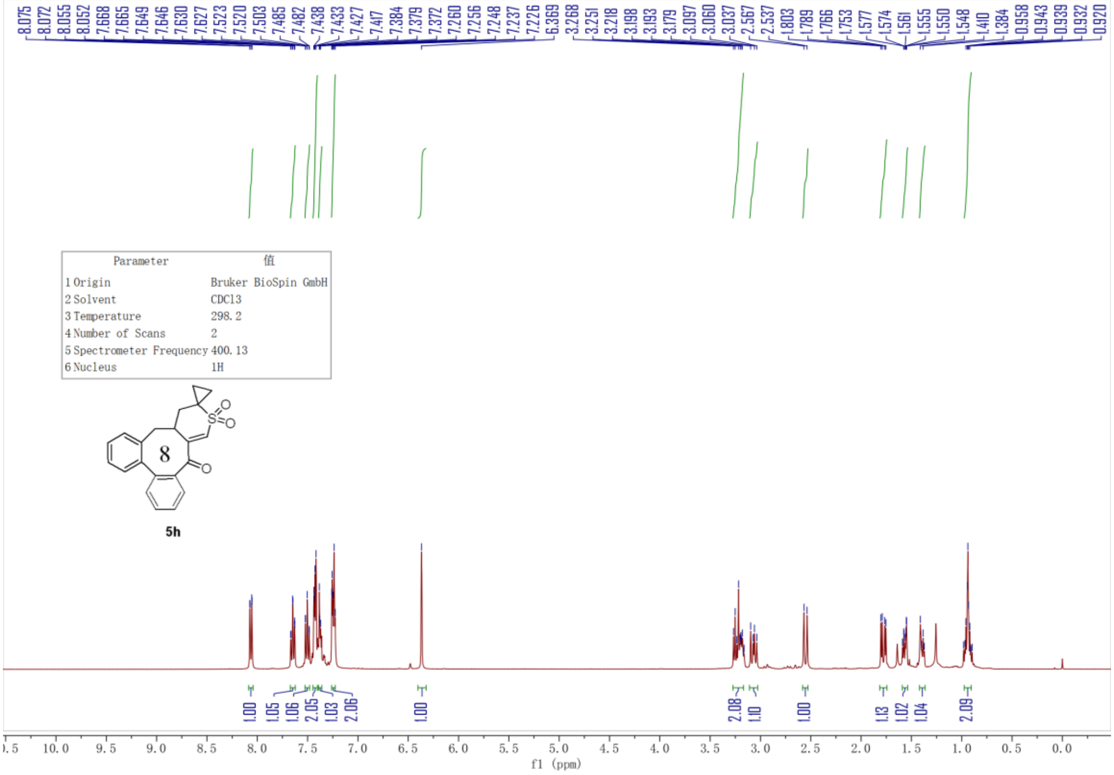
8.075
8.072
8.055
8.052
7.668
7.665
7.649
7.646
7.630
7.627
7.523
7.520
7.503
7.485
7.482
7.438
7.433
7.427
7.417
7.384
7.379
7.372
7.260
7.256
7.248
7.237
7.226
6.369
3.268
3.251
3.218
3.198
3.193
3.179
3.097
3.060
3.037
2.567
2.537
1.803
1.789
1.766
1.753
1.577
1.574
1.561
1.555
1.550
1.548
1.410
1.384
0.958
0.943
0.939
0.932
0.920
Parameter 值
1 Origin Bruker BioSpin GmbH
2 Solvent CDCl3
3 Temperature 298.2
4 Number of Scans 2
5 Spectrometer Frequency 400.13
6 Nucleus 1H
8
S
O
5h
1.00
1.05
1.06
2.05
1.03
2.06
1.00
2.08
1.10
1.00
1.13
1.02
1.04
2.09
f1 (ppm)

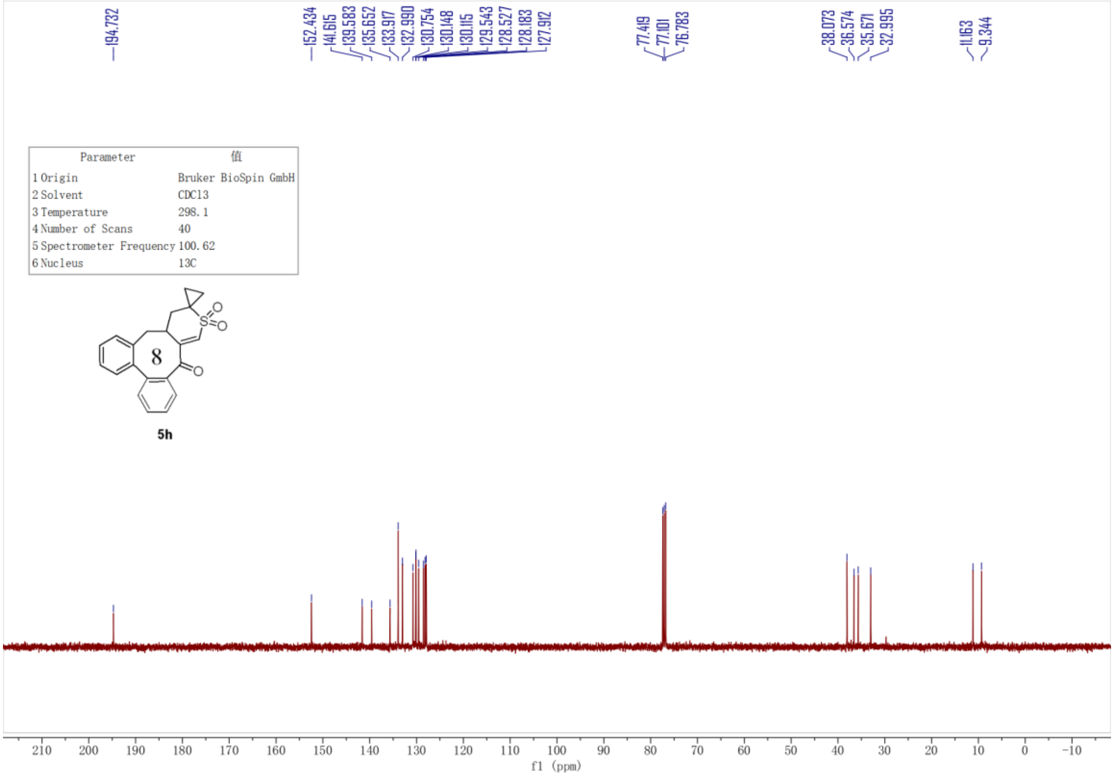
194.732
152.434
141.615
139.583
135.652
133.917
132.990
130.754
130.148
130.115
129.543
128.527
128.183
127.912
77.419
77.101
76.783
38.073
36.574
35.671
32.995
11.163
9.344
Parameter 值
1 Origin Bruker BioSpin GmbH
2 Solvent CDCl3
3 Temperature 298.1
4 Number of Scans 40
5 Spectrometer Frequency 100.62
6 Nucleus 13C
8
5h
f1 (ppm)

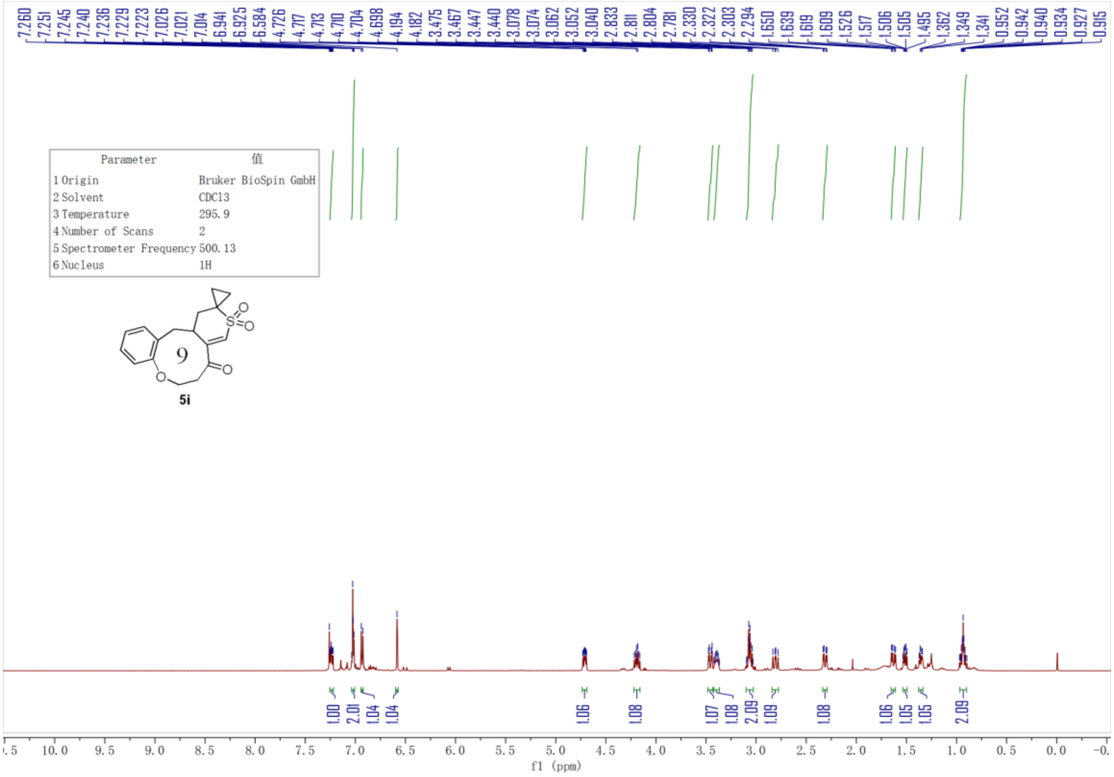
7.260
7.251
7.245
7.240
7.236
7.229
7.223
7.026
7.021
7.014
6.941
6.925
6.584
4.726
4.717
4.713
4.710
4.704
4.698
4.194
4.182
3.475
3.467
3.447
3.440
3.078
3.074
3.062
3.052
3.040
2.833
2.811
2.804
2.781
2.330
2.322
2.303
2.294
1.650
1.639
1.619
1.609
1.526
1.517
1.506
1.505
1.495
1.362
1.349
1.341
0.952
0.942
0.940
0.934
0.927
0.915
Parameter 值
1 Origin Bruker BioSpin GmbH
2 Solvent CDCl3
3 Temperature 295.9
4 Number of Scans 2
5 Spectrometer Frequency 500.13
6 Nucleus 1H
9
5i
1.00
2.01
1.04
1.04
1.06
1.08
1.07
1.08
2.09
1.09
1.08
1.06
1.05
1.05
2.09
f1 (ppm)

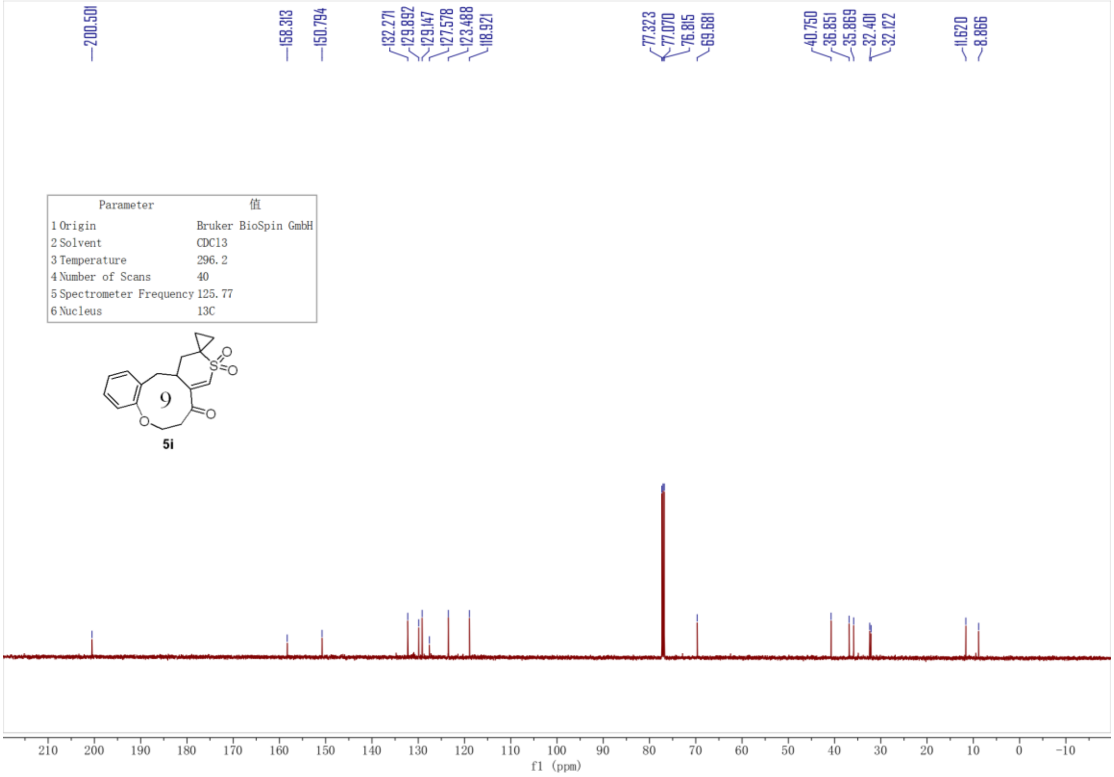
200.501
158.313
150.794
132.271
129.892
129.147
127.578
123.488
118.921
77.323
77.070
76.815
69.681
40.750
36.851
35.869
32.401
32.122
11.620
8.866
Parameter 值
1 Origin Bruker BioSpin GmbH
2 Solvent CDCl3
3 Temperature 296.2
4 Number of Scans 40
5 Spectrometer Frequency 125.77
6 Nucleus 13C
5i
f1 (ppm)

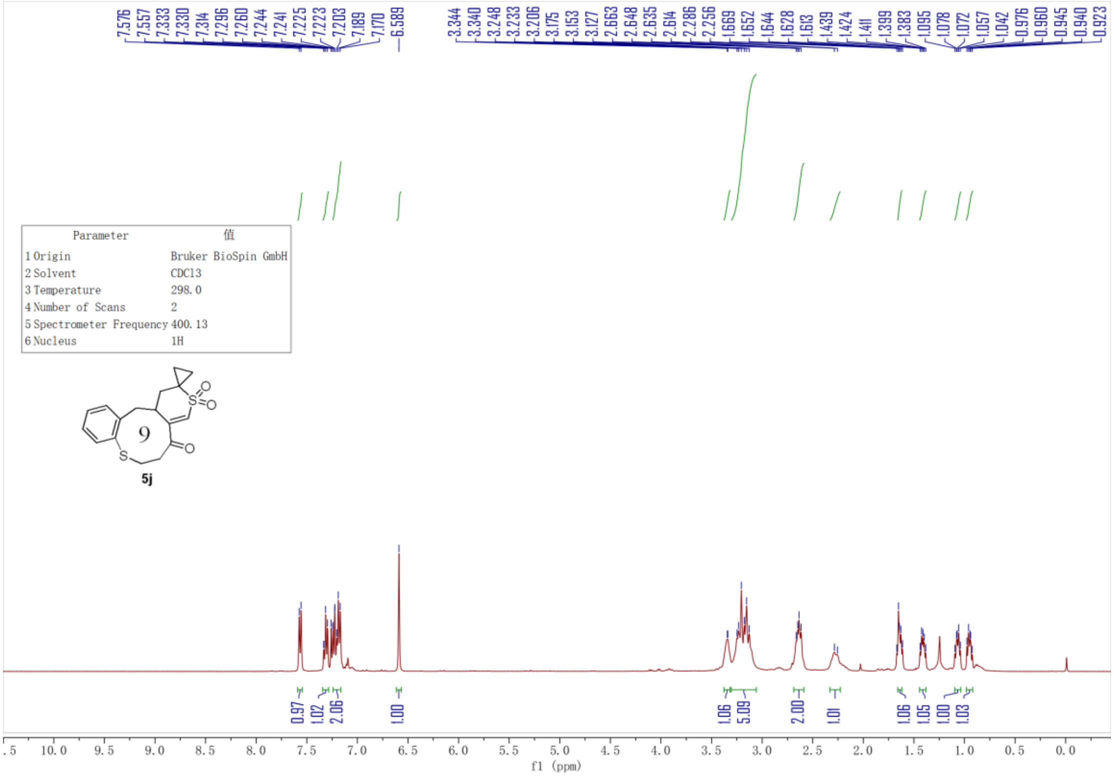
7.576
7.557
7.333
7.330
7.314
7.296
7.260
7.244
7.241
7.225
7.223
7.203
7.189
7.170
6.589
3.344
3.340
3.248
3.233
3.206
3.175
3.153
3.127
2.663
2.648
2.635
2.614
2.286
2.256
1.669
1.652
1.644
1.628
1.613
1.439
1.424
1.411
1.399
1.383
1.095
1.078
1.072
1.057
1.042
0.976
0.960
0.945
0.940
0.923
Parameter 值
1 Origin Bruker BioSpin GmbH
2 Solvent CDCl3
3 Temperature 298.0
4 Number of Scans 2
5 Spectrometer Frequency 400.13
6 Nucleus 1H
5j
0.97
1.02
2.06
1.00
1.06
5.09
2.00
1.01
1.06
1.05
1.00
1.03
f1 (ppm)

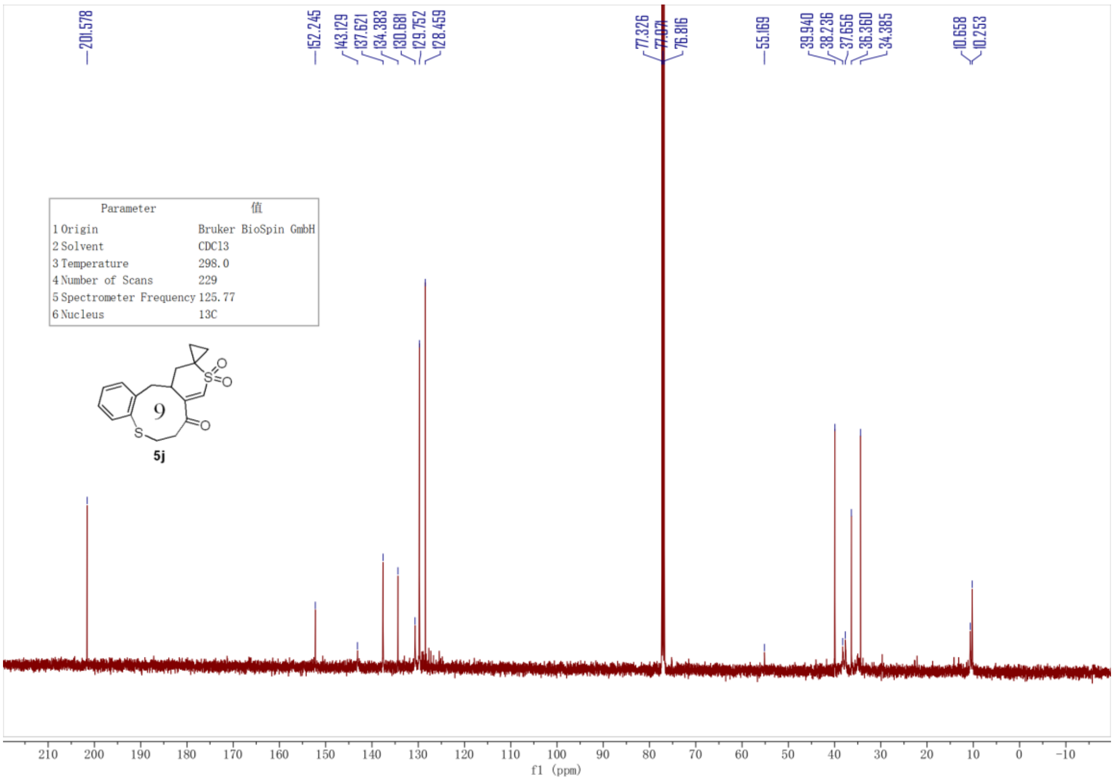

Parameter 值
1 Origin Bruker BioSpin GmbH
2 Solvent CDCl3
3 Temperature 298.0
4 Number of Scans 229
5 Spectrometer Frequency 125.77
6 Nucleus 13C
5j
201.578
152.245
143.129
137.621
134.383
130.681
129.752
128.459
77.326
77.071
76.816
55.169
39.940
38.236
37.656
36.360
34.385
10.658
10.253
f1 (ppm)

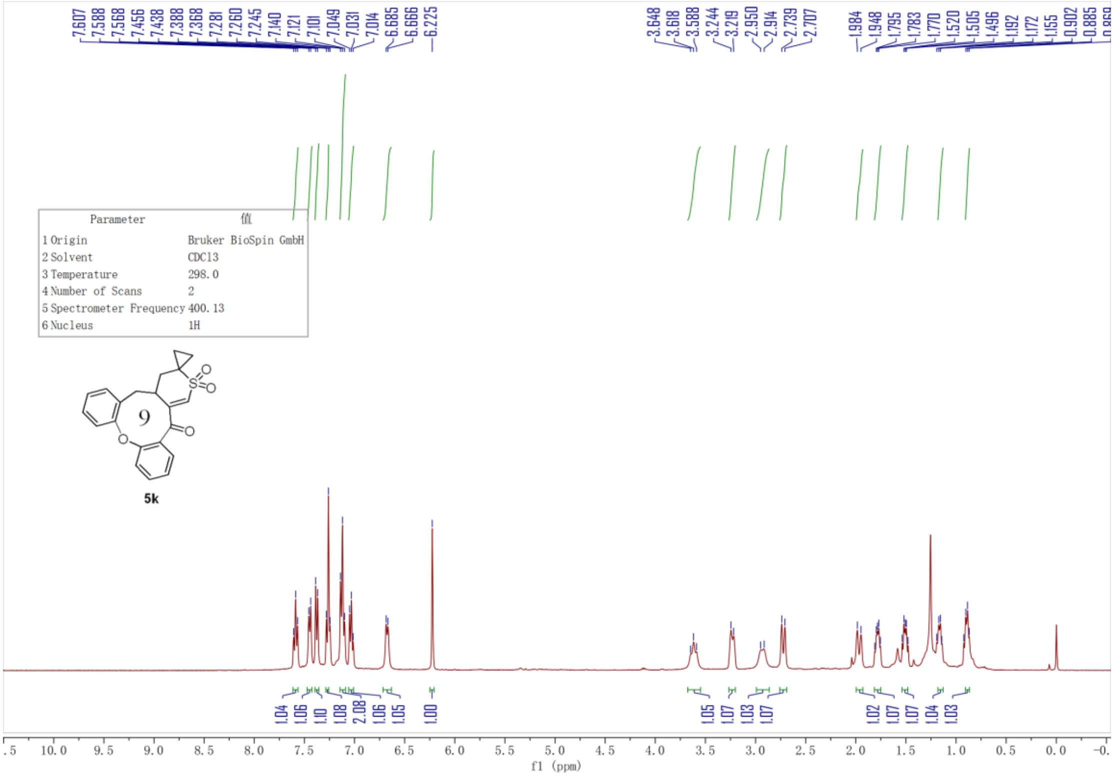

Parameter 值
1 Origin Bruker BioSpin GmbH
2 Solvent CDCl3
3 Temperature 298.0
4 Number of Scans 2
5 Spectrometer Frequency 400.13
6 Nucleus 1H
5k
1.04
1.06
1.10
1.08
2.08
1.06
1.05
1.00
1.05
1.07
1.03
1.07
1.02
1.07
1.07
1.04
1.03
f1 (ppm)

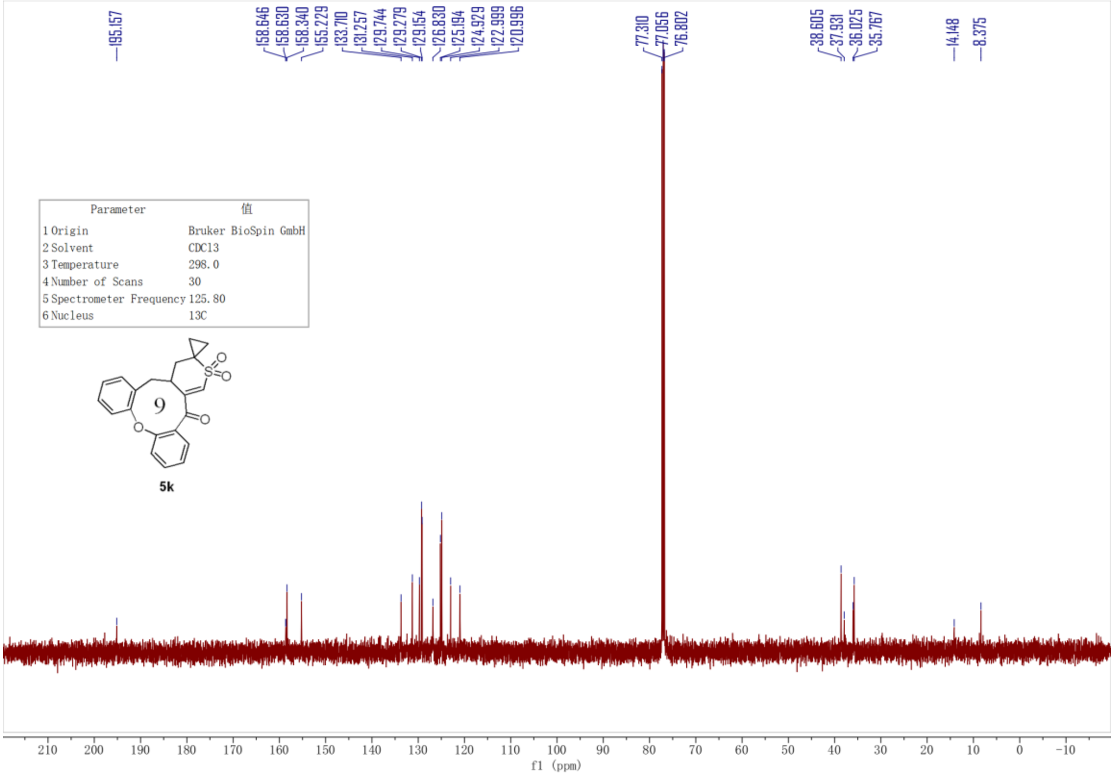
195.157
158.646 158.630 158.340 155.229 133.710 131.257 129.744 129.279 129.154 126.830 125.194 124.929 122.999 120.996
77.310 77.056 76.802
38.605 37.931 36.025 35.767
14.148
8.375
Parameter 值
1 Origin Bruker BioSpin GmbH
2 Solvent CDCl3
3 Temperature 298.0
4 Number of Scans 30
5 Spectrometer Frequency 125.80
6 Nucleus 13C
9
5k
210 200 190 180 170 160 150 140 130 120 110 100 90 80 70 60 50 40 30 20 10 0 -10
f1 (ppm)

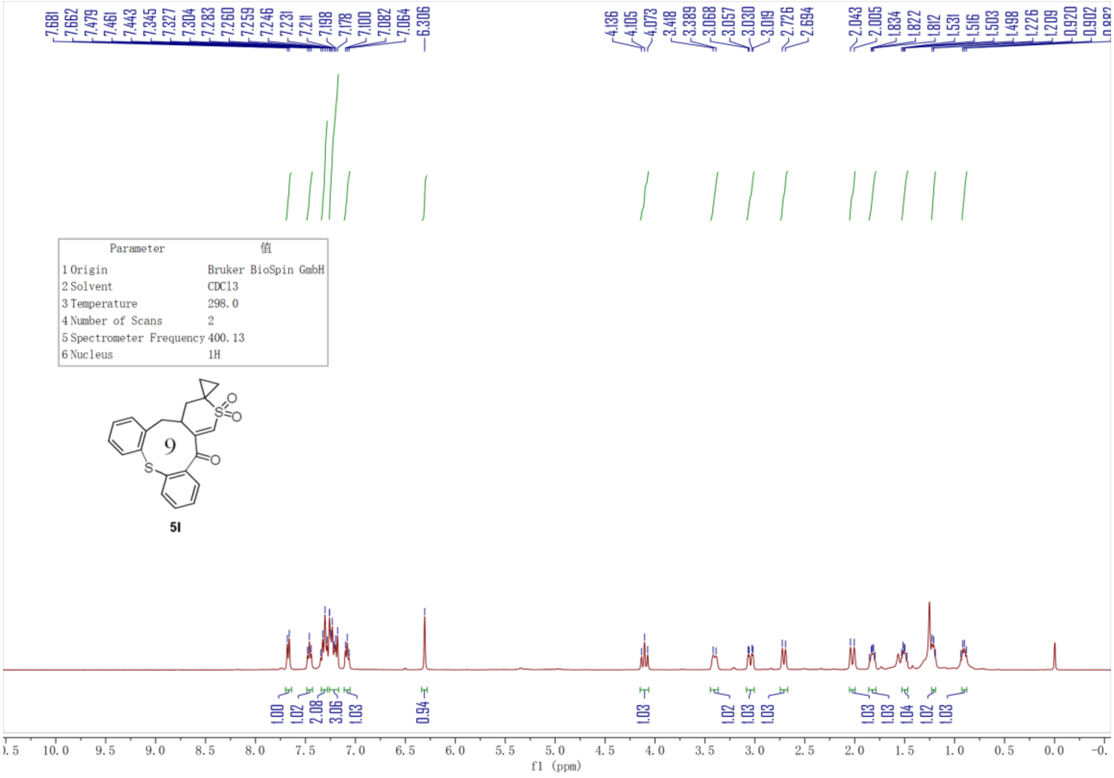
7.681 7.662 7.479 7.461 7.443 7.345 7.327 7.304 7.283 7.260 7.259 7.246 7.231 7.211 7.198 7.178 7.100 7.082 7.064
6.306
4.136 4.105 4.073 3.418 3.389 3.068 3.057 3.030 3.019 2.726 2.694
2.043 2.005 1.834 1.822 1.812 1.531 1.516 1.503 1.498 1.226 1.209 0.920 0.902
Parameter 值
1 Origin Bruker BioSpin GmbH
2 Solvent CDCl3
3 Temperature 298.0
4 Number of Scans 2
5 Spectrometer Frequency 400.13
6 Nucleus 1H
9
5l
1.00 1.02 2.08 3.06 1.03 0.94 1.03 1.02 1.03 1.03 1.03 1.03 1.04 1.02 1.03
10.0 9.5 9.0 8.5 8.0 7.5 7.0 6.5 6.0 5.5 5.0 4.5 4.0 3.5 3.0 2.5 2.0 1.5 1.0 0.5 0.0
f1 (ppm)

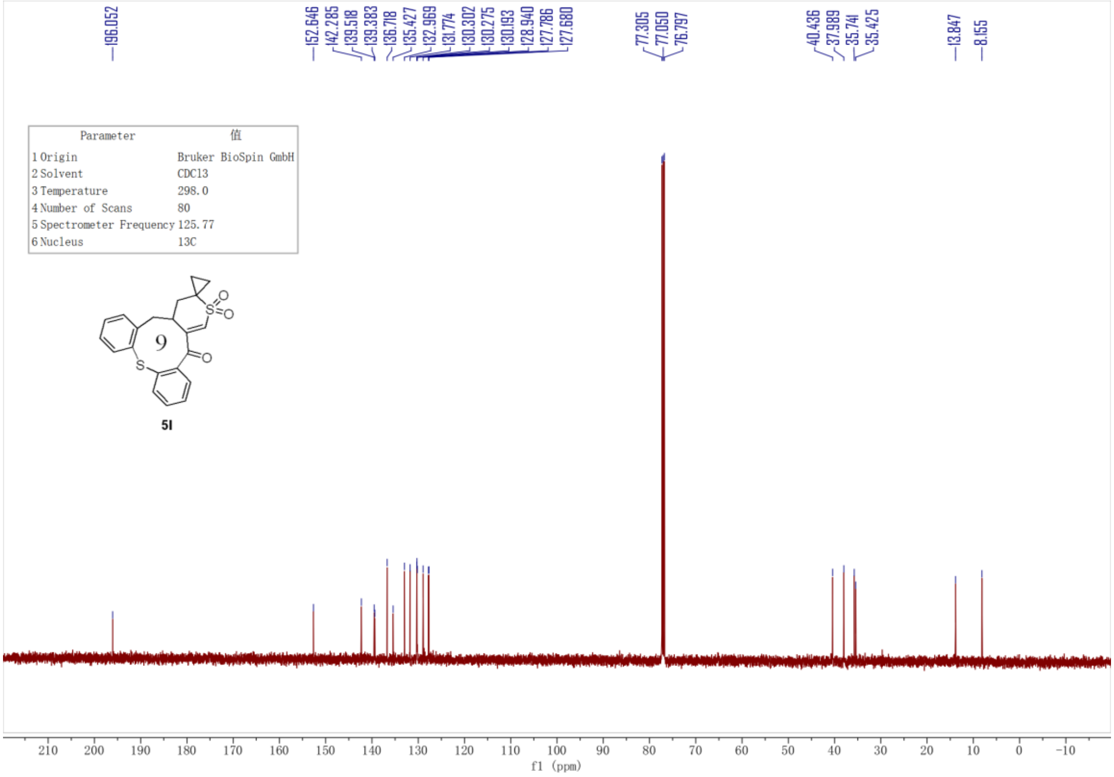

Parameter
值
1 Origin
Bruker BioSpin GmbH
2 Solvent
CDCl3
3 Temperature
298.0
4 Number of Scans
80
5 Spectrometer Frequency
125.77
6 Nucleus
13C
f1 (ppm)

5l

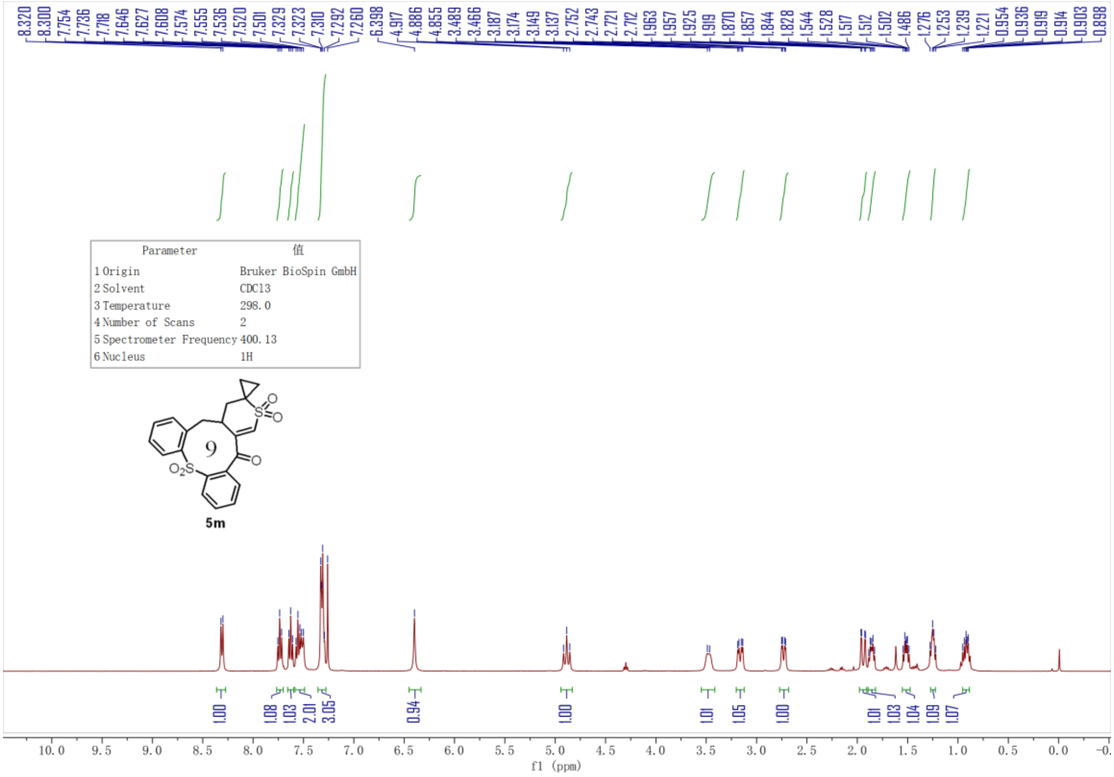

Parameter
值
1 Origin
Bruker BioSpin GmbH
2 Solvent
CDCl3
3 Temperature
298.0
4 Number of Scans
2
5 Spectrometer Frequency
400.13
6 Nucleus
1H
f1 (ppm)

5m

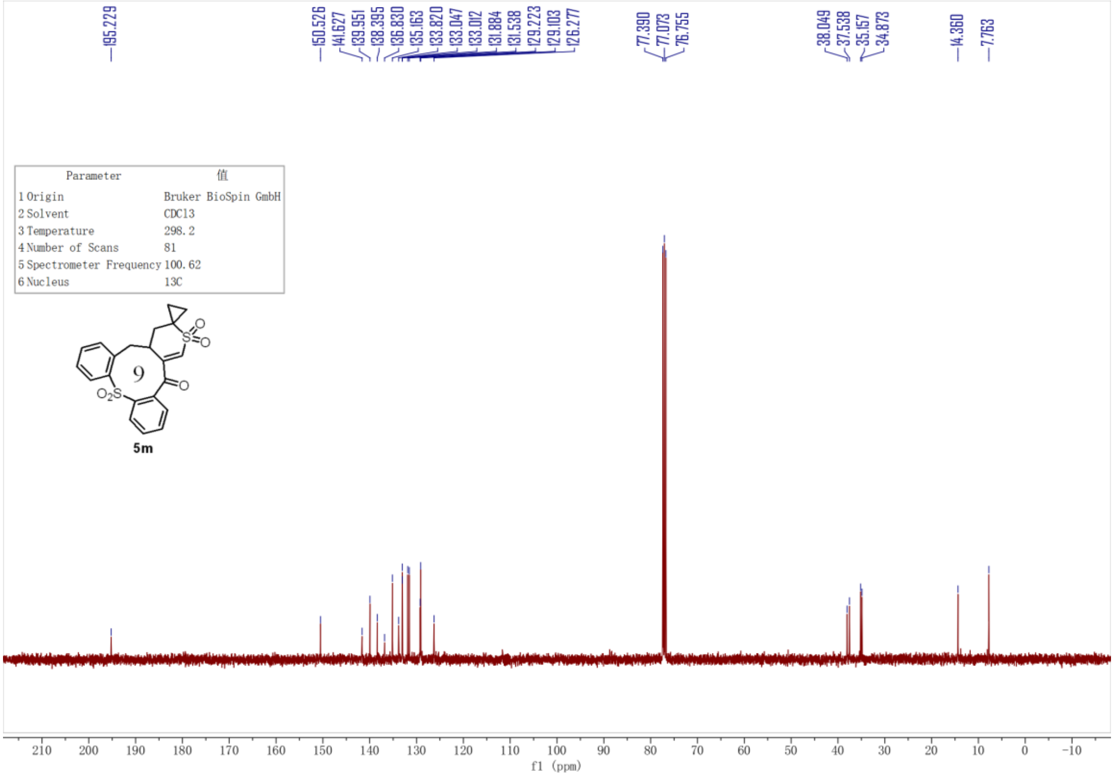
195.229
150.526
141.627
139.951
138.395
136.830
135.163
133.820
133.047
133.012
131.884
131.538
129.223
129.103
126.277
77.390
77.073
76.755
38.049
37.538
35.157
34.873
14.360
7.763
Parameter 值
1 Origin Bruker BioSpin GmbH
2 Solvent CDCl3
3 Temperature 298.2
4 Number of Scans 81
5 Spectrometer Frequency 100.62
6 Nucleus 13C
5m
f1 (ppm)

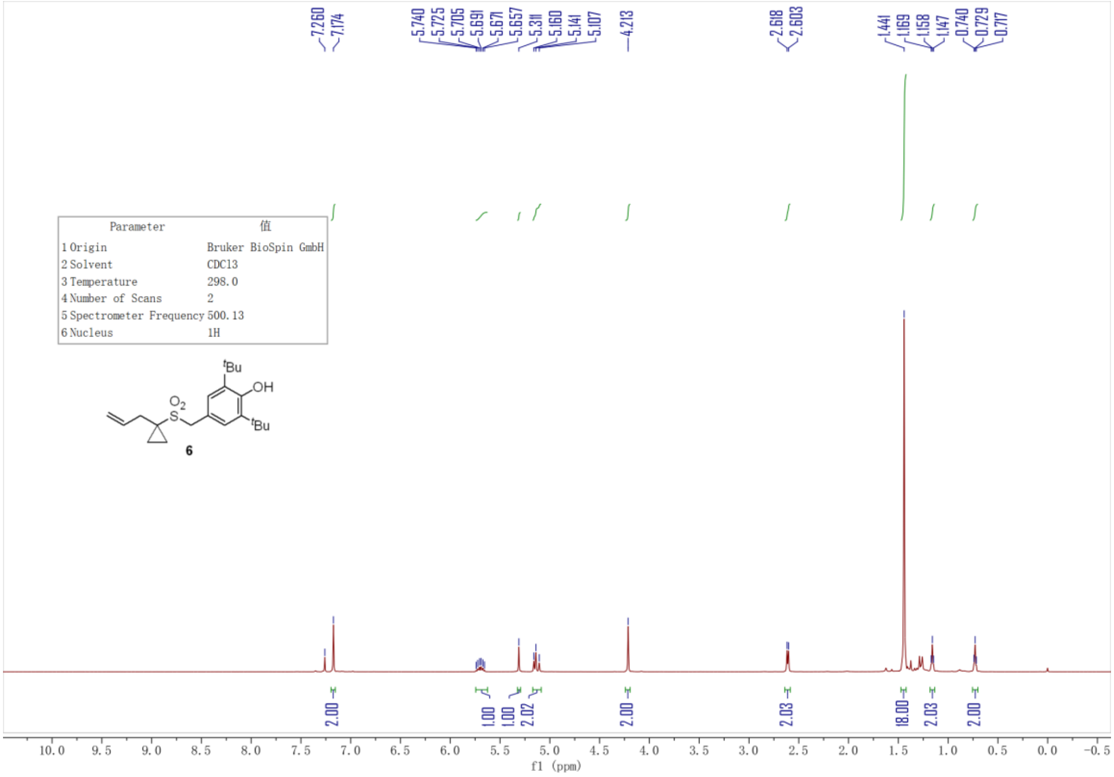
7.260
7.174
5.740
5.725
5.705
5.691
5.671
5.657
5.311
5.160
5.141
5.107
4.213
2.618
2.603
1.441
1.169
1.158
1.147
0.740
0.729
0.717
Parameter 值
1 Origin Bruker BioSpin GmbH
2 Solvent CDCl3
3 Temperature 298.0
4 Number of Scans 2
5 Spectrometer Frequency 500.13
6 Nucleus 1H
6
2.00
1.00
1.00
2.02
2.00
2.03
18.00
2.03
2.00
f1 (ppm)

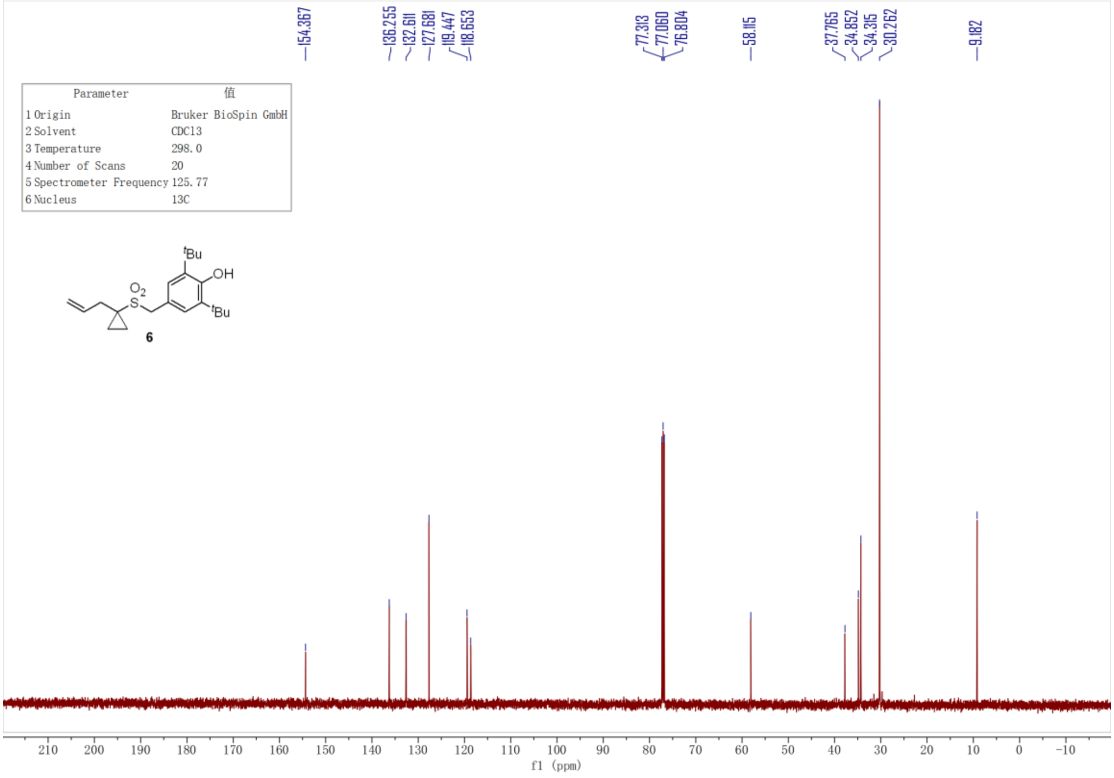

—154.367
136.255
132.611
127.681
119.447
118.653
77.313
77.060
76.804
—58.115
37.765
34.852
34.315
30.262
—9.182
Parameter 值
1 Origin Bruker BioSpin GmbH
2 Solvent CDCl3
3 Temperature 298.0
4 Number of Scans 20
5 Spectrometer Frequency 125.77
6 Nucleus 13C
tBu
OH
O2
S
tBu
6
f1 (ppm)

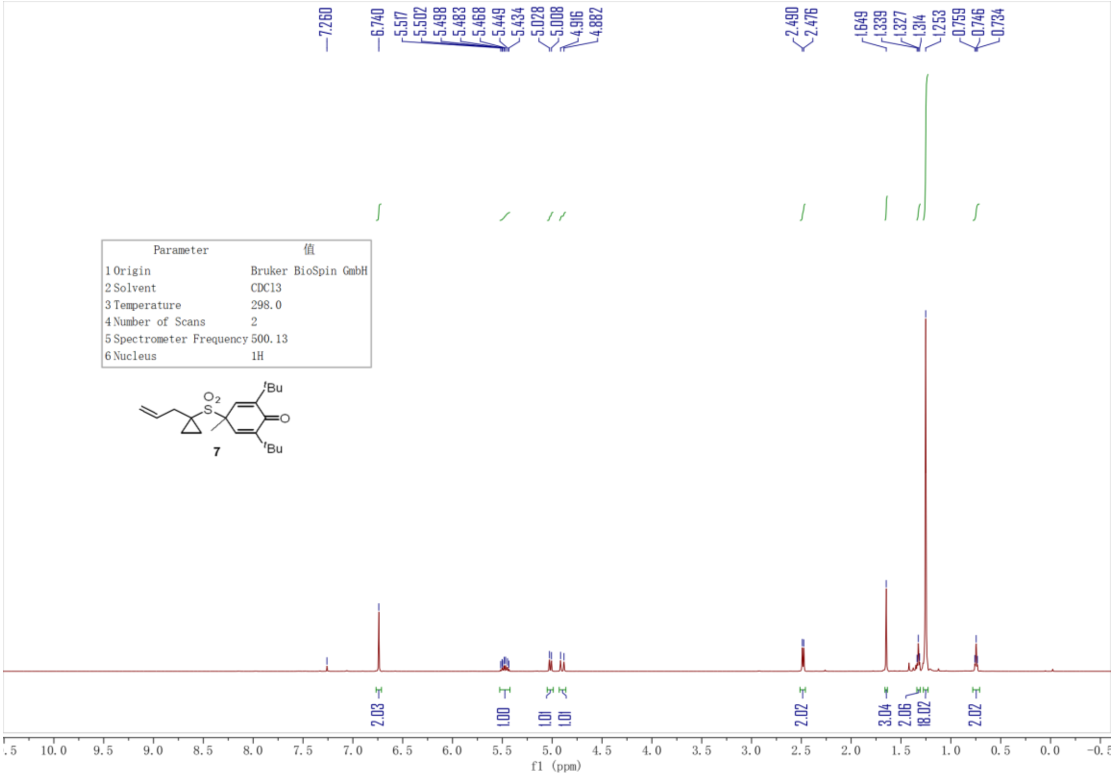

—7.260
—6.740
5.517
5.502
5.498
5.483
5.468
5.449
5.434
5.028
5.008
4.916
4.882
2.490
2.476
1.649
1.339
1.327
1.314
1.253
0.759
0.746
0.734
Parameter 值
1 Origin Bruker BioSpin GmbH
2 Solvent CDCl3
3 Temperature 298.0
4 Number of Scans 2
5 Spectrometer Frequency 500.13
6 Nucleus 1H
O2
S
tBu
O
tBu
7
2.03
1.00
1.01
1.01
2.02
3.04
2.06
18.02
2.02
f1 (ppm)

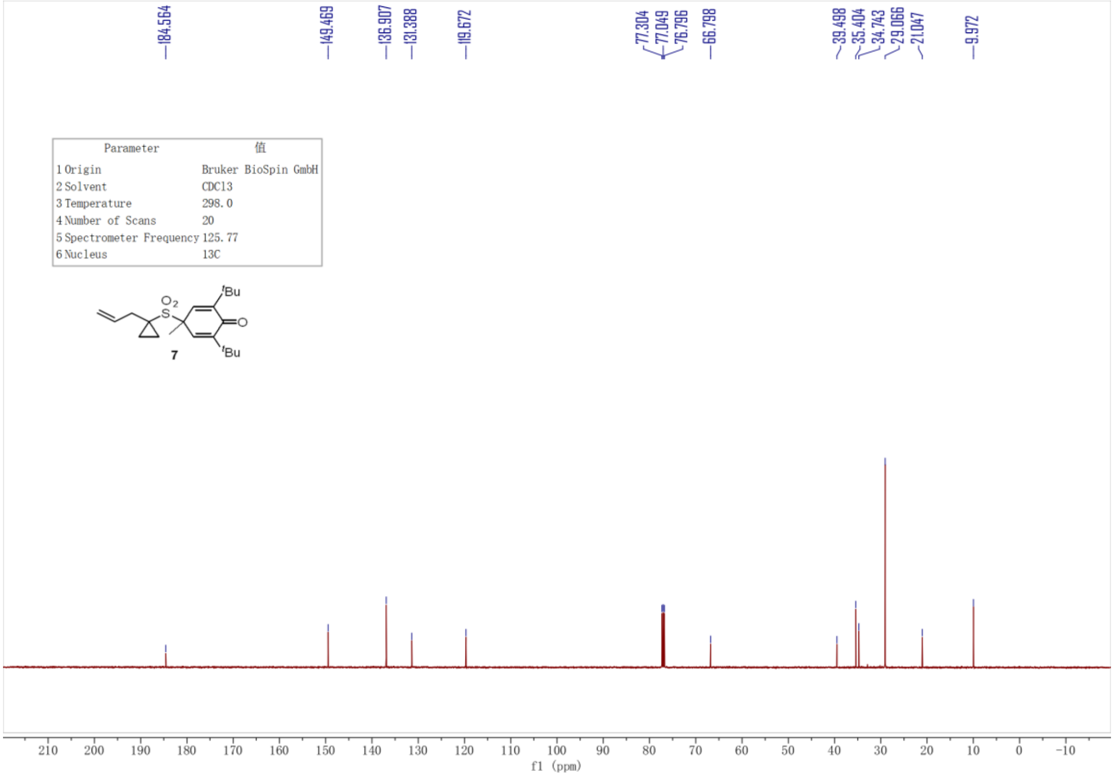
Parameter 值
1 Origin Bruker BioSpin GmbH
2 Solvent CDCl3
3 Temperature 298.0
4 Number of Scans 20
5 Spectrometer Frequency 125.77
6 Nucleus 13C
7
f1 (ppm)

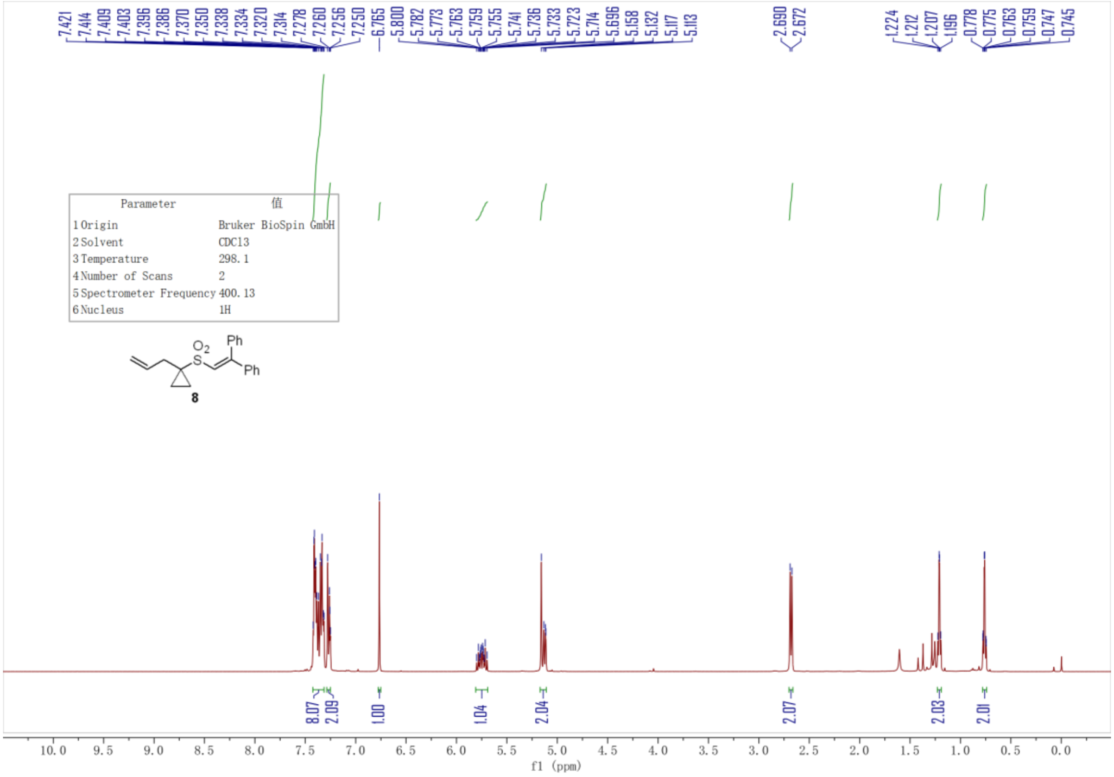
Parameter 值
1 Origin Bruker BioSpin GmbH
2 Solvent CDCl3
3 Temperature 298.1
4 Number of Scans 2
5 Spectrometer Frequency 400.13
6 Nucleus 1H
8
f1 (ppm)

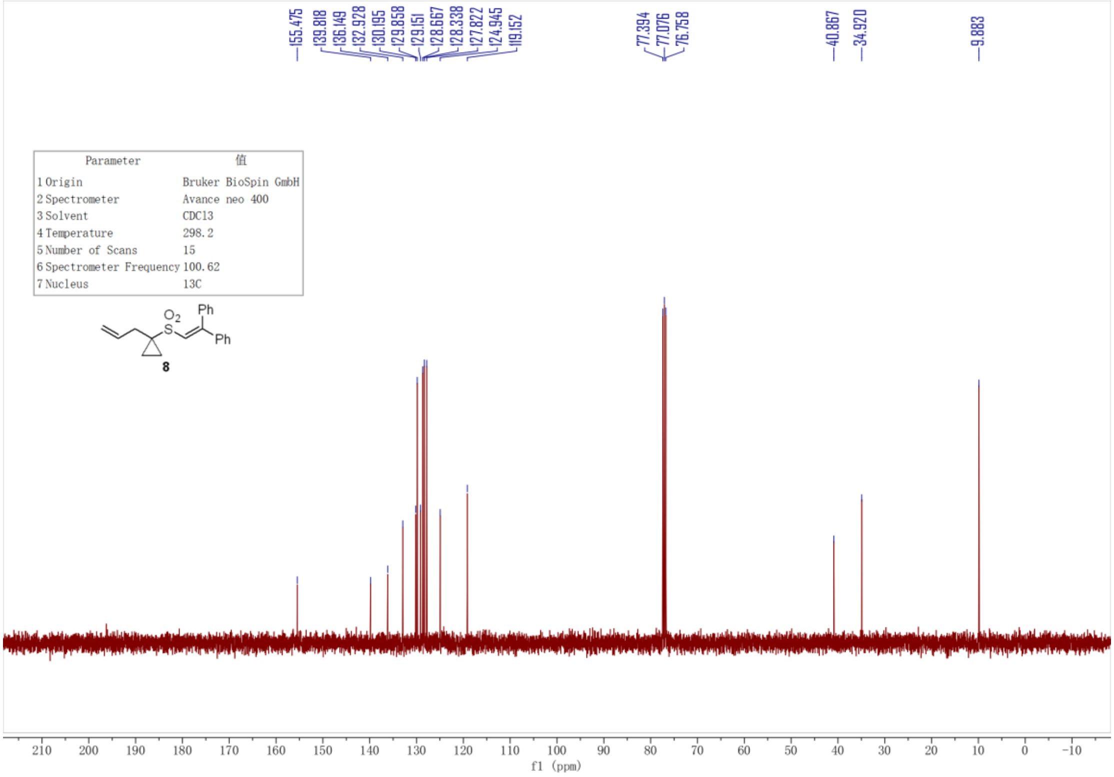
155.475
139.818
136.149
132.928
130.195
129.858
129.151
128.667
128.338
127.822
124.945
119.152
77.394
77.076
76.758
40.867
34.920
9.883
Parameter
值
1 Origin
Bruker BioSpin GmbH
2 Spectrometer
Avance neo 400
3 Solvent
CDCl3
4 Temperature
298.2
5 Number of Scans
15
6 Spectrometer Frequency
100.62
7 Nucleus
13C
8
f1 (ppm)
